# FACE AHEAD 2026 Abstract Report

**DOI:** 10.3390/cmtr19040039

**Published:** 2026-09-23

**Authors:** Yiu Yan Leung, Mohamed Abdelazeem, Narissaporn Chaiprakit, Amir Elbarbary, Susana Heredero-Jung, Gregorio Sánchez-Aniceto

**Affiliations:** 1 Discipline of Oral and Maxillofacial Surgery, Faculty of Dentistry, The University of Hong Kong, Hong Kong SAR, China; 2 Craniomaxillofacial Surgery, Faculty of Medicine, Cairo University, Cairo 11956, Egypt; 3 Division of Oral and Maxillofacial Surgery, Faculty of Dentistry, Thammasat University, Pathum Thani 12120, Thailand; 4 Craniomaxillofacial Unit, Ain Shams University, Abbassia, Cairo 11566, Egypt; 5 Department of Plastic and Reconstructive Surgery, Hospital de la Santa Creu i Sant Pau, 08041 Barcelona, Spain; 6 Department of Oral and Maxillofacial Surgery, Hospital Universitario 12 de Octubre, Avenida de Córdoba, s/n, 28041 Madrid, Spain


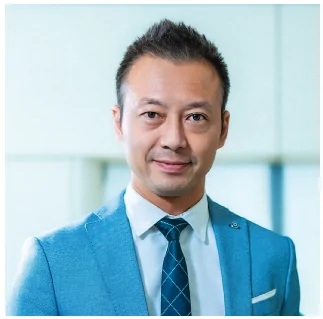


**Prof. Dr. Yiu Yan Leung**

Welcome to FACE AHEAD 2026, organized by AO CMF. FACE AHEAD is designed to be one of the most important gatherings of young surgeons active in the treatment of craniomaxillofacial lesions from all over the world. We are very happy that after the second edition of FACE AHEAD in Prague in 2024, our third summit is taking us to Bangkok on October 1st–3rd, 2026.

FACE AHEAD gives accepted abstract submitters the option to publish their work in this conference report for the *Craniomaxillofacial Trauma and Reconstruction* journal (*CMTR*).

This supplement contains 336 carefully selected abstracts in the following topics:

- Aesthetics;
- Basic and translational research;
- Cleft lip and palate;
- Craniofacial surgery;
- Dental rehabilitation;
- Reconstruction;
- Technology;
- TMJ;
- Trauma;
- Oncology;
- Oral surgery;
- Orthognathic surgery;
- Other areas.

We are convinced that the oral and e-poster presentations, along with the scientific program focusing on bridging the gap between generations and embracing the latest advancements and shaping the future of craniomaxillofacial surgery, will encourage lively discussions among participants and generate new ideas to help advance CMF care. At the same time, they will empower, engage, and inspire participants through meaningful collaboration and knowledge sharing.

Thank you to all abstract submitters for being part of the third edition of FACE AHEAD and for publishing your contributions and presenting the progress of your scientific work onsite in Bangkok.

Mike Leung, Chair.
Mohamed Abdelazeem.
Narissaporn Chaiprakit.
Amir Elbarbary.
Susana Heredero-Jung.
Gregorio Sánchez-Aniceto.
FACE AHEAD Organizing Committee.

## ORAL

**FA26/166—Aesthetics**
**The Fusion Sling–SOON Composite Flap for Three-Dimensional Midfacial Balance in Asian Rhinoplasty: Anatomical Basis and Outcomes in 214 Consecutive Cases**
Takamasa Nakai ^1^
*^1^* *Wom Clinic Ginza, Tokyo, Japan*
**Objectives**: Asian midfacial retrusion—characterized by maxillary hypoplasia and anterior nasal spine deficiency—cannot be corrected by dorsal augmentation alone; implant-based nasal base corrections carry complication rates exceeding 12%. We identify two previously undescribed anatomical structures—the Fusion Sling and Superficial Orbicularis Oris Nasalis (SOON)—and evaluate their combined use as an autologous, motion-preserving composite flap to restore three-dimensional nasal base support and midfacial projection without implants or cartilage harvest.
**Materials and Methods**: From a prospective database of >1400 rhinoplasty cases, 214 consecutive Asian patients (2023–2025) underwent nasal base augmentation using the Fusion Sling–SOON Composite Flap (FSCF). The Fusion Sling—a nondistensible fibrous band along the cephalic border of the lower lateral cartilage—was elevated en bloc with the SOON layer to form a vascularized composite flap anchored at the piriform aperture. Three fixation levels (caudal septal hinge, mid-level SEG, superior SEG) enabled graded, titratable augmentation. Sixty-three patients underwent 3-D stereophotogrammetry (Vectra) pre- and postoperatively (mean follow-up 9.2 months); nasolabial angle and columellar projection were compared using paired *t*-tests.
**Results**: No major complications occurred (0/214, 0%), and complete upper-lip animation was preserved in all patients at final follow-up. Mean nasolabial angle increased from 93.4° ± 4.2° to 101.0° ± 3.8° (Δ + 7.6°, 95% CI 6.8–8.4, *p* < 0.001, n = 63). Columellar projection improved by 1.9 ± 0.6 mm (95% CI 1.7–2.1, *p* < 0.001). Titratable gains by fixation level: mild +3.2°, moderate +7.8°, large +12.4°.
**Conclusion**: FSCF delivers statistically significant, titratable midfacial augmentation with zero complications, no donor-site morbidity, and complete preservation of perioral dynamics—outcomes unachievable with conventional implant or graft-based methods. This technique establishes a reproducible, autologous framework for addressing Asian craniomaxillofacial skeletal imbalance through motion-preserving soft-tissue reconstruction.

**FA26/319—Aesthetics**
**Comparison of One-Piece Versus Two-Piece Repositioning Guides in Osseous Genioplasty: A CT-Based Three-Dimensional Accuracy Study**
Jianfei Zhang ^1^
*^1^* *Department of Oral & Cranio-maxillofacial Surgery, Shanghai Ninth People’s Hospital, Shanghai, China*
**Objectives**: Patient-specific guides for genioplasty typically comprise an osteotomy guide and a subsequent repositioning/fixation guide. While osteotomy guides are largely comparable, repositioning guides differ in anatomic coverage, commonly as a central one-piece configuration or a marginal two-piece configuration. This study compared postoperative three-dimensional accuracy between these designs and tested whether accuracy advantages are region dependent.
**Materials and Methods**: Sixty patients undergoing osseous genioplasty with virtual planning and three-dimensional printed guides were retrospectively analyzed (30 central one-piece; 30 marginal two-piece). Postoperative CT data were superimposed onto the virtual plan using a standardized craniofacial registration workflow. Three-dimensional positional deviation (mm) was quantified at the pogonion (chin point) and bilateral lateral segments (“wings”) and reported as mean ± standard deviation. Intergroup comparisons were performed using two-sided tests (α = 0.05).
**Results**: The one-piece guide achieved significantly higher accuracy at the pogonion (1.25 ± 0.24 mm) compared with the two-piece guide (3.05 ± 0.98 mm; *p* < 0.001). Conversely, the one-piece design demonstrated inferior accuracy at the bilateral wings (left: 2.31 ± 0.54 mm; right: 2.56 ± 0.39 mm) relative to the two-piece design (left: 1.45 ± 0.37 mm; right: 1.38 ± 0.41 mm; both *p* < 0.001). This pattern indicates a consistent region-specific trade-off: central control favored the pogonion, whereas marginal constraint improved bilateral lateral (“wing”) precision.
**Conclusion**: Repositioning guide configuration influences genioplasty accuracy in a region-dependent manner. Central one-piece guides may be preferable when maximizing pogonion precision is the primary objective, while marginal two-piece guides provide superior bilateral wing control and may be advantageous when lateral symmetry and wing positioning are critical.

**FA26/482—Aesthetics**
**The Outcome of the Reconstructive Procedure Using Buccal Pad of Fat Flap and Deep Plane Facelift After Permanent Filler Removal**
Maram Ismail ^1^
*^1^* *Ain Shams University, Cairo, Egypt*
**Objectives**: Deep plane facelift in patients with prior permanent filler injection is a formidable reconstructive and aesthetic procedure. Various reconstructive techniques have been previously described in the literature to address the defects after permanent fillers removal. This study investigates the effectiveness and complication rate of reconstruction of these soft tissue defects using the buccal pad of fat flap.
**Materials and Methods**: This prospective cohort study was conducted at Opal Aesthetic Center in Cairo from 2016 to 2022. Patients underwent removal of the permanent fillers, followed by a deep plane facelift and reconstruction of the resulting defect using a buccal pad of fat flap. Follow up was done for a minimum period of six months to a maximum of five years to assess contour and complications.
**Results**: A study of 151 patients, comprising 92.7% females with a mean age of 47.8 years, examined the outcomes of reconstruction using buccal pad of fat flap following permanent filler removal. Patient satisfaction was high, with 80.7% rating their outcomes as “very good” or “excellent”. Primary surgeries had an 88.5% satisfaction rate, whereas revision surgeries had only a 33.4% satisfaction rate. Contour irregularities were reported in 10.6% of cases and were linked to the occurrence of complications (*p* < 0.001).
**Conclusion**: The buccal fat pad flap combined with a deep plane facelift after permanent filler removal is a safe and effective reconstructive method with high patient satisfaction. Primary surgeries generally have better outcomes than secondary revisions. The outcome of the buccal fat pad procedure has shown to be negatively affected by smoking, previous surgeries and the presence of post-operative complications. Future research should refine techniques for secondary cases and investigate treatments to improve tissue quality.

**2FA26/1012**
**Aesthetics**
**Modified Extended SMAS Technique in Facelift Surgery at Gangwhoo Cosmetic Hospital**
Le Ngoc Tuan Anh ^1^ and Phung Manh Cuong ^1^
*^1^* *Gangwhoo Cosmetic Hospital, Ho Chi Minh City, Viet Nam*
**Objectives:** To evaluate the aesthetic effectiveness and safety of the modified Extended SMAS technique in facelift surgery at Gangwhoo Cosmetic Hospital.
**Materials and Methods:** A prospective descriptive longitudinal study was conducted from January 2024 to June 2025 on 72 patients with a mean age of 53 ± 7.8 years (62 females, 10 males) at Gangwhoo Cosmetic Hospital. All patients underwent facelift surgery using the modified Extended SMAS technique (SMAS dissection with endoscopic assistance, ligament release, multi-point SMAS fixation suturing combined with multi-vector platysmaplasty). Outcomes were evaluated using the FACE-Q scale, cervicomental angle measurement, jowl score preoperatively and at 1, 3, and 6 months postoperatively; complications were also recorded.
**Results:** The mean operative time was 175 ± 30 min, with a hospital stay of 1 day. After 12 months of follow-up, FACE-Q facial appearance scores increased significantly from 53.1 ± 9.2 preoperatively to 85.3 ± 14.6 postoperatively (*p* < 0.001). 93% of patients showed marked improvement in jawline and neck contours, mean jowl score decreased by 70%, cervicomental angle improved by approximately 13°, and patients self-reported appearing 7.2 ± 1.5 years younger. Complications were minimal: hematoma in 2.8% of cases (managed conservatively), temporary facial nerve weakness in 1.4% (full recovery within 6 weeks), skin necrosis in 1.4%, with no cases of infection, or permanent facial nerve paralysis.
**Conclusion:** The modified Extended SMAS technique at Gangwhoo Cosmetic Hospital has demonstrated its effectiveness in rejuvenating the lower face and neck, producing natural results, rapid recovery, and a high safety profile with low complication rates. Technical modifications such as endoscopic assistance and multi-point fixation suturing have shown clear advantages over traditional techniques.

**2FA26/1203—Aesthetics**
**Rethinking Facial Aesthetic Ideals in Southeast Asia: A Comprehensive Anthropometric Evaluation of Young Malay Females**
Nurul Fatihah Hussien ^1^, Nurhaifa Adlina Mohd Yunos ^1^, Michelle Clare Mah ^1^, Roger Zwahlen ^2^ and Su Keng Tan ^1^
*^1^* *Universiti Teknologi MARA, Sg Buloh, Malaysia*
*^2^* *Ospedale Regionale di Lugano, Lugano, Switzerland*
**Objectives**: To evaluate the applicability of classical facial aesthetic standards in young Malay females and to determine whether facial anthropometric parameters differ among various skeletal patterns.
**Materials and Methods**: Sixty-four healthy Malay females with normal body mass index were recruited. Standardised frontal and lateral facial photographs were obtained in natural head position. Nineteen golden ratio parameters, eight neoclassical canon parameters, twenty-four facial ratios and twenty-six angular measurements were analysed using digital photogrammetry. Measured values were compared with established reference standards. Subgroup analysis was performed according to skeletal classification.
**Results**: Among the 73 anthropometric parameters evaluated, only 10 demonstrated no significant difference from their corresponding reference values (*p* > 0.05). When clinically acceptable margins of deviation were applied, applicability increased substantially, with nine parameters demonstrating applicability exceeding 90%. The ratio Sto–Me:Sn–Me exhibited 100% applicability across the cohort. In contrast, eight parameters were not observed in any participant despite allowance for acceptable variation. Significant differences between skeletal patterns were identified in one golden ratio parameter, two neoclassical canon parameters, six facial ratios and ten angular measurements (*p* < 0.05).
**Conclusion**: Most classical facial aesthetic standards were not consistently observed in young Malay females. These findings highlight the limitations of applying universally accepted aesthetic standards to Malay women and underscore the importance of recognising population-specific facial characteristics during aesthetic assessment and treatment planning.
Conflicts of Interest disclosure:
Receipt of grants/research supports: This study is funded by MyRA Research Grant, Universiti Teknologi MARA—600-RMC/MyRA 5/3/LESTARI (077/2020).

**FA26/237—Basic and Translational Research**
**Scaffold Architecture Accelerates Early Osteogenic Differentiation: A Comparative Study of 3D-Printed β-Tricalcium Phosphate Constructs Under Dynamic Bioreactor Culture**
Yannick Sillmann ^1,2^, Ana Baggio ^2^, Pascal Eber ^2^, Benjamin Freedman ^3^, Cynthia Liu ^3^, Youssef Jounaidi ^4^, Alexander Schramm ^5^, Frank Wilde ^5^ and Fernando Guastaldi ^2^
*^1^* *Department of Oral and Plastic Maxillofacial Surgery, University Hospital Ulm, Ulm, Germany*
*^2^* *Division of Oral and Maxillofacial Surgery, Massachusetts General Hospital, Boston, United States, Harvard School of Dental Medicine, Boston, United States*
*^3^* *John A. Paulson School of Engineering and Applied Sciences, Harvard University, Cambridge, United States, Wyss Institute for Biologically Inspired Engineering at Harvard University, Boston, United States*
*^4^* *Department of Anesthesia, Critical Care and Pain Medicine, Massachusetts General Hospital, Harvard Medical School,, Boston, United States*
*^5^* *Department of Oral and Plastic Maxillofacial Surgery, University Hospital Ulm, Ulm Germany, Department of Oral and Plastic Maxillofacial Surgery, Military Hospital Ulm, Ulm, Germany*
**Objectives**: To determine whether increasing macropore size in 3D-printed β-tricalcium phosphate (β-TCP) scaffolds accelerates osteogenic differentiation of porcine bone marrow-derived mesenchymal stem cells (pBMSCs) under dynamic bioreactor culture.
**Materials and Methods**: Additively manufactured β-TCP scaffolds with identical composition and strut size but different macropore sizes (500 µm vs. 1000 µm) were characterized structurally (µ-CT, Energy Dispersive X-Ray Spectroscopy, Electron Microscopy) and mechanically (compression testing). pBMSCs were seeded onto scaffolds and cultured for 7 or 14 days in a rotational oxygen-permeable bioreactor system under osteogenic conditions. Cell viability was assessed by LIVE/DEAD staining, cell distribution by 4′,6-diamidino-2-phenylindole (DAPI) staining, alkaline phosphatase (ALP) activity in culture supernatant, and osteogenic differentiation by qRT-PCR. Statistical significance was set at *p* < 0.05.
**Results**: Both scaffold groups supported high cell viability and increasing cell density, with no relevant group-dependent differences in cell distribution. However, the 1000 µm scaffolds showed a markedly enhanced osteogenic response. ALP activity was significantly higher in the 1000 µm group at day 7 (15.60 ± 1.31 vs. 6.36 ± 1.36 µmol/min/L; *p* < 0.0001) and remained higher at day 14 (9.55 ± 2.90 vs. 4.37 ± 1.64 µmol/min/L; *p* = 0.0122). Gene expression analysis confirmed accelerated osteogenic differentiation, with significantly higher early markers including BMP-2, Runx2, and ALP at day 7 and higher maturation-associated markers including Osx, Col1A1, and Ocl by day 14 in the 1000 µm group. Mechanical testing demonstrated lower compressive strength (843.9 ± 216.6 N vs. 491.2 ± 130.2 N; *p* = 0.0183) in the 1000 µm scaffolds, indicating a trade-off between biological performance and mechanics.
**Conclusion**: Larger macropores accelerated and advanced osteogenic differentiation under dynamic culture. By promoting faster generation of osteogenically primed constructs, optimized scaffold architecture may shorten pre-implantation culture time and advance the clinical translation of bone tissue engineering for craniofacial reconstruction. These findings support the development of zonally graded scaffold-architectures.

**FA26/355—Basic and Translational Research**
**Single-Cell RNA Sequencing-Guided Identification of Cell Surface Targets for PEG-SPION–Mediated Ultrasound Therapy in HPV-Positive Head and Neck Squamous Cell Carcinoma**
Abdulrahman Sakeen Alkandari ^1^ and Siu-Wai Choi ^2^
*^1^* *Oral & Maxillofacial Surgery, Ministry of Health, Kuwait, Kuwait*
*^2^* *Orthopedics & Traumatology, University of Hong Kong, Hong Kong, Hong Kong*
**Objectives**: This study aimed to identify cell surface proteins expressed on tissue from HPV-positive head and neck squamous cell carcinoma (HNSCC) using publicly available single-cell RNA sequencing (scRNA-seq) datasets. Identified targets were used to develop a polyethylene glycol (PEGylated)- superparamagnetic iron oxide nanoparticle (SPION)-based therapy for tongue tumours in a mouse model.
**Materials and Methods**: Public datasets, Gene Expression Omnibus (GEO) and Human Cell Atlas were used to identify proteins from HPV-positive HNSCC tumours/normal tissues. scRNA-seq data was analysed using Scanpy.
Following normalization, dimensionality reduction, clustering, and cell-type annotation, malignant epithelial cells were identified and compared with normal epithelial, stromal, endothelial, and immune populations.
Differential gene expression analyses were used to identify cell surface peptides selectively enriched on tumour cells.
An orthotopic HPV-positive tongue tumour mouse model was established using HPV16 E6/E7-expressing mEERL cells implanted into the tongues of immunocompetent C57BL/6J mice.
Selected tumour surface peptide ligands were conjugated to PEGylated SPIONs (~50–70 nm) to improve stability, and reduce nonspecific uptake. Following administration, nanoparticle accumulation in tumours were assessed using imaging and histological analysis in six SPION and six vehicle control mice. External focused magnetism was applied to the tumour region to activate SPION-mediated thermal and mechanical effects and induce tumour cell death.
**Results**: Candidates identified as being tumour specific were FGFR3-TACC3, an oncogenic fusion (chimeric) protein, and neoantigens E542K and E545K. These were successfully ligated to the SPIONs.
SPION injected at the tumour site was confirmed through magnetic resonance imaging.
Observation for reduction in tumour size between SPION and control group is ongoing.
**Conclusion**: This study combines scRNA-seq-guided target discovery with an established HPV-positive HNSCC mouse model to develop a targeted nano-therapy for precision treatment of HNSCC.
SPION activation is expected to reduce tumour burden, by the increase in apoptosis/necrosis, and improve treatment response compared with controls.

**FA26/475—Basic and Translational Research**
**A Dual-Responsive Theranostic Probe for Targeted Diagnosis and Treatment of Temporomandibular Joint Osteoarthritis**
Linli Jiang ^1^
*^1^* *West China Hospital Of Stomatology, Sichuan University, Chengdu, China*
**Objectives**: Temporomandibular joint osteoarthritis (TMJOA) is a chronic degenerative disease with limited early diagnostic and therapeutic options. This study aims to develop a dual enzyme-responsive theranostic probe (Coa-KGN@PPN) for early detection and targeted therapy of TMJOA by integrating responsive imaging with cartilage-regenerative drug delivery.
**Materials and Methods**: The probe was constructed by incorporating MMP-9-specific cleavage peptide sequences (GPLGVRGC) and the near-infrared fluorophore Cy5.5 into a cationic peptide, which co-assembled with negatively charged chondroitin sulfate to form a coacervate (Coa) encapsulating the chondrogenic small molecule KGN. A surface peptide–phenolic network (PPN), composed of tannic acid and the type II collagen-targeting peptide WYRGRL, was applied to enhance cartilage-specific targeting. The enzymatic responsiveness of the probe was evaluated in vitro and in vivo by monitoring its disassembly upon exposure to MMP-9 and hyaluronidase, resulting in activation of fluorescence and photoacoustic (PA) signals. Therapeutic efficacy was assessed by examining inflammation, oxidative stress, and KGN-induced chondrogenic differentiation in chondrocytes.
**Results**: Coa-KGN@PPN selectively accumulated in inflamed TMJ regions and responded to overexpressed MMP-9 and hyaluronidase, generating enhanced PA and fluorescence signals for dual-modality imaging. The biocompatible probe effectively reduced inflammation and oxidative stress, targeted chondrocytes, and promoted KGN-induced chondrogenic differentiation. PA imaging demonstrated strong potential for early diagnosis and guided therapy of TMJOA, confirming precise targeting, enzyme responsiveness, and synergistic therapeutic effects.
**Conclusion**: The Coa-KGN@PPN theranostic probe enables early-stage TMJOA detection and targeted cartilage regeneration, providing a smart platform for responsive imaging-guided therapy. This strategy offers a promising paradigm for designing enzyme-responsive delivery systems for chronic degenerative and inflammatory joint diseases.
Conflict of interest disclosure
Receipt of grants/research supports: GZC20251211

**FA26/39—Cleft Lip and Palate**
**Novel Technique (3Ps) for Faster Identification and Dissection of Buccal Fat Pad (BFP) Flap Developed During the Comparison of Surgical Outcomes of Using BFP as Adjunct to Palatoplasty Versus Palatoplasty with Absorbable Cellulose Hemostat Along the Lateral Relaxing Incision**
Dana Cheska Santos ^1^, Dax Carlo Pascasio ^2^
*^1^* *Southern Philippines Medical Center, Tebow Cure Children’s Hospital, Davao City, Philippines*
*^2^* *Noordhoff Craniofacial Foundation Philippines Inc., Davao City, Philippines*
**Objectives**: Palatoplasty addresses functional and developmental impairments by recreating a functional velopharyngeal mechanism. This study compared the surgical outcomes of buccal fat pad (BFP) flap to fill the lateral relaxing incision during primary palatoplasty compared to two-flap palatoplasty. Subsequently, a novel technique was developed for faster identification and dissection of BFP.
**Materials and Methods**: Twenty-eight patients were randomly assigned between two-flap palatoplasty and palatoplasty with BFP to compare surgical outcomes via photodocumentation on alternate days from day 1–13 post-operatively, day 21, & day 90. Dissection time of BFP was also documented.
**Results**: Baseline characteristics showed no statistically significant difference. The days of mucosalization of the lateral relaxing incision showed statistically significant improvement in the BFP group Wilcoxon Rank Sum Test (*p* < 0.001), with median for BFP group at day 5 and complete mucosalization at day 9 post-operatively while the control group showed mucosalization by day 28 and beyond. In terms of complications, there is statistically significant more partial wound dehiscence at the junction of the soft and hard palate noted in the control group (6 of 14 patients) and none in the BFP group (*p* < 0.0058).
A novel technique to harvest BFP was presented and summarized in 3Ps which amounted to a statistically significant 60–88% reduction in operative dissection compared to published duration with a mean of 3 min 14 sec. 3Ps summarizes the key steps to remember for faster identification and dissection of BFP corresponding to: (1) pterygomandibular raphe, (2) posterolateral dissection, and (3) pull in a circular motion.
**Conclusion**: A number of studies have shown the ease of use and benefits of BFP during palatoplasty which is confirmed in this study. The novel technique of 3Ps in the dissection of BFP can significantly reduce the additional operative time needed to identify and dissect BFP during palatoplasty.

**FA26/40—Cleft Lip and Palate**
**External Halo Versus Internal Devices in LeFort I Distraction: Effects on Velopharyngeal Insufficiency and Americleft Speech Outcomes**
Ayush Saha ^1^, Huay-Zong Law ^1^ and Mohamed Elfar ^1^
*^1^* *International Craniofacial Institute, Dallas, United States*
**Objectives**: To compare postoperative velopharyngeal insufficiency (VPI) incidence and longitudinal changes in Americleft speech domains between external halo devices (EHD) and internal distraction devices (ID) following LeFort I distraction osteogenesis.
**Materials/Methods**: Patients undergoing LeFort I distraction (2003–2025) were grouped by external halo versus internal devices. Speech outcomes were assessed using Americleft speech domains including hypernasality, nasal air emission, speech intelligibility, speech acceptability, and compensatory errors. Longitudinal speech outcomes were analyzed with cumulative link mixed models, and group differences in postoperative VPI were assessed using Fisher’s exact test.
**Results**: Of 473 patients who underwent orthognathic surgery, 15.2% (72/473) met criteria for LeFort I distraction with at least one preoperative and one postoperative speech evaluation. 94.4% (68/72) of patients had cleft lip/palate, 45.6% (31/68) bilateral and 54.4% (37/68) unilateral. Mean age at surgery was 12.8 years (range, 8–23).
54.2% (39/72) of patients utilized EHD and 45.8% (33/72) utilized ID. Longitudinal analysis demonstrated significantly improved outcomes with internal distraction in nasal air emission at greater than 12 months (*p* = 0.036) and in speech intelligibility and speech acceptability at multiple postoperative intervals (*p* < 0.05), with no significant differences in hypernasality or compensatory errors.
70.8% (51/72) of patients had no preoperative VPI. New-onset postoperative VPI occurred in 34.5% (10/29) of EHD patients versus 18.2% (4/22) in the ID group (odds ratio = 2.33; *p* = 0.2248).
29.2% (21/72) demonstrated preoperative VPI. By final follow-up, 57.1% (12/21) demonstrated improvement in VPI-related speech domains, 23.8% (5/21) worsened, and 19.0% (4/21) remained unchanged, with no significant difference between EHD and ID.
**Conclusion**: Internal distraction was associated with better longitudinal speech outcomes in nasal air emission, speech intelligibility, and acceptability. No differences were observed in hypernasality or compensatory errors. Internal distraction also demonstrated a lower observed incidence of new-onset VPI post-LeFort I distraction, suggesting a clinically relevant trend warranting further research with larger cohorts.

**FA26/281—Cleft Lip and Palate**
**Novel Technique for Treating Oronasal Fistulae Using Autologous Auricular Composite Graft**
Sari Kishida ^1^, Masanobu Yamashita ^1^, Mikio Yagishita ^1^, Toru Miyanaga ^1^, Miyuki Kishibe ^1^ and Kenichi Shimada ^1^
*^1^* *Kanazawa Medical University, Kanazawa, Japan*
**Objectives**: Oronasal fistulae (ONF) are common complications of palatoplasty, and their treatment remains challenging. The risk of recurrence is particularly high in cases with a history of multiple surgeries and severe scarring. We developed a novel closure technique for ONF using autologous auricular skin–cartilage composite grafts and evaluated its clinical outcomes.
**Materials and Methods**: This retrospective study included patients with ONF treated between 2021 and 2024 who had previously undergone unsuccessful fistula closure surgery. The mucosa around the fistula was elevated as a mucoperiosteal layer and inverted as a hinge flap to close the nasal side. A submucosal pocket was created around the fistula, and a composite graft harvested from the auricular concha was inserted with the cartilaginous portion positioned within the pocket. Finally, the surrounding oral mucosa and the skin of the graft were sutured.
**Results**: The procedure was performed in 13 patients. The primary cleft type was Veau II in three cases, Veau III in seven cases, and Veau IV in three cases. The fistula location was classified as Type IV in nine cases and Type V in four cases according to the Pittsburgh classification. The mean number of previous closure procedures was two (range: 1–4). Complete closure was achieved in nine cases (69%). After repeat surgery in three patients, closure was obtained in two additional cases, resulting in an overall closure rate of 85%. Small residual fistulae in two cases were subsequently closed using alveolar bone grafting.
**Conclusion**: Compared with previously reported cartilage grafting alone, this technique uses composite tissue including skin, providing a strong mechanical barrier until stable closure of the nasal layer is achieved. Auricular skin–cartilage composite grafting may represent a useful minimally invasive option for treating refractory fistulae.

**2FA26/1052—Cleft Lip and Palate**
**Minimally Invasive Palatoplasty Surgical Outcomes: Speech Evaluation and 3d Digital Analysis of Maxillary Arch Shape**
Ken Ayu Miranthy ^1^, Nur Aini ^1^, Muhammad Syafrudin Hak ^1^ and Rita Rahmawati ^1^
*^1^* *Woman and Children Harapan Kita Hospital, Jakarta, Indonesia, DKI Jakarta, Indonesia*
**Objectives**: This study compares speech and maxillary arch shape as surgical outcomes in cleft lip palate patient who underwent conventional two flap palatoplasty and minimal invasive palatoplasty.
**Materials and Methods**: Retrospective study was conducted involving patient with unilateral labiognathopalatoschizis complete sinistra who underwent primary palatoplasty. All procedure were performed by same surgeon and standardized surgical protocol in Cleft Center. Patients were divided into two groups according to surgical technique: conventional two flap palatoplasty and minimally invasive palatoplasty. 3D digital model analysed using mesh mixer software. Speech out comes evaluation performed by speech therapist for hypernasality, speech intelligibility and articulation.
**Results**: 125 plaster models scanned, showed statistically significant difference between mean dental arch width estimated in conventional two flap palatoplasty and modified two flap palatoplasty. Conventional two flap palatoplasty demonstrated 43.7% V-shaped and 56.3% U-shaped maxillary dental arch, while minimally invasive palatoplasty presented 7% V-shaped and 93% U-shaped maxillary dental arch. Speech of 15 patients evaluated perceptually by speech therapist. Speech intelligibility was good in both groups, hypernasality was normal in both groups, articulation was normal in minimally invasive palatoplasty.
**Conclusion**: Maxillary arch shape in minimally invasive palatoplasty were less constricted that in conventional two flap palatoplasty. Despite that, the speech outcomes were favourable in both group, this might happened because the z-plasty in nasal layer following with push back in both groups.

**2FA26/1251—Cleft Lip and Palate**
**Nutritional Status Comparison Between Children with Isolated Cleft and Cleft-Associated Multiple Congenital Anomalies Based on z-Score Values**
Salsabila Ananda ^1^, Shaffira Herawati ^1^, Ajeng Puspa ^1^, Nathanael Ferrel Nuralim ^1^, Fitriana Fitriana ^2^, Ken Ayu Miranthy ^1^, Nur Aini ^1^, Prajnya Narendraswari ^1^, Novitria Dwinanda ^1^ and Muhammad Syafrudin Hak ^1^
*^1^* *Woman and Children Harapan Kita Hospital, Jakarta, Indonesia*
*^2^* *Department of Oral and Maxillofacial, Faculty of Dentistry, Brawijaya University, Malang, Indonesia*
**Objectives**: This study aimed to compare nutritional status, represented by z-score values, between children with isolated cleft and children with cleft-associated multiple congenital anomalies.
**Materials and Methods**: Data were analysed using SPSS software. Patients were categorized into two groups: isolated cleft and cleft-associated multiple congenital anomalies. Demographic characteristics, including age and gender, were descriptively analysed. The normality of continuous variables was assessed using the Shapiro-Wilk test. Age was not normally distributed and was therefore presented as median and interquartile range and compared using the Mann-Whitney U test. Z-score was normally distributed and was presented as mean ± standard deviation and compared using an independent *t*-test. A *p*-value of <0.05 was considered statistically significant.
**Results**: A total of 197 patients were included, consisting of 100 children with isolated cleft and 97 children with cleft-associated multiple congenital anomalies. The isolated cleft group consisted of 53 males and 47 females, while the cleft-associated multiple congenital anomalies group consisted of 44 males and 53 females. The median age was 18 days in the isolated cleft group and 48 days in the cleft-associated multiple congenital anomalies group, with a statistically significant difference between groups (Mann-Whitney U = 2662.500, *p* < 0.001). The mean z-score was significantly lower in the cleft-associated multiple congenital anomalies group compared with the isolated cleft group (−1.983 ± 1.671 *vs.* −0.971 ± 0.900; *p* < 0.001). This finding indicates poorer nutritional status among children with cleft-associated multiple congenital anomalies.
**Conclusion**: Children with cleft-associated multiple congenital anomalies showed significantly lower z-score values compared with children with isolated cleft. These findings suggest that the presence of additional congenital anomalies may contribute to greater vulnerability to nutritional status in children. Early assessment and intervention are essential to support optimal growth of the patients.

**FA26/219—Craniofacial Surgery**
**Syngnathia: Clinical Manifestations and Treatment Approaches from a European Case Series**
Saranda Ombashi ^1^, Roman Khonsari ^2^, Víctor Zafra Vallejo ^3^, Michael Krimmel ^4^, Krzysztof Dowgierd ^5^ and Eppo Wolvius ^1^
*^1^* *Erasmus Medical Center, Rotterdam, The Netherlands*
*^2^* *Hôpital Necker—Enfants Malades, Paris, France*
*^3^* *University Hospital 12 de Octubre, Madrid, Spain*
*^4^* *University Medical Center Tübingen, Tübingen, Germany*
*^5^* *Regional Specialist Children’s Hospital, Olsztyn, Poland*
**Objectives**: Syngnathia is a very rare craniofacial anomaly, referring to different forms of fusion of the upper and lower jaw, with or without involvement of the temporomandibular joint (TMJ). Due to its rarity, limited knowledge is available about the clinical manifestation and treatment strategies. The aim of the study is to map out the clinical manifestation and the currently used treatment options by collection information systematically in a case series.
**Materials and Methods**: Within the European Reference Network of Craniofacial Anomalies and ENT disorders, a call was sent out to identify cases of syngnathia. With the involved centers, a clinical checklist was developed. Data was collected via the checklist and radiological images and photographs were collected. A comparison with previously reported cases from the literature was made.
**Results**: A total of eleven cases of syngnathia were identified. Cases included bony and fibrous syngnathia, as well as a combination. Most patients were diagnosed directly after birth. Cases were often associated with additional (craniofacial) anomalies. Syndromes occurred in four of the cases. In six cases, the TMJ was involved. Treatment strategies varied and timing of the first surgery differed between four days and nine years, sometimes leading to relapse. Feeding problems occurred in all patients. The need for a tracheal stoma was reported in eight patients. In literature, mild cases (such as oral bands) are more often reported, requiring a fundamentally different clinical treatment strategy that more severe cases.
**Conclusion**: This study presents the largest case series of syngnathia so far, emphasizing the high occurrence of oral health problems in the population. Suggestions for adjustment of a clinical classification system are made. Concerning clinical practice, treatment teams should be aware that respiratory problems and feeding problems may occur directly or shortly after birth. Furthermore, caution should be taken with early release operations.

**FA26/278—Craniofacial Surgery**
**Choanal Stenosis and/or Atresia with Midface Airway Morphology in Pediatric Craniofacial Disorders: A Retrospective Imaging Characterization Study**
Batmaraj Rawisandran ^1^, Maudina Dwi Heriasti ^2^, Zheni Kong ^2^ and Firdaus Hariri ^2^
*^1^* *Kementerian Kesihatan Malaysia, University Malaya, Putrajaya, Malaysia*
*^2^* *University Malaya Medical Centre, Kuala Lumpur, Malaysia*
**Objectives**: To characterize posterior nasal airway obstruction in pediatric craniofacial disorders by evaluating the spectrum of choanal stenosis and/or atresia, associated midface skeletal morphology, syndromic associations, and implications for craniofacial airway management.
**Materials and Methods**: A retrospective review was performed of **109 pediatric craniofacial patients** managed within a multidisciplinary craniofacial service. Clinical records were screened for documented choanal stenosis, choanal atresia, and posterior nasal airway obstruction. Patients with relevant pathology underwent CT scan review using available pre-operative, serial, or single time-point imaging. Morphologic assessment included choanal patency, posterior nasal cavity narrowing, septal and rostral morphology, nasopharyngeal dimensions, and radiologic indicators of midface hypoplasia. Historical diagnoses were retained and correlated with imaging findings to evaluate diagnostic concordance. Surgical procedures, including craniofacial advancement procedures, were reviewed.
**Results**: Posterior nasal airway obstruction was identified in **16 of 109 patients (14.7%)**. Choanal stenosis demonstrated syndromic clustering, occurring in **5 of 18 (27.8%)** patients with Crouzon syndrome, **5 of 19 (26.3%)** with Apert syndrome, and smaller numbers in Pfeiffer, Nager, Fraser, and Antley–Bixler syndromes. No stenosis was observed in Pierre Robin sequence, Goldenhar, or Treacher Collins cohorts. CT review demonstrated residual choanal patency in all cases, supporting varying degrees of stenosis rather than complete atresia and highlighting potential discordance between clinical and radiologic diagnoses. Serial imaging demonstrated progressive enlargement of choanal and nasopharyngeal dimensions over time in both operated and non-operated patients.
**Conclusion**: Choanal stenosis is observed in syndromic craniofacial patients, particularly craniosynostosis phenotypes, and appears to represent a continuum of posterior airway narrowing rather than true atresia. Airway enlargement is multifactorial, reflecting both normal growth and, in selected cases, craniofacial skeletal intervention. These findings support integrated endoscopic and imaging-based evaluation and have important implications for multidisciplinary craniofacial airway planning and timing of intervention.

**FA26/340—Craniofacial Surgery**
**Skeletal Stability Following Le Fort 1 Maxillary Advancement with Rigid Fixation: A Retrospective Study**
Sunil Kumar Gulia ^1^
*^1^* *SGT University, Gurugram, India*
**Objectives**: To evaluate the magnitude of horizontal skeletal relapse and identify predictors influencing stability following Le Fort 1 maxillary advancement with rigid fixation.
**Materials and Methods**: A retrospective study was conducted on 32 adult patients undergoing Le Fort 1 maxillary advancement with or without concomitant mandibular surgery. Standardized lateral cephalograms were analyzed at preoperative, immediate postoperative, and ≥12-month follow-up intervals. Horizontal relapse at point A was assessed. Statistical analysis included independent *t*-tests and multivariate linear regression, with significance set at *p* < 0.05.
**Results**: Mean maxillary advancement was 5.52 ± 1.18 mm. At a mean follow-up of 14.38 ± 1.29 months, mean horizontal relapse was 1.33 ± 0.31 mm, corresponding to 24.06 ± 2.92% relapse. Patients with advancements >5 mm demonstrated significantly lower percentage relapse (22.07 ± 2.64% vs. 26.06 ± 3.35%, *p* = 0.003) and reduced overjet relapse (*p* = 0.001). Multivariate regression identified magnitude of advancement as an independent negative predictor of relapse (*p* = 0.033). Age, sex, and concomitant mandibular surgery were not significant predictors.
**Conclusion**: Rigid fixation provides predictable skeletal stability following Le Fort 1 advancement, with approximately one-quarter relapse at one year. Larger advancements demonstrate superior stability compared to smaller movements, supporting adequate surgical correction rather than conservative under correction.

**FA26/463—Craniofacial Surgery**
**Virtual Surgical Planning and Patient-Specific PEEK Implants in the Management of Neglected Facial Fractures: A Case Report and Scoping Review**
Yesmine Rahim ^1^ and Trimartani Koento ^1^
*^1^* *Faculty of Medicine, Jakarta, Indonesia*
**Objectives**: To map surgical evidence on reconstruction of neglected maxillofacial fractures, determine whether VSP or patient-specific polyetheretherketone (PEEK) implants have been applied in this population, and present the first documented case of their combined use, demonstrating advantages in defect quantification, implant precision, and surgical predictability, within a multidisciplinary framework.
**Materials and Methods**: A scoping review was conducted following the Population, Concept, and Context framework across databases (PubMed, ScienceDirect, Cochrane) and manual search in April 2026, restricted to the preceding ten years. Eligible studies reported adult patients with neglected maxillofacial fractures undergoing reconstruction by any technique; studies involving acute fractures, pediatric populations, or animal/cadaveric models were excluded. No report documenting VSP, patient-specific implants, or PEEK in this population was identified. Concurrently, a case managed using VSP-guided patient-specific PEEK implantation is presented.
**Results**: Six studies (38 patients) met inclusion criteria, all employing conventional reconstruction exclusively, ORIF, bone grafting, or closed reduction with no use of VSP, patient-specific implants, or PEEK. Irreversible vision loss occurred in two studies: Wahdini et al. additionally reported cantopexy failure and soft tissue sagging requiring revision procedures, while Khairiza et al. reported permanent vision loss following late orbital repair, both highlighting the limitations of conventional reconstruction. The presented case, a 26-year-old male with one-year-old craniofacial malunion, underwent reconstruction a Le Fort I osteotomy for occlusal restoration, followed by VSP-guided zygomaticomaxillary and fronto-orbital reconstruction, with defect closure using a patient-specific PEEK implant, calvarial bone grafts, and augmentation rhinoplasty, with an uneventful recovery.
**Conclusion**: VSP and patient-specific PEEK implants remain exceedingly rare in neglected maxillofacial reconstruction, with no such report identified underscoring the comprehensive contribution of this combined report. VSP-guided PEEK implantation yielded satisfactory occlusal restoration, reduced operative time, decreased intraoperative blood loss, and shorter hospital stay, demonstrating feasibility and advantage as a proof of concept warranting further prospective investigation.

**FA26/501—Craniofacial Surgery**
**A Preliminary Three-Dimensional Assessment of Upper Airway Morphology in Children with Craniofacial Syndromes**
Maudina Dwi Heriasti ^1^, Lau May ^2^, Norliza Ibrahim ^3^ and Firdaus Hariri ^3^
*^1^* *Oral and Maxillofacial Sciences, Faculty of Dentistry, Universiti Malaya, Dr Cipto Mangunkusumo Hospital, Jakarta, Indonesia, Kuala Lumpur, Malaysia*
*^2^* *Department of Paediatrics & Orthodontics, Faculty of Dentistry, Universiti Malaya, Kuala Lumpur, Malaysia*
*^3^* *Oral and Maxillofacial Sciences, Faculty of Dentistry, Universiti Malaya, Kuala Lumpur, Malaysia*
**Objectives**: This preliminary study aimed to describe upper airway morphology and explore its relationship with craniofacial hard and soft tissue structures in children with craniofacial syndromes using three-dimensional imaging.
**Materials and Methods**: A retrospective preliminary study was conducted using craniofacial CT imaging from children treated at the Multidisciplinary Craniofacial Clinic, Universiti Malaya Medical Centre, Malaysia. Twelve subjects were included and divided into three groups: branchial arch syndrome, syndromic craniosynostosis, and controls, with four samples in each group. Children in the craniofacial syndrome groups had available pre- and post-operative CT scans following distraction osteogenesis. Upper airway morphology was analysed using Dolphin software from the nasopharynx to the hypopharynx. Parameters included total airway volume, minimum cross-sectional area, anteroposterior and lateral widths, and airway length. Relevant craniofacial hard- and soft-tissue landmarks were also assessed. Data were analysed descriptively due to the preliminary nature and limited sample size.
**Results**: Both craniofacial syndrome groups demonstrated a trend toward increased total airway volume and minimum cross-sectional area following distraction osteogenesis. However, airway dimensions remained smaller than those observed in controls. Airway length and transverse measurements showed variable findings. Several patients demonstrated an oval airway configuration with relative anteroposterior narrowing. SNB tended to increase following distraction in both craniofacial groups, while SNA changes were more apparent in syndromic craniosynostosis. Other hard and soft-tissue parameters showed heterogeneous patterns across patients.
**Conclusion**: This preliminary study suggests that distraction osteogenesis may improve selected upper airway parameters in children with craniofacial syndromes, particularly total airway volume and minimum cross-sectional area. However, airway dimensions remained smaller than controls, and craniofacial hard and soft-tissue relationships were variable. Larger studies are required to confirm these preliminary findings.

**2FA26/1158—Craniofacial Surgery**
**Orthodontic and Surgical Treatment of An Apert Patient—From childhood to Adulthood**
Louise Barnechow ^1^, Thomas Kofod ^1^ and Sanne Andersen ^1^
*^1^* *Department of Oral and Maxillofacial Surgery, Copenhagen University Hospital, Rigshospitalet, Copenhagen, Denmark, Copenhagen, Denmark*
**Objectives**: To present an 8-year interdisciplinary treatment course of a growing girl with Apert syndrome and demonstrate how combined orthodontic and craniofacial surgical interventions can improve functional occlusion, dental and facial aesthetics, and thereby reduce the risk of stigmatization from childhood to adulthood.
**Materials and Methods**: A 10-year-old girl diagnosed with Apert syndrome presented with midfacial retrusion, impacted 13, 21 and 23, extreme dental crowding, skeletal Class III relationship, and functional impairment. Early surgical removal of 14, 25, 54, 53, 61 and 64 and later interceptive orthodontic treatment included surgical exposure and orthodontic traction of impacted teeth.
Due to progressive midfacial retrusion during growth and impaired respiratory function, Le Fort III advancement surgery was performed at 13 years of age. Comprehensive orthodontic treatment in booth jaws continued throughout growth to align and coordinate the dental arches and prepare for definitive orthognathic surgery after skeletal maturity. At 17 years of age, orthognathic surgery consisting of Le Fort I and bilateral sagittal split osteotomy, was performed. The treatment was completed at the age of 18.
**Results**: Successful eruption and alignment of impacted teeth were achieved without additional tooth loss beyond the extracted, 14 and 25. Midfacial advancement improved facial convexity, orbital support, and respiration, likewise reducing the risk of stigmatization.
Final treatment outcomes demonstrated a stable Class I canine occlusion with normalized overjet and overbite, dental alignment, and significant enhancement of facial harmony and aesthetics. Functional improvement included increased masticatory efficiency and oral function, with high patient satisfaction.
**Conclusion**: Management of Apert syndrome requires long-term interdisciplinary treatment, planning and precise timing of orthodontic and surgical interventions throughout growth. This case demonstrates that staged treatment combining early interceptive orthodontics, Le Fort III advancement, and finally orthognathic surgery with comprehensive orthodontics can achieve substantial functional and aesthetic rehabilitation in patients with complex craniofacial deformities.

**FA26/251—Dental Rehabilitation**
**Tears of Agony to Tears of Ecstasy: The Full Spectrum of Mucormycosis Rehabilitation**
Utsav Bhatt ^1^
*^1^* *Government Dental College and Hospital Ahmedabad, Bhatt’s Dental Clinic Implant and Maxillofacial Rehabilitation Centre, Ahmedabad, India*
**Objectives**: Rhinomaxillofacial mucormycosis management requires aggressive debridement resulting in extensive defects causing functional, aesthetic and psychological deficit.
Rehabilitation is challenging and management options range from simple denture, obturator, conventional implants, corticobasal implants, zygomatic implants, fibula grafting with simultaneous/delayed implant placement and patient specific custom-made implants (PSI).
Present study aims to help clinician make a decision as to which type of treatment option to opt for depending on type of defect that is to be rehabilitated.
**Materials and Methods**: Study comprised 32 patients rehabilitated at least 6 months post-surgery. Number of patients rehabilitated with denture/obturator- 14, zygomatic implants-6, fibula-5 (conventional implants-4, basal implant-1), basal implants-3 and PSI-4. Treatment modality was decided on clinical condition (presence/absence of oroantral/oronasal communication) and radiographic assessment (residual bone in zygoma and nasomaxillary buttress). Evaluation parameters included retention, aesthetics, type of anaesthesia (Local/General), approach (intraoral/extraoral), infection, cost, ease of hygiene maintenance and prosthetic complications.
**Results**: Dentures were simplest but least convenient option. Recurrent inflammation at implant oral mucosal junction (27.7%{5/18}) and infection (16.6%{3/18}) were commonest complications in PSI, implants in fibula and zygoma where fixed prosthesis was given. Overall patients had significant improvement in general health, nutrition and quality of life.
**Conclusion**:
1. Zygomatic implants were less costly and simpler options compared to PSI because of ease of procedure and better prosthetic options.
When dimensions of zygoma didn’t permit placement of 2 zygomatic implants on each side, PSI was preferred since it allowed choice of abutment positioning and gave option of fixed prosthesis.
2. For patients with soft tissue defect and nasal regurgitation removable prosthesis or fibula permitting defect closure followed by fixed prosthesis was preferred. PSI was contraindicated in such patients.
3. Conventional implants were preferred over corticobasal implants, however the latter had an advantage in cases where the soft tissue over fibula was thick and rough surface conventional implants would cause soft tissue inflammation beneath prosthesis.

**FA26/200—Oncology**
**Linking Surgical Resection with Postoperative Adjuvant Radiotherapy with 3D Virtual Surgical Planning—A Retrospective Clinical Study**
Jane Jingya Pu ^1^ and Yuxiong Richard Su ^1^
*^1^* *The University of Hong Kong, Hong Kong, Hong Kong*
**Objectives**: Computer-assisted surgery (CAS) and virtual surgical planning (VSP) have revolutionized oral cancer resection and reconstruction. However, even with careful preoperative margin assessment, the proportion of close/positive (inadequate) margins after tumour resection was only reduced from 55.2% to 40.5%. Designing postoperative radiotherapy to address the inadequate margins in 3D relies on histopathology reports of the margin status in resected tumour specimen. Currently, histopathological reports issued by pathologists are in descriptive text, making it challenging to accurately locate areas of concern in 3D for PORT planning. To this end, we aim to establish and validate a protocol incorporating 3D VSP with PORT design using our existing clinical database.
**Materials and Methods**: Patients with successful VSP-guided resection and PORT in our center were included. Dose volume histograms (DVHs) were used to evaluate dose distribution in RT. DVH and conformity indices (CI) at the VSP tumour site and planning target volume (PTV) were calculated and compared. The matching accuracy of remaining jaw models from VSP and postoperative CT scans and RT coverage at the VSP tumour site was assessed.
**Results**: Alignment and measurements were performed in 20 patients. The mean absolute deviation of the remaining jaw models from VSP and postoperative CT scans was 0.31 mm (SD = 0.79 mm), showing a high level of accuracy in alignment. The PORT dosage at VSP tumour (62.16 Gy) and PTV (61.45 Gy) showed no significant difference (*p* = 0.52). The average CI for VSP tumour model and PTV were 94.23% and 93.82% respectively, showing no significant difference (*p* = 0.80). This demonstrates the correlation of the VSP tumour model with the current PORT design. However, 5 of the 20 patients had CI less than 95%, indicating the gap in addressing the high-risk area with the current design technique.
**Conclusion**: 3D VSP has been proven effective in linking surgical resection with postoperative adjuvant radiotherapy.
Conflict of interest disclosure
Receipt of grants/research supports: Oral Health Research and Innovation Fund (OHRIF) 2024
Funding source: The University of Hong Kong. No conflict of interest declared.

**FA26/241—Oncology**
**Beyond Binary: A Three-Axis Morphological Classification of Perineural Invasion Predicts Recurrence in Node-Negative Oral Squamous Cell Carcinoma**
Sreeram MP ^1^, Shanthi Velusamy ^1^, Prajwal Dange ^1^, Karthik N Rao ^1^, Rekha Vijaykumar ^1^ and B S Srinath ^1^
*^1^* *Sri Shankara Cancer Hospital and Research Centre, Bengaluru, India*
**Objectives**: Perineural invasion (PNI) is currently reported as a binary finding, obscuring its biological heterogeneity. This study aimed to risk-stratify/classify PNI in oral squamous cell carcinoma (OSCC) across anatomical, geographical, and biological dimensions and identify parameters that independently predict recurrence.
**Materials and Methods**: 101 consecutive, pathologically node-negative (pN0) OSCC patients undergoing curative intent resection were studied. Whole-slide images were independently reviewed by two blinded pathologists. Six morphological parameters were recorded: largest nerve diameter (P1; anatomical), nerve count (P2; anatomical), intratumoral versus extratumoral location (P3; geographical), circumferential involvement (P4; geographical), nerves with tumour mitotic activity (P5; biological), and distance to nearest uninvolved nerve (P6; biological). Cutpoints were identified by maximally selected rank statistics. Prognostic analysis used univariate Cox regression, LASSO-penalised Cox regression with 500-resample bootstrap stability analysis, and bivariable Cox models.
**Results**: PNI was present in 33 patients (32.7%). Large nerve diameter (P1 > 429 µm) was the strongest univariate predictor of disease-free survival (HR 3.92, 95% CI 1.18–13.02; *p* = 0.026). Bootstrap LASSO stability analysis identified P1 (64.8%), lymphovascular invasion (55.0%), and WPOI-5 (53.2%) as stable independent predictors. Bivariable models confirmed P1 remained significant after adjusting for all confounders (adjusted HR 4.43–7.59). A phi-coefficient co-occurrence heatmap revealed a biologically aggressive triad, large nerve involvement, extratumoral spread, and intraneuronal mitotic activity, clustering distinctly from clinicopathological variables and defining a high-risk PNI phenotype. A composite PNI score (0–6 points) demonstrated a dose-response relationship (HR per point 1.34, 95% CI 1.01–1.77; *p* = 0.044); high-risk patients (score 3–6) had 24-month DFS of 22.5% versus 75.3% (*p* = 0.003).
**Conclusion**: Morphological sub-classification of PNI into anatomical, geographical, and biological dimensions reveals clinically meaningful risk strata. Large nerve diameter independently predicts recurrence beyond established risk factors, and a composite PNI score stratifies pN0 OSCC patients into significantly divergent survival groups, warranting prospective validation.

**FA26/334—Oncology**
**Axial Pattern Platysma Myocutaneous Flap in Reconstruction of Intraoral Oncologic Defects**
Ei Thinzar Kyaw ^1^
*^1^* *Department of Medical Service, Ministry of Health, Yangon, Myanmar*
**Objectives**: This study was aimed to evaluate the survival rate and outcomes of the modified axial pattern platysma myocutaneous flap in reconstruction of intraoral soft tissue defects.
**Materials and Methods**: Thirty-four subjects of biopsy proven oral cancerous lesions (cT1/T2/T3N0Mx) at tongue, buccal mucosa, mandibular alveolar ridge and retromolar region were included in this study. The defect after tumor ablation was reconstructed with modified axial pattern platysma myocutaneous flap. The flap was harvested during neck dissection with preservation of facial artery and external jugular vein included within the flap. The viability of flap and postoperative outcomes of donor and recipient sites were evaluated within postoperative 90th day.
**Results**: The mean age was 52.85 ± 10 years and 85.2% of population was betel quid chewers. The operation time was 210 ± 30 min. The flap size ranged from 10 cm^2^ to 41.3 cm^2^ (mean 21.1 ± 6.3 cm^2^). Full flap survival was found in 67.6% with loss of skin in seven cases (20.6%) and partial necrosis in 4 cases (11.8%) but total loss in none of the cases. No oro-cutaneous fistula and marginal mandibular nerve palsy was seen. Minor wound dehiscence in neck wound was noted in 5 cases (14.7%) with history of smoking and young age with lack of skin laxity. Haematoma was found in 4 cases with marginal mandibulectomy (11.8%). These complications were minor and none required surgical revision. Regarding the neck scar, none of the patients complained of impairment of neck movements or aesthetic dissatisfaction.
**Conclusion**: The findings of this study showed that modified axial pattern platysma myocutaneous flap could provide an alternative method for intraoral reconstruction of small-to medium-sized defects of tongue, buccal mucosa and mandibular alveolar ridge.
Conflict of interest disclosure
Receipt of grants/research supports: Ministry of Health, Myanmar

**FA26/353—Oncology**
**Decision Making in T4b Lesions of Oral Cancer: When Not to Operate!**
Atish Kundu ^1^
*^1^* *Fortune Hospital, Rama Hospital, Apollo Hospital, Kanpur Nagar, India*
**Introduction:** T4b diseases as staged by AJCC 7th and 8th classification comprises of very advanced local disease where tumor invades masticator space, pterygoid plates, skull base, and / or encases the internal carotid artery. Whether to operate or not a T4b disease has been a topic of debate among surgeons worldwide.
**Aim and Objective:** This is a retrospective study of 80 patients between August 2016 to December 2019 categorising T4b lesions into medial and lateral compartments and gives an overview on decision making for operability
**Method:** Patients were classified into masticatory space without going infra-notch (20), going infra-notch of ITF (36), lateral part of supra-notch of ITF (16) and medial part of supra-notch of ITF (20) as Group A, B, Ca and Cb respectivel.
**Result:** Disease free survival and overall survival was observed to be least in group Cb. Most patients of all groups received adjuvant radiotherapy. Group A and Group B patient had better overall and disease-free survival than the rest. Another finding from this study is better prognosis in lateral compartment than medial compartment.
**Discussion:** Medial compartment include structures like foramen ovale, pterygomaxillary fissure, parapharyngeal space while lateral compartment have structures like lateral pterygoid muscle, masseter muscle. Due to factors like more perineural invasion, more vital structure and CSF leakage in the medial compartment, lesions lateral to ITF has better prognosis than those medial to it.
**Conclusion:** Contrary to the previous school of thought where resection in this area was considered inoperable, this study suggests these locally advanced lesions (T4b) to be operable with sound knowledge of anatomical structures, good visibility and adequate expertise of the surgeon.
KEYWORDS: Infratemporal fossa, advanced disease, T4b lesions, medial compartment, lateral compartment

**FA26/361—Oncology**
**Is Rim Resection Sufficient for the Management of Gingival Carcinoma with Bone Invasion?**
Antti Pakkanen ^1^, Tommy Wilkman ^2^ and Johanna Snäll ^2^
*^1^* *Department of Oral and Maxillofacial Diseases, University of Helsinki, Helsinki, Finland*
*^2^* *Department of Oral and Maxillofacial Diseases, University of Helsinki and Helsinki University Hospital, Helsinki, Finland*
**Objectives**: Traditionally, the treatment of carcinoma invading the mandibular bone has been segmental resection of the mandible. Less mutilating rim resections have been used only in cases where there is no true invasion of the bone. Recently, treatment protocols have begun to favor rim resection also in selected cases of gingival cancer, where the tumor invasion has a sufficient bony margin to the inferior alveolar nerve.
**Materials and Methods**: A total of 96 patients diagnosed with ICD-10 code C03.1 (malignant neoplasm of the lower gum) were included in this retrospective study. All treatment plans were determined by a multidisciplinary head and neck tumor board. Of these, 57 patients met the final inclusion criteria. The primary reason for exclusion was the lack of follow-up data, as many patients were referred from tertiary to secondary care centers for follow-up. A total of 35 patients underwent rim resection, while 22 patients received segmental resection. The follow-up period was three years, during which recurrence and metastasis were monitored. Additionally, mortality due to cancer and other causes was recorded.
**Results**: Among patients with pT4a tumors, metastasis or local recurrence occurred in 35.7% of those who underwent rim resection and in 35.0% of those who underwent segmental resection. In the pT4a group, cancer-related mortality was 28.6% in the rim resection group and 25.0% in the segmental resection group. The mean depth of invasion with T4a tumors was 11.7 mm (standard deviation 9.1 mm) in the rim resection group and 19.2 mm (standard deviation 12.2 mm) in the segmental resection group.
**Conclusion**: In selected cases, rim resection, which is associated with lower morbidity, is comparable to segmental resection in the treatment of mandibular gingival carcinoma, including cases with bone invasion. Due to the small sample size, the study should be considered descriptive.

**FA26/431—Oncology**
**Data to Diagnosis: AI’s Expanding Footprint in Head and Neck Oncology**
Hitesh Bansal ^1^, Hannah Arnantha ^2^
*^1^* *London North West University Healthcare NHS Trust, London, UK*
*^2^* *St George’s, Epsom and St Helier Hospital Group, London, UK*
**Objectives**: Artificial intelligence (AI) has emerged as a transformative tool in oncology, particularly in head and neck cancer (HNC), where diagnostic complexity and variability impact outcomes. This literature review aims to evaluate current applications of AI in the detection, diagnosis, prognostication, and management of head and neck malignancies.
**Materials and Methods**: A narrative literature review was conducted focusing on studies involving machine learning (ML), deep learning (DL), and computer vision techniques applied to head and neck oncology. Emphasis was placed on radiomics, histopathological image analysis, and predictive modelling in oral epithelial dysplasia (OED) and oral squamous cell carcinoma (OSCC). Key developments in digital pathology and multimodal data integration were also reviewed.
**Results**: AI demonstrates significant potential in improving early detection and diagnostic accuracy in HNC. Deep learning algorithms applied to histopathological whole-slide imaging enable automated tumour classification, grading, and prediction of malignant transformation. Models such as OMTscore and lymphocyte-based scoring systems have shown improved prognostic capability compared to traditional histological grading. Radiomics and ML approaches enhance tumour staging and prediction of treatment response. Additionally, AI reduces interobserver variability in diagnosis and provides reproducible, quantitative outputs. However, limitations persist, including small dataset sizes, lack of standardisation, and the “black box” nature of many algorithms, which hinders clinical trust and adoption
**Conclusion**: AI has the potential to revolutionise head and neck oncology by enabling earlier diagnosis, improving prognostication, and supporting personalised treatment strategies. Despite promising results, widespread clinical implementation requires larger multicentre datasets, improved model interpretability, and robust validation. Future integration of multimodal data and explainable AI frameworks will be essential for safe and effective translation into clinical practice.

**FA26/473—Oncology**
**Factors Associated with Severe Delay in Postoperative Radiation Therapy After Microvascular Free Flap Reconstruction for Head and Neck Squamous Cell Carcinoma**
Saif Shah ^1^ and John Bishara ^1^
*^1^* *University of California San Francisco—Fresno, Fresno, CA, USA*
**Objectives**: To identify key factors associated with delays in initiation of postoperative radiation therapy in patients undergoing microvascular free flap reconstruction after head and neck squamous cell carcinoma resection.
**Materials and Methods**: We performed a retrospective, single-center cohort study of 58 patients with head and neck squamous cell carcinoma treated with tumor resection, microvascular free flap reconstruction, and postoperative radiation therapy. Time to radiation initiation was defined as the interval from surgery to radiation start, confirmed by treatment records. Delays were categorized as timely (≤42 days), moderate (43–59 days), and severe (≥60 days), with severe delay as the primary endpoint for multivariable analysis. Multivariable logistic regression was used to evaluate predictors including reconstruction type, pathologic T stage, margin status, tracheostomy, gastrostomy tube placement, and major postoperative complications.
**Results**: Median time to radiation was 55 days, with a mean of 62.6 ± 23.0 days. Timely radiation (≤42 days) was achieved in 7 patients (12.1%), while 25 (43.1%) experienced moderate delay and 26 (44.8%) experienced severe delay. Overall, 51 patients (87.9%) exceeded 42 days. Postoperative complications were associated with prolonged time to radiation compared with no complications (75.1 vs. 54.4 days; *p* < 0.001). On multivariable analysis, postoperative complications independently predicted severe delay (odds ratio, 4.37; 95% CI, 1.36–14.09; *p* = 0.014), whereas flap type was not associated (odds ratio, 0.77; 95% CI, 0.19–3.05; *p* = 0.708). Tracheostomy and gastrostomy tube placement demonstrated positive but non-significant associations. Advanced T stage and positive or close margins showed non-significant trends. On sensitivity analysis, complications remained significantly associated with an additional 21.5 days to radiation (*p* < 0.001).
**Conclusion**: Severe delays in postoperative radiation therapy were common. Postoperative complications were the primary driver, outweighing disease-related factors and reconstruction type. Reducing perioperative complications may improve timely radiation initiation more than flap selection in head and neck cancer patients.

**FA26/517—Oncology**
**Genomic Predictors of Occult Lymph Node Metastasis in Early-Stage Head and Neck Squamous Cell Carcinoma: A Pan-Cancer Analysis**
Deddy Septian ^1^, Andri Hardianto ^1^ and Abel Yuza ^1^
*^1^* *Universitas Padjadjaran, Kota Bandung, Indonesia*
**Objectives**: Managing the clinically negative (cN0) neck in early-stage (T1–T2) head and neck squamous cell carcinoma (HNSCC) remains a challenge in craniomaxillofacial surgery. This study aims to identify specific genomic alterations and clinical markers associated with pathologically positive nodes (pN+) to refine patient selection for elective neck dissection.
**Materials and Methods**: Clinical and genomic data from a large public HNSCC cohort (187 patients) were analyzed. The study was restricted to primary T1-T2 tumors. Subjects were stratified by pathologic nodal status: pN0 (n = 81) and pN+ (n = 78). Mutation frequencies, fraction genome altered (FGA), and demographics were compared. Cohorts were matched for age (*p* = 0.094), race (*p* = 0.239), and histologic grade (*p* = 0.339).
**Results**: While ubiquitous driver mutations such as *TP53* were equally prevalent (*p* = 1.00), ZNF804B mutations were significantly enriched in the pN0 cohort (13.8% vs. 1.3%, *p* = 0.0047). Furthermore, pN+ cases exhibited significantly higher global genomic instability (Fraction Genome Altered, *p* = 0.0279) and a stronger correlation with advanced distant metastasis staging (*p* = 0.0244). Overall survival showed a trend toward a decrease in pN+ patients (Log-rank *p* = 0.123).
**Conclusion**: Specific genomic signatures, particularly the presence of *ZNF804B* mutations, correlate with a node-negative phenotype in early-stage HNSCC. This is clinically significant, as pN+ status was associated with significantly higher rates of adjuvant radiation therapy (*p* < 0.001). Integrating these biomarkers with traditional staging could help identify low-risk patients who may be safely spared the morbidity of elective neck dissections.

**FA26/520—Oncology**
**Active Matrix Metalloproteinase-8 (aMMP-8) Levels and Their Association with Health Status and Disease Characteristics in Patients with Oral Cancer**
Krista Voutilainen ^1,2^, Johanna Snäll ^1,2^, Tero Puolakkainen ^1,2^, Johanna Uittamo ^1,2^, Aurora Jokelainen ^1^, Elias Kultanen ^1^ and Timo Sorsa ^1,2^
*^1^* *University of Helsinki, Helsinki, Finland*
*^2^* *Helsinki University Hospital (HUS), Helsinki, Finland*
**Objectives**: Oral cancer is a heterogeneous disease often associated with chronic inflammation. Active matrix metalloproteinase-8 (aMMP-8), a collagen-degrading enzyme produced mainly by neutrophils, reflects inflammatory activity and has been linked to several systemic and oral diseases. However, its role in oral cancer remains insufficiently studied. This study aims to evaluate aMMP-8 levels in oral rinse samples of oral cancer patients and to assess their association with patients’ health status, disease characteristics and prognosis.
**Materials and Methods**: This study includes patients diagnosed with oral cancer requiring surgical treatment at a university hospital. Preoperative oral rinse samples are collected and analyzed for aMMP-8 levels using a non-invasive point-of-care test. Clinical data, including general health status, medical history and known risk factors, are obtained through patient interviews and medical records. Oral and periodontal status is assessed clinically using the Community Periodontal Index (CPI). Associations between aMMP-8 levels and clinical variables are analyzed.
**Results**: Data collection is ongoing, with completion expected in July 2026 and a target sample size of at least 100 patients. Preliminary observations suggest variability in aMMP-8 levels among oral cancer patients. Ongoing analyses explore associations between aMMP-8 levels and patient-related factors, disease characteristics, and postoperative outcomes, including recurrence and metastasis risk.
**Conclusion**: aMMP-8 may serve as a non-invasive biomarker reflecting inflammatory activity in oral cancer patients. If associations between aMMP-8 levels and disease progression or prognosis are confirmed, this biomarker could support early risk assessment, diagnostic processes and individualized treatment planning.
Conflict of interest disclosure
Receipt of grants/research supports: Grant for travel expenses to Face Ahead: University of Helsinki

**2FA26/1074—Oncology**
**Salivary Liquid Biopsy Biomarker Signatures for Prediction of Cervical Nodal Metastasis in Oral Squamous Cell Carcinoma**
Ayesha Rais ^1^ and Hafiz Waqas Ahmed ^2^
*^1^* *Department of Oral & Maxillofacial Surgery, Bolan Medical Complex / Bolan University of Medical and Health Sciences, Quetta, Pakistan*
*^2^* *Department of Oral & Maxillofacial Surgery, Mayo Hospital / King Edward Medical University, Lahore, Pakistan*
**Background**: Cervical nodal metastasis is a key prognostic factor in oral squamous cell carcinoma (OSCC). Non-invasive biomarkers may improve preoperative detection of occult nodal disease and support individualized treatment planning.
**Objective:** To evaluate the predictive utility of salivary liquid biopsy biomarkers for preoperative identification of cervical nodal metastasis in OSCC.
**Methods:** This multicenter translational study included 200 treatment-naive OSCC patients stratified into node-negative (N0) and node-positive (N+) groups according to the AJCC 8th edition TNM classification. Pre-treatment saliva samples were analyzed for transcriptomic biomarkers (miR-21, miR-423-5p), protein biomarkers (EGFR, CYFRA 21-1, Ki-67), and exosomal biomarkers (Exo-miR-1246, Exo-EGFR) using qRT-PCR, ELISA, and standardized exosome isolation protocols. Biomarker expression was assessed using ANOVA and multivariate logistic regression. ROC analysis evaluated predictive performance.
**Results:** Node-positive OSCC patients demonstrated significantly increased salivary expression of Exo-miR-1246, miR-21, EGFR, CYFRA 21-1, and Ki-67 compared with node-negative patients (*p* < 0.001). Exo-miR-1246 demonstrated the strongest independent association with cervical nodal metastasis (OR 4.6; 95% CI: 2.7–7.1; *p* < 0.001). AUC values were 0.89 for Exo-miR-1246, 0.83 for miR-21, and 0.81 for EGFR. Combined biomarker signatures achieved superior predictive performance (AUC 0.92; sensitivity 88%; specificity 85%).
**Conclusion:** Salivary liquid biopsy biomarker signatures show promise for predicting cervical nodal metastasis in OSCC. Integration of transcriptomic, protein and exosomal biomarkers may support precision-guided neck management and improve risk stratification.
**Keywords:** Oral squamous cell carcinoma; Cervical nodal metastasis; Salivary liquid biopsy; Exosomal biomarkers; Risk stratification

**2FA26/1124—Oncology**
**Single-Cell Analysis Reveals Putative Heterogeneous Cancer Stem Cell Subpopulations in Oral Squamous Cell Carcinoma Cell Lines**
Surendra Kumar Acharya ^1^ and Yee Fan Choon ^2^
*^1^* *Oral and Maxillofacial Clinical Sciences, Faculty of Dentistry, University Malaya, OM Samaj Dental Hospital, Kathmandu, Nepal, Kuala Lumpur, Malaysia*
*^2^* *Oral and Maxillofacial Clinical Sciences, Faculty of Dentistry, MAHSA University, Selangor, Malaysia*
**Objectives**: To explore the presence of cancer stem cells subpopulations by investigating (1) the expression of four putative cancer stem cell markers (CD44, CD133, alcohol dehydrogenase, and BMI1), and (2) the holoclone and sphere formation ability of cancer cells in these cell lines.
**Materials and Methods**: Expression of *CD44*, *CD133*, *ALDH1A1*, and *BMI1* was examined at a single-cell level using the C1 Single-Cell Autoprep System and BioMark HD system (Fluidigm). Expression was validated by flow cytometry. Cancer cells were cultured at clonal density to observe for holoclone and sphere formation.
**Results**: Gene expression and flow cytometry analyses showed that CD44 and BMI1 were ubiquitously expressed (>95% of total cells) in all ORL cell lines with three different subpopulations expressing: (1) alcohol dehydrogenase, (2) CD133, (3) alcohol dehydrogenase and CD133. Holoclone and sphere formations of the cancer cells were also observed.
**Conclusion**: The results indicate that ORL cell lines comprise putative heterogeneous cancer stem cell subpopulations with holoclones and sphere formation ability. *CD44* and *BMI1.* Further studies are needed to characterize each subpopulation.
Conflict of interest disclosure:
Receipt of grants/research supports: BKP

**2FA26/1148—Oncology**
**Image-Based Diagnosis of Oral Lesions: Performance of a Vision–Language Model Versus Human Clinicians**
Sebastiano Stellino ^1^, Luigi Vaira ^1^ and Giacomo De Riu ^1^
*^1^* *University Hospital of Sassari, Sassari, Italy*
**Objectives**: To evaluate the real-world diagnostic performance of a multimodal large language model for image-based assessment of oral mucosal lesions compared with clinicians of varying expertise.
**Materials and Methods**: We conducted a prospective international multicenter diagnostic accuracy study across 20 university and tertiary head and neck centers in five countries. We enrolled 350 consecutive patients (320 with oral lesions, 30 with normal mucosa). Clinical photographs and basic epidemiologic data were analyzed using a publicly available vision–language model with a standardized prompt. Model outputs: lesion detection, malignancy versus benign versus normal classification, precise histologic diagnosis, and urgency class (U1–U4). These were compared with histopathology and with four clinicians (general practitioner, general dentist, oral pathologist, head and neck surgeon). Sensitivity, specificity, accuracy, and agreement were computed.
**Results**: The model achieved 97.1% accuracy for lesion detection and malignancy classification, with sensitivity 98.5% and specificity 96.2% for malignancy, and 88.0% accuracy for precise histologic diagnosis, approaching the head and neck surgeon (97.7%) and exceeding the other readers. Three-class diagnostic accuracy was 94.2% for the model versus 67.0–86.5% for non-surgeon clinicians. Urgency assignment was correct in 70% of cases (κ = 0.716), with a conservative tendency to overestimate risk. Agreement with histologic diagnosis was almost perfect (κ = 0.929).
**Conclusion**: In this multicenter exploratory study, a multimodal large language model showed diagnostic performance approaching that of an expert clinician and exceeding that of the non-specialist readers included in this sample. These findings support further prospective validation before considering potential clinical deployment in telemedicine or primary care settings.

**2FA26/1164—Oncology**
**Inverse Relationship Between Neoantigen Clonality and T-Cell Activity Reveals Distinct Immune Phenotypes in HNSCC**
Babak Saravi ^1^, Daman Deep Singh ^1^, Lara Schorn ^1^, Julian Lommen ^1^, Felix Schrader ^1^, Max Wilkat ^1^, Andreas Vollmer ^2^, Michael Vollmer ^3^, Stefan Hartmann ^2^, Marius Hörner ^2^, Kaustubh Adhikari ^4^, Elisabeth Roider ^5^, Jakob Wollborn ^6^, Norbert Kübler ^1^ and Christoph Sproll ^1^
*^1^* *Department of Oral, Maxillofacial and Facial Plastic Surgery, Medical Faculty and University Hospital Düsseldorf, Heinrich-Heine-University Düsseldorf, Düsseldorf, Germany*
*^2^* *Department of Oral and Maxillofacial Plastic Surgery, University Hospital of Würzburg, Würzburg, Germany*
*^3^* *Department of Oral and Maxillofacial Surgery, Tübingen University Hospital, Tübingen, Germany*
*^4^* *School of Mathematics and Statistics, Faculty of Science, Technology, Engineering and Mathematics, The Open University, Department of Genetics, Evolution and Environment, and UCL Genetics Institute, University College London, Milton Keynes, UK*
*^5^* *Department of Dermatology, University Hospital of Basel, Cutaneous Biology Research Center, Department of Dermatology, Massachusetts General Hospital, Harvard Medical School, Basel, Switzerland*
*^6^* *Department of Anesthesiology, Brigham and Women’s Hospital, Mass General Brigham, Harvard Medical School, Boston, MA, USA*
**Objectives**: Immune checkpoint inhibitors benefit only 15–20% of patients with recurrent or metastatic head and neck squamous cell carcinoma (HNSCC). Clonal neoantigens are considered favourable T-cell targets, yet their relationship with the HNSCC immune microenvironment is unclear. We examined how neoantigen clonality relates to T-cell activity, immune phenotypes and survival.
**Materials and Methods**: We analysed 527 TCGA HNSCC tumours. Class I neoantigens were predicted with pVACseq/MHCflurry against consensus HLA genotypes. A binder-weighted VAF clonality score was validated against ABSOLUTE Subclonal Genome Fraction. Immune activity was quantified using a 17-gene exhaustion signature, TIDE dysfunction, CIBERSORT, the Pan-Immune Score and eleven additional readouts. Tumours were stratified into four clonality–immune phenotypes; survival was modelled with multivariable Cox regression including HPV status.
**Results**: Neoantigen clonality showed strong inverse correlations with T-cell signatures: 17-gene exhaustion/activation panel (ρ = −0.41, *p* = 9.8 × 10^−22^), TIDE dysfunction (ρ = −0.53, *p* = 6.7 × 10^−38^) and Pan-Immune Score (ρ = −0.50, *p* = 8.5 × 10^−33^). Four phenotypes emerged: Hot/Low Clonality (33%) combined dense infiltration and high cytolytic activity, whereas Cold/High Clonality (33%) showed minimal immune engagement. High clonality also correlated with reduced antigen presentation machinery expression (ρ = −0.31, *p* = 1.0 × 10^−12^). Survival analysis identified a significant clonality × immune interaction (*p* = 0.030, HPV-adjusted): high clonality predicted improved survival in hot tumours (HR = 0.71, *p* = 0.029) but not in cold tumours (HR = 0.99, *p* = 0.96).
**Conclusion**: Neoantigen clonality inversely predicts T-cell activity in HNSCC, identifying many antigen-rich tumours as immunologically cold. The prognostic benefit of clonality is realised only when T-cell effectors are present, supporting integration of clonality with immune-context measures and immune-priming strategies prior to checkpoint inhibitor therapy in cold tumours.

**2FA26/1174—Oncology**
**Utility of Biopsy Worst Pattern of Invasion for Predicting Adverse Pathologic Features in Oral Tongue Squamous Cell Carcinoma**
Joseph Masselli ^1^, Laurent Ganry ^1^, David Hirsch ^1^ and Andrew Salama ^1^
*^1^* *Northwell Health Division of Oral and Maxillofacial Surgery, New Hyde Park, NY, USA*
**Objectives**: Worst pattern of invasion (WPOI) is an established prognostic factor in oral tongue squamous cell carcinoma (OTSCC). However, its prognostic utility when evaluated on incisional biopsy specimens remains unclear. This study aimed to evaluate whether WPOI assessed on biopsy specimens is associated with adverse pathologic features (APFs) and depth of invasion (DOI) in OTSCC.
**Materials and Methods**: A retrospective cohort study was conducted of patients with OTSCC who underwent incisional biopsy followed by definitive surgical resection. Biopsy WPOI was assigned on H&E-stained biopsy specimens according to criteria described by Brandwein-Gensler, based on the most aggressive invasive pattern identified within the biopsy specimen (20× magnification). Biopsy WPOI was categorized as low-risk (WPOI 1–3) or high-risk (WPOI 4–5). The primary outcome was the presence of APFs, defined as ≥1 of the following on final pathology: extranodal extension (ENE), perineural invasion (PNI), lymphovascular invasion (LVI), or close margin (<2 mm). Secondary outcomes included DOI ≥ 5 mm. Associations were assessed using Fisher’s exact test.
**Results**: Eighteen patients met inclusion criteria. High-risk biopsy WPOI demonstrated a higher prevalence of APFs compared to low-risk WPOI (54.5% vs. 42.9%), though this did not reach statistical significance (Fisher’s exact *p* = 1.00; OR = 1.6). Similarly, high-risk WPOI was associated with a higher proportion of tumors with DOI ≥ 5 mm (54.5% vs. 42.9%), without statistical significance (Fisher’s exact *p* = 1.00; OR = 1.6).
**Conclusion**: In this preliminary cohort, high-risk biopsy WPOI trended toward increased APFs and greater DOI. Statistical significance was not achieved. These findings suggest that biopsy WPOI may reflect underlying tumor aggressiveness; however, limitations of incisional biopsy in capturing the invasive tumor front may affect its predictive utility. Further investigation in a larger cohort is underway at our institution to clarify the role of biopsy WPOI in preoperative risk stratification.

**2FA26/1238—Oncology**
**Clinicopathological Profile and Cervical Lymph Node Burden in Tongue and Oral Cavity Malignancies: A Single-Center Retrospective Study**
Alexander Asmara ^1^ and Kiki Rizki ^1^
*^1^* *Dr. Hasan Sadikin General Hospital, Padjadjaran University, Bandung, Indonesia*
**Objectives**: Cervical lymph node metastasis is an important prognostic factor in tongue squamous cell carcinoma. This study aimed to describe the clinicopathological profile of tongue squamous cell carcinoma cases and evaluate factors associated with cervical lymph node positivity.
**Materials and Methods**: A retrospective analytical study was conducted using an anonymized medical-record-derived dataset from Dr. Hasan Sadikin General Hospital, Bandung, Indonesia, covering 1 May 2025 to 30 April 2026. After screening 38 records, 36 tongue squamous cell carcinoma cases were included. The primary outcome was cervical lymph node status, categorized as positive or negative based on the recorded Lymph Nodes field. Candidate factors included age, sex, largest tumor dimension, anatomical involvement pattern, posterolateral location, and depth of invasion when available. Categorical variables were compared using Fisher exact test, and continuous variables were compared using Mann-Whitney U test.
**Results**: Thirty-six cases were analyzed. The mean age was 47.4 +/− 13.2 years, with a median of 48 years (range, 24–77). There were 20 male patients (55.6%) and 16 female patients (44.4%). Cervical lymph node positivity was recorded in 30/36 cases (83.3%). The median largest tumor dimension was 35.9 mm. No evaluated clinicopathological factor reached statistical significance for association with lymph node positivity. Age ≥ 50 years (OR, 4.38; *p* = 0.367), largest tumor dimension ≥ 40 mm (OR, 1.75; *p* = 0.672), and depth of invasion ≥ 10 mm among available cases (OR, 6.00; *p* = 0.242) showed higher point estimates but wide confidence intervals
**Conclusion**: In this single-center cohort, cervical lymph node positivity was frequent among patients with tongue squamous cell carcinoma. Although no factor reached statistical significance, older age, larger tumor dimension, and greater depth of invasion showed clinically relevant effect estimates. Larger studies with standardized pathological variables are needed to clarify predictors of cervical lymph node involvement

**FA26/357—Oral Surgery**
**Tongue Morphometry for Flap Design and Template Optimization in Neo-Tongue Reconstruction: A Cadaveric Study**
Thunyanun Nitadkitti ^1^, Ajchariya Sarovath ^1^, Chinnawut Suriyonplengsaeng ^2^ and Thiti Tantitham ^1^
*^1^* *Department of plastic and maxillofacial surgery, faculty of medicine, Mahidol university, Ramathibodi hospital, Bangkok, Thailand*
*^2^* *Department of anatomy, Faculty of science, Mahidol university, Bangkok, Thailand*
**Objectives**: Neo-tongue reconstruction for tongue cancer is regarded as a major and complex operation. Despite advances in microsurgical techniques, flap configuration and inset remain challenging. Numerous flap designs have been described; however, there is limited information on tongue dimensions and thickness. This cadaveric study aimed to evaluate tongue dimensions and thickness, examine their correlation with patient characteristics, and develop a practical anatomical template to guide neo-tongue reconstruction.
**Materials and Methods**: This study included 40 cadavers. Tongue dimensions (width, length, and volume) and oral cavity measurements were recorded and analyzed for correlations with age, sex, body weight, height, and BMI. The tongues were subsequently sectioned and flattened into a two-dimensional shape to assess regional thickness. All specimens were photographed and processed using a shape extraction program set at 90% pixel overlap to generate a representative tongue template.
**Results**: The mean tongue volume was 61.3 ± 13.8 cc, with a width of 5.7 ± 0.7 cm and length of 8.8 ± 1.2 cm. The intermolar width and anteroposterior length were 6.5 ± 1.2 cm and 7.8 ± 1.0 cm, respectively. Tongue volume and length showed significant positive correlations with body weight (r = 0.35, *p* = 0.026; r = 0.48, *p* = 0.002) and height (r = 0.37, *p* = 0.019; r = 0.40, *p* = 0.010), while tongue length was negatively correlated with age (r = −0.46, *p* = 0.003).
A neo-tongue reconstruction template measuring 11 × 11.25 cm was developed, with recommended thicknesses of 2.5 mm (mucosa), 3.6 mm (tip), 8–12 mm (body), and 8–9 mm (base).
**Conclusion**: Tongue dimensions, particularly volume and length, correlate with body size (weight and height), while tongue length decreases with age and tongue width varies by gender. These factors should be considered in individualized neo-tongue reconstruction planning.

**FA26/259—Orthognathic Surgery**
**Surgical Anatomy of the Inferior Alveolar Nerve: A Three-Dimensional Image Study**
Sotatsu Fukuyama ^1^, Takahiro Yamamoto ^2^ and Yuzo Komuro ^3^
*^1^* *Teikyo University Hospital, Chang Gung Memorial Hospital, Contour Clinic, Tokyo, Japan*
*^2^* *Contour Clinic, Tokyo, Japan*
*^3^* *Teikyo University, Tokyo, Japan*
**Objectives**: The mandible is frequently subjected to surgical procedures that carry a risk of injury to the inferior alveolar nerve (IAN) within the bone. Therefore, a precise understanding of its three-dimensional anatomical course is essential. This study aimed to evaluate the spatial relationship of the IAN within the mandible and provide clinically relevant information for safer surgical planning.
**Materials and Methods**: A retrospective three-dimensional imaging study was performed using cone-beam computed tomography data from 710 cases (mean age: 23.3 years) treated between 2010 and 2022. Patients were classified into four groups based on Angle classification (Class I, II, III) and the presence of cleft lip and palate. Linear measurements were obtained at the ramus, mandibular angle, and body to assess the relationship between the IAN canal and surrounding cortical bone.
**Results**: The distance from the lateral cortical bone to the IAN canal was greatest in the second molar region (mean 5.16 ± 1.42 mm), with the largest values observed in Class II patients (5.77 ± 1.07 mm). The distance from the mandibular angle to the canal was also longest in the Class II group (20.03 ± 2.63 mm) and greater in males (20.55 ± 3.02 mm). In all cases, the IAN canal was located above the modified Kamiishi line, which we defined as a reference line for safe osteotomy. In unilateral cleft lip and palate patients, no significant differences were found between cleft and non-cleft sides in most measurements.
**Conclusion**: This study provides three-dimensional anatomical insights into the course of the IAN that may improve surgical safety. While general patterns were identified, anatomical variability should be considered in clinical practice.

**FA26/262—Orthognathic Surgery**
**Artificial Intelligence-Assisted Orthognathic Surgery Planning in Skeletal Class III Patients: Early Accuracy and Model Performance**
Benjamin Massenburg ^1,2^, Elisa Vargiu ^3^, Jordan Swanson ^2^, Scott Bartlett ^2^ and Jesse Taylor ^2^
*^1^* *Rady Children’s Hospital, San Diego, CA, USA*
*^2^* *Children’s Hospital of Philadelphia, Philadelphia, PA, USA*
*^3^* *University of Padua, Padua, Italy*
**Objectives**: Accurate analysis and preoperative planning are critical to successful orthognathic surgery. We hypothesized that artificial intelligence (AI)–assisted surgical planning can achieve clinically acceptable accuracy in predicting osteotomy positioning and segmental bone movements, potentially reducing time required for clinical engineering workflows.
**Methods**: We retrospectively collected virtual surgical plans from patients with Class III malocclusion undergoing orthognathic surgery at a large craniofacial center. Surgical movements were derived from planned segment positions of the maxilla, mandible, and cranial base. A deep learning model was trained using preoperative bone models, cephalometric landmarks, and clinical features to predict segmental bone movements using 5-fold cross-validation (80/20 split). Accuracy was evaluated using mean squared error between predicted and clinically planned bone models.
**Results**: A total of 458 patients were identified, 214 patients with class III malocclusion that met inclusion criteria were included in the final dataset. For the maxilla, we found a mean translational error of 1.15 ± 0.73 mm and mean rotational error of 1.47 ± 0.85°. For the mandible, mean errors of 1.49 ± 0.89 mm and 1.71 ± 1.21° were observed. For craniometrics, mean errors of 0.04 ± 2.06° in the SNA angle, 0.12 ± 1.58° in SNB angle, and 0.16 ± 2.03° in the ANB angle were found.
**Conclusion**: Artificial Intelligence-assisted planning shows promise, though refinement is needed in both model design and postprocessing. The greatest prediction error was in the sagittal direction and in the occlusal plane angle. The AI-generated plans represent a modest starting point, that needs to be refined by clinical experts to optimize the virtual surgical plan. Prospective blinded clinical validation will be essential to determine whether these tools can meaningfully augment surgical planning in practice, and decrease the time needed for a clinical engineering call.
Conflict of interest disclosure
Receipt of grants/research supports: Synthes Research Grant

**FA26/338—Orthognathic Surgery**
**Determination of a Safe Zone for Intraoral Vertical Ramus Osteotomy**
Krishan Sarna ^1^, Fawzia Butt ^1^, Symon Guthua ^1^, Wei Cheong Ngeow ^2^, Martin Kamau ^1^
*^1^* *University of Nairobi, Nairobi, Kenya*
*^2^* *University of Malaya, Kuala Lumpur, Malaysia*
**Objectives**: Intraoral vertical ramus osteotomy (IVRO) corrects mandibular deformities and requires osteotomy placement posterior to the mandibular lingula (ML) to avoid injury to the inferior alveolar neurovascular bundle (IANB). Lateral landmarks such as the antilingula (AL), mid-waist point (MWP), and midpoint of the coronoid-gonion line (MCG), are used to estimate ML position, but the required posterior distance remains unclear and operator-dependent. Their relationship to the ML is highly variable, highlighting the need for population-specific data. This study, the first in an African population, evaluates the reliability of these landmarks and proposes a novel safe zone using simulated osteotomies.
**Materials and Methods**: A retrospective analysis of 152 CBCT scans (66 males, 86 females) was performed using Slicer 3D. The ML, AL, MWP, and MCG were identified, plotted on a polar plotSimulated osteotomies were then conducted, which allowed identification of a safe zone using this technique.
**Results**: Polar plot analysis showed the AL was widely distributed and anterior to the ML in 91.9% of cases (mean 5.73 ± 2.34 mm; max 14.40 mm). The MWP was more concentrated, antero-inferior to the ML (mean 4.62 ± 2.57 mm; max 13.40 mm), while the MCG was antero-superior (mean 2.94 ± 2.36 mm; max 10.48 mm). Absolute safety required osteotomy placement posterior to the AL by 15 mm, MWP by 14 mm, and MCG by 11 mm. Simulated osteotomies identified the safest zone at 1–5 mm from the posterior border, with safety decreasing beyond 5 mm and dropping below 50% at 12 mm. Regression analysis showed a significant negative relationship between increasing anterior distance and safety in IVRO.
**Conclusion**: The relationship between the ML and AL, MWP, and MCG is inconsistent. A safe osteotomy zone lies 1–5 mm from the posterior ramus border, with safety dropping below 50% beyond 12 mm.

**FA26/412—Orthognathic Surgery**
**Achieving Accurate Internal Maxillary Distraction Osteogenesis Among Patients with Cleft with Virtual Planning and 3D Printed Surgical Guide**
Jingtao Li ^1^
*^1^* *West China Hospital of Stomatology, Sichuan University, Chengdu, China*
**Objectives**: Compared with traditional orthognathic surgery, distraction osteogenesis provides greater extent of maxillary advancement but is of inferior accuracy. With the aid of virtual planning and 3D printed surgical guide for distractor placement, we set out to establish a workflow for internal maxillary distraction osteogenesis with accuracy and longterm stability
**Materials and Methods**: In virtual planning, Lefort I osteotomy was performed and the maxillae was moved to the target position according to the final occlusion. A distraction guide was fabricated by generating a pair of tubes parallel to the distraction vector. The tubes were positioned lateral to the maxillary tuberosities and connected to the occlusal plate. Both the skull and the distraction guide were 3D printed, with the aid of which distractors were pre-bended. During the surgery, maxillary osteotomy and alignment was performed as designed. The distractors were inserted into the tubes on the surgical guide and fixed with screws. One week after surgery, the maxilla was advanced at a rate of 0.8 mm/day. Once approach the target position, adjustment was made according to the final occlusion. The accuracy of the distraction was evaluated by overlapping the post-distraction CT scanning with virtual planning.
**Results**: A total of 54 patients with maxillary hypoplasia received internal distraction osteogenesis, and 30 were aided by the above mentioned distraction guide. The accuracy of distractor placement and horizontal and sagittal bone movement in the surgical guide group was significantly more accurate than the others. At six months after surgery, the horizontal, sagittal, and vertical discrepancy at maxillary landmarks as compared with the virtual planning was less than 1 mm among patients aided with distraction guide.
**Conclusion**: Internal maxillary distraction osteogenesis aided by virtual planning and distraction guide achieves accuracy comparable to traditional orthognathic surgery, serving as a powerful tool for the correction of cleft-ralted maxillary hypoplasia.

**FA26/499—Orthognathic Surgery**
**Postoperative Soft-Tissue Needs After Aesthetic Orthognathic and Facial Contouring Surgery**
Takahiro Yamamoto ^1^
*^1^* *Contour Clinic Tokyo, Tokyo, Japan*
**Objectives**: In East Asia, aesthetic orthognathic surgery is often performed not only to correct dentofacial deformity but also to achieve a smaller, more refined facial contour. However, reduction-oriented skeletal procedures may unmask or aggravate soft-tissue laxity. This study evaluated postoperative additional treatments after aesthetic orthognathic and facial contouring surgery.
**Materials and Methods**: A retrospective review was performed of 230 consecutive patients who underwent facial skeletal surgery between 23 November 2023 and 30 April 2026. Patients were classified as orthognathic surgery or sagittal split ramus osteotomy alone; orthognathic surgery or sagittal split ramus osteotomy with V-line or mandibular contouring; or orthognathic surgery with zygomatic reduction. Postoperative additional treatments were categorized as non-surgical treatment for soft-tissue laxity, surgical treatment for soft-tissue laxity, rhinoplasty, periocular surgery, injectable treatment, and plate removal.
**Results**: The mean age was 29.4 years. There were 188 female and 40 male patients. Thirteen patients underwent orthognathic surgery or sagittal split ramus osteotomy alone, 141 underwent additional V-line or mandibular contouring, and 76 underwent zygomatic reduction. Overall, 217 patients (94.3%) underwent facial contouring in addition to orthognathic surgery. Postoperative additional treatment was performed in 131 patients (57.0%). Non-surgical treatment for soft-tissue laxity was performed in 97 patients (42.2%), surgical treatment for soft-tissue laxity in 31 (13.5%), rhinoplasty in 13 (5.7%), periocular surgery in 14 (6.1%), injectable treatment in 6 (2.6%), and plate removal in 24 (10.4%). Additional treatment was more frequent after zygomatic reduction than without zygomatic reduction (63.2% versus 53.9%).
**Conclusion**: More than half of patients required postoperative additional treatment after aesthetic orthognathic and facial contouring surgery, most commonly for soft-tissue laxity. These findings suggest that reduction-oriented facial skeletal surgery should be planned as a comprehensive aesthetic strategy incorporating skeletal, soft-tissue, nasal, and periocular management.

**FA26/516—Orthognathic Surgery**
**Evaluation of Postoperative Stability of the Mandibular Midline Widening Osteotomy Technique with Concurrent Genioplasty for Correction of Bimaxillary Transverse Discrepancy**
Nithish reddy Sankepally ^1^, Mrunalini Ramanathan ^2^, Masako Fujioka-Kobayashi ^3^ and Takahiro Kanno ^3^
*^1^* *MNJ Institute of Oncology & Regional Cancer Center, Lakdikapul, Hyderabad, Telangana, India*
*^2^* *Meenakshi Ammal Dental College, Department of Oral and Maxillofacial Surgery, Chennai, India*
*^3^* *Department of Oral and Maxillofacial Surgery, Shimane University Faculty of Medicine, Izumo, Shimane, Japan*
**Objectives**: Bimaxillary transverse discrepancy is uncommonly reported, and surgical mandibular widening remains under-utilised due to concerns over relapse and TMJ sequelae. Conventional vertical symphyseal osteotomy is also linked to diastema and segment torsion. We assessed postoperative stability and condylar behaviour of a modified mandibular midline widening osteotomy with sliding genioplasty, performed alongside Le Fort I and bilateral sagittal split ramus osteotomy (BSSRO) setback, for mild to moderate transverse deficits.
**Materials and Methods**: This retrospective cohort included 21 patients (mean age 27.9 ± 10.8 years) operated between August 2020 and March 2024. All underwent single-stage Le Fort I, BSSRO setback, mandibular midline widening (range 2–5 mm), and genioplasty using bioresorbable osteosynthesis. Frontal cephalograms at preoperative, immediate, 1-, 3-, and 6-month timepoints measured intermolar and intergonial widths. Pre- and postoperative multidetector CTs assessed condylar angulation. Non-parametric statistical tests and Spearman correlation were applied (*p* < 0.05).
**Results**: Planned widening was 3.23 ± 0.76 mm. Intergonial width remained stable across all timepoints. Intermolar width was stable through 3 months; a statistically significant change at 6 months (*p* = 0.03) was attributable to 2D measurement variability rather than skeletal relapse. Condylar angulation showed no significant change (right *p* = 0.19, left *p* = 0.24) and weak correlations to widening. No TMJ symptoms occurred. One patient had an intraoperative gingival tear that healed by secondary intention without further complications.
**Conclusion**: BSSRO setback with mandibular midline widening and genioplasty is a stable, effective alternative to multi-piece osteotomies for mild-to-moderate (≤5 mm) transverse discrepancies, demonstrating excellent six-month skeletal stability and low postoperative morbidity.
Conflict of interest disclosure
Receipt of grants/research supports: AOCMF Start-up Grant 2025

**2FA26/1056—Orthognathic Surgery**
**Airway First, Aesthetics Always: Soft Tissue Profile Outcomes of Maxillomandibular Rotational Advancement Across Skeletal Classes in Asian OSA Patients**
Izzati Nabilah Binti Ismail ^1^ and Cheng Hui Lin ^2^
*^1^* *Taoyuan Chang Gung Memorial Hospital, Guishan District, Taoyuan City, Taiwan*
**Objectives**: Counterclockwise maxillomandibular advancement (CCW-MMA) is increasingly performed for obstructive sleep apnea (OSA) in Asian populations, yet its aesthetic implications across varying skeletal patterns remain poorly characterized. This study objectively evaluated soft tissue facial profile outcomes following CCW-MMA stratified by preoperative ANB skeletal classification, using the Zero Meridian Line (ZML) as the primary aesthetic reference.
**Materials and Methods**: A retrospective cohort study included 60 Asian adults (mean age 32.0 ± 9.9 years; 80% male) with OSA (AHI > 5) who underwent standardized CCW-MMA at a single tertiary centre. Patients were categorized into skeletal Class I (n = 21), Class II (n = 19), and Class III (n = 20). Preoperative and 6-month postoperative lateral cephalograms were analysed for skeletal parameters, soft tissue landmarks, and aesthetic indices including soft tissue subnasale (Sn’ZML) and pogonion distances to the ZML (Pog’ZML), Z-angle and facial convexity angle (FCA). Paired *t*-tests, Wilcoxon signed-rank tests, one-way ANOVA, and Tukey HSD post-hoc analyses were applied.
**Results**: Successful OSA correction was achieved across all groups with no significant intergroup difference in postoperative AHI (*p* = 0.117). Despite differing surgical vectors, no significant between-group differences were found in pog’ZML (*p* = 0.336), upper lip to E-line (*p* = 0.305), or lower lip to E-line (*p* = 0.425), indicating convergence of soft tissue lip and chin positions to a comparable aesthetic zone regardless of preoperative skeletal class. Meanwhile, SnZML (*p* = 0.018), NLA (*p* = 0.030), Z-angle (*p* = 0.049) and FCA (*p* = 0.009) showed significant between-group differences which reflects inherent class-specific skeletal geometry and soft tissue anatomy rather than aesthetic failure.
**Conclusion**: CCW-MMA produces favorable, harmonized soft tissue profiles in Asian OSA patients regardless of skeletal class, without inducing bimaxillary protrusion.

**2FA26/1176—Orthognathic Surgery**
**“The Banana Face”: Effect of Orthognathic Surgery on Mandibular Asymmetry**
Mike Leung ^1^ and Joyce Li ^1^
*^1^* *The University of Hong Kong, Hong Kong*
**Objectives**: This study uses the Arch Shape Ratio (ΔASR), a scale-independent metric derived from mandibular arch curvature measured along the outer cortical contour. The objective is to compare pre-operative and post-operative ΔASR values to evaluate surgical outcomes on mandibular asymmetry patients following orthognathic surgery.
**Materials and methods**: Pre-operative and post-operative cone-beam computed tomography (CBCT) scans of 80 consecutive orthognathic patients were analyzed. Inferior-view cortical contours were traced and fitted with splines in MATLAB, and arc and chord lengths were computed for each side to derive the Arch Shape Ratio (Larc/Lchord). Side-to-side differences in ASR (ΔASR) were used to define severity thresholds: ΔASR < 0.01 = mild, 0.01 ≤ ΔASR < 0.02 = moderate, and ΔASR ≥ 0.02 = high asymmetry.
**Results**: In the High Arch Shape Ratio (ASR) group, outcomes showed marked variability. Strong responders achieved near-symmetry with reductions approaching 99%, while others retained substantial residual asymmetry with reductions closer to 46%. Overall, the group demonstrated a mean pre-operative ASR of ≈0.030 (High ASR) and a mean post-operative ASR of ≈0.015 (Moderate ASR), corresponding to an average reduction of about 50%. In the Moderate Arch Shape Ratio (ASR) group, outcomes were characterized by greater variability. The mean reduction was approximately 40–45%, with some patients normalizing almost completely, achieving reductions of up to ≈97%. On average, the group demonstrated a mean pre-operative ASR of ≈0.015 and a mean post-operative ASR of ≈0.013, reflecting moderate improvement overall.
**Conclusion**: Surgical intervention demonstrated significant effectiveness in reducing mandibular asymmetry. However, residual asymmetry remained common, indicating that complete normalization is difficult to achieve especially in highly asymmetric group. These findings suggest that adjunctive treatments—such as soft-tissue management, or secondary procedures such as mandibular contouring, angle reduction—may be necessary to optimize outcomes in patients with persistent imbalance.

**2FA26/1177—Orthognathic Surgery**
**Simultaneous Endoscope-Assisted Intraoral Condylectomy and Orthognathic Surgery: A Surgery-First Approach for Condylar Tumors with Facial Asymmetry**
Minh Nguyen ^1^
*^1^* *School of Dentistry, Hanoi Medical University, Hanoi, Vietnam*
**Objectives**: Conventional management of condylar tumors with secondary facial asymmetry often involves extraoral approaches and delayed orthognathic surgery. This protocol can result in visible scarring, multiple surgical intervention, and psychological distress due to prolong pre-surgical orthodontics. We present a comprehensive, single-stage surgical protocol that addresses both the pathology and the aesthetic deformity simultaneously, prioritizing minimal invasiveness and immediate facial asymmetry treament through a surgery-first approach.
**Materials and Methods:** We present our clinical experience utilizing an entirely intraoral protocol for patients with condylar tumors and secondary facial asymmetry. The core surgical concept involves endoscope-assisted intraoral condylectomy combined with corrective orthognathic surgery in a single operation. Post-operative orthodontic management was tailored to individual patient needs. Particularly, in selected cases, we integrated clear aligner therapy for post-surgical orthodontic treatment. Clinical and radiographic follow-ups were conducted to evaluate surgical safety, aesthetic outcomes, and functional stability.
**Results**: The intraoral single-stage protocol effectively eradicated the tumors while restoring facial symmetry immediately. Across the clinical spectrum, there were no extraoral scars and no incidences of permanent facial nerve palsy, highlighting the safety of the endoscopic-assisted technique. The customized post-operative orthodontic phase particularly the integration of clear aligners in a subset of patients demonstrated excellent versatility, promoting efficient occlusal managemetn, superior oral hygiene, and high patient sastifaction. Long-term follow-up of initial cases confirms stable functional and aesthetic results.
**Conclusion**: Simultaneous endoscope-assisted intraoral condylectomy and orthognathic surgery via a Surgery-First Approach is a highly effective, safe, and scarless alternative for dentofacial deformity caused by condylar tumour. The adaptability of this protocol, particularly its successful integration with modern clear aligner therapy, offers a significant evolution in minimizing treatment morbidity and maximizing patient satisfaction.

**2FA26/1262—Orthognathic Surgery**
**Evaluation of the Stability of Maxillary Expansion After Segmental LeFort I Osteotomies Using Cone Beam CT**
Erik Tamberg ^1^, Chad Robertson ^1^, Nir El ^1^ and Vanessa Dairo-Singerr ^1^
*^1^* *Dalhousie University, Halifax, NS, Canada*
**Objectives**: Transverse maxillary deficiency is commonly managed surgically using surgically assisted rapid palatal expansion (SARPE) or segmental LeFort I osteotomies (SLF1Os) in the skeletally mature patient. While SARPEs effectively address transverse discrepancies, they do not permit simultaneous correction of maxillary anteroposterior (AP) or vertical positioning, often requiring secondary procedures. In contrast, SLF1Os enable concurrent correction of transverse, AP, and vertical dimensions, rendering them an attractive single-stage intervention. Their long-term stability has nonetheless been questioned, as they are considered prone to relapse and physiologically limited in expansion. Prior studies assessed this indirectly via dental relapse, which may inaccurately represent underlying transverse skeletal change. This prospective cohort study evaluates SLF1O three-dimensional stability using serial cone-beam computed tomography (CBCT).
**Materials and Methods**: Twenty patients (12M:8F; mean age 28.7 ± 9.8 years) undergoing SLF1Os for transverse maxillary deficiency were analyzed using CBCT at three time points: preoperatively (T1), splint removal four weeks postoperatively (T2), and orthodontic completion approximately one year postoperatively (T3). Skeletal expansion was measured between the medial greater palatine foramina, and dental expansion between the maxillary canine cusp tips and distobuccal cusps of the first molars.
**Results**: Mean skeletal expansion of 4.24 ± 1.47 mm was achieved postoperatively (T1 to T2). Skeletal relapse of 0.04 ± 0.53 mm was observed at T3. Mean dental expansion measured 0.02 ± 1.03 mm at the canines and 2.76 ± 1.66 mm at the first molars, with subsequent constriction of 1.98 ± 1.44 mm and 0.75 ± 1.10 mm, respectively, at T3. Age, sex, BMI, third molar extractions, and anteroposterior and vertical maxillary movements did not significantly affect skeletal relapse.
**Conclusion**: Findings demonstrate minimal skeletal relapse one year postoperatively, suggesting long-term transverse stability and indicating that SLF1Os are a reliable modality for maxillary constriction. Longer follow-up and expansions exceeding 7 mm would provide further insight into SLF1O utility.
Conflict of interest disclosure
Receipt of grants/research supports: Canadian Association of Oral and Maxillofacial Surgeons research grant.

**FA26/71—Reconstruction**
**Understanding Factors That Are Associated with Keloid Disease Recurrence Risk After Surgery**
Lamont Jones ^1^, Mingming Pan ^1^, Lane Dondalson ^1^, Mrudula Nair ^1^, William Mason ^1^, Dhananjay Chitale ^1^, Xiangguo Dai ^1^, Oghenefejiro Okifo ^1^, Johnathan Panzer ^1^ and Albert Levin ^1^
*^1^* *Henry Ford Health, Detroit, MI, USA*
**Objectives**: Keloid disease (KD) is an abnormal scaring condition which commonly occurs in the craniofacial region. Surgery is a first line treatment for KD but has high recurrence rates. The objective of this research is to determine factors that are associated with KD recurrence risk after surgery.
**Materials and Methods**: We completed a retrospective review of Henry Ford Health medical records, between 1990 and 2019, to (a) identify patients undergoing surgical treatment for KD and (b) to determine which had a subsequent recurrence.
A total of 1251 patients with 1697 keloid surgical records were analyzed. The association between demographic (e.g., sex, age, self-reported race) and keloid specific characteristics (e.g., status as a primary keloid or a recurrence of a previously treated keloid, number and maximum keloid size) and recurrence risk were evaluated using Cox proportional hazards models, accounting for multiple keloid surgeries from a single individual using a sandwich estimator of the variance.
**Results**: After surgical resection, recurrent keloids were 1.69 times (95%CI 1.34–2.13, *p* < 0.001) more likely to subsequently recur than a primary keloid. Among primary keloids an increase in the largest dimension of the keloid was associated with increased risk of recurrence, and increasing patient age was associated with a decreased risk of recurrence. African American (AA) patients undergoing surgery for a primary keloid(s) were 2.60 times (95%CI 1.71–3.95; *p* < 0.001) more likely than European American (EA) patients to have a keloid recurrence, and a similar hazard ratio (HR 2.36; 95%CI 1.23–4.55; *p* = 0.010) was also observed when individuals of all other race categories were combined and compared with EA patients.
**Conclusion**: To our knowledge, this is the first large scale epidemiologic study of keloid and patient factors that impact KD recurrence. Findings suggest the existence of a phenotype that is intolerant to surgical treatment, which requires future mechanistic investigations.
Conflict of interest disclosure
Receipt of grants/research supports: NIH, Henry Ford Health, AAFPRS grants for keloid research

**FA26/134—Reconstruction**
**Reconstructive Perspectives After Major Cranial Vault & Craniofacial Resections**
Vedran Zubčić ^1^
*^1^* *University Hospital Osijek, Osijek, Croatia*
**Objectives**: Reconstruction after craniofacial and cranial vault resections can be quite challenging. Complication rates figures are high. Conventional approaches to soft tissue and facial/cranial skeleton reconstruction can be long and exhausting for surgical teams, as well as imprecise. With the advancement of medical technologies, especially imaging technologies, and the further advancement of computer systems, advanced visualization techniques, including 3D printing and virtual or mixed reality is the logical next step in medical imaging of pathology. Their implementation enables clinicians to take a more personalized approach to patient treatment.
**Materials and Methods**: A retrospective review of 53 patients treated between 2017. and 2022. was conducted. Microvascular tissue transfer were used as the main reconstructive methods. Preoperative color Doppler and CT angio planning for perforator mapping were obtained in every case including creation of the 3D printed models and cutting guides for the cranial/facial skeleton resection and reconstruction as well.
**Results**: A wide range of facial/cranial defects were reconstructed in cases of cancer pateints, osteomyelitis, osteonecrosis and burn patients. This small series of patients describes represent our institutional approach to solve reconstructive requests in these challenging patients.
**Conclusion**: Main goal of the reconstructive surgeons work is an achievment of the waterthight seal between cranial cavity and upper aerodigestive tract, support brain/orbit and restore facial structure using vascularized tissue.

**FA26/280—Reconstruction**
**Evolving Trends in Cranioplasty: Insights from a Single-Institution Experience**
Cailin Cruess ^1^, Alexander Parker ^1^, Tessa Sapiro ^1^, Pharibe Pope ^2^, Adam Kaufman ^2^ and Natalie Justicz ^2^
*^1^* *University of Maryland School of Medicine, Baltimore, MD, USA*
*^2^* *Department of Otorhinolaryngology-Head and Neck Surgery, University of Maryland School of Medicine, Baltimore, MD, USA*
**Objectives**: To evaluate the association between timing of cranioplasty, implant type, and short-term postoperative outcomes in a single-institution cohort.
**Materials and Methods**: A retrospective chart review was performed of all patients who underwent cranioplasty between June and December 2025. Demographics, procedure details, and postoperative outcomes were analyzed.
**Results**: Sixty-one patients underwent cranioplasty (mean age 45.5 ± 18.2 years; 67% male), including 13% revision cases. The median interval between craniectomy and cranioplasty was 80.5 days (IQR 24.75–169). Autologous bone was more common within 12 weeks, whereas alloplastic implants predominated after 12 weeks (*p* = 0.022).
The postoperative complication rate was 20%, with no significant difference between autologous and alloplastic implants (*p* = 0.57). Eight patients returned to the OR (RTOR): three with alloplastic implants and five with autologous implants (*p* = 0.45). Mean hospital stay was 11.2 ± 16.9 days, with no difference by complication status (*p* = 0.10) or RTOR (*p* = 0.17). Craniectomy indications included trauma (51%), vascular pathology (26%), oncologic disease (20%), and infection (3%). Oncologic and infectious cases used alloplastic implants; 83% of vascular cases used autologous bone.
Trauma cases had five complications and four RTORs; oncologic cases had two complications and one RTOR; vascular cases had five complications and three RTORs; no adverse events occurred in infectious cases. Patients receiving alloplastic implants vs. autologous trended toward a shorter stay (7.7 vs. 15 days; *p* = 0.053). Dispositions included home (n = 41), acute rehab (10), subacute rehab (6), correctional facility (3), and transfer to an outside hospital (1).
**Conclusion**: Implant type was associated with timing and indication of reconstruction, with autologous bone used earlier and alloplastic materials later. No significant differences in complication rates or RTOR rates were detected between material groups. These findings support further investigation into optimal timing and material selection for cranioplasty.

**FA26/291—Reconstruction**
**Patient Specific Subperiosteal Implants for Primary Maxillary Reconstruction**
Giacomo De Riu ^1^, Andrea Biglio ^1^, Luigi Vaira ^1^ and Enrica Ligas ^1^
*^1^* *University Hospital of Sassari, University of Sassari, Sassari, Italy*
**Objectives**: Primary reconstruction of complex maxillary defects after oncologic resection remains challenging because restoration of facial contour, soft tissue support, oral function, and dental rehabilitation usually requires multiple staged procedures. The aim of this presentation is to evaluate the use of **patient-specific subperiosteal implants (PSI)** combined with reconstructive flaps for immediate maxillary reconstruction and simultaneous preparation for fixed dental rehabilitation.
**Materials and Methods**: A case series of 10 patients affected by malignant or locally aggressive maxillary tumors underwent maxillary reconstruction using patient specific subperiosteal implants designed with CT-based virtual planning. The PSI devices were used as a precise three-dimensional framework to stabilize bone grafts or flaps, support midfacial soft tissues, and provide prosthetically driven abutments for future fixed dental rehabilitation. Reconstructive techniques included iliac crest bone grafts and regional or free flaps according to defect extension. Follow-up ranged from 6 to 29 months (mean 18.3 months).
**Results**: The customized implants allowed accurate restoration of the maxillary skeletal contour with reliable fixation of reconstructive tissues. Adequate support of the upper lip, cheek, and midfacial soft tissues was achieved, contributing to improved aesthetics and oral competence. The integrated prosthetic design eliminated the need for secondary guided implant placement, reducing overall treatment time and biological burden. Early functional recovery in speech, mastication, and swallowing was observed. Surgical workflow was simplified, and patient costs were reduced compared with conventional staged protocols. No major implant-related failures were reported during the follow-up period.
**Conclusion**: Patient-specific subperiosteal implants represent a promising option for primary maxillary reconstruction after tumor resection. They provide immediate structural support, facilitate soft tissue restoration, and enable faster fixed dental rehabilitation in a single reconstructive pathway. This approach may improve quality of life while reducing morbidity and treatment duration. Larger series and longer follow-up are required to confirm long-term reliability.

**FA26/310—Reconstruction**
**Manjunath STRESS Classification: A Novel Framework for Patient-Specific Implant Failures in Maxillofacial Reconstruction**
Dr Manjunath N M ^1^
*^1^* *PMNM Dental college and Hospital, Bagalkot, Sri Madhusudan sai institute of Medical sciences, Chikballapur, Bangalore, India*
**Objectives**: To propose a novel, mechanism-based classification system for PSI failures and introduce a clinically relevant severity grading.
**Materials and Methods**: A conceptual framework was developed based on commonly reported complications in maxillofacial reconstruction. Failures were categorized into five domains using the Manjunath STRESS classification: Soft tissue, Technical, Radiation-related, Infective, and Structural. A parallel severity grading system (STRESS–G) stratifies complications into four levels based on clinical severity and management requirements.
**Results**: The STRESS classification provides a structured and comprehensive framework encompassing biological, technical, and biomechanical causes of PSI failure. It uniquely incorporates PSI-specific factors such as implant design and positioning, while distinguishing radiation-related complications as an independent domain. The STRESS–G grading system facilitates clinical decision-making by linking severity to management strategies, ranging from conservative treatment to implant removal and secondary reconstruction. The model also accommodates multifactorial overlap, reflecting realworld clinical scenarios.
**Conclusion**: The Manjunath STRESS classification offers a standardized, clinically applicable approach for evaluating PSI failures in maxillofacial reconstruction. It improves communication, guides management, and provides a foundation for future validation and outcome-based research.

**FA26/312—Reconstruction**
**Versatile Free Fibula Flap Modifications for Composite Mandibular Defects: A Clinical Evaluation**
Abdullah Masud ^1^
*^1^* *Dhaka Dental College & Hospital, Dhaka, Bangladesh*
**Objectives**: We present a clinical evaluation of three modified fibula-based strategies—fibula chimeric flap, fibula osteocutaneous flap combined with a lateral sural artery flap and fibula osteocutaneous flap combined with anterolateral thigh (ALT) flap in a consecutive series of 29 patients with composite mandibular defects and report the role of secondary regional flap salvage (pectoralis major myocutaneous flap) where primary skin coverage failed.
**Materials and Methods**: A retrospective cohort of 29 patients (2024–2025) undergoing primary mandibular reconstruction was analyzed. Twelve patients received a fibula-based chimeric flap, 10 were reconstructed with a fibula osteocutaneous flap combined with a lateral sural artery flap from the same lower limb, and 7 underwent reconstruction combining a fibula osteocutaneous flap with an ALT flap. Two patients who experienced failure of external skin defect coverage were successfully rescued with a pectoralis major myocutaneous (PMMC) flap. Complications, flap survival, and functional outcomes were recorded.
**Results**: Composite reconstruction was successful in 27 of 29 patients (93.1%), with primary free-flap survival in all but Two patients who experienced failure of external skin defect coverage were successfully rescued with a pectoralis major myocutaneous (PMMC) flap. Bone union and acceptable dental rehabilitation were achieved in all patient at a mean follow-up of 14 months.
**Conclusion**: Modified fibula-based flaps—including fibula chimeric, fibula osteocutaneous with lateral sural and fibula osteocutaneous with ALT—provide a highly versatile armamentarium for composite mandibular defects, allowing one-stage bony and soft-tissue reconstruction with high success rates. In the event of skin-paddle failure, regionally based PMMC flaps offer a dependable salvage strategy, reinforcing the importance of individualized algorithmic planning in complex head and neck reconstruction.

**FA26/365—Reconstruction**
**Developing an International Core Outcome Set for Mandibular Reconstruction After Mandibulectomy: A Multidisciplinary, Patient-Inclusive Delphi Study**
Saranda Ombashi ^1^, Brend Jonker ^1^ and Eppo Wolvius ^1^
*^1^* *Erasmus Medical Center, Rotterdam, The Netherlands*
**Objectives**: To improve comparability and quality of care for reconstructive surgery following mandibulectomy, this project aims to develop an international Core Outcome Set (COS) for mandibular reconstruction after mandibulectomy. To include both clinical outcomes and patient-reported outcome measures in a systematic and standardized manner, a Delphi process was set up. To provide the working group with relevant information, a scoping review identified relevant themes in mandibular reconstruction.
**Materials and Methods**: Full-text included studies were reviewed in detail. This process was performed in an iterative manner and done chronologically. Data extraction continued until data saturation was obtained, which was stated as that no new outcomes were identified in five consecutive studies. After the process of data extraction, coding took place. Each outcome was coded by applying a top-level code and one or more sub-codes. Coding was constructed by one researcher and cross checked by a second researcher.
**Results**: A total of 1467 studies were identified and 346 underwent full text screening. Extraction took place until data saturation. A total of 321 unique outcomes were identified and categorized into themes, including pre-operative (n = 132), peri-operative (n = 55), and postoperative (n = 240) domains, with sub-level classifications including clinical outcomes (n = 194), patient outcomes (n = 97), radiological outcomes (n = 24), and baseline variables (n = 33). Additional subcategories included complications (n = 72) and PROMs (n = 64). Findings resulted in a comprehensive preliminary outcome model.
**Conclusion**: The first steps towards an internationally developed COS for mandibular reconstruction after mandibulectomy have been conducted. The results provide a robust foundation for further development. Additional systematic reviews will identify appropriate case mix variables, important outcome measures and validated PROMs. This COS is expected to enhance data comparability, support benchmarking across centers, and accelerate innovation in reconstructive surgery, ultimately improving patient outcomes worldwide.

**FA26/375—Reconstruction**
**A Three-Dimensional Evaluation of Facial Esthetics Based on Facial Soft Tissue Landmarks: Two-Year Follow Up After Computer-Assisted Osteocutaneous Free Flap Jaw Reconstruction**
Xin Tong Li ^1^
*^1^* *The University of Hong Kong, Hong Kong*
**Objectives**:
Primary Objectives: To quantitatively evaluate the postoperative facial aesthetic outcomes based on facial soft tissue landmarks after computer-assisted jaw reconstruction with osteocutaneous free flaps
Secondary Objectives:
  (a) To quantify postoperative soft tissue changes over 1, 12 and 24 months.
  (b) To identify potential factors influencing aesthetic outcomes of jaw reconstruction with osteocutaneous free flaps, including preoperative diagnosis, disease staging, choice of flaps, postoperative adjuvant treatments.
**Materials and Methods**:
Study population: Patients diagnosed with oral malignant or benign tumors who underwent free flap reconstructive surgery at Queen Mary Hospital performed by the same surgical team from 2021 to 2024.
Study Design: Based on the patients’ 3dMD scanning data, 3dMDvultus software was used to evaluate sequential postoperative facial soft tissue changes and analyse their dynamic trends. For quantitative analysis, multiple facial soft tissue landmarks were selected, including bilateral Gonion, Chin, Cheilion, Zygion, Alare and Crista Philtri. Linear distances from these landmarks to the sagittal and coronal planes were measured at 1, 12 and 24 months postoperatively.
Moreover, relevant clinical data were recorded. IBM SPSS Statistics Version 30 was used to analyse the correlations between facial soft tissue changes and potential influencing factors, including radiotherapy and chemotherapy; location, type, stage of tumours; types and donor sites of osteocutaneous Flap; implants or other dental prostheses; brown classification; demographic and behavioural factors.
**Results**: The study is currently in the data collection phase. Quantitative data assessing soft tissue changes after reconstructive surgery will be presented at the congress.
**Conclusion**: This study introduces a novel approach to assess soft tissue changes following reconstructive surgery, providing a new perspective for evaluating surgical outcomes and long-term stability.

**FA26/379—Reconstruction**
**Jaw-in-a-Day: Single-Stage Mandibular Reconstruction with Virtual Surgical Planning, Free Fibula Flap, and immediate Implant-Supported Prosthesis Following Segmental Resection of Ameloblastoma**
Jirapat Supamitkitja ^1^
*^1^* *Mahidol University, Bangkok, Thailand*
**Objectives**: To describe the clinical outcomes of a fully digital-guided “jaw-in-a-day” approach—integrating virtual surgical planning, CAD/CAM patient-specific components, immediate free fibula reconstruction, and same-day implant-supported prosthetic delivery—in a patient undergoing segmental mandibulectomy for ameloblastoma.
**Materials and Methods**: A 51-year-old Thai female with biopsy-confirmed ameloblastoma of the right mandible (multilocular hypodense lesion, 31 × 12 mm, extending from tooth 31 to 46 with buccolingual expansion, no cortical perforation) underwent right segmental mandibulectomy with immediate jaw-in-a-day reconstruction. Preoperative digital workflow included VSP-guided osteotomy simulation, CAD/CAM patient-specific titanium reconstruction plate replicating native mandibular and chin contour, stereolithographic resin cutting guides, resin implant surgical guides, and a prefabricated provisional prosthesis with temporary copings. Surgery employed a simultaneous dual-team approach. The fibula was osteotomized into two segments (23.4 mm and 32.8 mm) using patient-specific cutting guides and assembled ex vivo onto the customized plate. Three Neodent implants (4.0 × 10 mm) with multi-unit abutments (height 5 mm, diameter 4.8 mm) were placed into the fibula construct using the resin surgical guide prior to inset. Microvascular anastomosis was performed between the peroneal artery and facial artery. Provisional prosthesis was delivered via intraoral temporary coping pickup on the day of surgery.
**Results**: Complete flap survival was achieved with no vascular compromise or flap-related complications. All three implants achieved primary stability with no implant loss at 2-month follow-up. Functional occlusion was established on the day of surgery. Progressive improvement in masticatory function and occlusal stability was documented at every follow-up visit, with no peri-implant pathology or hardware failure observed.
**Conclusion**: A fully digital-guided jaw-in-a-day workflow is technically feasible and yields favorable early oncologic and functional outcomes in mandibular ameloblastoma, enabling immediate aesthetic and masticatory rehabilitation from the day of tumor resection within a single surgical episode.

**FA26/382—Reconstruction**
**Establishing Maxillofacial Microvascular Services in the Northern Province of Sri Lanka-Young Surgeon’s Perspective**
Liyanaarachchige Anushan Hiranya Jayasinghe ^1^, Masilamany Thavarajah ^2^ and Selvarajah Kirushihan ^1^
*^1^* *District General Hospital Vavuniya, Vavuniya, Sri Lanka*
*^2^* *Teaching Hospital Jaffna, Jaffna, Sri Lanka*
**Objectives**: To evaluate the feasibility of establishing a sustainable maxillofacial microvascular reconstructive service in a resource-limited setting in the Northern Province of Sri Lanka. The primary challenges included initiating the service, maintaining consistency, and achieving clinically significant improvements in patient outcomes despite infrastructural and workforce constraints.
**Materials and Methods**: Between November 2025 and April 2026, over a six-month period, District General Hospital Vavuniya performed seven head and neck reconstructions using soft tissue microvascular flaps. Of the seven cases, six followed ablative surgery for oral malignancies, while one case addressed severe atrophic changes in facial soft tissues. A single-team surgical approach was adopted in most cases due to limited availability of trained surgeons within the region.
**Results**: Among the seven reconstructions performed—comprising five radial forearm free flaps (RFFF), one perforator anterolateral thigh (ALT) flap, and one vastus lateralis muscle flap—there was a 100% flap survival rate. No cases required re-exploration, and there were no postoperative infections observed in the head and neck region. Donor sites for RFFFs were managed with skin grafting, including four full-thickness abdominal grafts and one split-thickness graft from the thigh. Notably, three of the full-thickness grafts demonstrated greater than 80% graft loss, necessitating prolonged wound care and delayed healing.
**Conclusion**: Establishing a maxillofacial microvascular service in a single-surgeon district general hospital with limited resources presents considerable logistical and clinical challenges. Constraints include restricted operating time, minimal intensive care facilities, and a shortage of trained personnel, making a two-team surgical approach unfeasible in most cases. Additionally, reliance on visiting surgeons from the limited number of regional hospitals may disrupt services elsewhere. Nevertheless, despite these challenges, the successful outcomes and substantial improvements in patient quality of life underscore the value and feasibility of developing such specialized services in underserved regions.

**FA26/433—Reconstruction**
**The Future Face of Reconstruction: Artificial Intelligence in Head and Neck Surgery**
Hannah Arnantha ^1^ and Hitesh Bansal ^2^
*^1^* *St George’s, Epsom and St Helier Hospital Group, London, UK*
*^2^* *London North West University Healthcare NHS Trust, London, UK*
**Objectives**: Artificial intelligence (AI) and digital technologies are increasingly shaping head and neck reconstructive surgery, particularly through advances in virtual surgical planning (VSP). This review aims to evaluate current applications of AI in reconstruction, with a focus on VSP and its role in improving surgical precision, efficiency, and patient-specific outcomes.
**Materials and Methods**: A narrative literature review was conducted examining AI applications across reconstructive workflows, including preoperative planning, intraoperative guidance, and postoperative outcome prediction. Particular emphasis was placed on VSP, computer-aided design/computer-aided manufacturing (CAD/CAM), and AI-driven imaging analysis in microvascular reconstruction.
**Results**: AI-enhanced VSP has become central to modern head and neck reconstruction, enabling the creation of patient-specific 3D anatomical models and preoperative simulation of complex resections and reconstructions. These systems facilitate accurate osteotomies, optimal flap design, and improved spatial alignment, particularly in mandibular and maxillary reconstruction. Integration of AI with VSP allows automated segmentation of imaging data, prediction of surgical margins, and optimisation of reconstructive strategies based on anatomical and functional parameters. Intraoperatively, computer vision and navigation systems provide real-time guidance, improving precision and reducing operative variability. AI applications also extend to perforator identification and vascular mapping, supporting flap selection and microvascular planning. Collectively, these technologies contribute to reduced operative time, improved functional and aesthetic outcomes, and enhanced surgical reproducibility. However, challenges remain, including limited high-quality datasets, variability in imaging protocols, cost barriers, and concerns regarding data standardisation and algorithm transparency
**Conclusion**: AI-driven VSP represents a paradigm shift in head and neck reconstructive surgery, enabling highly personalised and precise surgical planning. While current applications are largely assistive, continued advancements in automation, data integration, and real-time decision support are likely to further enhance surgical outcomes. Future progress will depend on robust validation, interdisciplinary collaboration, and the development of explainable AI systems to ensure safe and widespread clinical adoption.

**FA26/471—Reconstruction**
**Recipient Vessel Strategies in Challenging Head and Neck Reconstruction: End-to-Side Anastomosis to the Carotid Artery and Salvage Approaches in Vessel-Depleted Necks**
Chun Chia Chen ^1^
*^1^* *Chi Mei Medical Center, Tainan*
**Objectives**: To describe the feasibility, safety, and outcomes of using the carotid artery (common or external) and internal jugular vein as recipient vessels for free flap transfer in vessel-depleted necks, particularly in patients undergoing secondary microsurgical reconstruction for challenging head and neck reconstruction patients.
**Materials and Methods**: A retrospective analysis was conducted on 234 patients with recurrent squamous cell carcinoma of the buccal region who had undergone previous free flap reconstruction and required a second free anterolateral thigh (ALT) flap transfer. Among these, 41 patients (17.5%) lacked suitable ipsilateral recipient vessels due to prior radical neck dissection and were assigned to the study group, in which end-to-side anastomosis to the carotid artery and internal jugular vein was performed. The remaining 193 patients, who utilized minor branches of neck vessels, served as the control group. Perioperative and postoperative complications, flap survival rates, and neurological events were analyzed and compared between groups. In this article, cases presentation showed challenging head and neck cancer patients as well.
**Results**: Overall flap survival across all 234 cases was 96.2%. The overall complication rate was 15.8%, comprising flap-related complications (12.4%), infection (2.6%), partial flap necrosis (1.7%), and orocutaneous fistulae (2.6%). The complication rate in the study group was 9.8% (4/41), with no statistically significant difference in free flap failure rates between groups.
**Conclusion**: End-to-side anastomosis to the carotid artery and internal jugular vein represents a reliable and hemodynamically effective recipient vessel strategy for secondary free flap reconstruction in vessel-depleted necks. These vessels should be considered a viable salvage option as well as a last resort in challenging head and neck reconstruction.

**2FA26/1153—Reconstruction**
**Secondary Reconstruction of Malar Asymmetry and Medial-Inferior Orbital Defects Using a Custom PEEK Implant and Template-Bent Mesh: A Case Report**
Minh Bui ^1^, Chanh Le ^1^, Tuan Van Nguyen ^1^ and Trung Tran ^1^
*^1^* *National Hospital of Odonto-Stomatology, Ho Chi Minh City, Vietnam*
**Objectives:** Management of severe post-traumatic maxillofacial deformities remains a significant challenge, especially after multiple suboptimal surgeries. This case report demonstrates a precise secondary reconstructive strategy combining a custom PEEK implant and a template-bent titanium mesh for complex malar-orbital defects.
**Materials and Methods:** A 27-year-old male presented with a 9-mm left enophthalmos and severe depression of the left malar and lateral orbital rim. He had a history of comminuted left zygomaticomaxillary complex and Le Fort I fractures. Despite initial open reduction and internal fixation 4 years ago and a secondary orbital floor reconstruction 2 years ago, aesthetic outcomes remained unsatisfactory. Preoperative CT and virtual surgical planning (VSP) utilizing Mirroring and Boolean operations precisely quantified the hard-tissue deficiency. A custom PEEK implant was fabricated for the malar and lateral orbital contour, alongside a 3D-printed bending guide for the orbital titanium mesh. Surgical access included hemicoronal, maxillary vestibular, and extended transconjunctival approaches with retrocaruncular extension. Following hardware removal, the PEEK implant was fixed, and the prebent mesh was placed to reconstruct the medial-inferior orbital walls, successfully closing the inferior orbital fissure.
**Results:** The postoperative course was uneventful. At the 3-month follow-up, the patient exhibited normal ocular motility. Left enophthalmos was significantly improved, with a residual asymmetry of only 2 mm compared to the unaffected side, as measured by Hertel exophthalmometry. Postoperative CT scans confirmed accurate placement of both the custom PEEK implant and the prebent mesh, demonstrating high fidelity to the VSP design. The patient reported high satisfaction with the postoperative aesthetic outcomes and facial symmetry.
**Conclusion:** The synchronized application of a custom PEEK implant for malar contours and a template-bent titanium mesh for complex multi-wall orbital defects provides a highly predictable solution. This VSP-guided strategy successfully addresses complex functional deficits and demanding aesthetic requirements in secondary maxillofacial reconstruction.

**2FA26/1154—Reconstruction**
**Does Neoadjuvant Chemotherapy Increase Reconstructive Risk? A Comparative Analysis of Surgical Efficiency and Microvascular Success in Advanced OSCC**
Yasmin Ghantous ^1^
*^1^* *Mahoz Tzafon, Tzafon Medical Center, Bar Illan University, Tiberies, Israel*
**Objective**: To investigate the “double-edged sword” effect of Neoadjuvant Chemotherapy (NACT) by evaluating its impact on surgical efficiency and postoperative morbidity in patients undergoing complex free-flap reconstruction for advanced oral cavity squamous cell carcinoma (OSCC).
**Methods**: A retrospective comparative study was conducted on 86 patients (NACT cohort: n = 20; Primary Surgery cohort: n = 66) undergoing microvascular free tissue transfer. Primary outcomes included surgical efficiency (ischemic time, total operative duration) and postoperative complications (major microvascular events vs. minor wound issues).
**Results**: The NACT group presented with significantly more advanced disease (100% Stage IV vs. 39.4%; *p* < 0.001). Despite this higher tumor burden, the NACT cohort demonstrated significantly shorter mean ischemic times (39.0 ± 7.5 vs. 52.5 ± 17.9 min; *p* = 0.001) and reduced operative durations (7.5 ± 0.8 vs. 8.6 ± 1.3 h; *p* = 0.001). Total complication rates were comparable (35% vs. 33.3%). However, minor wound-related complications were significantly more frequent in the NACT group (30% vs. 4.5%; *p* = 0.004). The flap salvage rate following re-exploration was high and comparable between groups (80.0% vs. 85.7%).
**Conclusion**: NACT is a safe and efficient strategy for advanced OSCC. While it increases minor wound morbidity—likely due to microvascular cytotoxicity—it does not compromise major microvascular integrity. The use of robust donor sites like the Scapula Chimeric flap can mitigate NACT-related technical challenges, leading to an “Efficiency Paradox” where advanced cases are managed with greater procedural predictability.

**2FA26/1160—Reconstruction**
**Hypopharyngeal Reconstruction: A Flap Series and a Technology-Assisted Protocol Integrating Virtual Planning, Doppler Ultrasound, Indocyanine-Green Fluorescence, and Augmented Reality**
Alicia Dean ^1^, Concepcion Centella ^1^, Abel Marin Martin ^1^, Orlando Estevez ^1^ and Francisco Jesús Alamillos ^1^
*^1^* *Oral and Maxillofacial Surgery Department, University Hospital Reina Sofía, Córdoba, Spain*
**Objectives**: To report oncological and functional outcomes of a microsurgical and pedicled hypopharyngeal reconstruction series, and to describe a technology-assisted protocol: virtual planning, Doppler ultrasound, indocyanine-green (ICG) fluorescence and augmented reality, progressively incorporated into our recent cases.
**Materials and Methods**: Retrospective study of 16 patients (13 men, 3 women) undergoing hypopharyngeal reconstruction (11 immediate, 5 secondary for pharyngostoma) for squamous cell carcinoma. Variables: TNM stage, defect type, flap, complications, perforators, time to oral intake, and swallowing at 1, 3 and 12 months. The technology-assisted protocol was applied: multimodal image fusion (CT, PET-CT, MRI) for virtual planning (Elements, Brainlab^®^ navigation system); perforator mapping by lower-limb CT angiography, Doppler ultrasound and handheld Doppler; augmented reality for planning, consensus and teaching; and intraoperative ICG fluorescence at three time points: identifying cutaneous perforators before flap incision, assessing viability after elevation, and confirming perfusion after anastomosis.
**Results**: 16 patients, mean age 67.3 years, 81.2% male; 75% had prior radiotherapy. Anterolateral thigh (ALT) flap was used in 14 (87.5%), radial forearm in 1 (6.25%) and supraclavicular in 1 (6.25%). Free-flap viability was 100%. Three pharyngocutaneous fistulas (18.75%) occurred, all managed conservatively: two healed spontaneously and one was closed under local anaesthesia. No major donor-site complications. One patient died from carotid blowout on postoperative day 10. Time to oral intake: 16 days (ALT), 13 (radial), 15 (supraclavicular). At 12 months, 87.5% maintained functional oral swallowing without enteral support. Preoperative Doppler ultrasound and ICG fluorescence agreed on perforator location; fluorescence also helped confirm flap viability.
**Conclusion**: This microsurgical and pedicled flap series confirms that hypopharyngeal reconstruction is safe and effective, with high viability and satisfactory swallowing; flap choice should be individualised. The technology-assisted protocol, proved feasible and added objective intraoperative information. Larger prospective studies are needed to quantify its impact on complications and function.
Conflict of interest disclosure:
Participation in a company sponsored speaker’s bureau: In 2021

**2FA26/1180—Reconstruction**
**Outcomes of Lateral Arm Flap, Medial Sural Artery Perforator Flap and Radial Forearm Flap in Tongue and Buccal Mucosa Reconstruction; A Comparative Study**
Md. Mostafizur Rahman ^1^ and Sabiha Alam ^2^
*^1^* *Shaheed Suhrawardy Medical College Hospital, Dhaka, Bangladesh*
*^2^* *Delta Hospital Ltd., Dhaka, Bangladesh*
**Objectives**: Reconstruction of tongue and buccal mucosal defects is essential for restoring speech, swallowing, and aesthetics following oncologic resection. While the radial forearm flap (RFF) remains widely used, increasing attention is being given to the lateral arm flap (LAF) and medial sural artery perforator (MSAP) flap due to concerns over RFF donor-site morbidity. This study compares outcomes among these three flaps in oral cavity reconstruction.
**Materials and Methods**: A comparative study was conducted involving 76 patients undergoing reconstruction with RFF, LAF, or MSAP flaps for tongue, buccal mucosa and palatal defects between 2020 to 2025. Parameters assessed included flap viability, operative time, functional recovery (speech and swallowing), donor-site morbidity, and patient satisfaction. Data were analyzed using SPSS.
**Results**: 76 patients were included: 32 RFF, 24 LAF, and 20 MSAP flaps. Over all flap survival was 93.4%. MSAP flaps demonstrated lower donor-site morbidity and superior cosmetic outcomes. RFFs had shorter harvest times but higher donor-site complications. LAFs provided reliable coverage with intermediate results in terms of function and donor-site morbidity. Functional outcomes were comparable across groups, with slight improvements in speech intelligibility and oral intake noted in the MSAP group.
**Conclusion**: RFF, LAF, and MSAP flaps are all viable for oral cavity reconstruction. The MSAP flap offers an optimal balance of functional restoration and donor-site aesthetics, making it a strong alternative to the traditional RFF. Flap selection should be individualized based on defect characteristics and patient factors.

**2FA26/1199—Reconstruction**
**Surgical and Reconstructive Strategies of Orbital Exenteration: A Single-Center Retrospective Cohort Study**
André Eckardt ^1^, Efthymios Papazacharias ^1^, Philippe Korn ^1^, Michael-Tobias Neuhaus ^1^, Nils-Claudius Gellrich ^1^
*^1^* *Hannover Medical School, Department of Oral & Maxillofacials Surgery, Hannover, Germany*
**Objectives**: Tumors of the orbit present a significant diagnostic and therapeutic challenge. Orbital exenteration is a radical surgical procedure mainly performed for advanced orbital and periorbital malignancies. Despite its disfiguring nature, it remains an important surgical option to control the disease and improve survival. Our retrospective cohort study evaluates the indications, surgical and reconstructive outcomes, and overall survival of patients who underwent orbital exenteration.
**Materials and Methods**: Medical records of patients who underwent orbital exenteration during a twenty-year period were reviewed. Data collection included patient demographics, histology, extent of tumor resection, surgical reconstruction, adjuvant treatment, and overall survival. Descriptive and survival analysis were performed.
**Results**: A total of 60 patients have been included so far, with a median age of 64.0 years (45.0 to 71.0). Main indications for orbital exenteration revealed a variety of malignant orbital and periorbital tumors, with squamous cell carcinoma being the most type, followed by adenocarcinoma. Surgical reconstruction ranged from local to microvascular flap surgery, and in selected patients rehabilitation with implant-retained epistheses was performed. Adjuvant radiotherapy was indicated in 20 patients. Median overall survival was 137.0 months (95% C.I.: 76.7–197.3).
**Conclusion**: Although immunotherapy seems to play an emerging role in the management of orbital and periorbital tumors, orbital exenteration remains an important part of our surgical portfolio to manage advanced tumors of the orbit. The surgical procedure can be associated with significant morbidity, but it can provide effective local tumor control which translates into longer patient survival. Rehabilitation of orbital defects with implant-retained prostheses plays an essential part to improve patients individual quality of life.

**2FA26/1296—Reconstruction**
**Primary Reconstruction of the Total Facial Nerve and Radical Parotidectomy Defects with Free Anterolateral Motor Neuromyocutaneous Flap**
Dinesh Kadam ^1^
*^1^* *A J Institute of Medical Sciences, Mangalore, India*
**Introduction**: High-grade parotid malignancies often require radical surgical resection and adjuvant radiation, resulting in complex skin and soft tissue defects and total facial nerve loss. Options hitherto known are nonvascular nerve grafts to bridge the facial nerve trunk to branches, along with free flap, either primarily or staged functional muscle transfer. We describe utilizing the nerve to vastus lateralis muscle as a source of vascularized nerve graft for facial nerve and defect reconstruction.
**Methods**: Four patients with complex defects following radical parotid malignancy resection involved loss of skin, facial nerve from trunk to all 5 branches, and soft tissue. The Anterolateral thigh flap was harvested as per the skin defect. The nerve to the vastus lateralis and its distal multiple branches were preserved along with adequate muscle tissue to support the nerve. The proximal end was coapted to the available proximal trunk of facial nerve and all distally with the multiple divisions of nerve to vastus. The flap was inset and anastomosed to neck vessels.
**Results**: All 4 patients were female, aged 21 to 56 years. All flaps had uneventful survival. 3 patients regained facial animation at 12 months post-op. One patient succumbed to the recurrence within 6 months. Three patients received adjuvant Radiation. One patient underwent secondary debulking of the flap.
**Conclusion**: The nerve to the vastus lateralis mimics the facial nerve branching pattern, with 5–7 branches from the proximal trunk, and its length serves as an ideal source of vascularized nerve graft for reconstruction primarily. The vastus lateralis muscle also acts as a functional muscle transfer to the defect and protects the nerve from external radiation therapy. Furthermore, the rest of the complex defect reconstruction is achieved with the ALT flap itself. This innovative approach offers a primary reconstruction of the facial nerve and complex defects.

**FA26/63—Technology**
**Augmented Reality-Assisted Planning and a Novel Three-Jaw Microclamp in ALT Flap Reconstruction: A Randomised Clinical Trial**
Aakash Kohli ^1^
*^1^* *Aiims, Jodhpur, India*
**Objectives**: To evaluate the effectiveness of a novel three-jaw adventitia-holding (TADH) microclamp for intraoperative performance in microvascular anastomosis, and to assess the adjunctive role of augmented reality (AR) in preoperative planning for anterolateral thigh (ALT) flap reconstruction.
**Materials and Methods**: A single-centre, parallel-group randomised clinical trial was conducted in 40 patients undergoing ALT flap reconstruction. Patients were allocated into two groups: control (Doppler-guided planning with Acland microclamps, n = 20) and experimental (AR-assisted planning with TADH microclamps, n = 20). Primary outcomes included arterial and venous clamp and suture times. Secondary outcomes included flap harvest time, vascular visualization, ease of use, and need for assistance.
**Results**: The experimental group demonstrated improved vascular visualization compared to Doppler mapping (75 ± 6.4 vs. 49 ± 3.6, *p* < 0.001) and reduced flap harvest time (126 ± 4.5 vs. 154 ± 5.9 min, *p* < 0.001). The TADH microclamp was associated with significant reductions in arterial and venous clamp and suture times (all *p* < 0.001). Surgeon-reported ease of use was higher, and need for assistance was significantly lower in the experimental group (*p* < 0.001). The TADH microclamps did not require flipping to enable suturing of the posterior walls of vessel. Flap survival rates were comparable between groups.
**Conclusion**: The TADH microclamp significantly improves intraoperative efficiency in microvascular anastomosis. Augmented reality serves as a useful adjunct for preoperative planning by enhancing vascular visualization. This combined workflow approach demonstrates potential to improve surgical efficiency and warrants further validation.
Conflict of interest disclosure
Stock shareholder: The author has a financial interest in the development of related surgical technology.

**FA26/298—Technology**
**In Vitro and In Vivo Application of Augmented Reality in Maxillo-Facial Surgery: Pushing the Boundaries of Surgical Navigation**
Diana D’Alpaos ^1^, Francesco Ceccariglia ^1^, Laura Cercenelli ^2^, Emanuela Marcelli ^2^, Giovanni Badiali ^3^ and Achille Tarsitano ^3^
*^1^* *Oral and Maxillofacial Surgery Unit, IRCCS Azienda Ospedaliero—Universitaria di Bologna, Via Albertoni 15, 40138 Bologna, Italy*
*^2^* *Edimes Lab DIMEC Alma Mater Studiorum, University of Bologna, Bologna, Italy*
*^3^* *Maxillofacial Surgery Unit, Head and Neck Department, IRCCS Azienda Ospedaliera Universitaria di Bologna, Department of Biomedical and Neuromotor Sciences, Alma Mater Studiorum University of Bologna, Bologna, Italy*
**Objectives**: The aim of this study is to give an insight into the application of augmented reality (AR) to Maxillofacial surgery and to explore potentials and qualities, as well as drawbacks, of this technology. This research aims at demonstrating the feasibility and assessing the intraoperative accuracy of an augmented reality-based surgical navigation system. Following an in vitro phase using stereolithographic models to simulate AR-guided maxillary osteotomies, a testing phase was performed in the operating room to evaluate the system’s performance in a clinical setting.
**Materials and Methods**: Three head-mounted displays (HMDs) (VOSTARS^®^, HoloLens 2^®^, Magic Leap^®^) were tested among the years to simulate maxillary and mandibular osteotomies in vitro. The above-mentioned HMDs were then used for observational and interventional testing phases in 15 in vivo procedures, including orthognathic and oncological surgeries. The surgeon used the VSP displayed on the headset to trace the corticotomies. The accuracy of AR-guided corticotomies was compared with osteotomies made using CAD-CAM cutting guides measuring the discrepancy between planned and performed.
**Results**: We found that performing corticotomies on maxillary bones with augmented reality guidance is feasible and applicable in a surgical setting. AR seems to be a reliable tool with good accuracy, (mean range of about ±2 mm), as demonstrated by the comparison with traditional methods, such as osteotomies performed with CAD-CAM cutting guides. There was no increase in intraoperative time, showing adaptability to an intraoperative setting.
**Conclusion**: This technology offers advantages, particularly for less experienced surgeons, such as providing visibility without the need to divert attention from the surgical field and offering personalized intraoperative guidance, even in deep planes. AR-assisted navigation appears promising in cranio-maxillofacial surgery and warrants further investigation in larger cohorts and more complex surgical scenarios.

**FA26/300—Technology**
**Deep Learning Frameworks for Automated Classification of Odontogenic Lesions on Panoramic Radiographs: A Single-Stage and Two-Stage Approach**
Natchaya Tansuphaswasdikul ^1^
*^1^* *Faculty of Dentistry Khon Kaen University, Amphoe Mueang Khon Kaen, Thailand*
**Objectives**: To compare single-stage and two-stage deep learning frameworks for classifying normal jaw, OKC, and ameloblastoma, and to identify optimal model development strategies under limited-data conditions, including the effects of dataset size, cross-validation scheme, fine-tuning level, and data distribution.
**Materials and Methods**: This retrospective study included two panoramic radiograph datasets (N = 150 and N = 200) with three classes: normal, OKC, and ameloblastoma. Six pretrained architectures (EfficientNetV2, InceptionV3, MobileNetV3, ResNet18, ResNet50, YOLOv11) were evaluated using two diagnostic strategies: (1) single-stage three-class classification and (2) two-stage sequential classification (normal vs. pathology, followed by OKC vs. ameloblastoma). A two-phase design was implemented. Phase 1 benchmarked models across dataset size, fine-tuning level (0%, 50%, 100%), and cross-validation (5- and 10-fold). Phase 2 simulated deployment using fixed train-validation-test splits (80-10-10 and 60-20-20). Performance was assessed using accuracy, macro F1-score, sensitivity, and specificity.
**Results**: In Phase 1, the best single-stage model (ResNet18) achieved 0.610 ± 0.057 accuracy, while InceptionV3 and YOLOv11 performed best in Stage 1 (0.727 ± 0.043) and Stage 2 (0.700 ± 0.087), respectively. In Phase 2, the best single-stage model (InceptionV3) achieved accuracy 0.800 and macro F1-score 0.747. The best two-stage pipeline (EfficientNetV2-S + YOLOv11) achieved similar accuracy (0.800) but higher macro F1-score (0.767) and specificity (0.900), with more balanced sensitivity and precision.
**Conclusion**: Diagnostic strategy significantly influences AI performance. While single-stage and two-stage approaches achieved similar accuracy, the two-stage framework provided more balanced and clinically interpretable results. These findings support the use of modular, clinically aligned AI pipelines for panoramic radiograph screening under limited-data conditions.

**FA26/405—Technology**
**Precision Maxillary Recontouring in Craniofacial Fibrous Dysplasia Using 3D-Printed Depth Guides and Real-Time Navigation**
Sireethon Saithong ^1^, Boworn Klongnoi ^1^
*^1^* *Mahidol University, Bangkok, Thailand*
**Objectives**: Maxillary fibrous dysplasia remains challenging to recontour due to poorly defined margins and limited surgical access. Traditional free-hand techniques often lack predictability, particularly when attempting to restore symmetry. This report describes the use of 3D-printed depth guides together with real-time navigation to improve intraoperative control during recontouring via an intraoral approach.
**Materials and Methods**: A 40-year-old male with a 25-year history of fibrous dysplasia involving the left maxilla and zygomatic body presented with facial asymmetry. Imaging demonstrated a diffuse ground-glass lesion extending from the alveolar process to the orbital floor, with the infraorbital canal still identifiable.
Virtual planning was performed using the contralateral side as a reference. A 3D-printed depth guide was fabricated with predefined drilling depths (6.8, 14.0, and 17.6 mm). Surgery was performed under general anesthesia via an intraoral approach. The guide was used to establish depth control on the maxillary surface, while a navigation system (Brainlab) was used to confirm the reduction plane in areas that were difficult to visualize directly. The lesion showed a typical gritty texture during reduction.
**Results**: Bone reduction generally followed the preoperative plan with preservation of the infraorbital nerve and orbital floor. Intraoperatively, the contour was considered satisfactory. At 2 weeks, the patient showed improved facial contour with minimal swelling and no external scar. Mild paresthesia of the left upper lip and alar region (approximately 20–30%) was noted. Wound healing was uneventful.
**Conclusion**: The combined use of depth guides and navigation improved intraoperative control in maxillary recontouring. This technique can be performed through an intraoral approach and may help avoid external incisions.

**FA26/470—Technology**
**Standardizing C-Arm Fluoroscopy in Maxillofacial Surgery: A Practical Protocol for Angulation and Radiographic Correlation**
Sruthi Rao ^1^
*^1^* *Rungta College of Dental Sciences and Research, Bhilai, VY Hospital Raipur, Global Star Hospital Raipur, Raipur, India*
**Objectives**: To establish and critically evaluate a standardized intraoperative imaging protocol for C-arm fluoroscopy in maxillofacial surgery through the delineation of reproducible angulation and exposure parameters, with systematic correlation to conventional extraoral radiographic projections.
**Materials and Methods**: A retrospective cohort analysis of 19 maxillofacial surgical scenarios encompassing trauma, benign and malignant pathology, and reconstructive interventions was undertaken. In parallel, cumulative operative experience from over 100 cases employing intraoperative C-arm fluoroscopy informed the development of a structured imaging framework. C-arm angulations and exposure parameters were defined and mapped against established extraoral radiographic views to enable reproducible intraoperative visualization. Outcome measures included intraoperative imaging fidelity, accuracy of osseous reduction, implant positioning, and the impact on real-time surgical decision-making.
**Results**: Intraoperative C-arm fluoroscopy demonstrated consistent acquisition of high-fidelity real-time images, facilitating precise fracture reduction, optimal implant positioning, and enhanced intraoperative assessment across all cases. The proposed protocol enabled reliable replication of conventional radiographic projections, thereby reducing operator dependency and minimizing iterative imaging. Adoption of standardized angulation and exposure parameters translated into improved surgical precision, streamlined workflow, and enhanced efficiency, with pronounced utility in complex craniofacial trauma and reconstructive procedures.
**Conclusion**: The integration of a protocolized approach to C-arm fluoroscopy represents a paradigm shift from ad hoc intraoperative imaging to a structured, reproducible modality in maxillofacial surgery. Standardization of imaging parameters, anchored in radiographic correlation, enhances intraoperative accuracy and decision-making while reducing variability. This framework underscores the potential for broader adoption of C-arm fluoroscopy as a dependable intraoperative imaging adjunct, with implications for optimizing surgical outcomes and advancing operative precision in maxillofacial practice.

**FA26/496—Technology**
**Three-Dimensional Segmentation-Based Imaging of Odontogenic Jaw Cysts: Quantitative Diagnosis and Remodeling Assessment**
Amjad Shhadeh ^1,2^, Shadi Daoud ^1,2^, Adeeb Zoabi ^1,2^, Oded Nahlieli ^1,2^ and Samer Srouji ^1,2^
*^1^* *Department of Oral and Maxillofacial Surgery, Galilee College of Dental Sciences, Galilee Medical Center, Nahariya 2210001, Israel*
*^2^* *The Azrieli Faculty of Medicine, Bar-Ilan University, Safed 1311502, Israel, Nahariya, Israel*
**Objectives**: Odontogenic cystic jaw lesions may show overlapping radiographic features, limiting reliable preoperative differentiation. In addition, response to decompression is usually reported by volumetric reduction alone, without spatial information about where remodeling occurs. This study presents two complementary novel applications of three-dimensional segmentation: whole-boundary attenuation analysis for differential diagnosis and surface-based heat-map analysis for post-decompression remodeling assessment.
**Materials and Methods**: Two retrospective imaging-based analyses were performed. In the diagnostic arm, preoperative cone-beam computed tomography scans of histopathologically confirmed radicular cysts, dentigerous cysts, and odontogenic keratocysts were segmented using a semi-automated three-dimensional workflow. Unlike previous density-based methods based on limited intraluminal regions of interest or selected peripheral points, this method analyzed attenuation values along the entire lesion boundary to reduce sampling bias and better reflect the cyst–bone interface. In the treatment-response arm, 27 patients with intra-osseous cystic jaw lesions treated by decompression underwent pre- and post-treatment cone-beam computed tomography segmentation. Lesion volume, surface area, and surface-distance remodeling metrics were calculated using registered color-coded distance maps.
**Results**: The diagnostic analysis evaluated whether whole-boundary attenuation values differ among cyst types and may assist in identifying odontogenic keratocysts. In the decompression cohort, all lesions demonstrated significant post-treatment reduction. Mean volume reduction was 63.04%, and mean surface area reduction was 41.57%. Heat-map analysis demonstrated inward boundary displacement in all cases, with mean surface deviation of 1.12 ± 0.36 mm and root mean square deviation of 1.39 ± 0.43 mm, suggesting broadly distributed remodeling.
**Conclusion**: Three-dimensional segmentation may transform assessment of odontogenic cystic jaw lesions from descriptive interpretation toward quantitative analysis. Whole-boundary attenuation analysis may improve preoperative cyst characterization, while heat-map analysis provides spatially resolved information on remodeling after decompression, supporting diagnosis, follow-up, surgical planning, and timing of definitive treatment.

**2FA26/1119—Technology**
**Innovation Instrumentation: Reinventing Bone Stability in Craniofacial Surgery**
Venkadasalapathi Narayanasamy ^1^, Senthilkumar Palanichamy ^2^ and Anandapadmanaban S ^3^
*^1^* *KGS Advanced MR & CT Scan, Hannah Joseph Hospital, Maeoris Dental Implants and Esthetic Care Center, Madurai, India*
*^2^* *Madurai General Hospital, Maeoris Dental Implants and Esthetic Care Center, Hannah Joseph Hospital, Dindigul, India*
*^3^* *Theni Government Medical College & Hospital, Hannah Joseph Hospital, Maeoris Dental Implants and Esthetic Care Center, Andipatti, India*
**Objective**: To evaluate the clinical effectiveness of a custom-designed craniofacial reduction instrument that enables controlled three-dimensional manipulation and stabilisation of fracture segments, with the aim of improving anatomical reduction, implant adaptation, and operative efficiency in cranio-maxillofacial trauma surgery.
**Materials and Methods**: Cranio-maxillofacial fractures present significant challenges due to complex anatomy, multidirectional forces, and the functional and aesthetic consequences of non-anatomical reduction. A retrospective review was conducted on trauma cases treated between 2018 and 2025. The novel instrument—comprising a mono-block stainless-steel screw with a perpendicular square bone stop and ergonomic fibre handle—was used to achieve precise three-dimensional reduction and temporary stabilisation during fixation. Two variants (1.5 mm × 6/10 mm for frontal and zygomaticomaxillary fractures; 2.0 mm × 10/12 mm for mandibular fractures) were utilised. Pre-operative CT imaging, intraoperative assessment, and postoperative CT were analysed for alignment quality, fixation stability, implant adaptation, operative duration, and intraoperative safety.
**Results**: The instrument enabled accurate three-dimensional anatomical reduction and maintained stable fragment positioning throughout fixation. Surgeons reported improved access in complex regions, enhanced tactile control, and easier plate adaptation. Operative workflow was more efficient, with a consistent reduction in surgical duration. No adjacent tissue or vital structure injury occurred, supported by the rigid mono-block design and controlled depth provided by the bone stop. Postoperative imaging demonstrated satisfactory three dimensional anatomical restoration and stable fixation across all fracture types.
**Conclusion**: This custom reduction and stabilisation instrument offers a simple, safe, cost-effective, and highly effective method for achieving three-dimensional anatomical reduction and stabilisation in cranio-maxillofacial trauma.

**2FA26/1182—Technology**
**Comparison of Static Guide vs. Robotic-Assisted Screw Placement in Jaw Reconstruction—A Cadaver Study**
Yuk Yu Cheung ^1^, Jienan Ding ^2^ and Jane Jingya Pu ^1^
*^1^* *The University of Hong Kong, Pok Fu Lam, Hong Kong*
*^2^* *Multi-Scale Medical Robotics Center Ltd., Hong Kong*
**Objectives**:
  - To demonstrate the use of robotic system in jaw reconstruction in cadaveric models and validate its accuracy, safety and feasibility.
  - To compare and evaluate the efficacy and accuracy of screw placement in jaw surgeries using static guide vs. robotic system.
**Materials and Methods**: A robotic system EndoMax was developed to perform drilling and screw placement for mandibular reconstruction. Preoperative computed tomography (CT) or cone-beam CT was used to build 3D models of the facial skeleton. Operative plan including an optimal trajectory for screw placement was planned. Reconstruction surgery was performed in the routine manner on cadaveric model, with EndoMax applied for drilling and fixation on the left side and static guide used for the right side, all other procedures were carried out manually. Clinical accuracy, efficacy, safety and feasibility of the system were assessed, and performance of the robotic system was compared with that of conventional approach.
**Results**: 3 screws were placed on each side of the cadaveric model—right side by surgical guide and left side by robotic system. For the static guide group, the maximum off-axis error was 0.965 mm and the mean angle error was 19.39°. For robotic group, the maximum off-axis error was 0.963 mm and the mean angle error was 3.37°. When comparing the total duration of procedure, surgeons used only 18 min in total while EndoMax took 46 min, with 17 min spent on installation of tracker and registration. Surgeons also reported substantial logistics when using surgical guides, including 3D printing, sterilization, verification, all of which can be eliminated in robotic procedures.
**Conclusion**: Robotic system demonstrates feasibility for cadaveric mandibular reconstruction, with position accuracy comparable to conventional method and a markedly higher orientation accuracy. It also offers time-saving potential when multiple guides are required.
Conflict of interest disclosure:
Participation in a company sponsored speaker’s bureau: Multi-Scale Medical Robotics Center Ltd.

**2FA26/1194—Technology**
**Objective Measurement of Pain Using Facial Optomyography: A Novel Approach to Non-Invasive Pain Assessment**
Mai Abouelnaga ^1^, Jaciron Kamalathasan ^2^, Julian Giles ^1^, Charles Nduka ^1^, Viktorija Projkova ^3^, Ivana Kiprijanovska ^3^ and Hristijan Gjoreski ^4^
*^1^* *Queen Victoria Hospital, East Grinstead, UK*
*^2^* *Brighton & Sussex Medical School, Brighton, UK*
*^3^* *Emteq, Brighton, UK*
*^4^* *Cyril & Methodius University in Skopje, Skopje, North Macedonia*
**Objectives**: To evaluate whether facial optomyography can provide a reliable and objective measure of pain intensity by quantifying facial muscle activity and its relationship with subjective and physiological markers.
**Materials and Methods**: A single-centre observational study was conducted on 20 healthy adult volunteers undergoing the submaximal effort tourniquet test. Pain was assessed using the Visual Analogue Scale (VAS), alongside heart rate and blood pressure. Facial optomyographic data were captured using wearable smart glasses and analysed across baseline (first 3 min), pain induction (full duration), and post-stimulation (first 3 min). Extracted features characterised temporal (duration), event-based (frequency), dynamic (expression transitions), and composite (distress index) aspects of facial behaviour. Statistical analysis included Wilcoxon signed-rank tests, Spearman correlation, and multivariate regression.
**Results**: Pain scores were recorded using a 10-point Visual Analogue Scale (VAS) where 0 indicates no pain & 10 indicates worst imaginable pain. VAS scores increased significantly during pain induction (*p* < 0.001) and remained elevated during recovery (*p* = 0.004). Facial optomyographic features demonstrated consistent and discriminative changes, with composite and duration-based measures showing the strongest association with pain. The Distress Index demonstrated a strong correlation with VAS (r = 0.72, *p* < 0.001), outperforming physiological parameters. Frown-related features reflected sustained negative expressions, while increased expression transitions indicated dynamic facial responses to nociceptive stimuli. Heart rate showed only moderate correlation (r = 0.38, *p* = 0.04), and blood pressure demonstrated no significant association. Several facial features remained elevated following stimulus removal.
**Conclusion**: Facial optomyography enables objective, non-invasive quantification of pain and demonstrates superior performance compared to traditional physiological markers. This approach offers a promising tool for real-time pain assessment, with potential

**2FA26/1258—Technology**
**Three-Stage Optical Navigation for Total Temporomandibular Joint Replacement: A Surgical Technique for Accurate Prosthesis Placement and Protection of Critical Structures**
Francisco Jesús Alamillos ^1^, Orlando Estevez ^1^, Inmaculada Centella ^1^ and Alicia Dean ^1^
*^1^* *Department of Oral and Maxillofacial Surgery. University Hospital Reina Sofía. Córdoba. Spain, Córdoba, Spain*
**Objectives**: To present a standardized three-stage optical navigation protocol with stereolithography (STL) verification for total temporomandibular joint (TMJ) replacement, designed for accurate placement of patient-specific prostheses and protection of critical structures (skull base, external auditory canal, facial nerve).
**Materials and Methods**: Structured protocol. Preoperative planning: high-resolution CT with 3D DICOM models and integration of the patient-specific prosthesis STL into the navigation software; marking of critical structures, screw trajectories and bone to be remodelled. Registration: dynamic reference frame fixed to the skull via tripod with reflective optical markers. Intraoperative navigation in three stages: 1st navigation or anatomical navigation, to locate the operative field and critical structures while maintaining safety margins; 2nd navigation or working navigation, to guide and check bone resection and piezoelectric osteotomy as it is performed; and 3rd navigation or checking navigation, to confirm the three-dimensional accuracy of fossa and condylar component placement against the planned STL.
**Results**: The protocol has been implemented in 8 consecutive patients with total TMJ replacements with patient-specific prostheses. Registration and all three navigation stages were reproducible in every case. 1st navigation or anatomical navigation provided real-time control of safety margins to critical structures; 2nd navigation or working navigation guided the planned osteotomy and bone remodelling; 3rd navigation or checking navigation compared final fossa and condylar component position with the planned STL. STL overlay allowed verification of the recipient bed before prosthesis seating. No intraoperative injury to critical structures occurred.
**Conclusion**: This three-stage navigation protocol with STL verification is a feasible, reproducible technique for total TMJ replacement, supporting protection of critical structures and intraoperative verification of prosthesis position against the surgical plan. Quantitative assessment of placement accuracy (planned-versus-achieved deviation) and clinical outcomes is needed to confirm its value.
Conflict of interest disclosure:
Participation in a company sponsored speaker’s bureau: In 2021

**FA26/90—TMJ**
**Effectiveness of Dextrose Prolotherapy Versus Lignocaine in Trigger Point Injection for Myofascial Pain Dysfunction Syndrome: A Randomized Controlled Trial**
Vyakhya Gupta ^1^, Naqoosh Haidry ^1^, Sibgutulah Rashid ^1^, Peeyush Shivhare ^1^ and Yokesh P ^1^
*^1^* *All India Institute of Medical Sciences Patna, Patna, India*
**Objectives**: To evaluate whether trigger point–directed dextrose prolotherapy combined with lignocaine provides superior clinical and functional outcomes compared with lignocaine alone in patients with myofascial pain dysfunction syndrome (MPDS).
**Materials and Methods**: A single-blind randomized controlled trial was conducted on 160 patients diagnosed with MPDS. Participants were allocated into two groups: dextrose (12.5%) with lignocaine (2%) (n = 80) and lignocaine (2%) alone (n = 80). Injections were administered at baseline, 2 weeks, and 4 weeks. Outcomes assessed at baseline, 2, 4, and 6 weeks included pain intensity using Visual Analogue Scale (VAS), pressure pain threshold (PPT) using electronic algometry, pain tolerance, and mandibular range of motion. Statistical analysis was performed using mixed-design repeated measures ANOVA and independent *t*-tests.
**Results**: The dextrose group demonstrated a significant reduction in VAS scores from 6.10 ± 1.44 at baseline to 0.73 ± 1.01 at 6 weeks, compared to minimal change in the control group (6.74 ± 1.73 to 5.98 ± 1.79) (*p* < 0.001). PPT increased significantly in the dextrose group (5.21 ± 1.13 to 8.90 ± 0.95) versus negligible change in controls (*p* < 0.001). Pain tolerance improved markedly (7.54 ± 1.58 to 1.70 ± 1.61), while mandibular movements including mouth opening and excursions showed statistically significant improvement (*p* < 0.001). Large effect sizes were observed across most parameters (η^2^ > 0.14).
**Conclusion**: Dextrose prolotherapy combined with lignocaine provides significantly superior pain relief and functional improvement compared with lignocaine alone in MPDS. It represents an effective regenerative approach for managing myofascial pain.

**FA26/96—TMJ**
**Precision in Complexity: Efficacy of VSP and Mock Surgery in Stock Prosthetic Reconstruction for TMJ Re-Ankylosis**
S. Hemanth Kumar ^1^, Shadab Mohammad ^1^, Vibha Singh ^1^ and Geeta Singh ^1^
*^1^* *King George’s Medical University, Lucknow, India*
**Objectives**: To evaluate the clinical efficacy of integrating VSP with physical mock surgery for stock Total Joint Replacement (TJR) in the management of TMJ re-ankylosis.
Primary outcome: Maximal inter-incisal opening (MIO) & quality of life (QoL).s
Secondary outcome: Biteforce, diet, range of motion, complications.
**Materials and Methods**: A prospective study was conducted on 10 patients (mean age 20.3 years) with unilateral TMJ re-ankylosis (90% Sawhney Type IV). Preoperative workflow involved:
  - VSP: DICOM-to-STL conversion for 3D assessment of ankylotic mass, resection limits, optimal stock prosthesis size and design of cutting guides.
  - Mock Surgery: 3D-printed models were used to perform “dry-run” resections, surface modifications and verifying the virtual plan, ensuring optimal adaptation of the stock prosthesis.
  - Outcome measures included MIO, QoL, bite force, diet, range of motion, complications over a mean follow-up of 50 months.
**Results**: Statistically significant improvements were observed across all primary endpoints (*p* < 0.001) at 50 months follow-up
  - MIO increased from a preoperative mean of 1.83 mm to 37.83 mm.
  - Bite Force improved from 0 kg to a mean of 10.31 kg, facilitating a transition from liquid to regular diet within 12 months, subsequently improving QoL.
  - No complications were recorded.
  - Mock surgery successfully verified digital plans while allowing for intraoperative flexibility.
**Conclusion**: The integration of VSP and mock surgery with stock prostheses provides a highly predictable, cost-effective equivalent to custom prostheses. This dual-validation protocol combines the precision of mock verified VSP with the intraoperative flexibility of stock for optimal prosthesis fit. These benefits translate to significant improvements in patient’s QoL and functional rehabilitation, thus making it a reliable standard in the management of TMJ re-ankylosis.
Conflict of interest disclosure
Support: Study Guide

**FA26/186—TMJ**
**The Kumar-Vasaiya Force Couple Technique: A Biomechanical Paradigm Shift in Management of Acute and Chronic Mandibular Dislocations**
S. Hemanth Kumar ^1^, Krishna Vasaiya ^1^
*^1^* *King George’s Medical University, Lucknow, India*
**Objectives**: Traditional reduction maneuvers for mandibular dislocation often lack ergonomic control, require extensor-dominant push vector and pose risk of clinician injury. We present a clinical series of 52 cases managed with the Kumar-Vasaiya Force Couple Technique, a novel flexor-dominant pull vector approach utilizing a biomechanically superior force couple leading to controlled reduction in both acute and chronic presentations.
**Materials and Methods**: A prospective study conducted on 52 patients presenting with bilateral mandibular dislocation. It included 42-acute and 10-chronic protracted dislocations (ranging from 1–2 months in duration). Chronic cases were administered muscle relaxant orally 30 mins pre-operatively.
**Technique**:
  - Positioning: Clinician stands behind the seated patient with head stabilized at the operator elbow level
  - Hand placement: “Complete mandible is stabilized bilaterally” with thumb on retromolar trigone intraorally and fingers stabilizing extraoral inferior border of body and symphysis region.
  - Reduction maneuver: Reduction is sequential; to reduce one side, a postero-inferior force is applied to the ipsilateral retromolar trigone while a simultaneous antero-superior reciprocal counterforce is applied by the contralateral hand to the mandibular body and symphysis. This creates a controlled force couple leading to rotational moment and reduction of condyle into fossa. The process is repeated on contralateral side.
**Results**: Successful reduction was achieved in 100% of cases, including the two-month protracted dislocation without surgical intervention.
  - Safety: Zero clinician “bite incidents” were recorded due to the extra-occlusal thumb placement.
  - Control: The “pull-force” vector by biceps and back muscles allowed for superior biomechanical control and tactile feedback compared to traditional “push” force by triceps.
**Conclusion**: The Kumar-Vasaiya Force Couple Technique offers a superior biomechanical advantage by integrating flexor-dominant pull vector with rotational force couple system. Its high efficacy in chronic protracted dislocations suggests that this non-invasive counterforce mechanism can overcome significant periarticular fibrosis and muscular contracture, establishing a new gold standard for non-surgical TMJ dislocation management.

**FA26/249—TMJ**
**Comparative Evaluation of Transport Disc Distraction Osteogenesis Versus Alloplastic Total Joint Replacement in Adult Temporomandibular Joint Ankylosis**
Rahul Yadav ^1^
*^1^* *All India Institute of medical sciences, New Delhi, India*
**Objectives**: To compare functional outcomes, complications, and rehabilitative potential of TDDO and TJR in adult TMJ ankylosis.
**Materials and Methods**: Thirty adult patients were treated: TDDO (n = 20) and TJR (n = 10). Parameters assessed included maximum mouth opening (MMO), lateral excursion, protrusion, recurrence, follow-up, and complications
**Results**: Both groups showed significant improvement in MMO, 3 mm to 33.5 mm in TDDO group whereas 2 mm to 27.1 mm in TJR group. TDDO demonstrated superior functional outcomes, with restoration of lateral excursion (3–5 mm) and protrusive movements, along with neo-condyle formation. In contrast, TJR provided adequate mouth opening but minimal translational movements. Complication profiles differed. TJR showed higher incidence of infection and occlusal disturbances. TDDO, being autogenous, had lower infection rates.
TDDO showed favourable outcomes even in selected recurrent cases.
**Conclusion**: Both techniques improve mouth opening. However, TDDO offers superior functional restoration, lower infection risk, and better rehabilitative potential, making it a biologically and functionally feasible option, even in recurrent cases.

**FA26/283—TMJ**
**Extended TMJ Alloplastic Replacement**
Guglielmo Ramieri ^1^ and Giovanni Gerbino ^1^
*^1^* *University of Turin, School of Maxillofacial Surgery, Turin, Italy*
**Objectives**: To present the clinical experience regarding the use of extended alloplastic TMJ reconstruction (eTMJR) in pathologies beyond the joint, involving the mandibular ramus and body or the zygomatic bone.
**Materials and Methods**: A case serie collected over a 20 years period are reconsidered with regard to complications, success rate, and clinical outcome.
**Results**: A series of 18 patients were considered and clinically evaluated. MIO, pain control, QoL and aesthetic satisfaction were improved in all patients.
No failure and a very low rate of surgical complication were encountered.
**Conclusion**:
  - eTMJR is suitable for reconstruction in patents with bony defects that extend beyond the boundaries of traditional TJR
  - eTMJR is a safe and effective management option and could be considered as a first-line treatment option
  - Surgical consistency, absence of donor-site morbidity reduce the overall invasiveness of the procedure
  - Limitations and contraindications: pts with poor local soft tissue quality (irradiation), primary treatment of malignancies

**FA26/327—TMJ**
**Updated Version Outcomes and Complications of Total Temporomandibular Joint Replacement inOmani Patients: Ambidirectional Cohort Study (2015–2026)**
Somaiah Al Maqbali ^1^, Ahmed Al Hashmi ^2^, Siad Al Rashdi ^2^
*^1^* *OMSB (Oman medical Speciality Borad), Muscat, Oman*
*^2^* *Nahdah Hospital, Oman Medical speciality board, Mutrah, Oman*
**Objectives**: Temporomandibular joint disease (TMD), characterized by osseous-fibrous fusion, degenerative disease, trauma, Tumour. TMD is severely impairs mandibular function and induce sever pain which could impact the life style. Alloplastic total joint replacement (TJR) is the gold standard, long-term outcomes and complication remain understudied. This study aimed to retrospectively analyse our 10-year experience with total alloplastic reconstruction of the temporomandibular joint prostheses. Outcomes, complication and life still impact were also evaluated. Methodology: This was a cohort study in which 21 patients who had alloplastic total Temporomandibular Joint prosthesis implanted between 2015 and 2025 were reviewed.
**Materials and Methods**: Outcomes (maximum mouth opening distance between lower anterior incisor teeth to upper anterior incisor teeth), subjective outcomes (VAS, JFLS, PHQ-9), and complications were evaluated.
**Results**: There were 17 females and 4 males with a mean age of 43.2 years (range 20–61 years). The custom made/patients-specific prostheses were used in all patients. Most of patient, degenerative disease was the primary causes followed by trauma induced ankylosis. The results showed that there was a significant improvement in maximum mouth opening distance particularly in patients who had ankylosis. There was improvement in diet consistency and quality of life. Postoperative complications ranged from facial nerve injuries, keloids (n = 1), and occasionally mild pain.
**Conclusion**: TMJ alloplasts provide satisfactory clinical and functional outcomes for patients with end-stage TMJ diseases, evidenced by overall improvement in maximum mouth opening, chewing ability and quality of life and reduction in pain. Previously operated joints and a preoperative diagnosis of ankylosis increase the risk of occurrence of complications post implantation of total alloplastic joints

**FA26/468—TMJ**
**Functional and Airway Outcomes Following TMJ Joint Replacement in Ankylosis: A Prospective Cohort Study**
Santhosh Rao ^1^
*^1^* *All India Institute of Medical Sciences, Raipur, India*
**Objectives**: Temporomandibular joint ankylosis results in severe restriction of mandibular function and compromised upper airway dynamics. This study aimed to evaluate the impact of alloplastic temporomandibular joint replacement on functional outcomes and sleep-related airway parameters in ankylosis patients.
**Materials and Methods**: A prospective cohort study was conducted in 24 patients with temporomandibular joint ankylosis undergoing total joint replacement. Clinical, polysomnographic, and radiological parameters were assessed preoperatively and at 3-, 6-, and 12-month follow-up. Primary outcomes included maximal mouth opening and apnea–hypopnea index. Secondary outcomes included airway volumetric measurements, respiratory disturbance index, oxygen saturation, and oral health–related quality of life scores. Repeated measures analysis of variance was performed, with significance set at *p* < 0.05.
**Results**: Mean maximal mouth opening improved significantly from 2.17 ± 1.98 mm preoperatively to 28.3 ± 3.8 mm at 12 months (*p* < 0.001). Mean apnea–hypopnea index decreased from 19.9 events/hour (range: 0.4–65.8) to 3.5 events/hour (range: 0–12.1) at 6 months (*p* < 0.001), representing a transition from moderate obstructive sleep apnea to near-normal levels and demonstrating a large effect size. Airway volumetric analysis showed significant expansion of the upper airway (*p* < 0.01). Respiratory disturbance index demonstrated a parallel reduction (*p* < 0.01), with improved oxygen saturation and fewer desaturation events. Oral health–related quality of life improved significantly across all domains (*p* < 0.001). No major complications or prosthesis failures were observed during follow-up.
**Conclusion**: Temporomandibular joint replacement in ankylosis patients produces substantial and sustained improvements in mandibular function and airway physiology. Beyond restoring joint mobility, the procedure significantly improves sleep-related breathing parameters with a large treatment effect, highlighting its role as a functionally transformative intervention with systemic benefits.
Conflict of interest disclosure
Receipt of grants/research supports: This study was funded by Indian Council of Medical Research (ICMR) under Extarmural grant academic trials.

**2FA26/1269—TMJ**
**Condylar Hyperplasia of the Mandible: Introducing the Combined Joint–Jaw Control (CJJC) Approach: Case Series**
Sara Al Rasbi ^1,2^ and Ahmed Al Hashmi ^1,2^
*^1^* *Oral and Maxillofacial Surgery Department, Al Nahdha Hospital, Ministry of Health, Oman*
*^2^* *Oman Medical Speciality Board, Muscat, Oman, Muscat, Oman*
**Objectives**: This retrospective case series presents 18 confirmed cases of condylar hyperplasia (CH) managed at Al Nahdha Hospital, Muscat, Oman, and introduces an institutional framework “the Combined Joint–Jaw Control (CJJC) Approach”, a structured, evidence-based protocol integrating condylar activity assessment, morphological evaluation, and individualised surgical planning into a reproducible algorithm.
**Materials and Methods**: All confirmed CH cases at the Department of Oral and Maxillofacial Surgery were reviewed. Diagnosis used a standardized multimodal workup: clinical examination, serial photographs, study models, and imaging. Active growth was defined as ipsilateral-to-contralateral condylar uptake asymmetry ≥ 10%, consistent with established thresholds. Cases were classified using both Obwegeser–Makek and Wolford systems. The CJJC protocol stratifies treatment into three tiers based on condylar activity, facial deformity, and malocclusion severity. Modalities included high or proportional condylectomy (with/without orthognathic surgery), orthognathic surgery alone in inactive cases, and adjunctive genioplasty or osseous contouring. Outcomes assessed facial symmetry, occlusion, complications, and need for secondary surgery.
**Results**: The cohort showed marked female predominance (14:4; F:M 4.75:1). Age at presentation ranged from the late teens to late twenties, reflecting the documented pubertal-onset pattern. Most cases were unilateral (20/23, 87%); 3 (13%) were bilateral. Most unilateral cases were hemimandibular elongation (Obwegeser Type 1 / Wolford Type 1B), with a smaller subset showing hemimandibular hyperplasia. Activity was confirmed by SPECT/CT before planning. All patients showed measurable improvement in facial symmetry, dental midline, and occlusal plane at follow-up. Complications were low, with no permanent neurosensory deficit or condylar avascular necrosis.
**Conclusion**: This is one of the region’s largest confirmed CH cohorts and the first to introduce the CJJC Approach. A structured, SPECT/CT-guided, classification-driven algorithm delivered consistent, reproducible outcomes across all CH presentations, including the rare bilateral subtype—offering a replicable framework integrating joint-level disease arrest with jaw-level skeletal correction.

**FA26/192—Trauma**
**Predicting Complications in Maxillofacial Fractures Using Clinical Variables: A Risk Stratification Model**
Ashwin Pattabhi ^1^
*^1^* *Saveetha Institute of Medical and Technical Sciences, Chennai, India*
**Objectives**: To develop and validate a clinical risk stratification model for predicting postoperative complications in patients undergoing open reduction and internal fixation for maxillofacial fractures.
**Materials and Methods**: A retrospective cohort study was conducted on patients presenting with maxillofacial fractures requiring surgical management. Clinical variables including age, gender, comorbidities, smoking status, fracture site, fracture complexity, time to surgery, presence of infection at presentation, and fixation method were recorded. The primary outcome was the occurrence of postoperative complications, including infection, malocclusion, hardware failure, and neurosensory deficit. Univariate analysis was performed to identify significant predictors, followed by multivariate logistic regression to develop a predictive model. A risk score was derived based on regression coefficients. Model performance was assessed using receiver operating characteristic curve analysis, with calculation of area under the curve. Statistical significance was set at *p* < 0.05.
**Results**: A total of 186 patients were included, with an overall complication rate of 19.3%. Significant predictors of complications included delay in surgery greater than 72 h (odds ratio 3.1; *p* = 0.008), presence of infection at presentation (odds ratio 3.6; *p* = 0.002), and complex fracture patterns (odds ratio 2.8; *p* = 0.01). The final multivariate model demonstrated good predictive ability with an area under the curve of 0.83. A risk stratification score categorized patients into low, moderate, and high-risk groups, with complication rates of 7.1%, 18.9%, and 41.2% respectively.
**Conclusion**: A clinically applicable risk stratification model based on readily available variables can effectively predict postoperative complications in maxillofacial fractures. This model may aid in early identification of high-risk patients, optimize surgical planning, and improve patient outcomes

**FA26/210—Trauma**
**Comparative Analysis of 3D Printed Reduction Splints vs. Conventional Methods in Zygomaticomaxillary Complex Fractures: Assessing Anatomical Reduction and Clinical Outcomes—A Pilot Randomized Controlled Trial**
Poojalekshmi Bhat ^1^ and Santhosh Rao ^1^
*^1^* *All India Institute of Medical Sciences, Raipur, India*
**Objectives**: To compare anatomical reduction accuracy and clinical outcomes between 3D printed reduction splints and conventional reduction methods in ZMC fractures.
**Materials and Methods**: Prospective pilot randomized controlled trial.
**Setting and Sample Size:** This study was conducted at the Oral and Maxillofacial Division of Department of Dentistry, All India Institute of Medical Sciences, Raipur. A total of n = 16 patients with unilateral ZMC fractures were recruited over a period of 14 months and randomized into two groups:
  - VSP-guided 3D printed splint group (n = 8)
  - Conventional reduction group (n = 8)
Participant recruitment, allocation, and follow-up were performed according to CONSORT guidelines.
**Predictor and Outcome Variables:**
  - Predictor variable: Method of reduction (3D printed splint vs. conventional).
  - Primary outcomes: Residual anatomical errors assessed on NCCT, including orbital volumetric discrepancy, anteroposterior, superoinferior and mediolateral translational errors and hemi-bizygomatic width differences.
**Results**: Descriptive statistics were computed for baseline characteristics. Intergroup comparisons were performed using General Linear Model (GLM) adjusting for initial injury magnitude. Kaplan–Meier survival analysis was used for time-dependent outcomes. Statistical significance was set at *p* < 0.05.
A total of 15 patients completed the study (VSP: 7; Conventional: 8). The VSP group demonstrated lower residual errors in orbital volume and translational parameters compared to the conventional group. Operative time for fracture reduction was reduced in the splint group. Improved facial symmetry and higher patient-reported aesthetic satisfaction scores were observed in the VSP group. No significant increase in complications was noted between groups.
**Conclusion**: VSP-guided 3D printed reduction splints provide superior anatomical accuracy and improved clinical outcomes compared to conventional techniques in ZMC fracture management. These findings support their use as a reliable adjunct in maxillofacial trauma surgery, although larger studies are required for validation.
Conflict of interest disclosure
Receipt of grants/research supports: ICMR thesis grant

**FA26/239—Trauma**
**Identifying Violence from Patient Records Using a Language Model**
Annamari Arpalahti ^1,2^, Elisa Hackenberg ^1^, Mikko Vaarala ^1,3^, Teppo Järvinen ^1,2^ and Johanna Uittamo ^1,2^
*^1^* *Helsinki University Hospital, Helsinki, Finland*
*^2^* *University of Helsinki, Helsinki, Finland*
*^3^* *Aalto-Yliopiston Teknillinen Korkeakoulu, Espoo, Finland*
**Objectives**: Facial fractures are characteristic in most assault patients. Depending on the circumstances and expressivity of the patient, their need for additional help may not be noted, with focus on the physical symptoms. Already, artificial intelligence has been implemented for example in the recognition of workplace assault and in predicting the reconviction of offenders. The aim of the current study was to utilize a language model to identify violent aetiologies from patient records.
**Materials and Methods**: FinBERT, a Bidirectional Encoder Representations from Transformers trained for Finnish, was adjusted to identify assault and accident cases from patient records acquired from Helsinki University Central Hospital. With mechanisms such as self-attention and tokenization, BERT asserts different weights for words and recognizes the linguistic surroundings of the word in a sentence, allowing the model to decode the context of the text. In this study, FinBERT was trained with 3968 patient cases that had been pre-evaluated manually to have the aetiology of assault (1103) or accident (2865). For the complete training and validation of the model, a total of 11 261 patient records were utilized, including also other orthopaedic patients.
**Results**: Without preliminary processing for the patient texts, the model acquired an F1-score of 91%. Through preprocessing, where the texts were modified for better structure and emphasis of the relevant information, the F1-score rose to 96%. For future deployment, developing the model with a large dataset of emergency patients is necessary to assess its final functionality.
**Conclusion**: The FinBERT language model has been successful and its implementation is beginning. In the future, the model can be utilized to inform the development of hospital operations. Specifically, it enables evaluation of the recognition of violence as an etiological factor in facial fractures and facilitates analysis of the temporal distribution of patient presentations across different times of day.

**FA26/243—Trauma**
**A Computed Tomography Analysis of Factors Impacting Inferior Rectus Rounding Within Isolated Orbital Floor Fractures**
Ralph Hsiao ^1^, Shawn Dodd ^1^, Alexander Platt ^2^, Emilie Robertson ^1^, Curtis Budden ^1^, Joshua DeSerres ^1^ and Gorman Louie ^1^
*^1^* *University of Alberta, Edmonton, AB, Canada*
*^2^* *University of Calgary, Calgary, AB, Canada*
**Objectives**: Rounding of the inferior rectus muscle (IRM) is a frequently observed radiographic finding in orbital floor fractures. This study investigates the anatomical basis of IRM rounding by examining the relationship between IRM morphology and characteristics of isolated orbital floor fractures seen on computed tomography (CT) imaging at initial presentation.
**Materials and Methods**: A multicentered retrospective cohort study of patients with unilateral, isolated orbital floor fractures were identified on CT imaging between 2008–2025. Demographic and radiographic data were obtained from institutional imaging and clinical databases and included: age, sex, fracture characteristics (morphology, location, mean sagittal length, and defect area), and IRM morphology (rounding, inferior displacement, and location of maximal rounding). Rounding was defined as an IRM height-to-width ratio ≥ 1.0. Inferior displacement of the IRM was categorized using the muscular subscore (MSS).
**Results**: Sixty-seven patients met study criteria. IRM rounding was observed in 59.7% of cases and was associated with sagittal fracture length ≥ 1.5 cm (72.5% vs. 37.0%; *p* = 0.006) and higher MSS grades (MSS 4: 10.0% vs. 3.7%, MSS 3: 15.0% vs. 11.1%, MSS 2: 62.5% vs. 33.3%, MSS 1: 12.5% vs. 51.9%; *p* = 0.008). Post hoc analysis demonstrated a relationship between increased sagittal fracture length and greater IRM displacement (*p* = 0.038). Fracture defect area (*p* = 0.089), morphology (*p* = 0.736), and posterior location (*p* = 0.054) were not associated with IRM rounding. Maximal IRM rounding was most frequently found in the middle thirds of the orbit (67.2%) and the fracture defect (55.7%), and this finding was independent of IRM height-to-width ratio (*p* = 0.602 and *p* = 0.729, respectively).
**Conclusion**: This study identified a relationship between IRM rounding, greater sagittal fracture lengths, and increased inferior displacement of the muscle within isolated orbital floor fractures. These findings suggest that IRM morphology is influenced by the loss of adjacent structural support along the orbital floor rather than overall defect size alone.

**FA26/295—Trauma**
**Broken Faces, Broken Systems: Socioeconomic Inequality in Interpersonal Violence Injuries**
Aleksi Haapanen ^1^, Jussi Furuholm ^2^ and Johanna Snäll ^1^
*^1^* *Helsinki University Hospital, University of Helsinki, Helsinki, Finland*
*^2^* *University of Helsinki, Helsinki, Finland*
**Objectives**: To investigate the association between socioeconomic factors and interpersonal violence–related facial fractures, and to evaluate whether these factors influence injury severity.
**Materials and Methods**: A retrospective case–control study was conducted including adult patients presenting with facial fractures caused by interpersonal violence between 2013 and 2020. For each case, population-based controls were randomly selected. Sociodemographic variables, including education, income, employment status, and country of birth, were analyzed using multivariate logistic regression. The need for surgical treatment was assessed as a proxy for injury severity.
**Results**: A total of 906 cases and 10,050 controls were analyzed. Patients with facial fractures were predominantly male (88.1%) and younger than controls. Socioeconomic disadvantage was strongly associated with fracture risk. Individuals with primary-level education had a significantly higher likelihood of injury (OR 4.8), as did those in the lowest income quartile (OR 2.2) and unemployed individuals (OR 3.9). These associations remained significant after adjustment. In contrast, none of the socioeconomic variables were associated with the need for surgical treatment. Younger age was the only factor linked to increased likelihood of operative management.
**Conclusion**: Interpersonal violence–related facial fractures are strongly linked to socioeconomic inequality, disproportionately affecting individuals with lower education, income, and employment status. These findings indicate that equitable treatment alone is insufficient without addressing upstream social determinants. Focusing on socioeconomic drivers of facial fractures may be essential to reducing their incidence and overall burden.

**FA26/322—Trauma**
**Faces of Trauma: A Five-Year Retrospective Study Review of Craniomaxillofacial Fractures at a Tertiary Trauma Center in Negros Island Region, Philippines**
Bea Marie Garbo ^1^, Patrick John Labra ^1^ and Michael Joseph David ^1^
*^1^* *Corazon Locsin Montelibano Memorial Regional Hospital, Bacolod City, Philippines*
**Objectives**: To determine the prevalence, clinico-epidemiologic profile, fracture patterns, mechanisms of injury, management strategies, and timing of intervention of Craniomaxillofacial (CMF) fractures at a tertiary trauma center over a five-year period.
**Materials and Methods**: The records of 490 patients were reviewed. The data obtained include age, gender, date of injury, addresses, types of fractures, etiology, and management patterns. Descriptive statistics were used to summarize findings, with comparative interpretation against recent local and international literature.
**Results**: The data obtained from the study were consistent with both local and international data however, some differences may be noted. The majority of patients were males (90.61%), with peak incidence in the 21–30-year age group (35.51%). Road traffic incidents were the leading cause (68.98%), followed by interpersonal violence and sports-related injuries. Midface fractures were the most common (61.3%), surpassing mandibular fractures (34.2%), which contrasts with several studies reporting mandibular predominance. Common fracture patterns included zygomaticomaxillary complex (16.1%) and Le Fort fractures (13.6%). Although 67.35% of patients required surgical intervention, only 13.67% underwent definitive surgery, with open reduction and internal fixation (ORIF) performed in 12.05%. A substantial proportion of patients were unable to undergo surgery, refused treatment, or were lost to follow-up.
**Conclusion**: In the last 5 years, CMF fractures in this regional trauma center predominantly affect young adult males and are largely related to road traffic incidents. The predominance of midfacial fractures reflects a high burden of high-energy trauma in the local setting. Despite a high number of patients requiring surgical intervention, definitive management remains limited. This gap is multifactorial, including delayed presentation, financial constraints, follow-up issues, and importantly, limited availability of surgical resources. These findings underscore the need to strengthen trauma systems through improved surgical resource allocation, streamlined referral pathways, and enhanced access to operative care, alongside sustained efforts in road safety prevention.

**FA26/397—Trauma**
**Rethinking Temporomandibular Joint Disc Assessment After Mandibular Condylar Fractures: A Fossa-Centered MRI Framework**
Yunan Zhang ^1^ and Jinlong Chen ^1^
*^1^* *West China Hospital of Stomatology, Sichuan University, Chengdu, China*
**Objectives**: Magnetic resonance imaging assessment of temporomandibular joint disc position after mandibular condylar fractures is usually interpreted relative to the condyle. However, when the fractured condyle is displaced, rotated, or separated from the disc, the condyle itself may no longer serve as a reliable anatomical reference. This study proposed a fossa-centered MRI framework for evaluating disc position after condylar fractures and examined how it differs from conventional condyle-based interpretation.
**Materials and Methods**: Patients with mandibular condylar fractures who underwent temporomandibular joint magnetic resonance imaging were retrospectively reviewed. Disc position was assessed using both conventional condyle-based criteria and a fossa-centered reference system. In the fossa-centered framework, disc position was quantified as continuous spatial parameters, including anterior displacement angle, horizontal displacement distance, and inferior displacement distance. Associations between these parameters, fracture patterns, and associated MRI findings were analyzed.
**Results**: Forty-six patients with 66 fractured condylar joints were included. Conventional condyle-based assessment classified most discs as being in a normal position. In contrast, fossa-centered assessment demonstrated a broad spatial distribution of disc position relative to the glenoid fossa. Disc position parameters differed among fracture types. Greater anterior displacement was associated with retrodiscal tissue signal abnormalities, joint capsule tears, and contact between the ramus stump and the temporomandibular fossa. Cases with disc–condyle decoupling showed greater variability in disc position, indicating that the apparent “normal” disc–condyle relationship may not reflect the true spatial relationship of the disc within the joint.
**Conclusion**: In mandibular condylar fractures, the condyle may be an unstable reference for MRI interpretation of disc position. A fossa-centered framework shifts disc assessment from categorical condyle-based labeling to continuous spatial mapping within the temporomandibular joint. This approach may provide a more anatomically meaningful interpretation of post-traumatic disc displacement and associated joint injuries.

**FA26/415—Trauma**
**Impact of Frontal Bone Fracture on Severity and Outcomes in Panfacial Trauma: A Retrospective Study**
Muhammad Huda Ichsantyarizqa Awang ^1^, Bramasto Purbo Sejati ^1^ and Yosaphat Bayu Rosanto ^1^
*^1^* *Department of Oral and Maxillofacial Surgery, Faculty of Dentistry, Universitas Gadjah Mada, Yogyakarta, Indonesia*
**Objectives**: Panfacial fractures represent one of the most complex injuries in craniofacial trauma, involving at least 3 out of 4 facial region. Frontal bone fractures are commonly associated with these injuries, yet their specific impact on severity and clinical outcomes remains poorly defined, particularly in high-energy trauma settings prevalent in developing countries. This study aim to analyze the association between frontal bone involvement and fracture severity, length of hospital stay, and concomitant injuries in panfacial fracture patients.
**Materials and Methods**: A retrospective study of 42 panfacial fracture patients at Temanggung Regional Hospital was conducted between March 2022 to April 2026. Variables including demographics, etiology, fracture pattern (FULM/FUL/FLM/FUM/ULM), frontal bone involvement, FISS score, head injury severity, concomitant injuries, and length of stay were recorded. Chi-square analysis were used to evaluate associations between variables, with statistical significance set at *p* < 0.05.
**Results**: Males predominated (69.05%), with most patients aged 10–20 years (45.24%). Traffic accidents were the leading cause (92.86%). Frontal bone involvement was present in 69.05% of cases; FULM was the most common pattern (42.86%). A significant association was found between etiology and fracture pattern (*p* = 0.000), with traffic accidents strongly associated with the FULM pattern, and between gender and fracture pattern (*p* = 0.038). Frontal bone involvement was significantly associated with FISS severity (*p* = 0.015): 51.7% of patients with frontal bone fractures were FISS Severe versus only 7.7% without frontal bone involvement. No significant associations were found with length of stay (*p* = 0.794), head injury severity (*p* = 0.151), or concomitant injuries (*p* = 0.773).
**Conclusion**: Frontal bone involvement emerges as a meaningful predictor of injury severity in panfacial trauma, offering clinicians a practical guide for risk stratification and perioperative decision-making, even when neurological compromise is not immediately apparent.

**FA26/438—Trauma**
**Safety and Efficacy of Endoscopic Subcondylar Repair Technique in Patients with Multiple Mandibular Fractures**
Weitao Wang ^1^
*^1^* *University of Rochester, Rochester, New York, NY, USA*
**Objectives**: Is the use of endoscopic subcondylar repair technique safe in mandibular multifracture patients with reliable outcomes?
**Materials and Methods**: Single surgeon retrospective consecutive cohort case series of multiple mandible fractures with condylar component who underwent ORIF compared to isolated subcondylar fractures. Prior endoscopic experience before first bilateral mandible case 1 endoscopic isolated case. All patients age > 18 at the time of injury were included. Patients with isolated subcondylar repair and condylar head fractures with open repair were excluded. A total of 12 consecutive patients were identified. 12 consecutive series of isolated subcondylar fractures were included for comparison.
**Results**: 24 patients (15 males and 9 females) were identified and included; 12 in the isolated subcondylar group and 12 in the multiple fractures group. Mechanism of injury were assault (n = 9), fall (n = 8), MVC (n = 5), unknown/other (n = 2). Postoperative reduction was assessed with CT maxillofacial scan. When patients made themselves available for follow-up, occlusion, maximal incisor opening, lip numbness, pain, scar assessment, hardware complications, and facial nerve function were assessed.
Patient outcomes: Premorbid occlusion was achieved in 12/12 in the multiple fractures group, and 10/12 in the isolated subcondylar group. No patients required conversion to open repair in the multiple fractures group, whereas 2 patients in the isolated subcondylar group required conversion (*p* = 0.48). Transient facial paresis was appreciated in 2 patients in the isolated subcondyle group. 1/24 patients required steroid injection to trocar scar, cosmesis was otherwise excellent for all patients. No hardware removal was necessary for any patients in either group. Follow-up duration ranged from 15 to 1647 days.
**Conclusion**: Endoscopic subcondylar repair technique is safe and reliable in multiple mandible fracture settings and should be considered based on surgeon preference and experience. Further studies are needed to assess long-term functional outcomes of this technique with reliable follow-up.

**FA26/467—Trauma**
**Medial-First Fixation in Condylar Neck Fractures: Radiologic Evaluation of Miniplate Angulation and Interplate Distance in 21 Patients**
Nabila Hanifiah Arifin ^1^, Bramasto Purbo Sejati ^1^, Robby Ramadhonie ^2^
*^1^* *Departmen of Oral and Maxillofacial Surgery, Faculty of Dentistry, Universitas Gadjah Mada, Yogyakarta, Indonesia*
*^2^* *Departmen of Oral and Maxillofacial Surgery, Margono Soekarjo General Hospital, Purwokerto, Indonesia*
**Objectives**: Neck fracture of the mandibular condyle is a challenge in management due to limited access and the need for optimal fixation stability. The ORIF technique with two miniplates is increasingly being used, but the ideal configuration—especially regarding the angle and distance between the plates—is not yet a consensus. The medial-first fixation approach enables the initial repositioning of the medial fragment as a reference, which may impact the final configuration of the plate and the quality of the reduction. This study aimed to radiologically evaluate the variation in angle and distance between two miniplates in the medial-first fixation technique.
**Materials and Methods**: A retrospective observational study was conducted on 21 patients with a mandibular condyle neck fracture who underwent ORIF via a retromandibular approach. The data analyzed included preoperative CT-scans, intraoperative documentation, and postoperative panoramic radiography. The variables measured included the angle between the miniplates, the distance between the plates, and the radiological parameters of the reduction results (fragment alignment, discrepancy, and condyle position). The analysis was performed to evaluate the plate configuration pattern and its relationship with the reduction quality.
**Results**: The angle variation between the miniplates was 30–90° with a mean of 45.24° and the majority of the distance between plates was less than 1 cm (85.71%). Configurations with greater smaller angles and narrower distances between plates indicate a tendency for more optimal reduction results. Most cases show good alignment with adequate condyle position on postoperative radiological evaluation.
**Conclusion**: The medial-first fixation approach provides flexibility in the configuration of two miniplates with specific angle and distance variations. Radiological evaluation shows that such configuration may be related to the quality of fracture reduction. These findings can be the basis for consideration in determining plate positioning to achieve clinically optimal fixation results.

**2FA26/1050—Trauma**
**3D Assisted Volumetric Assessment of Orbits in Patients with Unilateral Blow Out Orbital Fracture**
Khairul Bariah Chi Adam ^1^, Ainnisya Syafiqha Zulfahmi ^2^ and Auni Atiqah Husmad ^1^
*^1^* *International Islamic University Malaysia, Kuantan, Malaysia*
*^2^* *International Islamic University Malaysia, Kuantan, Malaysia*
**Introduction**: Orbital fractures frequently alter orbital volume, leading to complications such as enophthalmos and diplopia. Therefore, accurate three-dimensional (3D) quantification is essential for reliable diagnosis, precise surgical planning, and objective postoperative evaluation.
**Objectives**: This study aimed to; (1) compare orbital volume between the preoperative orbital blow out fracture volume and the unaffected contralateral side, and (2) to compare postoperative repaired orbital volume with the unaffected side.
**Materials and Methods**: This retrospective study included 24 Malaysian patients aged between 16–69 years old with unilateral orbital fractures and available three-dimensional imaging data. Computed tomography (CT) data from Radiology Unit SASMEC@IIUM were analyzed using 3D imaging software, which is Aliza Medical Imaging & DICOM Viewer to segment and calculate orbital volumes of the pre-operative fractured orbit, post-operative fractured orbit, and contralateral unaffected orbit. Paired statistical analysis was performed to evaluate differences between the orbits.
**Results**: The pre-operative fractured orbits demonstrated a significantly higher orbital volume compared to the unaffected orbits (*p* < 0.05). Post-operative orbital volumes showed no significant difference when compared with the unaffected side (*p* > 0.05).
**Conclusion**: Three-dimensional CT-based orbital volumetric analysis is a reliable method for evaluating unilateral orbital fractures. The fractured orbit demonstrated a larger pre-operative orbital volume compared to the unaffected orbit, reflecting increased orbital cavity space following trauma. Post-operative orbital volumes comparable to the unaffected orbit suggest effective surgical restoration of orbital anatomy. These findings also provide baseline orbital volume data for the Malaysian population.

**2FA26/1088—Trauma**
**A Comparative Study of Complications of Subciliary Incision Versus Infraorbital Incision for Access to the Zygomatic Bone and Orbital Floor Fractures**
Junaid Nadeem ^1^, Maria Nizam ^2^, Zeenia Arbab ^2^, Umer Ullah ^3^, Hansa Afridi ^2^, Musa Zeb ^2^, Hubaib Afridi ^2^, Faryal Gul ^2^ and Muhammad Huzaifa ^2^
*^1^* *Peshawar Dental College, Peshawar, Pakistan*
*^2^* *Sardar Begum Dental College, Peshawar, Pakistan*
*^3^* *Rehman College of Dentistry, Peshawar, Pakistan*
**Objective:** The purpose of this study was to compare the subciliary approach and the infraorbital approach to access the zygomatic bone and the orbital floor to see subsequent complications such as scar formation, ectropion and entropion development.
**Materials and Methods:** In this retrospective cohort study, a sample of 50 patients was selected, of which 25 patients were placed in each group. The data was collected in local hospitals in Peshawar from September 2018 to June 2022. Follow-up was done at 1, 3 and 6 months interval. Three-dimensional computed tomography scans were done for each patient during follow-up period. Clinical photographs were taken to assess scarring, ectropion and entropion. Evaluation was done using Ordinal and Binary Likert’s scale.
**Results:** Mean age group presenting with zygomatic bone or orbital floor fractures was 26–29 years (*p* = 0.25) with a higher incidence of male patients (*p* = 0.63) and unilateral fractures (*p* = 0.77). Grade 3 scar formation was seen in 88% patients who underwent infraorbital incision whereas only 56% patients showed grade 1 or 2 scar formation in subciliary incision (*p* < 0.001, 95% CI for risk difference: 60% to 92%). Similarly, the rate of entropion was high i.e., 21% in patients of infraorbital incision and no patients of subciliary incision showed entropion formation (*p* < 0.001). Ectropion was seen in 64% patients of infraorbital incision as compared to 24% patients of subciliary incision (*p* = 0.010; 95% CI for risk difference; 11% to 65%).
**Conclusions:** Subciliary approach when compared to the infraorbital approach may offer superior esthetics and functional outcomes and show decreased chances of complications.

**2FA26/1190—Trauma**
**The Traumatic Triad in Pediatric Maxillofacial Trauma: The Interplay of Age, Mechanism, and Craniofacial Anatomy in Fracture Patterns and Management Strategies**
Hamzah Sukma Anggoro ^1^, Pingky Krisna Arindra ^1^ and Bramasto Purbo Sejati ^1^
*^1^* *Universitas Gadjah Mada, Yogyakarta, Indonesia*
**Objectives**: Pediatric maxillofacial trauma exhibits unique injury characteristics influenced by age-related craniofacial development, behavioral risk factors, and injury-specific biomechanical stress patterns. Although increasing evidence suggests an interrelationship among these factors, their integrated role in determining fracture patterns and management strategies remains poorly understood. This study aimed to evaluate the interplay of age, injury mechanism, and craniofacial anatomy in pediatric maxillofacial trauma and to propose a clinically applicable Traumatic Triad Framework.
**Materials and Methods**: A retrospective observational study was conducted on pediatric patients (<18 years) with maxillofacial fractures between January 2022 and December 2025. Using a total sampling approach, all eligible patients were categorized into preschool (0–6 years), school-age (7–12 years), and adolescent (13–18 years) groups. Demographic characteristics, injury mechanisms, fracture patterns, and treatment modalities were analyzed using the Chi-square test, with *p* < 0.05 considered statistically significant.
**Results**: A total of 303 pediatric patients were included, with a mean age of 14.1 ± 4.0 years; 77.9% were adolescents and 68.3% were male. RTA (83.2%) were the predominant injury mechanism, followed by falls (13.9%). Dentoalveolar (36.6%) and mandibular fractures (14.2%) were the most common injury patterns. Open reduction was the most frequent treatment modality (57.6%). Age group was significantly associated with injury mechanism (*p* < 0.001), fracture pattern (*p* = 0.007), and treatment modality (*p* < 0.001), while fracture pattern varied significantly according to injury mechanism (*p* < 0.001). Adolescents predominantly sustained traffic-related complex fractures requiring operative intervention, whereas younger children more commonly sustained fall-related dentoalveolar injuries managed conservatively.
**Conclusion**: The findings support the Traumatic Triad Framework, in which age, injury mechanism, and craniofacial anatomy interact to shape fracture patterns and management strategies in pediatric maxillofacial trauma.

**2FA26/1197—Trauma**
**Can Condylar Displacement Predict Associated Facial Fractures? A Two-Center Retrospective Study**
Hariet Maliki ^1^, Bramasto Purbo Sejati ^1^, Cahya Hasan ^1^
*^1^* *University of Gadjah Mada, Yogyakarta, Indonesia*
**Objectives**: Mandibular condyle fractures are among the most common maxillofacial injuries. The direction of condylar displacement reflects the vector and magnitude of traumatic forces acting on the mandible. However, whether specific displacement patterns are associated with concomitant maxillofacial fractures remains poorly understood. To evaluate the relationship between condylar displacement patterns and associated maxillofacial fractures and to compare displacement characteristics between isolated and concomitant condylar injuries.
**Materials and Methods**: A retrospective radiographic study was conducted at two tertiary referral centers in Indonesia from 2020 to 2025. Computed tomography and panoramic radiographs of patients with mandibular condyle fractures were reviewed. Displacement direction (anterior, posterior, medial, lateral, and superior), fracture level (head, neck, and subcondylar), and associated fracture sites were recorded. Patients were categorized into isolated condylar fractures and concomitant injuries involving additional maxillofacial fractures. Associations between displacement patterns and fracture characteristics were analyzed using Chi-square and Fisher’s exact tests.
**Results**: Seventy-eight patients with 112 condylar fractures were included. Medial displacement was the most frequent pattern (43%). Concomitant injuries demonstrated significantly higher frequencies of medial and posterior displacement compared with isolated condylar fractures (*p* < 0.05). Parasymphysis fractures were the most common associated injury (48%), followed by zygomatic complex fractures (26%). In contrast, isolated condylar fractures predominantly exhibited mild anteromedial deviation without substantial vertical displacement. Specific displacement directions showed significant associations with particular fracture sites, suggesting distinct trauma vectors and load distribution patterns
**Conclusion**: Condylar displacement patterns differ significantly between isolated and concomitant maxillofacial injuries. Medial and posterior displacement are associated with the presence of additional facial fractures and may serve as radiographic indicators of underlying injury mechanisms. Recognition of these patterns may facilitate early detection of associated fractures and improve diagnostic assessment and treatment planning in maxillofacial trauma.

**2FA26/1277—Trauma**
**Multicenter Validation of the IMPACT: A New Instrument to Measure Quality-of-Life in Facial Trauma**
Aaron Zebolsky ^1^, Travis Clarke ^1^, David Weatherford ^1^, Alice Su ^2^, Alexander Marston ^2^, Travis Tollefson ^2^ and Anas Eid ^1^
*^1^* *University of Tennessee Health Science Center, Memphis, TN, USA*
*^2^* *University of California Davis, Sacramento, CA, USA*
**Objectives**: To evaluate convergent validity of the Integrated Modular Patient-Reported Outcome Assessment for Craniomaxillofacial Trauma (IMPACT) against the 15-Dimension (15D) health-related quality of life (QOL) instrument in a cross-sectional multicenter cohort of facial trauma patients.
**Materials and Methods**: Consecutive patients with craniomaxillofacial trauma at two academic centers completed the IMPACT and the 15D at during a routine follow-up appointment within one year of trauma. IMPACT composite and module scores (0 to 100; higher indicates worse disease-specific QOL) and 15D scores (0 to 1; higher indicates better generic health-related QOL) were calculated using published algorithms. Convergent validity was assessed with Pearson and Spearman correlation coefficients. Multivariable linear regression tested the independent association between IMPACT and 15D scores, adjusting for age, sex, and study site.
**Results**: Of 190 enrolled patients, 151 (79.5%) had scorable IMPACT and 15D data. The mean IMPACT composite score was 37.0 ± 19.9 and the mean 15D score was 0.80 ± 0.20. IMPACT composite correlated inversely with 15D (Spearman rho = −0.57, *p* < 0.001; Pearson r = −0.60, *p* < 0.001). Module scores also correlated with 15D: general module (rho = −0.57), jaw module (rho = −0.54), nasal module (rho = −0.39), and orbit module (rho = −0.41) (all *p* ≤ 0.005). In multivariable analysis adjusting for age, sex, and site, each 10-point increase in IMPACT composite was associated with a 0.050 decrease in 15D score (95% confidence interval −0.065 to −0.034; *p* < 0.001). Site-adjusted analysis of the full cohort confirmed the association (*p* < 0.001).
**Conclusion**: IMPACT demonstrates strong convergent validity with the established 15D instrument across composite and module-specific scores, supporting its use as a craniomaxillofacial trauma-specific instrument to measure QOL.
Conflict of interest disclosure:
Receipt of grants/research supports: AO CMF Resident Research Grant 2025

**FA26/196—Other**
**Evaluation of Neuropsychological Alterations in Isolated Maxillofacial Trauma**
Melvinder Tyndall ^1^, Vigneswaran Veeramuthu ^2^, Krishana Ananda Gopalakrishnan ^3^, Shubash Ganapathy ^4^ and Firdaus Hariri ^5^
*^1^* *Ministry of Health (MOH), Sibu, Malaysia*
*^2^* *Thompson Hospital Kota Damansara, Petaling Jaya, Malaysia*
*^3^* *Gleneagles Hospital Medini Johor, Iskandar Puteri, Malaysia*
*^4^* *National Institute of Health, Setia Alam, Malaysia*
*^5^* *Universiti Malaya, Kuala Lumpur, Malaysia*
**Introduction:** Maxillofacial fractures have resulted in white matter microstructural changes indicating an underlying traumatic brain injury (TBI). Neuropsychological assessments can be an alternative screening tool for traumatic axonal injuries (TAI).
**Objectives:** To evaluate the sensitivity of neuropsychological tests as a tool in detecting subtle neurocognitive changes following isolated maxillofacial trauma and to establish the relationship between pattern and severity of maxillofacial trauma and the neurocognitive alterations.
**Materials and Methods:** A total of 60 subjects who fulfilled the inclusion and exclusion criteria were enrolled into this prospective study from Universiti Malaya. There were 45 motor-vehicle accident (MVA) subjects with maxillofacial injuries and 15 healthy controls. The MVA subjects were assessed within 24 h and 6 months post-trauma. Four neuropsychological assessment tools were utilised sequentially: Abbreviated Mental Test (AMT), Montreal Cognitive Assessment (MoCA), Mini-Cog with Screening Neuropsychological Assessment Battery (SNAB) as the benchmark test. All tests were conducted by a single examiner trained and calibrated by a neuropsychologist. The maxillofacial injuries were recorded according to maxillofacial trauma proforma and Facial Injury Severity Score (FISS).
**Results:** 91.8% of MVA subjects were motorcycle related. MVA group showed significant neuropsychological impairment compared to healthy control at baseline and follow-up. Attention (*p* < 0.001), memory (*p* = 0.004) and visuospatial (*p* = 0.005) domains were impaired. MoCA was more sensitive (79.2%) followed by Mini-Cog (58.3%) and AMT (8.3%). However, AMT showed higher specificity (95.2%) than MoCA (76.2%) and Mini-Cog (76.2%). MoCA had the highest Pearson Correlation value (*r* = 0.617, *p* < 0.001). Hence, any maxillofacial injury associated with neurocognitive alterations were suggestive of TAI.
**Conclusion:** MoCA is a concise yet simple test to be administered in an emergency setting and during follow-up to screen for neuropsychological alterations in patients with maxillofacial injuries. Maxillofacial injury is a predictor for TAI therefore, neurocognitive screening should be done for all cases with maxillofacial injuries.

**FA26/255—Other**
**Clinical Outcomes of Greater Palatine Pedicled Flap in Surgical Management of Trismus in Oral Submucous Fibrosis**
Arunkumar Shadamarshan R ^1^
*^1^* *Vajra Corps Dental Unit, Jalandhar, India*
**Objectives**: To evaluate the clinical usefulness of the greater palatine pedicled flap in improving trismus in patients with oral submucous fibrosis.
**Materials and Methods**: This retrospective study included diagnosed cases of oral submucous fibrosis treated with fibrous band release and defect coverage in the retromolar trigone region using a greater palatine pedicled flap between 2008 and 2019. Patients were assessed for preoperative and postoperative mouth opening, donor site healing, and procedure-related complications.
**Results**: 29 patients were included, comprising 13 stage III and 16 stage IV cases. Mean follow-up was 29.6 months in stage III cases and 32.4 months in stage IV cases. Mouth opening improved significantly from 20.0 mm to 32.92 mm in stage III patients and from 6.81 mm to 26.31 mm in stage IV patients. The mean difference in the pre-operative mouth opening and Post-operative mouth opening was 12.92 mm in Stage III and 19.5 mm in Stage IV. This mean difference of 6.57 mm in the post-operative mouth opening measurements among the two groups was also statistically significant. Donor site healing occurred within a mean of 5 weeks, and flap necrosis was observed in 02 stage IV cases.
**Conclusion**: The greater palatine pedicled flap is a reliable reconstructive option for surgical release of trismus in advanced oral submucous fibrosis.

**FA26/276—Other**
**Single-Center Experience in the Management of Medication-Related Osteonecrosis of the Jaw**
Reema Mahmoud ^1^ and Amir Shuster ^1^
*^1^* *Tel Aviv Sourasky Medical Center, Tel Aviv, Israel*
**Objectives**: Medication-related osteonecrosis of the jaw (MRONJ) remains a challenging complication of antiresorptive therapy. While surgical management has gained increasing support, the influence of concomitant systemic medications on healing remains unclear. This study aimed to evaluate clinical outcomes in MRONJ, focusing on the impact of surgical intervention and commonly prescribed systemic drugs.
**Materials and Methods**: A retrospective cohort study included 222 patients with stage I-III MRONJ treated at a tertiary referral center between 2009 and 2025. Demographic, clinical, pharmacological, and treatment-related variables were analyzed. Outcomes were defined as lesion improvement and complete resolution (full mucosal coverage without infection) at final follow-up. Multivariable logistic regression assessed factors associated with healing.
**Results**: Overall, 84.7% of lesions improved and 69.4% achieved complete resolution. Surgical management was the strongest determinant of favorable outcome, accounting for 94.2% of resolved cases (*p* < 0.001), and increased the odds of resolution more than eightfold (OR = 8.63). Marginal mandibulectomy and partial maxillectomy were particularly associated with definitive healing. Resolution rates were higher in osteoporosis-related compared to oncology-related MRONJ (80.7% vs. 62.6%, *p* = 0.005). Concomitant use of proton pump inhibitors (OR = 2.11, *p* = 0.01), selective serotonin reuptake inhibitors (OR = 2.09, *p* = 0.05), and systemic corticosteroids (OR = 2.10, *p* = 0.02) was independently associated with persistent disease.
**Conclusion**: Surgical intervention is the principal determinant of MRONJ resolution, supporting a proactive, surgery-oriented approach. Common systemic medications, particularly PPIs, SSRIs, and corticosteroids, significantly impair healing and should be carefully considered during treatment planning.
E-POSTER

**FA26/25—Aesthetics**
**A Three-Dimensional Model Study on Beauty Standards for Cheekbones in Both Sexes**
Emile Huybrecht ^1^
*^1^* *Uz Brussel, Vrije Universiteit Brussel, Jette, Belgium*
**Objectives**: The aim of this study was to determine whether a ‘beautiful’ malar morphology is gender-specific and whether volume/projection play a role.
**Materials and Methods**: Models of the faces of one female and one male subject, selected in a two-stage process, were three dimensionally printed. Using Geomagic Sculpt, the malar bones were transposed from male to female and vice versa. The assessors were asked to identify the male and female faces. Next, volume and projection were added/reduced to create a series of four models for each sex. The assessors were asked to evaluate these for facial harmony in the malar area on a visual analogue scale (VAS).
**Results**: The original female and male models were wrongly classified by only 3% and 2% of the assessors, respectively. Regarding the transposed models, 96% of the female faces with male malar contours were classified as masculine, while 91% of the male faces with female malar contours were classified as feminine. In the evaluation of volume/projection, the original malar prominences scored significantly higher than the altered ones.
**Conclusion**: A preference for more and less volume and projection in the female and male models was observed respectively. Alteration of volume and projection had a less noticeable impact than in the female models.

**FA26/294—Aesthetics**
**Face Tapping and Face Yoga Anti-Ageing Practices to Delay Facial Ageing and Promote Well-Being Among Surgeons and Their Patients**
Dr Madhu Nagesh ^1^
*^1^* *Oceanside Private Practice, adelaide, Australia*
**Objectives**: This paper aims to highlight the impact of lifestyle-induced facial tension on ageing and wellbeing; introduce the principles of facial harmony and relaxed expression as determinants of perceived attractiveness and trust; educate participants on simple, evidence-informed practices such as facial harmony exercises, face tapping, and somatic breathing; and empower clinicians to integrate these techniques into self-care and patient education.
**Materials and Methods**: A structured instructional session was conducted, combining demonstration and guided participation. Techniques included targeted facial exercises addressing key muscles of expression and posture, face tapping methods to release muscular tension and enhance circulation, and somatic breathing practices to promote parasympathetic activation and neuromuscular relaxation. Observational insights were drawn from participants across prior workshops led by the presenter, a Maxillofacial Surgeon and certified Yoga practitioner.
**Results**: Participants reported reduced facial muscle tension, improved relaxation, and enhanced emotional wellbeing. Many also observed visible improvements in facial tone and early ageing signs. High compliance was attributed to the simplicity, minimal time requirement, and ease of integrating these practices into daily routines.
**Conclusion**: Chronic facial tension is a significant yet under-recognized contributor to premature ageing and diminished wellbeing. Simple, non-invasive interventions can restore facial relaxation, improve appearance, and support emotional balance. These scalable techniques offer clinicians a practical, preventive approach to holistic facial health.

**FA26/315—Aesthetics**
**Soft Tissue Defects: The Efficacy of Hydrocolloid Dressing for Tissue Regeneration**
Siti Khodijah Ahmad ^1^ and Melvinder Tyndall ^1^
*^1^* *Hospital Sibu, Sarawak, Malaysia*
**Objectives**: To demonstrates the efficacy of hydrocolloid dressing for tissue regeneration in cases of tissue loss in the facial region.
To emphasize the importance of wound bed preparation in facial tissue loss prior to reconstruction to achieve satisfactory aesthetic outcome.
**Materials and Methods**: This case series comprised of facial soft tissue loss secondary to either soft tissue injury or infection in the facial region. The age of these patients ranges from 24 to 70 years-old and some presenting with comorbidities. All 3 patients were treated with either hydrocolloid dressing and/or hydrocolloid gel. In the case of facial soft tissue loss, the management includes simple wound toileting followed by the application of hydrocolloid dressing which was replaced twice in the span of one week for the tissue bulk and facial contour to be totally restored. In cases of facial infection, the initial management to prevent further spread by incision and drainage is still the mainstay of treatment. Once the infection has resolved, hydrocolloid dressings were applied to prepare a healthy wound bed prior to reconstruction to achieve an aesthetic facial outcome.
**Results**: Utilization of hydrocolloid dressings in facial soft tissue injuries reduces the need for extensive soft tissue surgical procedures. It aids in gross facial tissue loss where the anatomical features were able to be restored by primarily.
**Conclusion**: Tissue loss in the facial can be challenging to manage due to its aesthetic importance and functional demands. The knowledge of various wound dressing materials and its indications are important to effectively manage such wounds and restore the anatomy, hence the need for a well-planned wound care and subsequent reconstruction if required. The use of hydrocolloid dressing in the early stage of wound management can promote rejuvenation and accelerate epithelialization which can be beneficial to optimize healing outcomes and enhanced recovery.

**FA26/432—Aesthetics**
**Postoperative Patient Reported Outcomes After Rhinoplasty: A Comparative Study Using NOSE, SCHNOS, FROI-17, and ROE at 6 Months**
Kuldeep Singh ^1^
*^1^* *All India Institute of Medical Sciences, Patna, India*
**Objectives**: To evaluate postoperative patient-reported outcome measures following rhinoplasty using validated scoring systems, including the Nasal Obstruction Symptom Evaluation (NOSE), Standardized Cosmesis and Health Nasal Outcomes Survey (SCHNOS), Functional Rhinoplasty Outcome Inventory-17 (FROI-17), and Rhinoplasty Outcome Evaluation (ROE), at 6 months follow-up, and to compare their performance and inter-relationship.
**Materials and Methods**: This is a cross-sectional study of adult patients at 6 months following rhinoplasty performed for functional and/or aesthetic indications. After detailed clinical history, examination, and documentation of surgical details, patients were assessed using four validated patient-reported outcome measures (PROMs): NOSE, SCHNOS, FROI-17, and ROE. Patients with incomplete questionnaires and those with acute facial trauma were excluded. Statistical analysis will be performed using descriptive statistics such as mean (SD), median (IQR), and frequency (%). Correlation between variables will be evaluated using Spearman’s correlation coefficient, with a *p*-value of <0.05 considered statistically significant.
**Results**: The study is ongoing, and results are yet to be fully compiled. It is anticipated that functional PROMs (NOSE, SCHNOS, and FROI-17) will demonstrate moderate to strong correlation with each other, while ROE may show weaker correlation with functional scales, reflecting differences in the constructs measured. Some degree of discordance between functional outcomes and patient satisfaction is also expected.
**Conclusion**: This study provides a comparative evaluation of multiple scoring systems used in rhinoplasty and aims to identify the most appropriate tools for outcome assessment. It is expected to demonstrate that different instruments capture distinct aspects of postoperative outcomes, suggesting that no single scoring system is sufficient. A multi-PROM approach may therefore be necessary for comprehensive evaluation, and the findings may help guide the selection of outcome measures in both clinical practice and research.

**FA26/437—Aesthetics**
**The Smart Face: AI’s Aesthetic Takeover**
Hannah Arnantha ^1^, Hitesh Bansal ^2^
*^1^* *St George’s, Epsom and St Helier Hospital Group, London, UK*
*^2^* *London North West University Healthcare NHS Trust, London, UK*
**Objectives**: This literature review aims to evaluate the current applications, effectiveness, and limitations of artificial intelligence (AI) in non-surgical facial aesthetics. It explores how AI technologies are transforming assessment, treatment planning, and outcome prediction in aesthetic medicine.
**Materials and Methods**: A comprehensive search of peer-reviewed articles published between 2015 and 2025 was conducted across databases including PubMed, Scopus, and Web of Science. Keywords included “artificial intelligence,” “machine learning,” “facial aesthetics,” “non-surgical,” “dermal fillers,” and “botulinum toxin.” Studies were included if they addressed AI-driven tools in facial analysis, patient consultation, treatment planning, or outcome evaluation. Both clinical and technical studies were reviewed. Data were extracted and synthesized qualitatively to identify common themes, innovations, and gaps in the literature.
**Results**: The review identified a growing body of evidence supporting the integration of AI in non-surgical facial aesthetics. AI-driven facial recognition and analysis systems demonstrated high accuracy in assessing facial symmetry, proportions, and aging patterns. Machine learning models have been used to assist clinicians in personalized treatment planning and to predict aesthetic outcomes. Additionally, AI-powered simulation tools improved patient communication and expectation management. However, limitations were noted, including variability in dataset quality, lack of standardization, and concerns regarding bias and generalizability across diverse populations. Ethical considerations, particularly around data privacy and informed consent, were also highlighted.
**Conclusion**: AI represents a promising adjunct in non-surgical facial aesthetics, offering enhanced precision, personalization, and efficiency. Despite its potential, further research is needed to validate clinical effectiveness, address ethical challenges, and establish standardized frameworks for implementation. Careful integration of AI alongside clinician expertise will be essential to ensure safe and equitable adoption in aesthetic practice.

**FA26/483—Aesthetics**
**Preoccupied with the Mirror: A Systematic Review on Body Dysmorphic Disorder in Cosmetic and Aesthetic Surgery**
Kuldeep Singh ^1^, Ankita Priya ^2^ and Himel Mondal ^2^
*^1^* *All India Instituite of Medical Sciences, Patna, Bihar, India*
*^2^* *All India Instituite of Medical Sciences, Deoghar, Jharkhand, India*
**Objectives**: To systematically map and synthesize evidence on body dysmorphic disorder (BDD) in cosmetic and aesthetic surgery, focusing on prevalence, psychological correlates, screening performance, surgical outcomes, and emerging risk factors.
**Materials and Methods**: This systematic scoping review was conducted according to PRISMA guidelines. A comprehensive search of PubMed, PsycINFO, and Web of Science was performed for English-language peer-reviewed studies published between January 2016 and March 2026. Search terms included combinations of body dysmorphic disorder, cosmetic surgery, rhinoplasty, adverse childhood experiences, body shame, self-esteem, patient satisfaction, and psychological outcomes. Eligible studies were original research involving adults (≥18 years) seeking cosmetic procedures and using validated psychological instruments to assess preoperative psychological characteristics and/or postoperative outcomes. Reviews, editorials, case reports, qualitative studies without validated measures, surgical technique papers without psychological assessment, and studies unrelated to cosmetic or plastic surgery were excluded. Two reviewers independently screened studies, extracted data, and resolved disagreements by consensus or third-party adjudication. Owing to heterogeneity in design, populations, and outcomes, thematic qualitative synthesis was undertaken.
**Results**: From 455 records, 8 studies met the inclusion criteria. These studies included clinical, survey-based, and database-derived evidence with sample sizes ranging from 66 to 226,564. Screening-positive BDD prevalence ranged from 7.7% to 41.0%, with the highest burden among rhinoplasty candidates. Younger age, female sex, psychiatric history, and heavy social media filter use were associated with increased BDD risk. The BDDQ showed excellent screening performance, whereas surgeon-based recognition was poor. BDD-positive patients reported lower satisfaction and worse postoperative outcomes after rhinoplasty, while administrative coding identified BDD in fewer than 0.1% of cosmetic surgery patients, indicating substantial under recognition.
**Conclusion**: BDD is common, clinically important, and frequently missed in cosmetic surgery settings. Routine screening with validated tools, followed by multidisciplinary psychological assessment, is essential for safer patient selection and improved outcomes.

**2FA26/1256—Aesthetics**
**Management of Earlobe Keloid—A Maxillofacial Perspective!**
Melvinder Tyndall ^1^
*^1^* *Ministry of Health (MOH), Sibu, Malaysia*
**Introduction**: Keloids are benign fibroproliferative growths characterised by excessive collagen deposition extending beyond the original wound margins. Earlobe keloids are commonly associated with ear piercings or trauma which is an aesthetic concern. Bilateral ear involvement presents an additional challenge in achieving a symmetrical outcome while preventing its recurrence.
**Objectives**: To physically eliminate the benign fibro-collagenous growth on both ears.
To restore normal earlobe contours and its anatomical structure following scar removal.
To inhibit hyper-proliferation of fibroblasts and excess collagen synthesis during the wound healing process.
**Methods**: The keloid outline was marked and the central bulk was excised while leaving a peripheral rim or flap of normal looking skin. Intralesional Triamcinolone Acetonide injections were administered intraoperatively and repeated every 4 weeks post-surgery for up to 6 months. The earlobe was approximated with resorbable sutures in layers and superficially. Postoperatively, both ear were placed under magnetic pressure ear rings. Good wound care to ensure satisfactory wound healing with minimal scarring. No additional adjunctive therapies such as lasers or radiotherapy were employed in this case.
**Results**: Ear keloids generally have a much better prognosis than those on the chest or shoulders, with recurrence rates dropping significantly when combined adjuvant therapies are used. When surgical excision is paired with intralesional corticosteroid injections, recurrence rates drop to approximately 10% to 25%. Meticulous surgical techniques combined with adjuvant treatments leave fine, flat scars. However, patients might experience transient, minor side effects from steroids like local skin atrophy or mild depigmentation.
**Conclusion**: Earlobe keloids can be effectively managed through meticulous surgical technique combined with adjunctive therapies. Magnetic pressure ear rings are an effective yet fashionable alternative to traditional oyster clamps utilised in ear keloid management. A long-term follow-up remains essential due to the high risk of recurrence.

**2FA26/1260—Aesthetics**
**A Practical Nose-Excluded Photographic Superimposition Method for Objective Profile Assessment After Asian Rhinoplasty with Paranasal Augmentation**
Kiyomi Tachibana ^1,2^, Chihiro Matsui ^3^ and Shuo Tie ^1^
*^1^* *Zetith Beauty Clinic, Tokyo, Japan*
*^2^* *The University of Tokyo Hospital, Department of Otorhinolaryngology and Head and Neck Surgery, Tokyo, Japan*
*^3^* *Juntendo University School of Medicine, Plastic and Reconstructive Surgery Department, Tokyo, Japan*
**Objectives**: Quantitative evaluation of profile changes after rhinoplasty remains challenging because photographic registration is susceptible to technical inconsistency and methodological bias. Conventional alignment methods often rely on nasal landmarks, introducing circular bias because the nose itself is surgically altered. We developed a practical photographic superimposition protocol anchored to stable facial regions to reduce circular bias and enable analysis using routine clinical photographs without dedicated three-dimensional imaging systems.
**Materials and Methods**: This retrospective observational study included 81 consecutive Asian patients who underwent rhinoplasty with adjunctive paranasal augmentation between April 2022 and April 2025. Standardized lateral photographs were obtained preoperatively and 6 months postoperatively. Images were superimposed using the forehead and periorbital area while excluding the nose from registration. Primary outcomes were postoperative/preoperative projection indices for R1, R2, and R3. The nasolabial angle was evaluated as a secondary outcome. Intra-rater reliability was assessed in a stratified random subset of 30 cases using intraclass correlation coefficients and Bland–Altman analysis.
**Results**: The cohort included 40 primary and 41 revision cases, with a mean age of 31.4 ± 7.6 years; 71 patients were female. All projection ratios were significantly greater than 1.0 (all *p* < 0.001). The nasolabial angle increased by 7.07 ± 11.13 degrees (*p* < 0.001). Intra-rater reliability ranged from 0.832 to 0.984. Exploratory subgroup analysis revealed no significant differences between primary and revision cases. Postoperative complications were infrequent.
**Conclusion**: Nose-excluded photographic superimposition provides a practical and reproducible framework for objective profile assessment after rhinoplasty with paranasal augmentation. By reducing circular bias and using standardized clinical photographs with free image-analysis software, this method may facilitate routine postoperative evaluation without dedicated three-dimensional imaging systems.
Conflict of interest disclosure:
Receipt of grants/research supports: KAKENHI

**FA26/99—Basic and Translational Research**
**Gum Arabic Exfoliated Molybdenum Diselenide Nanosheets to Enhance the Osteoinductive Property of Polyether Ether Ketone Implant**
Rohan Thomas Mathew ^1^ and Venkatesan Jayachandran ^2^
*^1^* *Zulekha Yenepoya Institute of Oncology, Yenepoya (Deemed to be University), Mangalore, India*
*^2^* *Malla Reddy Vishwavidyapeeth (Deemed to be University), Hyderabad, India*
**Introduction**: Implants are the standard of care for craniofacial reconstruction. Metallic implants are the gold standard, and titanium is the most commonly used material. Polyether ether ketone (PEEK) is another material that has good mechanical properties and can be 3D printed to create PSI. However, its low osseoinductive properties limit its application in craniofacial reconstruction, where integration is essential. In this study, we aimed to demonstrate that gum Arabic-mediated liquid exfoliation of molybdenum diselenide (MoSe_2_) and coating on PEEK by dip-coating method can modify PEEK into osseoinductive material.
**Objectives**: To investigate the capacity of MoSe2 Nanosheets to improve the osseoinductive properties of Polyether ether ketone (PEEK) implants.
**Materials and Methods**: Liquid exfoliation of MoSe_2_ using gum Arabic is performed to create MoSe_2_ nanosheets. Gum Arabic-exfoliated MoSe_2_ (GA-MoSe_2_) was chemically characterized using multiple analytical techniques. UV-Vis and FT-IR analyses confirmed the presence of nanosheets. Nano-sheet flakes were also identified and analysed by scanning electron microscope. Small discs (5–7 mm) were made from PEEK filament. They were surface-modified via acid etching method and then coated with GA-MoSe_2_ nanosheets by coating and drying. The discs were photographed using a stereomicroscope and characterized using FTIR and scanning electron microscope. In vitro tests, such as protein adsorption, cell viability, and alkaline phosphatase (ALP) assays using MG-63 cells, were performed to assess biocompatibility and osseoinductive properties.
**Results**: The MoSe_2_ dispersion uniformly adhered to the porous structures of the PEEK surface. The acid-etched PEEK-GA-MoSe_2_ discs were biocompatible with the MG-63 cells. High ALP activity was observed on acid etched-PEEK-GA-MoSe_2_ with MG 63 cells compared to the control.
**Conclusion**: The acid etched-PEEK-GA-MoSe_2_ material is a potential candidate for osseointegrative implants and bone grafts. However, this is a preliminary study, and further development and extensive testing must be undertaken before the material can be safely used in patients.
Conflict of interest disclosure
Receipt of grants/research supports: Part of the study is supported by a cancer research grant from the Faizal Shabana Foundation.
Stock shareholder: The authors have received multiple institutional seed grants and extra mural fundings for other projects. Complete list of funded projects and grants will be given upon request

**FA26/260—Basic and Translational Research**
**Distinct Ductal Morphometric Phenotypes in Salivary Gland Diseases: A 3D Quantitative Analysis**
Nadav Grinberg ^1^, Ariel Pokhojaev ^1^, Deborah Dahan ^1^, Clariel Ianculovici ^1^, Shlomi Kleinman ^1^, Gal Frenkel ^1^, Oren Peleg ^1,2^
*^1^* *Tel-Aviv Sourasky University Medical Center, Tel-Aviv, Israel*
*^2^* *The Maurice and Gabriela Goldschleger School of Dental Medicine, Grey Faculty of Medical & Health Sciences, Tel Aviv University, Tel-Aviv, Israel*
**Objectives**: To perform quantitative three-dimensional analysis of major salivary glands using CBCT-based sialography and to compare ductal and glandular morphometric characteristics across different salivary gland diseases in order to identify imaging features that may improve diagnostic differentiation.
**Materials and Methods**: This retrospective study included patients who underwent CBCT sialography at our institution between 2023 and 2025. The cohort included 205 parotid and 46 submandibular glands. Fifty representative volumes were manually segmented in 3D Slicer to generate training data for a deep-learning segmentation pipeline based on a 3D convolutional neural network (MONAI U-Net/SegResNet). All datasets were pre-processed using percentile-based intensity normalization and standardized region-of-interest padding prior to automated segmentation. The trained model generated volumetric gland and duct segmentations, which were subsequently reviewed and refined for morphometric analysis. Quantitative parameters extracted from the three-dimensional reconstructions. Case-wise statistical analysis was performed using Python-based scientific libraries.
**Results**: Case-wise analysis included 136 parotid and 44 submandibular glands. Obstructive salivary gland diseases demonstrated a distinct morphometric phenotype characterized by significantly larger duct mesh volume, greater duct thickness, and lower duct area-to-volume ratios, indicating a larger-caliber three-dimensional ductal architecture. In parotid glands, sialolithiasis and chronic obstructive sialadenitis showed markedly enlarged ductal structures compared with Sjögren’s syndrome, xerostomia, and asymptomatic controls (*p* < 0.001), while gland-tree morphometry remained largely unchanged, suggesting that the main structural differentiation occurs within the ductal compartment. In the submandibular glands, sialolithiasis demonstrated the most pronounced morphometric remodeling, with significant enlargement of ductal structures and changes in gland-tree parameters, indicating a broader three-dimensional structural response to obstruction.
**Conclusion**: Quantitative 3D CBCT sialography enables objective characterization of salivary gland architecture and reveals distinct morphometric signatures that differentiate obstructive from non-obstructive salivary gland disease, highlighting the potential of advanced imaging combined with deep-learning segmentation as a minimally invasive diagnostic biomarker for salivary gland pathology.

**FA26/351—Basic and Translational Research**
**Molecular Profiling of the Perineural Microenvironment of Oral Squamous Cell Carcinoma: A Spatial Transcriptomics Study**
Sreeram MP ^1^, Shanthi Velusamy ^1^, Aruna Korlimarla ^1^, Karthik N Rao ^1^, Prajwal Dange ^1^, Hari PS ^1^, Akhileshwar Namani ^1^, Rekha Vijaykumar ^1^ and B S Srinath ^1^
*^1^* *Sri Shankara Cancer Hospital and Research Centre, Bengaluru, India*
**Objectives**: Perineural invasion (PNI) the spread of tumour cells along neural structures is an independent predictor of locoregional recurrence and reduced survival in oral squamous cell carcinoma (OSCC), yet is reported binary (present/absent), masking profound biological heterogeneity. The molecular architecture of the perineural microenvironment in OSCC remains entirely uncharacterised.
**Materials and Methods**: We performed the first spatially resolved transcriptomic characterisation of the perineural tumour microenvironment in OSCC using the GeoMx Digital Spatial Profiler Whole Transcriptome Atlas (~18,677 probes). Six patients (four PNI-positive, two PNI-negative) contributed 45 regions of interest across four compartments: invaded nerve, perineural stroma, perineural tumour, and uninvolved nerve. Data were Q3-normalised; differential expression was tested using linear mixed models accounting for within-patient correlation.
**Results**: The dominant transcriptional event was profound desmoplastic stromal reprogramming: 332 differentially expressed genes (FDR < 0.05) separated perineural from uninvolved stroma. A coordinated cancer-associated fibroblast (CAF) signature emerged POSTN (+5.55 log_2_FC), COL1A1 (+5.01), SPARC (+3.84), CTHRC1 (+3.71) accompanied by 58 upregulated ribosomal protein genes (RPL/RPS family), indicating massive translational upregulation for ECM production. Midkine (MDK, +2.52) was the highest-enriched secreted neurotropic factor in perineural stroma, supporting an active tumour-to-nerve chemotactic axis. Galectin-1, TGF-β1, and Activin-A co-upregulation defined a multi-pathway immunosuppressive perineural niche. Critically, tumour regions proximal versus distal to invaded nerves differed in only two genes (DUSP14, GSDMC), establishing the stroma not tumour epithelium as the primary transcriptionally active driver of PNI.
**Conclusion**: These data constitute the first spatial molecular atlas of the perineural niche in OSCC. A POSTN-MDK-LGALS1-TGFβ1 stromal axis emerges as the dominant mediator of neural invasion. This pilot underpins to molecularly subtype PNI into low-risk and high-risk categories, validate biomarkers by qPCR and IHC, and correlate spatial signatures with disease-free survival, transforming PNI from binary pathology into a molecularly stratified, clinically actionable risk stratifier.

**FA26/472—Basic and Translational Research**
**Insights into the Effects of Carbamylated Erythropoietin on Schwann Cells in Peripheral Nerve Injury**
Ting Chak Lam ^1^
*^1^* *The University of Hong Kong, Hong Kong, China*
**Objectives**: To investigate the therapeutic potential of carbamylated erythropoietin (CEPO), a non-hematopoietic derivative of erythropoietin (EPO), in promoting peripheral nerve regeneration, and to evaluate its efficacy and safety as an alternative to conventional EPO treatment, which is associated with systemic thrombotic complications.
**Materials and Methods**: CEPO was synthesised and examined through both in vitro and in vivo experimental models. In vitro studies were conducted using cultured Schwann cells to evaluate cell viability, proliferation, migration, and nerve growth factor (NGF) expression under varying concentrations of CEPO, with the optimal concentration identified. For in vivo analysis, a sciatic nerve injury model was established, followed by local administration of CEPO. Functional recovery, axonal regeneration, and overall nerve repair were assessed at 21 days post-injury using histological and functional evaluation methods.
**Results**: In vitro results demonstrated that CEPO significantly enhanced Schwann cell viability, proliferation, and migratory capacity in a dose-dependent manner, with the optimal effect observed at 25 μg/mL. Additionally, CEPO markedly upregulated NGF expression, indicating its role in creating a favourable microenvironment for nerve regeneration. In vivo findings revealed that the CEPO-treated group exhibited significantly improved functional recovery compared to controls. Enhanced axonal regeneration across the injury site was observed, suggesting effective guidance of regenerating nerve fibres and improved structural repair.
**Conclusion**: CEPO effectively promotes peripheral nerve regeneration by enhancing Schwann cell function and possible neurotrophic signalling while avoiding the hematopoietic and thrombotic risks associated with EPO. These findings support CEPO as a promising and safer therapeutic candidate or adjunctive strategy for the clinical management of peripheral nerve injuries. Future studies investigating these underlying molecular pathways will be essential to comprehensively evaluate and validate CEPO as a viable therapeutic approach for improving nerve regeneration and restoring locomotor function after peripheral nerve injury.

**FA26/479—Basic and Translational Research**
**Peptide–Polyphenol Network-Stabilized Microalgal Thylakoid Vesicles for Light-Driven Energy Supplementation in TMJOA**
Linli Jiang ^1^
*^1^* *West China Hospital of Stomatology, Sichuan University, Chengdu, China*
**Objectives**: Temporomandibular joint osteoarthritis (TMJOA) is characterized by cartilage degeneration and chondrocyte energy deficiency, with marked ATP and NADPH depletion. Restoring cellular energy homeostasis is critical for delaying disease progression, yet effective therapies remain limited. This study aims to develop a light-driven, cartilage-targeted microalgal thylakoid vesicle system to restore chondrocyte metabolism, reduce oxidative stress, and promote cartilage repair.
**Materials and Methods**: Thylakoid vesicles were isolated from Chlorella vulgaris and coated with a peptide–polyphenol network (PPN) incorporating cartilage-targeting peptides to enhance stability and chondrocyte specificity. In vitro, degenerative TMJ chondrocytes were treated with PPN-coated vesicles under light exposure. ATP and NADPH levels, reactive oxygen species (ROS), mitochondrial function, apoptosis, and matrix protein expression were assessed. In vivo, vesicles were intra-articularly injected into a TMJOA rat model, and cartilage integrity, inflammation, and joint function were evaluated using histology, immunostaining, and behavioral assays.
**Results**: PPN-stabilized microalgal vesicles efficiently generated ATP and NADPH under light, restored chondrocyte energy homeostasis, reduced oxidative stress, inhibited apoptosis, and enhanced matrix protein synthesis. In vivo, vesicle treatment preserved cartilage structure, suppressed inflammation, and improved joint function without detectable adverse effects.
**Conclusion**: Cartilage-targeted, PPN-stabilized microalgal thylakoid vesicles offer a novel light-driven, non-mitochondrial energy supplementation strategy. By reprogramming chondrocyte metabolism and redox balance, this biohybrid system represents a promising translational approach for TMJOA therapy and metabolic intervention in degenerative joint diseases.
Conflict of interest disclosure
Receipt of grants/research supports: GZC20251211

**2FA26/1279—Basic and Translational Research**
**Perimandibular Tissue Response in Medication-Related Osteonecrosis of the Jaw Associated with Anti-RANKL Antibody Agents**
Masako Fujioka-Kobayashi ^1^, Reon Morioka ^1,2^, Hiroto Tatsumi ^1,3^ and Takahiro Kanno ^1^
*^1^* *Department of Oral and Maxillofacial Surgery, Shimane University Faculty of Medicine, Izumo, Japan*
*^2^* *Department of Pharmacology, Shimane University Faculty of Medicine, Izumo, Japan*
*^3^* *Innovative Cancer Center, Shimane University Hospital, Izumo, Japan*
**Objectives**: Anti RANKL antibody agents, such as denosumab, are associated with the development of medication related osteonecrosis of the jaw (MRONJ). However, few studies have examined their effects on perimandibular soft tissues. This study investigated the effect of anti RANKL antibody on perimandibular soft tissue responses in vitro and in clinical MRONJ cases.
**Materials and Methods**: Gingival fibroblasts were isolated from perimandibular lesions of C57BL/6J mice administered anti-mouse RANKL neutralizing antibody (mAb). Cellular responses under mAb stimulation were evaluated on primary gingival fibroblasts. In addition, macrophage related gene expression was histopathologically examined in perimandibular soft tissues, particularly the surrounding periosteum, from human MRONJ cases associated with denosumab.
**Results**: In mAb-treated mice, bone volume and bone mineral density were increased in both the mandible and the distal femur. mAb did not affect cell adhesion or viability; however, it significantly decreased mRNA levels of fibronectin and matrix metalloproteinases (MMP-2 and MMP-3) in primary gingival fibroblasts, particularly under LPS stimulation. In clinical MRONJ cases, macrophages were observed in perimandibular regions, and polarization toward M1 and M2 phenotypes showed slight changes, especially around new bone formation sites associated with periosteal reaction.
**Conclusion**: The effects of anti-RANKL antibody agents on perimandibular soft tissues were evaluated. Minor alterations in extracellular matrix–related gene expression and macrophage polarization were observed in anti-RANKL antibody treated perimandibular lesions. The present study suggested that macrophages might play a role in regulating periosteal bone reaction even under inflammatory conditions in denosumab-related MRONJ.
Conflict of interest disclosure:
Receipt of grants/research supports: AOCMF Start-up Grant 2025

**FA26/44—Cleft Lip and Palate**
**Alveolar Bone Grafting Using Polycaprolactone Scaffold Bone Filler in Cleft Lip and Palate Patient: A Case Report**
Thanyaphat Engboonmeskul ^1^ and Saran Worasakwutiphong ^1^
*^1^* *Naresuan University, Phitsanulok, Thailand*
**Objectives**: To evaluate the clinical efficacy of polycaprolactone (PCL) scaffolds as a bone filler in alveolar bone grafting for cleft lip and palate patients.
**Materials and Methods**: This retrospective case series (IRB No. P1-0179/2568) included two cleft lip and palate patients who underwent alveolar bone grafting using PCL scaffolds at Naresuan University Hospital (2022–present). Standardized four-corner flaps were used, and the defect was filled using 1.5–2 mL of autologous iliac cancellous bone graft supported by a trimmed PCL scaffold (Osteomesh). Volumetric Bone Gain (VBG) was analyzed via CBCT scans using 3D Slicer at four intervals: pre-op, immediate post-op, and at 3 and 6 months.
**Results**: Both patients achieved successful graft integration without complications. Case 1 showed an immediate overcorrection (183.31% fill), stabilizing to 83.8% (980.34 mm^3^) at 6 months. Case 2 reached 126.38% immediate fill, which reduced to 59.81% (468.01 mm^3^) at 6 months. Despite varying retention rates, both cases provided sufficient bone support for successful orthodontic treatment and functional rehabilitation.
**Conclusion**: PCL scaffolds, used in combination with autologous bone, are a viable material choice for alveolar cleft reconstruction, providing adequate structural support for subsequent dental interventions.
Conflict of interest disclosure
Support: Medev Healthcare Co., Ltd. provided polycaprolactone bone substitute materials used in two patients included in this study. The authors declare that there are no conflicts of interest that could have influenced the study design, data collection, analysis, interpretation of results, or preparation of the manuscript. The company had no role in the design, conduct, or reporting of the study. This research was conducted solely for academic purposes, and the findings will not be used for commercial purposes.

**FA26/226—Cleft Lip and Palate**
**Case Studies in Premaxillary Osteotomy: Managing a Non-Fitting Occlusal Splint with Simultaneous Gingivopalatoplasty**
Nurlidiah Md Ghazali ^1^, Sundrarajan Ramasamy ^1^ and Rosliza Parumo ^2^
*^1^* *Hospital Sultan Ismail, Johor, Malaysia*
*^2^* *Hospital Sultanah Aminah, Johor, Malaysia*
**Objectives**: To evaluate the effectiveness of different stabilization strategies—mechanical fixation versus “biological splinting”—following premaxillary osteotomy in bilateral cleft patients with varying clinical needs.
**Materials and Methods**: This report compares two distinct cases.
  - **Case 1:** A 10-year-old patient required premaxillary advancement for a skeletal reverse overjet. We used bilateral buccal incisions and a tunnelling technique to preserve the blood supply. Stabilization was achieved using a conventional acrylic occlusal splint based on stone model surgery.
  - **Case 2:** A 4-year-old hyperactive patient required a premaxillary setback for a severely protruding segment that caused frequent trauma and incompetent lip seal. We resected the vomer bone via a palatal approach. Due to uncooperative behaviour, we used an intraoral scan for 3D planning, but the resulting occlusal splint was non-fitting intraoperatively. We instead stabilized the segment using “biological splinting” through a simultaneous gingivopalatoplasty (GPP), relying on circumferential soft tissue tension.
**Results**: Both patients achieved successful outcomes. In Case 1, a lateral cephalogram at six months confirmed the planned advancement and successful bony union with no necrosis. In Case 2, despite the non-fitting splint, the premaxillary segment was clinically firm and non-mobile at the two-week follow-up. The GPP provided an immediate competent lip seal and effectively protected the bone from further injury.
**Conclusion**: An occlusal splint remains the standard for mechanical fixation. However, simultaneous gingivopalatoplasty serves as a reliable “biological” backup. This technique ensures segment stability when mechanical guides fail, particularly in pediatric cases where digital or conventional planning is challenging

**FA26/366—Cleft Lip and Palate**
**Optimizing a Standard Set for Cleft Lip and Palate Through Multicenter and International Collaboration: Integrating Evidence and Clinical Practice**
Saranda Ombashi ^1^, Philip van der Goes ^1^, Eppo Wolvius ^1^, Aebele Mink van der Molen ^2^ and Sarah Versnel ^1^
*^1^* *Erasmus Medical Center, Rotterdam, The Netherlands*
*^2^* *University Medical Center Utrecht, Utrecht, The Netherlands*
**Objectives**: To evaluate and optimize the International Consortium for Health Outcomes Measurement (ICHOM) Standard Set for cleft lip and palate (CL/P), this study aimed to integrate clinical experiences and previously conducted data analyses from participating centers to develop an updated, more practical and clinically relevant outcome set.
**Materials and Methods**: Previous studies of participating ICHOM cleft centers were used as a base for this study. Additionally, a structured literature review was performed to identify other relevant studies. A panel of clinicians from the participating centers was convened for online plenary sessions. Semi-structured discussions addressed utility, feasibility, data quality, and barriers to implementation/usage. Outputs were analyzed and combined with literature findings. Based on the findings, modifications to outcome domains, measurement instruments, and timing were proposed.
**Results**: Recommendations were identified across all outcome domains. For dental and oral health, usefulness of the COHIP-OSS was reconsidered. Concerning the appearance domain, current measures were validated and additional PROM scales and new measurement moments were proposed. PROMs for eating, drinking and breathing showed limited psychometric performance, suggesting revision or removal. In the speech domain, measures refinements in scoring and additional tools were recommended. Concerning the hearing domain, assessment should be expanded with earlier and more comprehensive hearing evaluations to identify problems timely. Psychosocial measures were found reliable, with recommendations to better tailor assessments to age. Panel input highlighted the value of PROMs. Challenges in data collection and infrastructure where discussed too, leading to a refined outcome set.
**Conclusion**: The ICHOM Standard Set for CL/P remains a valuable framework for outcome measurement and benchmarking. Targeted refinements in outcome selection, timing, and implementation are required to enhance clinical applicability and reduce burden. The proposed updated set, integrating evidence and real-world experience, provides an improved tool for standardized outcome assessment, supporting improved quality of care and international collaboration.

**FA26/367—Cleft Lip and Palate**
**Standardizing the Patient Perspective in European Cleft Care: Translating and Validating PROMs and Developing Methods to Interpret Outcomes in a Meaningful Way**
Saranda Ombashi ^1^, Sarah Versnel ^1^, Aebele Mink van der Molen ^2^ and Eppo Wolvius ^1^
*^1^* *Erasmus Medical Center, Rotterdam, The Netherlands*
*^2^* *University Medical Center Utrecht, Utrecht, The Netherlands*
**Objectives**: Patient Reported Outome Measures (PROMs) are increasingly used in cleft and craniofacial care centers. In order to ensure that collected PROM data is both applicable for research and useful on an individual/clinical level, data should be valid and reliable. Within the European Reference Network for Rare Craniofacial Anomalies and Ear-Nose-Throat disorders (ERN-CRANIO), an interest in collecting PROMs for Cleft lip and palate (CL/P) in a systematic, clinically meaningful matter was expressed. The aim of the current study is to translate and validate relevant PROMs in several European languages and to improve meaningful interpretation through additional data analyses.
**Materials and Methods**: Based on literature and previous research projects, consensus was reached in separate groups within the ERN CRANIO. Contact was sought with the developers of the relevant PROMs and protocols for translation were followed. For further standardization and interpretation, normative values for CLEFT-Q scales were developed. Additional analyses were performed to map out other ways to increase meaningful interpretation.
**Results**: All relevant PROMs for CL/P were identified and translation/validation projects were set up for the CLEFT-Q questionnaire; FACE-Q questionnaire; Pediatric Sleep Questionnaire; and VELO-questionnaire in the following languages: Catalan, Dutch, Finnish, Hungarian, Italian, Latvian, Polish and Spanish. Translations were carried out by professional translators and developed through a consensus formed by two forward translations, following a backward translation. Validation interviews were done in each cleft center. Normative values of the CLEFT-Q were established in a Dutch non-cleft population. By creating anchor measures and by exploring the minimally clinically important differences, the interpretation of PROM results is improved.
**Conclusion**: This study shows the steps undertaken in a European CL/P initiative to capture the patients perspective in a standardized, meaningful way. New research ideas, including similar PROM projects in other craniofacial conditions and development of normative values in other languages/cultures are suggested.

**FA26/369—Cleft Lip and Palate**
**Evaluating a Modified Dibbell Technique for Micro- and Minor-Form Cleft Lip Nose Correction**
Jingtao Li ^1^
*^1^* *West China Hospital of Stomatology, Sichuan University, Chengdu, China*
**Objectives**: Primary cleft lip nose correction is challenging even for patients with incomplete cleft. Here we propse a modified Dibbell technique for primary cleft repair with radical alar cartilage repositioning and philtral ridge reconstruction.
**Materials and Methods**: Patients with micro- or minor-form cleft lip who were operated between 2018 and 2021 by the same surgeon were reviewed. Patients receiving either Tajima or modified Dibbell technique were enrolled. All enrolled cases were of at least three-year followup with three-dimensional facial morphology captured. Those with incompete records or syndormic cleft were excluded. A total of 22 linear and angular measurements were performed along with the nasal symmetry index calculated.
**Results**: A total of 212 cases were retrieved, with 82 operated by Dibbell technique and 132 by Tajima. The Dibbell group demonstrated significantly superior symmetry in both nose and philtrum as revealed by the measurements.
**Conclusion**: The modified Dibbell technique is a valuable technique selection for the correction of micro- and mino-form cleft lip nose.

**FA26/407—Cleft Lip and Palate**
**The Impact of Alveolar Bone Grafting on the Long-Term Stability of Secondary Cleft Rhinoplasty**
Jingtao Li ^1^
*^1^* *West China Hospital of Stomatology, Sichuan University, Chengdu, China*
**Objectives**: Alveolar bone grafting (ABG) has been supposed to restore the symmetry of bony support underlying lower nasal base. The longterm influence of ABG on the rhinoplasty outcome, however, has rarely been discussed. This study set out to explore whether ABG contributes to the stability of secondary cleft rhinoplasty
**Materials and Methods**: Patients with complete unilateral cleft lip and alveolae who received secondary rhinoplasty between 2017 to 2022 in a tertiary hospital-based cleft center were reviewed. The enrolled patients were grouped into ABG and control (no ABG) groups. The ABG outcome was graded using cone-beam computed tomography and those with vertical bone resorption greater than 1/3 were excluded. Patients below the age of 14 were also excluded. The three-dimensional nasolabial morphology was captured using a 3DMD system before, immediately after and one year after surgery. A total of 22 linear and angular measurements were performed along with the nasal symmetry index calculated. Both intergroup and intragroup comparison was performed. The stability of rhinoplasty was evaluaed by longitudinal paired comparison between measuremts at different postoperative timepoints in the term of percentage.
**Results**: A total of 121 cases with complete records were retrieved, with 50 in the ABG group and 71 in the control. Both groups acquired significant improvement in nasal symmetry. No signifcant differnce was observed at any timepoints between the two groups. In addition, there was no significant difference in term of rhinoplasty stability between the ABG group (14.8% relapse) and the control (17.4% relapse).
**Conclusion**: Our study suggested no influence of ABG on the longterm stability of unilateral cleft secondary rhinoplasty.

**FA26/411—Cleft Lip and Palate**
**Peri-Port Muscle Reconstruction in Posterior Pharyngeal Flap Improves Both Ventilation and Speech Outcome**
Jingtao Li ^1^
*^1^* *West China Hospital of Stomatology, Sichuan University, Chengdu, China*
**Objectives**: Balancing nasal ventilation and velopharyngeal closure is challenging in performing phayrngeal flap (PF). This study proposed a modification with narrower flap and “dynamic” lateral ports and compared the outcome with conventional Hogan PF.
**Materials and Methods**: Patients who received PF between 2020 and 2023 in a tertiary hospital-based cleft center were reviewed and grouped into conventional PF group and modified PF group. For the modified PF, the flap width was only 2/3 of conventional PF, and the levator palatine was dissected and coupled with superior constrictor around the lateral ports. Consequently, the ports were larger and supposely more muscle-functional as compared with conventional PF. The pre-and postoperative speech and ventilation status was retrieved and compared.
**Results**: A total of 71 cases were enrolled, with 45 receiving conventional PF and 31 receiving modified PF. The preoperative velopharyngeal closure rate was comparable between the two groups. The group receiving modified PF demonstrated significantly higher rate of velophayrngeal competence (89.1% vs. 72.3%) and lower occurrence of nasal airway obstruction (7.6% vs. 19.2%). Nasoendoscopy examination revealed dynamic tissue mobility around the port following modified PF.
**Conclusion**: Our PF modification with peri-port muscle reconstruction achieved better balancing between speech and ventilation.

**FA26/414—Cleft Lip and Palate**
**Comparison Between Square-Root Palatoplasty and Conventional Furlow Palatoplasty in Relaxing Incision Use, Wound Healing and Speech Outcome: A Randomized Controlled Trial**
Jingtao Li ^1^
*^1^* *West China Hospital of Stomatology, Sichuan University, Chengdu, China*
**Objectives**: Furlow palatoplasty (FP) with double-opposing Z-plasty is effective in velum lengthening but potentially increases the risk of wound dehiscence with higher suture tension. Square-root palatoplasty (SP) with adjusted angles of Z-plasty limb was previously proposed to reduce suture tension, but its actual effectiveness has not been authenticated.
**Materials and Methods**: Patients with nonsyndromic cleft palate who visited our center between 2019 January and 2020 December were enrolled and randomly assigned into FP group and SP group. All procedures were perfomed by the same experienced surgeon. The cleft width and pharyngeal depth was measured before surgery, and the use of relaxing incision was recorded. The occurrence of flap dehiscence was followed up at 2 weeks after surgery. The fistulae formation and speech performance was evaluated at the age of five. Both univariate and multivariate analyses were performed.
**Results**: A total of 120 cases were enrolled, with 82 of full follow-up records (37 in FP group and 45 in SP group). No significant difference was detected in cleft type, age, gender or presurgical velopharyngeal measurements between the two groups. The two techniques produced comparable rate of primary velopharyngeal competence at five (81.1% for FP and 86.7% for SP). The SP group demonstrated significantly low rate of flap dehiscence (11.1%) and fisutlae (2.2%) as compared with the FP group (29.7% flap dehiscence and 10.8% fistulae).
**Conclusion**: The audience will be delivered with the evidence demonstrating the advantages of a modified double-opposing Z-plasty cleft palate repair.

**FA26/446—Cleft Lip and Palate**
**AI-Assisted Simulation of Postoperative Cleft Lip Appearance Using Paired Preoperative and Postoperative Photographs**
Joseph Kyu-hyung Park ^1,2^, Baek-Kyu Kim ^1,2^, Nakwon Choi ^1^ and Jun Seo Yoon ^1^
*^1^* *Seoul National University Bundang Hospital, Seongnam-si, Republic of Korea*
*^2^* *Seoul National University College of Medicine, Seoul, Republic of Korea*
**Objectives**: Preoperative counseling for cleft lip repair relies heavily on surgeon explanation and representative clinical images, which may not accurately reflect individual postoperative appearance. This study aimed to develop and evaluate a preliminary artificial intelligence–assisted image translation workflow for simulating postoperative cleft lip appearance from preoperative facial photographs.
**Materials and Methods**: A retrospective dataset of paired preoperative and postoperative frontal facial photographs from patients who underwent primary cleft lip repair was assembled. Images were standardized by facial alignment, cropping, resolution normalization, and paired registration. A conditional image-to-image translation model was trained to generate simulated postoperative images from preoperative inputs. Model performance was assessed using image similarity metrics between generated and actual postoperative photographs, preservation of key facial structures, and blinded qualitative evaluation by plastic surgeons. Reviewers scored anatomical plausibility, surgical realism, and potential usefulness for patient counseling using a structured rating scale.
**Results**: The model generated visually plausible postoperative simulations that reflected correction of the cleft-side lip contour, philtral alignment, and nasal base asymmetry while preserving overall facial identity. Quantitative similarity analysis demonstrated closer correspondence between generated images and actual postoperative photographs than between preoperative and postoperative image pairs. In blinded review, surgeons rated most generated images as anatomically plausible and potentially useful as a counseling aid, although residual artifacts were observed around the vermilion border, nostril sill, and scar region.
**Conclusion**: AI-assisted postoperative simulation using paired clinical photographs may provide a feasible adjunct for cleft lip surgical counseling and outcome visualization.

**FA26/465—Cleft Lip and Palate**
**Flap Selection in Oro-Nasal Fistula Repair: A Case Series in Cleft Lip and Palate**
Danaiya Supakanjanakanti ^1^
*^1^* *Department of Oral and Maxillofacial Surgery, Faculty of Dentistry, Prince of Songkhla University, Hat Yai, Songkhla, Thailand*
**Objectives**: Oro-nasal fistula is a common complication after palate repair in cleft lip and palate patients, causing nasal regurgitation and speech problems. Oro-nasal fistula closure is also frequently required prior to alveolar bone grafting to establish a hermetic seal and optimize graft conditions. This study aimed to describe the surgical outcomes of oro-nasal fistula closure and evaluate flap selection based on fistula characteristics.
**Materials and Methods**: A retrospective case series of 11 cleft lip and palate patients who underwent oro-nasal fistula closure between November 2020 and April 2026 was conducted. Fistulae were classified by anatomical position (anterior, posterior, midline) and size. Local flap was used for small-to-moderate fistulae; tongue flap for large fistulae. One patient with multiple large fistulae underwent a planned two-stage procedure. Most cases were performed before alveolar bone grafting. Follow-up was at 3, 6, and 12 months.
**Results**: Eleven patients (8 female, 3 male; mean age 13.5 years, range 4–19) were included. Six underwent local flap repair, four tongue flap repair, and one a two-stage combination. Nine cases were performed before alveolar bone grafting, one after, and one required no grafting. Outcomes were satisfactory overall. One patient developed partial tongue flap necrosis requiring debridement but achieved successful closure due to an intact nasal layer. One patient had minimal residual fistula managed at the time of alveolar bone grafting. No other major complications occurred.
**Conclusion**: Local and tongue flap techniques provided satisfactory oro-nasal fistula closure in cleft lip and palate patients. Technique selection based on fistula size and anatomical position is essential. An intact nasal layer can be a salvage factor following partial flap necrosis. Oro-nasal fistula closure prior to alveolar bone grafting facilitates optimal graft conditions. A staged approach is effective for complex cases.

**FA26/466—Cleft Lip and Palate**
**Secondary Open Rhinoplasty in Cleft Patients: Individualized Reconstruction and Clinical Outcomes**
Teepawat Witeerungrot ^1^
*^1^* *Department of Oral and Maxillofacial Surgery, Faculty of Dentistry, Prince of Songkla University, Songkhla, Thailand*
**Objectives**: Nasal deformity is a common sequela of cleft lip and palate that often persists after primary repair, necessitating secondary rhinoplasty. This case series describes surgical techniques, graft strategies, and outcomes of secondary open rhinoplasty in four cleft patients.
**Materials and Methods**: Four female patients aged 18 to 24 years underwent secondary open rhinoplasty following staged cleft repair. Three had unilateral and one had bilateral cleft nasal deformity. All procedures used an open approach with costal cartilage as the primary graft source. A consistent framework was applied: extended spreader grafts for midvault reconstruction, columella strut grafts for tip projection, and shield grafts for tip refinement. Dorsal augmentation was individualized—Cases 1 and 2 received diced costal cartilage; Case 3 had a silicone implant with concurrent dermofat lip augmentation; Case 4 underwent augmentation with a silicone implant reinforced by costochondral cartilage. Outcomes were assessed by clinical examination and CBCT soft tissue profile analysis at 6 to 12 months postoperatively.
**Results**: All procedures were completed without intraoperative complications. CBCT analysis showed consistent improvements in nasal projection, dorsal contour, tip definition, and columella height. Cases 3 and 4 had uneventful recoveries without revision. In Cases 1 and 2, late dorsal irregularity from diced cartilage resorption or migration was managed successfully with minor revision surgery. No implant exposure or deep infection occurred.
**Conclusion**: Secondary open rhinoplasty with individualized costal cartilage reconstruction yields reliable improvement in cleft nasal deformity. Extended spreader, columella strut, and shield grafts provide a reproducible foundation for unilateral and bilateral cases alike. Silicone-based augmentation produced stable contour, while diced costal cartilage carries a risk of late irregularity from resorption or migration requiring minor revision, patients should be counseled accordingly. CBCT soft tissue analysis is recommended for objective postoperative assessment.

**2FA26/1065—Cleft Lip and Palate**
**Premaxillary Osteotomy in Infants Born with Bilateral Cleft Lip Prior to NAM**
Sathish Vasishta ^1^
*^1^* *Aster CMI Hospital, Bengaluru, India*
**Objective**: To evaluate efficacy of premaxillary osteotomy much prior to NAM in infants born with Bilateral cleft lip with protruding pre maxilla.
**Materials and Methods**: Infants born with Bilateral cleft lip with protruding pre maxilla included in the study.
Pre maxillary osteotomy was carried out anterior to VPS within 10 days of birth & NAM therapy was started, continued for 8–12 weeks until definitive lip repair.
**Results**: Our results showed that there was improved columellar height following pre-maxillary osteotomy, better compliance, and better surgical outcome following lip repair.
**Conclusion**: Premaxillary osteotomy is an effective method in severely protruding maxilla. Helps in increasing columellar height & better surgical outcome after NAM.

**2FA26/1102—Cleft Lip and Palate**
**Alloplastic Complications in Cleft Rhinoplasty: Delayed Silicone Implant Infections in Two (2) Patients**
Wan Izzuddin Wan Zaidi ^1^, Ainilhusna Amin ^1^, Mohammad Azrin Abd Samad ^1^, Anis Najihah Mohd Ismail ^1^, Mohd Kherman Suparman ^1^, Nazer Berahim ^1^, Azmeel Mazlee Anuar ^1^ and Hazmyr Abdul Wahab ^1^
*^1^* *Centre of Study for Oral and Maxillofacial, Faculty of Dentistry, Universiti Teknologi MARA (UiTM), Selangor, Malaysia*
**Introduction:** Nasal asymmetry is one of the most common defining characteristics of cleft lip and palate (CLP), which can profoundly affect both aesthetic presentation and respiratory function. Therefore corrective rhinoplasty is still one of the most challenging structural reconstructions in this population. Alloplastic materials, especially silicone, are often preferred for augmentation owing to their low cost, intraoperative contouring simplicity, as well as the lack of donor site morbidity associated with autologous grafts. But despite the surgical benefits, alloplastic material present intrinsic risks.
**Case report:** The case report discusses the clinical trajectory of two women aged 23 and 26 who presented with previous unilateral CLP. The patients were both previously surgically corrected for cleft defects and underwent augmentation rhinoplasty with L-bar silicone implants related to residual nasal asymmetry. The immediate postoperative recovery periods were uneventful but both patients developed delayed-onset implant infections. These complications emerged at 6 months and 1 year after operation, respectively, calling for additional clinical management.
**Conclusion:** The implications of this data are that silicone implants provide unique advantages in CLP rhinoplasty (cost effective, structural pliability, and decreased surgical morbidity) and surgeons need to maintain a high index of suspicion on late term complications. These cases suggest the risk of delayed post-operative infections with alloplastic materials and underscore the need for an ongoing follow-up of patient care, precision in sterile technique, and an adequate risk benefit assessment in the cleft population with alloplastic versus autologous augmentation strategies.

**2FA26/1121—Cleft Lip and Palate**
**Patient-Specific Custom Arch Bar (PCAB) for Stabilization of Osteotomised Premaxilla with Simultaneous Alveolar Bone Grafting in Bilateral Complete Cleft Lip, Palate, and Alveolus**
Mohamad Shahrin Shahrul Zaman ^1^ and Muhamad Fadzlyazwad Kaider ^1^
*^1^* *Hospital Sultan Ismail Petra, Kuala Krai, Malaysia*
**Objective:** To evaluate the effectiveness of a patient-specific custom arch bar (PCAB) in stabilizing an osteotomised premaxilla during simultaneous premaxillary osteotomy and alveolar bone grafting in a patient with bilateral complete cleft lip, palate, and alveolus.
**Materials and Methods:** A 12-year-old girl with repaired bilateral cleft lip and palate presented with an unrepaired bilateral alveolar cleft and severely protuberant, supra-occluded, and mobile premaxilla. Preoperative cone-beam computed tomography and study model analysis were performed to determine the planned premaxillary repositioning. A patient-specific custom arch bar fabricated from cobalt-chrome was designed to guide and stabilize the repositioned premaxilla. Simultaneous premaxillary osteotomy and bilateral alveolar bone grafting were performed using an autogenous iliac crest bone graft. The osteotomised premaxilla was repositioned posteriorly according to the planned setback and immobilized using the PCAB. Postoperative assessment included clinical evaluation and cone-beam computed tomography imaging.
**Results:** The PCAB provided stable immobilization of the osteotomised premaxilla throughout the healing phase. Clinical follow-up demonstrated satisfactory soft tissue healing, with maintenance of premaxillary vitality and stability. Two-month postoperative cone-beam computed tomography imaging showed satisfactory bony incorporation at both alveolar cleft sites. No evidence of premaxillary mobility, wound dehiscence, graft failure, or avascular necrosis was observed. The custom arch bar was removed after confirmation of adequate healing, with the premaxilla remaining firm and well positioned.
**Conclusion:** Simultaneous premaxillary osteotomy and alveolar bone grafting can be successfully performed in selected bilateral cleft cases with a protrusive mobile premaxilla. The use of a patient-specific custom arch bar provides reliable stabilization, facilitates accurate repositioning, and supports favourable bone healing while avoiding transosseous fixation. This technique represents a useful adjunctive method in the management of complex bilateral alveolar cleft deformities.

**2FA26/1143—Cleft Lip and Palate**
**Surgical Techniques for Correcting Vermillion Deformities in Secondary Cleft Lip Repair**
Toshiaki Numajiri ^1^
*^1^* *Kyoto Prefectural University of Medicine, Department of Plastic and Reconstructive Surgery, Kyoto, Japan*
**Objectives**: Among postoperative deformities of cleft lip, vermillion deformities include notch, volume deficiency, and exposure of the wet vermillion. For vermillion notch and volume deficiency, we employ Z-plasty or transposition flaps from the affected vermillion. To determine the efficacy of these techniques in improving cosmetic appearance, a postoperative survey was conducted.
**Materials and Methods**: This is a retrospective observational study. Subjects comprised cleft lip patients undergoing secondary lip revision at our institution between 2017 and 2025. Patient age, gender, cleft side, cleft pattern, surgical technique, and postoperative assessment were retrieved from medical records. Postoperative assessment involved two specialists evaluating the outcome using photographs taken at least 6 months postoperatively, using a four-point scale (Excellent, Good, Fair, Poor).
**Results**: The cohort comprised 39 patients aged 5–23 years (male: n = 24; female: n = 15). The clefts were unilateral (n = 35) or bilateral (n = 4). Surgical techniques included Z-plasty (n = 34), unilateral transposition flap (n = 28), bilateral transposition flap (n = 4), simple suture closure (n = 2), and Abbe procedure (n = 1). Postoperative assessments were Excellent (n = 11), Good (n = 20), Fair (n = 5), Poor (n = 3).
**Conclusion**: Z-plasty and unilateral transposition flap techniques were the most frequently employed surgical methods, yielding good or better postoperative results in approximately 80% of cases. However, some cases exhibited noticeable volume deficiency in the vermillion, suggesting that alternative techniques may be required in such instances. Z-plasty and transposition flap techniques were judged to be effective for correcting deformities of the vermillion.

**2FA26/1168—Cleft Lip and Palate**
**Does Preoperative Nasal Severity Predict Postoperative Nasal Aesthetics?**
Shaffira Herawati ^1^, Fitriana Fitriana ^2^, Ken Ayu Miranthy ^1^, Nur Aini ^1^ and Muhammad Syafrudin Hak ^1^
*^1^* *Woman and Children Harapan Kita Hospital, Jakarta, Indonesia*
*^2^* *University of Brawijaya, Malang, Indonesia*
**Objectives**: To evaluate the correlation between preoperative nasal deformity and postoperative nasal aesthetic outcome in unilateral cleft lip patients, using subjective and objective assessment methods, and to determine if different postoperative assessment methods correlate with each other.
**Materials and Methods**: A retrospective analysis was conducted in a cleft center using preoperative and postoperative clinical photographs of 32 patients with non-syndromic left-sided unilateral cleft lip. Preoperative nasal severity was assessed using the clock diagram. Postoperative nasal aesthetic outcomes at 6 months following primary lip repair were evaluated objectively using the deformity rating scale and subjectively using the cleft aesthetic rating scale. Inter-observer and intra-observer reliability of four raters was evaluated using the intraclass correlation coefficient. Spearman’s rank correlation coefficient was used to assess the relationships between preoperative nasal deformity and subjective postoperative nasal aesthetic outcomes, between preoperative nasal deformity and objective postoperative nasal aesthetic outcomes, and between subjective and objective postoperative measures.
**Results**: The relationship between preoperative nasal deformity and postoperative nasal aesthetic outcome was not significant when assessed using subjective and objective methods (r_s_ [30] = 0.123, *p* = 0.502 and r_s_ [30]= −0.120, *p* = 0.514, respectively). Similarly, the relationship between subjective and objective postoperative assessment methods was also non-significant (r_s_ [30]= −0.115, *p* = 0.530). Postoperative nasal aesthetic outcomes ranged from fair to very good subjectively and from good to excellent objectively, regardless of initial deformity severity.
**Conclusion**: Preoperative nasal deformity did not appear to predict postoperative nasal aesthetic outcome when assessed using either subjective or objective methods. In addition, subjective and objective postoperative assessments were also not significantly associated. These findings suggest that factors beyond initial nasal deformity may contribute to postoperative nasal aesthetic outcomes, potentially including the use of nasoalveolar moulding and nasal conformer as components of the treatment protocol at this center.

**2FA26/1172—Cleft Lip and Palate**
**Three-Dimensional Virtual Surgical Planning and Customized Surgical Guide-Assisted Two-Piece Maxillary Distraction Osteogenesis in a Patient with Cleft Lip and Palate: A Case Report**
Kannika Rungsaeng ^1^, Thongchai Nuntanaranont ^2^, Nita Viwattanatipa ^3^, Somchai Satravaha ^4^, Saran Worasakwutiphong ^5^, Ratchawan Tansalarak ^1^, Sajjaporn Pakanant ^6^ and Thitima Sukkasem ^4^
*^1^* *Faculty of Dentistry, Naresuan University, Phitsanulok, Thailand*
*^2^* *Prince of Songkla University, Hatyai, Thailand*
*^3^* *Faculty of Dentistry, Mahidol University, Bangkok, Thailand*
*^4^* *Thai Association of Orthodontists, Bangkok, Thailand*
*^5^* *Faculty of Medicine, Naresuan University, Phitsanulok, Thailand*
*^6^* *Phra Nakhon Si Ayutthaya Hospital, Phra Nakhon Si Ayutthaya, Thailand*
**Objective**: To describe the application of three-dimensional virtual surgical planning (3D VSP) and customized surgical guide-assisted two-piece maxillary distraction osteogenesis (DO) in the management of severe maxillary hypoplasia in a patient with cleft lip and palate.
**Materials and Methods**: A 15-year-old Thai male with a history of repaired right complete unilateral cleft lip and palate presented with severe maxillary hypoplasia, midface deficiency, and a skeletal Class III relationship. Treatment was planned using 3D virtual surgical planning. Patient-specific CAD/CAM surgical guides were designed to assist with two-piece Le Fort I osteotomy and accurate placement of the distraction devices.
**Results**: Following the latency period of distraction osteogenesis, the distraction phase was initiated at a rate of 1 mm/day and continued for 9 days, resulting in 9 mm of maxillary advancement. After a 10-week consolidation period, the distraction devices were removed, and the maxilla was stabilized with miniplates and screws in the proper position. The planned advancement was achieved, with improvement in occlusal relationship and facial profile.
**Conclusions**: The use of 3D virtual surgical planning in combination with customized surgical guides enabled accurate transfer of the preoperative plan to the operative field and facilitated control of osteotomy design and distraction vectors. This approach may be a useful adjunct in the management of complex cleft-related maxillary deformities requiring distraction osteogenesis.

**2FA26/1173—Cleft Lip and Palate**
**3D Analysis of Maxillary Arch in Cleft Lip and Palate Patients with and Without Feeding Plate**
Raynor Favianozaki ^1^, Ken Ayu Miranthy ^1^, Nur Aini ^1^ and Muhammad Syafrudin Hak ^1^
*^1^* *Woman and Children Harapan Kita Hospital, Jakarta, Indonesia*
**Objectives**: This study aimed to compare maxillary arch dimensions between patients who received a feeding plate therapy and those who did not, and to evaluate dimensional differences in the maxillary arch among different durations of feeding plate usage prior to palatoplasty.
**Materials and Methods**: This retrospective comparative cross-sectional study included 36 non syndromic complete unilateral cleft lip and palate patients with available pre-palatoplasty three-dimensional dental casts gathered from a cleft center. Subjects were categorized into feeding plate and non feeding plate groups for the primary analysis. An exploratory analysis further feeding plate user subjects classified into short-duration (<3 months), moderate-duration (3–6 months), and long-duration (>6 months) groups. Maxillary arch dimensions were assessed using intercanine width, posterior arch width, greater segment width, and lesser segment width. Normality and homogeneity of variance were evaluated using the Shapiro-Wilk and Levene’s tests. Independent samples *t*-test and one-way ANOVA were applied, with statistical significance set at *p* < 0.05.
**Results**: The primary analysis showed no statistically significant differences between feeding plate and non feeding plate groups for intercanine width (*p* = 0.442), posterior arch width (*p* = 0.132), greater segment width (*p* = 0.789), or lesser segment width (*p* = 0.369). Exploratory analysis also revealed no statistically significant differences among groups for intercanine width (*p* = 0.399), posterior arch width (*p* = 0.364), greater segment width (*p* = 0.296), or lesser segment width (*p* = 0.667).
**Conclusion**: Maxillary arch dimensions at the pre-palatoplasty stage did not differ significantly according to feeding plate usage or duration. Within the limitations of this sample, these findings suggest that feeding plate use did not appear to affect maxillary arch dimensions at the pre-palatoplasty stage. Larger longitudinal studies are warranted to further clarify its potential influence on maxillary arch development.

**2FA26/1184—Cleft Lip and Palate**
**CBCT-Based 3D Surgical Guide-Assisted Reconstruction of wide unilateral Maxillary Gnathoschisis Using Mandibular Symphyseal Autograft: An Alternative To Iliac Crest Harvesting—A Case Report**
Ulfah Safitri ^1^, Yosaphat Bayu Rosanto ^1^, Cahya Hasan ^1^
*^1^* *Universitas Gadjah Mada, Yogyakarta, Indonesia*
**Objectives**: Alveolar bone grafting for wide unilateral maxillary gnathoschisis involving the alveolus, palate, and nasal floor is commonly performed using iliac crest grafts because of their abundant cancellous bone volume and high osteogenic potential. However, donor-site morbidity remains a major limitation. Mandibular symphysis autografts have emerged as a promising alternative because of their favorable intramembranous corticocancellous properties, lower morbidity, faster healing, and absence of external scars. This report describes CBCT-based digital planning and 3D surgical guide-assisted reconstruction to improve operative precision, optimize graft adaptation, and support the predictable use of mandibular symphyseal autografts as an alternative to iliac crest grafting in wide unilateral gnathoschisis reconstruction combined with gnatoplasty.
**Materials and Methods**: A 16-year-old male presented with unilateral left maxillary gnathoschisis associated with persistent oronasal communication and nasal regurgitation dduring 1 year of orthodontic treatment. Clinical and CBCT evaluation revealed a wide alveolar defect involving teeth 21–23. CBCT-based 3D digital planning was used to assess defect morphology, estimate graft volume, and design a patient-specific 3D-printed surgical guide for mandibular symphysis graft harvesting and adaptation. Reconstruction was performed using gnatoplasty and piezosurgery-assisted mandibular symphysis bone grafting. The graft was fixed with microplates and screws, augmented with autogenous particulate bone, and covered with an A-PRF membrane to enhance stability and soft-tissue healing.
**Results**: At 6 months postoperatively, CBCT evaluation showed satisfactory graft integration, improved alveolar continuity, and near-normal interdental bone height corresponding to Bergland type I–II. The patient remained free from nasal regurgitation, with no major donor- or recipient-site complications.
**Conclusion**: Guided alveolar bone grafting integrated with CBCT-based digital planning may improve operative precision and support the use of mandibular symphysis autografts as an alternative to iliac crest grafting for wide unilateral gnathoschisis reconstruction with reduced donor-site morbidity.

**2FA26/1193—Cleft Lip and Palate**
**Cleft Lip Palate Patients Asscociated with Multiple Congenital Anomalies at Cleft Center**
Ajeng Puspa ^1^, Fitriana Fitriana ^2^, Ken Ayu Miranthy ^1^, Nur Aini ^1^ and Muhammad Syafrudin Hak ^1^
*^1^* *Woman and Children Harapan Kita Hospital, Jakarta, Indonesia*
*^2^* *Faculty of Dentistry Universitas Brawijaya, Malang, Indonesia*
**Objectives**: Cleft lip and palate (CLP) is one of the most commonly occurring congenital craniofacial anomalies that can be associated with other congenital anomalies. This study aims to evaluate the incidence and distribution of associated congenital anomalies among patients with CLP treated at the Cleft Center.
**Materials and Methods**: This retrospective descriptive study was conducted through analysis of the medical records of patients with CLP treated at the Cleft Center between 2019 and 2025.
**Results**: In 532 patients of CLP, 103 (19.4%) patients were found to have other congenital anomalies, including 16 syndromes, the most common being Pierre Robin sequence (5 cases, 4.9%). The commonest congenital anomaly seen was of the craniofacial type, namely microcephaly (38 cases, 36.9%). The most common cardiac anomaly was atrial septal defect (26 cases, 25.2%). The most common musculoskeletal anomaly was congenital talipes equinovarus (12 cases, 11.7%). Otorhinolaryngological anomalies were common in the form of laryngomalacia (10 cases, 9.7%). Bilateral subependymal cysts were the most common neurological abnormality (6 cases; 5.8%). Other abnormalities include umbilical hernia (4.9%), undescended testis (3.9%), microphthalmia (1.9%), and hematological conditions (1%).
**Conclusion**: CLP is frequently associated with a variety of congenital anomalies. Comprehensive evaluation and multidisciplinary management are essential to optimize patient outcomes.

**2FA26/1208—Cleft Lip and Palate**
**The Right Alignment: Reimagining the Premaxillary Setback**
Siti Fatihah Ramli ^1^, Nor Anis Razali ^1^ and Mohd Rosli Yahya ^2^
*^1^* *Hospital Raja Perempuan Zainab 2, Kota Bharu, Malaysia*
*^2^* *Hospital Umum Sarawak, Kuching, Malaysia*
**Objectives**: Cleft of lip and palate are most common serial congenital anomalies to affect the orofacial region. It can occur isolated or together in various combination and/or along with other congenital deformities particularly congenital heart diseases. Complete bilateral CLP (BCLP) is the most severe of the common orofacial cleft subtypes. Patient with orofacial cleft deformity needs to be treated at right time and at right age to achieve functional and aesthetic wellbeing.
**Materials and Methods**: A case of 13 years old Malay boy, medically fit, known case of bilateral cleft lip and palate presented to our department with hypoplastic mobile premaxilla. Cheiloplasty and palatoplasty was done previously at another centre. Patient defaulted all multidisciplinary follow ups due to family matter and was referred for management of cleft alveolus and dental caries. Extraoral examination revealed class III skeletal pattern and depressed nasal bridge with scar at philtrum region. Intraoral examination revealed mobile hypoplastic premaxilla with presence of peg lateral tooth, and bilateral cleft alveolus.
**Results**: Patient underwent alveolar bone graft harvested from right iliac crest and premaxilla was osteotomised and stabilized using customised plate and pre band wire.
**Conclusion**: In patients with bilateral cleft lip and palate (BCLP), the alveolar clefts lead to premaxilla mobility, with the premaxilla articulating superiorly to the vomer bone results in protrusion of the premaxilla due to the absence of the sphincter function of the orbicularis oris muscle. The standard of care involves early presurgical orthopaedics (nasoalveolar moulding), stabilization using fixed appliances, surgical osteotomy (repositioning), and alveolar bone grafting to fuse the segments into a functional arch. Surgical repositioning is achieved by centralizing the segment to reduce severe overjet, allowing for tension-free lip closure, facilitating concurrent alveolar bone grafting, and creating a stable, functional foundation for normal speech, breathing, and mastication.

**FA26/52—Craniofacial Surgery**
**Orbital Wall Fracture Reconstruction Using Conchal Cartilage Graft**
Erwin Manipol ^1^, Mark Jansen Austria ^1^ and Angela Marie Jao ^1^
*^1^* *Amang Rodqiguez Memorial Medical Center, Marikina, Philippines*
**Objectives**: To present the clinical application of autologous auricular conchal cartilage graft for orbital floor reconstruction in complex craniofacial trauma, and to evaluate its effectiveness as a practical alternative to alloplastic materials in resource-constrained settings.
**Materials and Methods**: We report a case of a 26-year-old male who sustained extensive maxillofacial injuries following a motor vehicular accident. Clinical examination and craniofacial computed tomography revealed Le Fort I–III fractures, naso-orbito-ethmoid injury, and an orbital floor defect with herniation of intraorbital contents. Definitive management involved open reduction and internal fixation using titanium plates, combined with orbital floor reconstruction utilizing autologous auricular conchal cartilage. Surgical technique, perioperative considerations, and postoperative outcomes were systematically evaluated.
**Results**: The conchal cartilage graft demonstrated excellent adaptability to the orbital floor defect, providing stable support and adequate restoration of orbital volume. There was successful reduction of herniated orbital contents with preservation of extraocular muscle function and absence of diplopia or visual impairment. No intraoperative or postoperative complications, including infection, graft resorption, or donor site morbidity, were observed. Aesthetic outcomes were satisfactory, with improved facial symmetry.
**Conclusion**: Autologous auricular conchal cartilage graft offers a reliable, low-cost, and biologically compatible option for orbital floor reconstruction. Its inherent curvature and ease of harvest make it particularly advantageous in complex fractures where access to synthetic implants is limited. This case supports its broader applicability in craniofacial trauma surgery and highlights the importance of adaptable, patient-centered reconstructive strategies in achieving optimal functional and aesthetic outcomes.

**FA26/60—Craniofacial Surgery**
**Growth-Anticipatory Overcorrection in Syndromic Unicoronal Craniosynostosis (Muenke Syndrome)**
Nurfarah Nadhira Fadhil ^1^, Hamidah Mohd Zainal ^1^ and Mohammad Ali Mat Zain ^1^
*^1^* *Hospital Wanita & Kanak-Kanak Kuala Lumpur, Hospital Kuala Lumpur, Kuala Lumpur, Malaysia*
**Objectives**: To describe the application of growth-anticipatory overcorrection during fronto-orbital advancement (FOA) in a child with Muenke syndrome and to evaluate immediate postoperative craniofacial outcomes.
**Materials and Methods**: A 2-year-old female presented with frontal flattening, orbital retrusion, anterior plagiocephaly, and Harlequin deformity consistent with right unicoronal synostosis. Genetic testing confirmed an FGFR3 mutation characteristic of Muenke syndrome. Three-dimensional computed tomography demonstrated right coronal suture fusion and cranio-orbital asymmetry. FOA with bandeau remodeling asymmetrical advancement was performed incorporating a deliberate overcorrection towards the synostotic side. Surgical planning was guided by the known persistence of FGFR3-mediated osteogenic activity and anticipated postoperative growth drift.
**Results**: Immediate clinical assessment postoperatively demonstrated controlled overcorrection achieved on the affected side. The asymmetrical advancement did not result in excessive soft-tissue tension or orbital compromise. No significant perioperative complications were observed. Subsequent radiological and clinic follow-up demonstrated stable craniofacial reconstruction with improved resultant frontal contour and orbital symmetry. The patient maintains normal function, with no deficits observed.
**Conclusion**: This case highlights the biological limits of symmetry-based craniofacial reconstruction in syndromic craniosynostosis. While FOA effectively restores immediate cranial contour, the dysregulated FGFR3 signaling intrinsic to Muenke syndrome continues to influence osteogenesis and cranial growth postoperatively. The adaptation of a growth-compensated overcorrection strategy proves effective in achieving improved craniofacial balance by proactively accounting for pathological growth patterns. Recognizing this dynamic helps plastic surgeons move from *restoration of form* to *anticipation of growth*.

**FA26/114—Craniofacial Surgery**
**Sphenomandibular Ligament Ossification Following Dental Extraction: A Case Report & Literature Review**
S. Hemanth Kumar ^1^, Uma Shanker Pal ^1^, Ezhilarasi Sundaram ^1^ and Tanvi Vinarkar ^1^
*^1^* *King George’s Medical University, Lucknow, India*
**Objectives**: The sphenomandibular ligament (SML) is a thin accessory ligament of the temporomandibular joint. Pathologies of the SML are rare but can lead to severe functional deficits. This report describes an exceedingly rare case of recurrent SML ossification following a routine dental extraction and literature review.
**Materials and Methods**: A 29-year-old female presented with progressive trismus (maximal interincisal opening of 3 mm) one year after a mandibular right third molar extraction. Despite previous surgical and conservative interventions elsewhere, the restriction persisted. Computed tomography (CT) was utilized for diagnostic imaging. Surgical management involved a radical excision of the ossified mass via a submandibular approach, followed by reconstruction with an alloplastic plate and an aggressive physiotherapy protocol.
**Results**: CT imaging revealed a well-defined bony bridge extending from the medial mandibular ramus to the skull base, following the anatomical path of the SML. Intraoperatively, the ossified ligament was successfully excised. Immediate postoperative mouth opening improved from 3 mm to 36 mm. At the 6-month follow-up, the patient maintained a stable and functional mouth opening of 38 mm with no signs of further recurrence.
**Conclusion**: SML ossification is a rare but critical differential diagnosis for progressive, post-extraction trismus. In cases of recurrence, radical surgical excision combined with alloplastic reconstruction and early physiotherapy yields superior functional outcomes. Clinicians should maintain a high index of suspicion when conventional trismus treatments fail.

**FA26/257—Craniofacial Surgery**
**An Alternative Strategy for Pediatric Traumatic Frontal Cranioplasty Using u-HA/PLLA Composite: A Case with Favorable Cosmetic Outcome**
Han Ping Hsu ^1^
*^1^* *Doctoral Program of Clinical and Experimental Medicine, College of Medicine, National Sun Yat-sen University, Chi Mei Medical Center, Division of Plastic and Reconstructive Surgery, Department of Surgery, Kaohsiung, Taiwan*
**Objectives**: Pediatric cranioplasty presents unique challenges due to ongoing cranial growth and limitations of conventional reconstructive materials. This study presents a decision-driven application of a bioresorbable unsintered hydroxyapatite/poly-L-lactic acid (u-HA/PLLA) composite as an alternative strategy for reconstruction in a traumatic cranial defect.
**Materials and Methods**: An 11-year-old girl presented with a traumatic frontal bone defect characterized by a sizable and irregular calvarial loss. Considering the patient’s age, defect morphology, and the limitations of conventional options such as titanium, PEEK, and autologous bone grafting, a bioactive resorbable u-HA/PLLA composite system was selected to avoid permanent implantation. The reconstructive strategy aimed to restore contour while accommodating future cranial growth in a developing skull.
**Results**: Postoperatively, the patient demonstrated a favorable cosmetic outcome, with restoration of a smooth frontal contour and satisfactory symmetry. No complications, including infection, implant exposure, or contour deformity, were observed during follow-up. Postoperative radiologic evaluation was not available; however, the external contour remained clinically stable with satisfactory symmetry.
**Conclusion**: This case highlights a decision-driven use of u-HA/PLLA composite in pediatric cranial reconstruction. In selected cases involving growing skulls and irregular traumatic defects, bioresorbable and osteoconductive materials may provide a reasonable alternative to permanent implants. Further studies are required to evaluate long-term structural outcomes and bone regeneration.

**FA26/265—Craniofacial Surgery**
**Rhinoplasty in Adult Crouzon Syndrome: Adaptation to Intraoperative Cerebrospinal Fluid Leak**
Ujwal Komatla ^1^, Shivamurthy K C ^1^ and Karthik G B ^1^
*^1^* *S.S. Institute of Medical Sciences and Research Centre, Davangere, India*
**Objectives**: Crouzon syndrome is characterized by craniofacial dysostosis with hypertelorism, proptosis, midface retrusion, and nasal deformities, typically managed through staged interventions. We report a case of tip-focused rhinoplasty in an untreated adult with Crouzon syndrome, modified intraoperatively due to a cerebrospinal fluid (CSF) leak.
**Materials and Methods**: A 22-year-old female with untreated Crouzon syndrome presented with hypertelorism, shallow orbits, midface hypoplasia, and nasal deformity, including a bulbous tip and alar flaring. Radiological evaluation revealed a copper-beaten skull pattern with fused cranial sutures. Anthropometric analysis showed an intercanthal distance of 42 mm and a nasal tip projection ratio of 0.48. Open rhinoplasty was planned via a trans columellar approach under general anesthesia. During attempted lateral osteotomy, an intraoperative CSF leak was encountered, leading to abandonment of bony work. The procedure was limited to tip refinement, including cephalic trim, domal suturing, placement of a columellar strut using septal cartilage, and alar base reduction.
**Results**: At 12-month follow-up, the patient demonstrated improved nasal tip definition, with an increase in projection ratio to 0.60. Patient-reported aesthetic satisfaction improved from 3/10 preoperatively to 7.5/10 postoperatively. The CSF leak resolved spontaneously without intervention or long-term sequelae. Residual hypertelorism and proptosis persisted but appeared proportionate.
**Conclusion**: Tip-focused rhinoplasty may be a safe and effective option in untreated adult patients with Crouzon syndrome when intraoperative challenges such as CSF leak preclude osteotomy. Careful preoperative imaging and intraoperative vigilance are essential given the altered craniofacial anatomy in syndromic patients.

**FA26/301—Craniofacial Surgery**
**Patient-Specific 3D Printing-Guided Surgical Decompression for Traumatic Superior Orbital Fissure Syndrome: A Case Report**
Loi Phuoc Nguyen ^1^, Chon Thanh Ho Nguyen ^1^, Tuan Van Nguyen ^2^, Chanh Trung Le ^2^ and Jun-Young Kim ^3^
*^1^* *Department of Maxillofacial Surgery, Faculty of Odonto-Stomatology, University of Medicine and Pharmacy, Ho Chi Minh city, Vietnam*
*^2^* *Department of Maxillofacial Surgery, National Hospital of Odonto-Stomatology, Ho Chi Minh, Vietnam*
*^3^* *Department of Oral & Maxillofacial Surgery, Yonsei University College of Dentistry, Seoul, Republic of Korea*
**Background and Objectives**: Traumatic superior orbital fissure syndrome (SOFS) is an uncommon but potentially devastating complication of craniofacial trauma, resulting from injury or compression of the cranial nerves traversing the superior orbital fissure. The optimal management strategy remains controversial, particularly regarding the role and timing of surgical decompression.
**Case presentation**: We report a case of traumatic SOFS caused by direct bony compression following craniofacial trauma. The patient presented with ophthalmoplegia, ptosis, sensory deficit in the ophthalmic division of the trigeminal nerve, and diplopia. Computed tomography demonstrated displaced orbital wall bone fractures adjacent to the superior orbital fissure.
Three-dimensional (3D) reconstruction was performed to precisely identify the compressive bony fragments. A physical 3D-printed model was fabricated, allowing preoperative simulation of the surgical approach and selection of appropriate instruments. Based on these findings, early surgical decompression was undertaken.
**Conclusion:** This case highlights the value of 3D reconstruction and physical model simulation in the diagnosis, surgical planning, and management of traumatic SOFS due to direct bony compression. Early, image-guided surgical decompression may improve neurological outcomes in selected patients.

**FA26/311—Craniofacial Surgery**
**Evaluating the Cognitive Impacts of Surgery in Non-Syndromic Craniosynostosis: A Systematic Review**
Mitchell Fagan ^1^, Georgia Fraulin ^1^, Janice Kung ^2^ and Curtis Budden ^1^
*^1^* *Division of Plastic Surgery, Department of Surgery, University of Alberta, Edmonton, AB, Canada*
*^2^* *Geoffrey & Robyn Sperber Health Sciences Library, University of Alberta, Edmonton, AB, Canada*
**Objectives**: The neurodevelopmental benefit of surgical correction in non-syndromic craniosynostosis (NSC) remains controversial. While surgery is standard for cranial remodeling and prevention of complications such as elevated intracranial pressure, its impact on cognitive outcomes is unclear. This systematic review evaluates whether surgical intervention improves neurocognitive development compared to conservative management.
**Materials and Methods**: A systematic review was conducted in accordance with PRISMA guidelines. MEDLINE, Embase, PubMed, Web of Science, and Cochrane Library were searched (October 2025). Inclusion criteria were studies involving NSC patients with objective neurocognitive assessments and comparisons to either preoperative or non-operative cohorts. Two independent reviewers screened studies and assessed methodological quality using the MINORS criteria.
**Results**: Seventeen studies comprising 1055 patients met inclusion criteria, of whom 78.7% underwent surgery and 21.3% were managed conservatively. The mean age at surgery was 10.6 months, with study follow-up ranging from 9 months to 15 years. Study designs were predominantly prospective, with moderate to high methodological quality. Findings were heterogeneous: 47% of studies reported neurocognitive improvement following surgery, 41% found no significant benefit, and 12% were inconclusive. Variability in outcomes was influenced by study design, craniosynostosis subtype, and assessment tools, most commonly the Bayley Scales of Infant Development. Selection bias, confounding variables (e.g., severity, socioeconomic status), and inconsistent follow-up limited interpretability.
**Conclusion**: Current evidence does not definitively support a neurocognitive benefit of surgery in NSC. Observational data are conflicting and limited by methodological heterogeneity and bias. Future research should prioritize long-term functional outcomes and account for disease severity and subtype to better guide clinical decision-making and family counseling.

**FA26/390—Craniofacial Surgery**
**Deep Cervical Lymphaticovenous Anastomosis for the Treatment of Alzheimer’s Disease: A Review of the Current Evidence and Future Directions**
Nathan Pinnawala ^1^
*^1^* *The Royal Melbourne Hospital, Parkville, Australia*
**Objectives**: The pathophysiology of Alzheimer’s Disease (AD) has traditionally focused on the amyloid cascade hypothesis, which emphasizes the overproduction of amyloid-beta though recent literature shifts the focus toward a failure of clearance mechanisms—the brain’s inability to purge neurotoxic metabolic waste. Three key mechanisms through which central nervous system metabolic waste are the blood brain barrier, glymphatic system and meningeal lymphatic vessels (MLVs). Deep cervical lymphaticovenous anastomosis (dcLVA) has recently emerged as a proposed microsurgical intervention to bypass cervical lymphatic resistance and accelerate the clearance of amyloid-beta (Aβ) and tau proteins in Alzheimer’s disease (AD).
**Materials and Methods**: A literature review prioritized recent retrospective cohorts, systematic reviews, and neuroimaging data evaluating cognitive morbidity following elective lymph node dissection (ELND) in head and neck cancer patients. Primary endpoints included cumulative dementia incidence and risk stratification by surgical extent.
**Results**: Mechanical excision of deep cervical lymph nodes significantly accelerates neurodegeneration. Longitudinal tracking demonstrates a 10.34% cumulative clinical dementia incidence over 8.6 years (0.7 per 100 patient-years) (Chao et al., 2024). A surgical “dosage effect” exists: bilateral comprehensive dissections independently predict Alzheimer’s Disease pathogenesis more than unilateral procedures. Systematic pooling of 28,054 patients (Omar et al., 2026) reveals early cognitive decline in up to 40% of patients via prospective screening. Bilateral ELND combined with adjuvant radiotherapy results in severe functional decline in up to 37% of cohorts, corroborated by DTI-ALPS imaging showing significantly reduced glymphatic clearance.
**Conclusion**: ELND creates an iatrogenic mechanical bottleneck in the central nervous system’s glymphatic clearance axis, trapping amyloid-beta and accelerating Alzheimer’s Disease. Bilateral lymphatic disruption poses a severe neurocognitive threat, necessitating tissue-sparing techniques or super microsurgical reconstructive interventions (e.g., dcLVA) to restore vital glymphatic-venous efflux pathways.

**FA26/399—Craniofacial Surgery**
**Surgical Management of Craniofacial Microsomiawith Temporomandibular Joint Ankylosis**
Rawinkan Cheungsirakulwit ^1^ and Niphon Klai-on ^1^
*^1^* *Department of Oral and Maxillofacial Surgery, Faculty of Dentistry, Khon Kaen University, Khon Kaen, Thailand*
**Objectives**: Craniofacial microsomia (CFM) is a second most common congenital craniofacial deformity characterized by underdevelopment of facial structures. Its estimated prevalence ranges from 1 in 3000 to 1 in 5000 live births. CFM is frequently associated with macrostomia, mandibular hypoplasia and extracraniofacial anomalies. In a pediatric CFM patients with TMJ ankylosis, 62% of ankyloses were congenital.
**Materials and Methods**: A 17-year-old Thai female presented with congenital facial asymmetry with limit mouth opening. The patient shows right lower facial asymmetry, right oral commissure 2 mm lower, right orbital dystopia (5 mm lower). Panoramic radiograph showed a left TMJ mass with the ramus, loss of joint space and absent condylar, and mandibular asymmetry. The diagnosis was CFM with left TMJ ankylosis.
**Results**: The surgical goals are to establish a functional joint, restore mandibular length and symmetry, achieve a stable occlusion, and improve overall facial balance. The patient underwent Le Fort I osteotomy and BSSRO, with left TMJ gap arthroplasty and interpositional arthroplasty using a temporoparietal fascia (TPF). Intraoperatively after arthroplasty, the patient achieved a maximum interincisal opening of 32 mm with skeletal class I occlusal relationship as virtual surgical planing. Postoperatively, the skeletal was corrected to Class I and with regular follow-up and aggressive TMJ physiotherapy the patient has maintained a 35 mm maximum interincisal opening, indicating a stable functional result.
**Conclusion**: Management of CFM with TMJ ankylosis, Orthognathic surgery combined with gap arthroplasty and interpositional arthroplasty is an alternative treatment of CFM with TMJ ankylosis. Although combined surgery requires longer operative time and may increase blood loss, it allows simultaneous release of limited mouth opening, which otherwise impedes orthognathic correction, and definitive restoration of jaw relationship in a single stage. Careful virtual surgical planning and perform by an experienced surgeon are essential to optimize accuracy and lead to a successful outcome.

**FA26/402—Craniofacial Surgery**
**An Alternative Approach for Managing Frontoethmoidal Encephalocele**
Ditspol Somsirivatana ^1^
*^1^* *Faculty of Dentistry Khon Kaen University, Amphoe Mueang Khon Kaen, Thailand*
**Objectives**: Frontoethmoidal Encephalocele is a rare congenital anomaly, where part of meninges, cerebrospinal fluid (CSF), herniates through a skull. The general clinical features include a prenasal mass with or without anatomical distortion, orbital dystopia, telecanthus, and long nose. The goals of surgical correction are to remove the mass, reconstruct skull defect, and restore aesthetic appearance. Conventional management is a two-stage approach involving mass removal followed by reconstruction of the defect. This report aim to highlight the one-stage surgical approch using “Chula Technique”.
**Materials and Methods**: A 44-year-old male presents with a 5 cm. prenasal mass with an intercanthal distance of 45 mm. Normal visual acuity. MDCT reveals frontoethmoidal encephalocele (nasoorbital & nasoethmoidal type) causing frontal & nasal bone defect without abnormal brain parenchyma.
**Results**: The patient underwent surgical correction with “Chula Technique” under GA. A facial skin incision was made above the mass followed by mass removal and correction of dural defect. A piece of frontal bone was removed via bicoronal incision. The central part of the frontal bone was cut and reshaped into T-shape. The reconstructed T-shape was then replace. The patients was discharge 7 days after operation. No prenasal mass is detected; however, a surgical scar is present. The intercanthal distance has decreased to 42 mm. Neurological symptoms are unremarkable. A long-term follow-up has been scheduled.
**Conclusion**: One-stage surgical correction using the ‘Chula Technique’ is an optional procedure for the management of frontoethmoidal encephalocele. This technique offers several advantages over the conventional two-stage approach, including a reduced number of procedures, avoidance of frontal craniotomy, shorter operative time, decreased intraoperative complications, reduced hospital stay, and accelerated postoperative recovery. However, it requires the technical proficiency and experience of the surgeon, especially one who is familiar with this technique.

**2FA26/1111—Craniofacial Surgery**
**The Hybrid Planning Concept: A Streamlined In-House VSP Workflow for Paediatric Mandibular Distraction**
Jonathan Rengarajoo ^1^, Lee Chee Wei ^1^ and Kok Tuck Choon ^1^
*^1^* *Hospital Kuala Lumpur, Kuala Lumpur, Malaysia*
**Objectives**: This study evaluates the efficiency, precision, and clinical efficacy of an in-house virtual surgical planning (VSP) workflow centred on a unified hybrid planning concept. We sought to determine whether a single, digitally integrated workflow could successfully consolidate two traditionally distinct surgical phases—osteotomy execution and distractor device positioning—into a single physical mechanism to maximize vector accuracy and minimize operative time.
**Materials and Methods**: A review was conducted on three paediatric patients with mandibular hypoplasia who underwent mandibular distraction osteogenesis (MDO). Utilizing an in-house hybrid planning architecture, pre-operative CT datasets were processed in Meshmixer and 3D Slicer to simultaneously map osteotomy planes and pre-register the distractor hardware configuration within a single coordinate matrix. From this unified virtual blueprint, a custom hybrid guide—engineered to feature both the precise cutting guide slot and the distractor footprint screw holes—was 3D-printed in-house using biocompatible resin. Primary outcomes included design-to-print turnaround time, operative time, and vector accuracy via post-operative CT surface-mesh registration.
**Results**: The in-house hybrid planning workflow yielded a design-to-print turnaround of 2 days. By translating the dual-concept virtual plan into a single physical tool, the hybrid guide streamlined the intraoperative transition from osteotomy to hardware fixation without guide swapping, resulting in a mean operative time of 150 min. Post-operative imaging confirmed high anatomical fidelity to the virtual blueprint.
**Conclusion**: Embracing an in-house VSP workflow built on a hybrid planning concept provides a highly precise, streamlined paradigm for paediatric MDO. By digitally consolidating independent surgical variables into a single physical blueprint, this approach eliminates sequential transfer errors, optimizes theatre efficiency, and maintains strict fidelity to the intended distraction vector.

**2FA26/1206—Craniofacial Surgery**
**Massive Ameloblastoma of the Mandible: A Case Report**
Krishna Vasaiya ^1^, Hari ram ^1^ and Saniya zia ^1^
*^1^* *King George’s Medical University, Lucknow, India*
**Objectives**: To document and analyze a clinically exceptional case of a massive mandibular ameloblastoma extending from angle to angle, highlighting the severe facial disfigurement, functional impairment (dysphagia and compromised mastication), and profound psychosocial impact associated with delayed surgical intervention.
**Materials and Methods**: A 25-year-old female presented to the Department of Oral and Maxillofacial Surgery with gross facial deformity and asymmetry. The patient reported a progressive 1.5-year history of worsening symptoms, including severe difficulty in swallowing, impaired mastication, and restricted social interaction due to the aesthetic morbidity of the lesion.
**Results**: An incisional biopsy performed two years prior for a localized swelling at the left mandibular angle had confirmed an ameloblastoma. Although immediate surgical intervention was advised, the patient initially defaulted on treatment. Over the subsequent two years, the tumor exhibited extensive, locally aggressive growth, expanding across the entire mandibular body from angle to angle.
The patient subsequently underwent definitive treatment under general anesthesia consisting of a wide local excision. Immediate microvascular reconstruction was performed using a vascularized free fibula flap, rigidly stabilized with a 2.5 mm reconstruction titanium profile plate to restore facial height, contour, and continuity.
**Conclusion**: This case serves as a stark reminder that despite being histologically benign, ameloblastomas are locally destructive, relentlessly expansile tumors. Delayed treatment can transform a manageable localized lesion into a massive, debilitating pathology that completely decimates mandibular architecture. Timely surgical intervention is critical to avert severe functional and psychosocial mutilation and to mitigate the complexities of extensive multi-segment microvascular reconstruction.

**FA26/286—Dental Rehabilitation**
**PSI for Simultaneous Reconstruction and Dental Rehabilitation of Difficult Cases of Complex Jaw Defects**
Giacomo De Riu ^1^, Andrea Biglio ^1^, Luigi Vaira ^1^ and Enrica Ligas ^1^
*^1^* *University Hospital of Sassari, University of Sassari, Sassari, Italy*
**Objectives**: Reconstruction and dental rehabilitation of complex jaw defects remain highly challenging when severe bone loss, scarred soft tissues, previous surgeries, or systemic conditions limit the use of bone grafts and standard endosseous implants. The aim of this study is to evaluate the role of **patient-specific implants (PSI)** for simultaneous reconstruction and fixed dental rehabilitation in difficult maxillary and mandibular defects.
**Materials and Methods**: Custom-made subperiosteal titanium implants were designed using high-resolution CT imaging, virtual surgical planning, and laser sintering technology. The implants were anatomically adapted to the residual skeletal structures and provided fixation points together with prosthetic abutments for fixed restorations. Clinical indications included bilateral cleft lip-maxillo-palatal deformities with large alveolar defects and scarred flaps, total maxillectomy after advanced maxillary tumors, complete mandibular atrophy, mandibular resections for medication-related osteonecrosis, and extreme jaw atrophies. This approach avoided the need for bone grafting, microvascular bone flaps, or conventional endosseous implants.
**Results**: PSI placement allowed simultaneous restoration of skeletal support, soft tissue projection, and immediate or early fixed dental rehabilitation. Functional outcomes included rapid recovery of mastication, speech, and oral competence, with improved facial aesthetics. The personalized design facilitated implant stability even in cases with limited residual bone and compromised soft tissues. Surgical morbidity and overall treatment time were reduced compared with traditional staged reconstructive protocols. Particularly favorable benefits were observed in previously operated, irradiated, or medically fragile patients.
**Conclusion**: Patient-specific subperiosteal implants represent an effective and innovative solution for complex jaw defects that are difficult to treat with conventional techniques. By combining reconstruction and prosthetic rehabilitation in a single stage, PSI may significantly improve patient quality of life while reducing morbidity and treatment duration. This method may represent a paradigm shift in the management of severe maxillary and mandibular defects. Further long-term studies are needed to confirm durability and prosthetic success.

**FA26/388—Dental Rehabilitation**
**Rehabilitation of Fibula-Reconstructed Mandible Using Patient-Specific Implants: A Digital-Surgical Approach**
Pravish Vishnudas ^1^, Arjun Krishnadas ^2^, Manoj B ^3^, Sangeetha Nayak ^3^ and Lin Jacob ^1^
*^1^* *Sree Anjaneya Institute of Dental Sciences, Kozhikode, India*
*^2^* *Amrita Institute of Medical Sciences, Kochi, India*
*^3^* *Malabar Hospitals, Kozhikode, India*
**Objectives**: To evaluate the clinical applicability, surgical predictability, and prosthetic outcomes of patient-specific implants (PSIs) in fibula-reconstructed mandibles. Conventional implants are often limited by the altered geometry and volume of fibula grafts, presence of fixation pates and screws resulting in compromised positioning and load distribution. This report highlights the role of a prosthetically driven digital workflow—integrating CBCT, virtual surgical planning, and guided surgery—in enabling anatomically adaptive implant placement and improved functional outcomes, including patient-reported quality of life.
**Materials and Methods**: A patient with a segmental mandibular defect reconstructed using a fibula free flap presented with compromised ridge anatomy. Clinical and radiographic evaluation, including CBCT, was performed. A fully digital workflow was employed involving virtual surgical planning, prosthetically driven implant positioning, and CAD-CAM fabrication of patient-specific implants. Guided surgical placement ensured precise adaptation to the reconstructed mandible. Following healing, a digital workflow with intraoral scans and CAD-CAM fabricated screw-retained fixed prosthesis was delivered.
**Results**: PSIs enabled optimal implant positioning despite anatomical limitations, achieving primary stability and favorable load distribution. The digital workflow improved surgical precision and reduced intraoperative adjustments. Healing was uneventful with successful osseointegration. Functional outcomes followed a fixed screw retained prosthesis included improved mastication, speech, and facial aesthetics. Patient-reported outcomes indicated significant enhancement in quality of life.
**Conclusion**: Patient-specific implants combined with a digital-surgical workflow provide a predictable and clinically significant solution for rehabilitation of fibula-reconstructed mandibles. This approach overcomes anatomical constraints, enhances prosthetically driven outcomes, and improves overall function and patient satisfaction, supporting its role in advanced reconstructive implantology. The digital workflow significantly helped in seamless restoration of patient occlusal rehabilitation.

**FA26/434—Dental Rehabilitation**
**Digital Minds, Restored Smiles: The AI TDriven Post-Cancer Dental Rehabilitation**
Hannah Arnantha ^1^ and Hitesh Bansal ^2^
*^1^* *St George’s, Epsom and St Helier Hospital Group, London, UK*
*^2^* *London North West University Healthcare NHS Trust, London, UK*
**Objectives**: To evaluate and synthesize current evidence on the application of artificial intelligence (AI) in dental rehabilitation following treatment for head and neck oncology patients, with a focus on improving functional, aesthetic, and patient-centered outcomes.
**Materials and Methods**: To evaluate and synthesize current evidence on the application of artificial intelligence (AI) in dental rehabilitation following treatment for head and neck oncology patients, with a focus on improving functional, aesthetic, and patient-centered outcomes.
**Results**: The reviewed literature demonstrates growing integration of AI technologies in post-oncologic dental rehabilitation. Machine learning algorithms have shown utility in treatment planning, particularly in implant placement and prosthetic design, by enhancing precision and reducing clinician-dependent variability. AI-driven image analysis improves detection of anatomical changes following surgery or radiotherapy, aiding in customized rehabilitation strategies. Predictive models have also been developed to assess treatment success and complications, such as osteoradionecrosis risk. However, most studies remain preliminary, with limited large-scale clinical validation and heterogeneity in methodologies.
**Conclusion**: AI holds significant promise in advancing dental rehabilitation for head and neck oncology patients by enabling more personalized, efficient, and predictive care. Despite encouraging early findings, further high-quality clinical studies and standardized protocols are needed to validate these technologies and facilitate their integration into routine practice. Collaboration between clinicians, researchers, and data scientists will be essential to fully realize the potential of AI in this complex patient population.

**2FA26/1013—Dental Rehabilitation**
**The Management of Maxillary Hypertrophy Associated with Disoriented Teeth and Ectopia in Sturge–Weber Syndrome: A Case Report**
Adeeb Zoabi ^1^, Mimi Ashqar ^2^ and Samer Srouji ^1^
*^1^* *Galilee Medical Center, Bar-Ilan University, Nahariya, Israel*
*^2^* *Galilee Medical Center, Nahariya, Israel*
**Objectives:** Sturge–Weber syndrome (SWS) is a rare sporadic neurocutaneous disorder characterized by facial capillary malformations, leptomeningeal angiomas, and ocular anomalies. While neurologic manifestations are well-recognized, craniofacial skeletal involvement can result in significant dentofacial deformity. Orthognathic surgery in SWS patients is rarely reported due to the risk of severe hemorrhage. This report presents a complex case of a 23-year-old patient with SWS and right-sided maxillary hypertrophy secondary to a severe premaxillary vascular malformation.
**Materials and Methods:** The patient underwent a Le Fort I-like resection of the maxilla, including the premaxillary vascular malformation and posterior alveolar bone. Management included preoperative selective embolization of the maxillary artery three days prior to surgery. A custom-made surgical guide, designed via Virtual Surgical Planning (VSP), was used to ensure precision. Dental implants were placed immediately following resection for immediate full-arch rehabilitation. The procedure was performed in a multidisciplinary setting.
**Results:** The surgical procedure was successful with minimal blood loss (~200 mL) and no need for transfusion. Postoperative healing was uneventful, and clinical results remained stable at the 2-year follow-up, achieving favourable functional and aesthetic outcomes.
**Conclusion:** Carefully planned resection of maxillary vascular malformations in SWS patients is feasible when combined with vascular mapping, selective embolization, and VSP. This multidisciplinary approach minimizes operative risks and ensures predictable, stable results in treating complex dentofacial deformities.

**2FA26/1166—Dental Rehabilitation**
**From Virtual Articulator to the 4D Avatar: Moving Towards Zero-Adjustment Personalized Occlusion**
Nadia El Mouahid ^1^, Fatimazahra Ghazzar ^1^ and Samira Bellemkhannate ^1^
*^1^ Dental Consultation and Treatment Centre, Ibn Rochd University Hospital, Department of Prosthodontics, Casablanca, Morocco*
**Objectives**: Traditional digital dentistry has long relied on static CAD/CAM inputs and mechanical virtual articulators to replicate mandibular movements. However, these methods often simplify complex, individualized temporomandibular joint (TMJ) dynamics and kinematics, frequently resulting in the need for tedious intraoral occlusal adjustements at the chairside.
This presentation aims to demonstrate the paradigm shift from standar virtual articulators to the implementation of a dynamic «4D Avatar». By integrating real-time mandibular kinematics, facial scanning, and patient-specific biology, we explore the pathway toward achieving a «zero-adjustment» personalized occlusion in complex restorative and prosthetic dentistry.
**Materials and Methods**:
The transition to 4D dentistry leverages a combination of:
  - High-speed jaw tracking devices to record true, patient-specific functional movements.
  - 3D facial and intraoral scans merged via advanced alignment algorithms to create a holistic digital twin.
  - Dynamic collision-detection software that simulates functional chewing cycles and parafunctional movements rather than just standard linear paths.
**Results**: By incorporating real-time functional kinematics from the design phase, the 4D Avatar will allow for the precise prediction of dynamic occlusal interferences before manufacturing. This approach will significantly reduce clinical placement time, minimizes material stress, and enhances patient comfort. Moving toward a zero-adjustment workflow shifts the clinical focus from correcting errors to delivering of personalized and biologically synchronized prostheses.
**Conclusion**: The 4D Avatar represents the next frontier in digital dentistry, moving beyond static geometries into true functional biology.
Embracing this shift allows clinicians and technicians to achieve unprecedented predictability, ultimately redefining the standars of occlusal harmony and patient-centered care.

**2FA26/1175—Dental Rehabilitation**
**Zygomatic Implants as a Lifeline: Restoring Oral Function After Maxillary Resection for Mucormycosis**
Senthilkumar Palanichamy ^1^, Venkadasalapathi Narayanasamy ^2^ and Anandapadmanaban S ^3^
*^1^* *Govt. Rajaji Medical College, Madurai, Maeoris Dental Implants and Esthetic Care Center, Hannah Joseph Hospital, Dindigul, India*
*^2^* *Hannah Joseph Hospital, KGS Scan Center, Maeoris Dental Implants and Esthetic Care Center, Madurai, India*
*^3^* *Govt Theni Medical College and Hospital, Maeoris Dental Implants and Esthetic Care Center, KGS Scan Center, Theni, India*
**Objectives:** To present our clinical experience and outcomes in rehabilitating patients with post-mucormycosis maxillary defects using zygomatic implants, particularly in cases reconstructed with temporalis flaps where conventional implant placement is not feasible.
**Background:** Mucormycosis leaves patients with devastating maxillary defects following hemi-maxillectomy or total maxillectomy. Although temporalis flaps restore soft-tissue continuity, the loss of maxillary bone severely compromises oral function. Patients often struggle with mastication, speech, and social confidence. Traditional dental implants are rarely an option due to insufficient residual bone. Zygomatic implants, anchored in the dense zygomatic bone, offer a powerful alternative—bypassing the compromised maxilla and providing a stable foundation for prosthetic rehabilitation. For many of these patients, this approach becomes a true lifeline, restoring essential functions that profoundly affect daily life.
**Materials and Methods:** A retrospective review was conducted on approximately 30 patients who underwent dental rehabilitation with zygomatic implants after mucormycosis-related maxillary resection. All patients had either hemi-maxillectomy or total maxillectomy defects reconstructed with temporalis flaps. Zygomatic implants were placed using a standard extra-maxillary approach, followed by prosthetic rehabilitation once soft-tissue healing stabilised. Outcomes assessed included implant stability, prosthetic success, functional improvement, patient comfort, and complications.
**Results:** Zygomatic implants demonstrated predictable stability and supported successful prosthetic rehabilitation across all cases. Patients reported significant improvements in chewing, speech clarity, and overall confidence. No major complications, implant failures, or flap-related issues were observed during follow-up.
**Conclusion:** Zygomatic implants provide a reliable, transformative solution for dental rehabilitation in patients with post-mucormycosis maxillary defects. Even in the presence of extensive resection and temporalis flap reconstruction, this approach restores function, aesthetics, and quality of life with consistent, predictable outcomes.

**FA26/53—Oncology**
**Beyond Tumor Removal: Facial Reanimation in Advanced Malignant Parotid Tumors**
Didiek Darmadi Tri Setyo ^1^
*^1^* *Airlangga University, Soetomo General Hospital, State University of Malang, Surabaya, Indonesia*
**Objectives**: To emphasize that in advanced malignant parotid tumors accompanied by severe facial nerve palsy, surgical management must extend beyond oncologic clearance to incorporate planned facial reanimation, aiming to preserve acceptable facial symmetry and ocular protection at rest when dynamic facial function cannot be preserved.
**Materials and Methods**: We report the case of a 42-year-old woman with a right retroauricular parotid mass for one year, associated with progressive pain, fixation, and facial asymmetry over six months. Clinical examination revealed severe facial nerve dysfunction classified as house–brackmann facial nerve grading system grade V, and the tumor was staged as T4aN0M0. Fine-needle aspiration biopsy suggested adenoid cystic carcinoma. Contrast-enhanced CT demonstrated a right parotid soft-tissue mass abutting the distal facial nerve near the stylomastoid foramen, with facial canal widening and bony dehiscence, consistent with perineural spread.
An extended radical parotidectomy was performed, including resection of involved temporal bone, digastric muscle, and sternocleidomastoid muscle. Immediate static facial reanimation using a tensor fascia lata sling combined with lateral canthoplasty was undertaken according to established static reanimation principles.
**Results**: Intraoperative exploration revealed extensive facial nerve and skull base involvement exceeding imaging findings. Complete macroscopic tumor resection was achieved with tumor-free margins on frozen section. Static facial reanimation resulted in satisfactory facial symmetry at rest, complete eye closure without lagophthalmos, restoration of smile contour, and a defined nasolabial fold, consistent with published outcomes. No major early postoperative complications were observed. Final histopathology confirmed adenoid cystic carcinoma with perineural invasion.
**Conclusion**: Severe facial nerve palsy in malignant parotid tumors is a critical indicator of advanced disease. Facial reanimation should be considered an integral component of definitive surgery to maintain acceptable facial appearance and ocular protection at rest while preserving oncologic radicality.

**FA26/125—Oncology**
**Central Giant cell Granuloma in a Patient with Noonan Syndrome: A Case Repot**
Somaiah Al Maqbali ^1^, Sami Al Kindi ^2^ and Abdulaziz Bakathir ^2^
*^1^* *OMSB (Oman medical Speciality Borad), Muscat, Oman*
*^2^* *SQUH (Sultan Qaboos University Hospital), Muscat, Oman*
**Objectives**: Noonan syndrome is a rare genetic condition that affects one of several genes (e.g., PTPN1, SOSO1, RAF1, or KRAS). It affects any gender and as a genetic condition presents from birth. It involves multiple systems. Central giant cell granuloma is rare in Noonan syndrome patients, but occurs more often in patients suffering from Noonan syndrome than in other populations. It occurs as multiple lesions, forming a recognized subtype with phenotypic overlap with Noonan syndrome, called Noonan-like/multiple giant cell lesion. It represents less than 7–10% of benign jaw tumours.
**Materials and Methods**:
Patient Details: A 3-year-old Omani girl
Case description: Noonan syndrome child patient, with the complaint of a bilateral mandibular swelling related to the posterior mandible region, standing for a long time. Clinical examination showed that the swelling of the mandible was more pronounced than that of the left side. There was no purulent discharge or limited mouth opening.
Diagnostic Tools: Serial Cone-beam computed tomography, Incisional biopsy.
Treatment: Steroid injection Therapy
Follow up: 3 years
**Results**: Steroid injection therapy used to treat growing child show significant outcome with reserved tooth bed. Noonan syndrome child with Central giant cell lesion has high recurrent rate even if primary excision or Medication injection therapy show success outcome still there will be high recurrent rate.
**Conclusion**: Noonan syndrome is a lifelong genetic disorder that affects multiple systems in the body. This is challenging for parents to deal with, especially if parents don’t understand the syndrome and are not aware of the difficulty that they will face with it. Civil associations for Noonan syndrome patients exist because of the complexity of this syndrome. Noonan Syndrome Association is a charity association. It is located in the UK. It is interested in supporting families and NS patients. It provides information, oral webinars presentation, support, and awareness.

**FA26/145—Oncology**
**Mucoepidermoid Carcinoma of the Sublingual Gland: A Rare Presentation**
Rita Martins ^1^, Andreia Silva ^1^, Sílvio Fortes ^1^, Júlio Rodrigues ^1^ and Sara Lopes ^1^
*^1^* *Hospital de Braga, Braga, Portugal*
**Objectives**: This report presents a clinical case of mucoepidermoid carcinoma of the sublingual gland in an 89-year-old male patient. Given the uncommon location for this type of tumour, we aim to describe he diagnostic workup, treatment modalities, and prognosis.
**Materials and Methods**: A thorough clinical history was obtained; the patient was unaware of the lesion, making the time of evolution uncertain. Intraoral examination revealed a painless, mobile, fibrous-elastic mass in the anterior left oral floor, with intact overlying mucosa. Evaluation with magnetic resonance imaging suggested a tumor of the left sublingual gland. An incisional biopsy confirmed the diagnosis of mucoepidermoid carcinoma.
**Results**: The left sublingual gland and the lesion were excised under general anesthesia, along with the overlying mucosa, which had developed ulceration by the time of surgery. Histopathological analysis confirmed a low-grade mucoepidermoid carcinoma of the sublingual gland. The patient underwent regular clinical follow-up, without evidence of recurrence, until the time of his death.
**Conclusion**: This case highlights the clinical approach to an insidious and suspicious oral floor mass. Magnetic resonance imaging is the preferred imaging modality for evaluating soft tissue and salivary gland lesions, while biopsy remains the gold standard for diagnosis. Surgical resection alone is the primary treatment for low-grade tumors, whereas high-grade cases may require selective neck dissection and adjuvant radiotherapy. Close follow-up is recommended, as even low-grade tumors carry a risk of metastasis. Although mucoepidermoid carcinoma is a common salivary gland malignancy, its occurrence in the sublingual gland is relatively rare.

**FA26/168—Oncology**
**Pattern and Histopathological Presentation of Oral and Maxillofacial Sarcomas in Nairobi: A Cross-Sectional Study**
Raphael Onsongo ^1^ and Martin Kamau ^1^
*^1^* *The University of Nairobi Dental Hospital, Kenyatta National Hospital, Nairobi, Kenya*
**Objectives**:
BROAD OBJECTIVES To determine the pattern of occurrence of oral and maxillofacial sarcomas and their histopathological presentations over a period of 30 years (January 1994–December 2023) at The Dental School Hospital, University of Nairobi as well as Kenyatta National Hospital.
SPECIFIC OBJECTIVES 1. To determine the demographic characteristics of patients diagnosed with head and neck sarcomas over a 30-year period. 2. To describe the clinicopathological characteristics of head and neck sarcomas over a 30-year period (January 1994–December 2023) at KNH and UONDH.
**Materials and Methods**: A retrospective review of histopathology reports was conducted, focusing on demographic data, tumour subtypes, anatomical sites, and histological classifications.
Findings were compared to previous studies from Kenya and other regions.
**Results**: Kaposi sarcoma (32.2%) was the most common neoplasm, followed by osteosarcoma (27.8%) and fibrosarcoma (15.1%). The age range of patients was 3 months to 93 years, with the highest prevalence in the 21–30 years age group. Females constituted 50.6% of the cases, aligning with some prior studies but differing from others that showed male predominance. Hard tissue sarcomas accounted for 28% of cases, with osteosarcomas being the most frequent, predominantly affecting females and the mandible. Soft tissue sarcomas constituted 72%, with Kaposi sarcoma being the most prevalent. The mandible was the most affected anatomical site (24%), followed by the maxilla (19%) and the palate. Limited subclassification and grading of tumours was noted in 21% and 10% of cases, respectively, likely due to resource constraints.
**Conclusion**: This study highlights the burden and characteristics of neck and craniofacial mesenchymal malignant neoplasms in a resource-limited setting. The findings emphasize the need for improved diagnostic infrastructure and resource allocation to enhance tumour characterization, prognostication, and treatment planning.

**FA26/172—Oncology**
**Trans-Oral Enucleation of a Lingual Schwannoma in an Adolescent: A Case Report**
Bramadita Satya ^1^, Andi Budihardja ^1^ and Sudirman Sudirman ^1^
*^1^* *Siloam, Kota Tangerang, Indonesia*
**Objectives**: Lingual schwannomas are uncommon benign neural tumors. This report presents a case in an adolescent, highlighting clinical features, imaging findings, surgical management, histopathology, and outcome.
**Materials and Methods**: A 16-year-old boy was evaluated for a painless, slowly enlarging submucosal nodule on the right anterolateral tongue. Clinical examination revealed a dome-shaped, well-circumscribed lesion measuring 1–1.5 cm with intact mucosa. Magnetic resonance imaging (MRI) demonstrated a sharply defined mass, isointense to muscle on T1-weighted sequences and homogeneously hyperintense on T2-weighted sequences, without infiltration or lymphadenopathy. The lesion was excised via trans-oral capsular enucleation under general anesthesia, with careful preservation of the lingual and hypoglossal nerves.
**Results**: The tumor was removed completely through a keyhole trans-oral approach with minimal blood loss. Grossly, the specimen measured 1.4 × 1.1 × 1.2 cm, was firm, tan-yellow, and encapsulated. Histology revealed alternating Antoni A and Antoni B areas, nuclear palisading, Verocay bodies, and a thin fibrous capsule. Immunohistochemistry showed diffuse S-100 and SOX10 positivity, confirming Schwann cell origin. Recovery was uneventful, and at six months the patient had normal speech, intact tongue mobility and sensation, and no evidence of recurrence.
**Conclusion**: Schwannoma should be considered in the differential diagnosis of painless, well-demarcated lingual masses, particularly when MRI demonstrates T1 isointensity and homogeneous T2 hyperintensity. Preoperative MRI aids surgical planning, and trans-oral nerve-sparing enucleation allows complete excision while preserving lingual function. Histopathological confirmation remains essential. With meticulous technique, outcomes are excellent, recurrence is rare, and malignant transformation is exceedingly uncommon.

**FA26/178—Oncology**
**Experience TACE as Neoadjuvant Chemotherapy in Locally Advanced Oral Cancer With Out Lymph Node Metastasis Use Single Regiment**
Kiki Rizki ^1^
*^1^* *Surgical Oncology Division Departement of Surgery Hasan Sadikin Hospital/Padjadjaran University, Bandung, Indonesia*
**Objective**: To evaluate the effectiveness and safety of transarterial chemoembolization (TACE) as neoadjuvant chemotherapy in patients with inoperable head and neck cancer without lymph node metastasis.
**Materials and Methods**: We report a case series of 11 patients with head and neck cancer consisting of 7 oral cancers and 4 salivary gland cancers who underwent transarterial chemoembolization (TACE) as neoadjuvant chemotherapy. All patients were diagnosed with inoperable tumors without lymph node metastasis.
TACE was performed using a single chemotherapeutic agent, doxorubicin, administered at one-third to one-half of the standard systemic dose. Treatment response was evaluated clinically and radiologically. Adverse events were also recorded.
**Results**: Among the 11 patients, 9 patients showed partial response and 2 patients had stable disease after TACE treatment. All patients experienced minimal adverse events.
Patients with partial response subsequently underwent surgical treatment, including wide excision or hemiglossectomy followed by reconstructive surgery. Patients with stable disease were switched to conventional chemotherapy or alternative chemotherapy regimens.
**Conclusion**: Transarterial chemoembolization (TACE) can be considered a potential neoadjuvant treatment option for inoperable head and neck cancer without lymph node metastasis. TACE demonstrated good tumor response with minimal adverse events and allowed previously inoperable tumors to become operable in most patients. Further studies with larger sample sizes are needed to evaluate its efficacy and long-term outcomes.

**FA26/234—Oncology**
**Anxiety Depression Coping Strategies of Newly Diagnosed of Oral Cancer Patients Attending in Selected Tertiary Care Hospitals of Dhaka, Bangladesh**
Mausumi Iqbal ^1^ and Sumaya Mostofa ^2^
*^1^* *Apollo Clinic, JMI Specialized Hospital, Tooth Care, Facio-Maxillary reseach & Development Hub, Dhaka, Bangladesh*
*^2^* *Bangladesh University of Professionals, Tooth Care, Facio-Maxillary reseach & Development hub, Dhaka, Bangladesh*
**Objectives**: The primary aim of the study was to estimate the proportion of anxiety and depression among these patients and identify their coping strategies.
**Materials and Methods**: The cross-sectional study involved 114 participants from DDCH, BSMMU, SHMCH, and DMCH. The Hospital Anxiety and Depression Scale (HADS) was used to assess anxiety and depression levels, while the Brief COPE inventory evaluated coping strategies.
**Results**: The study showed high levels of psychological distress among the patients, with 37.7% experiencing borderline anxiety and 39.5% experiencing abnormal anxiety. Regarding depression, 34.2% of participants showed borderline depression, while 33.3% were classified as having abnormal depression. Common coping strategies included informational support, emotional support, active coping, acceptance, and self-distraction. Sociodemographic factors, such as joint families and rural residents, also influenced these strategies
**Conclusion**: The study highlights the need for comprehensive mental health support in cancer care, tailored to the cultural and socio-economic context of Bangladesh.

**FA26/242—Oncology**
**A Rare Case of Peripheral Giant Cell Granuloma Converting into Rhabdomyosarcoma of the Maxilla**
Krishna Vasaiya ^1^, Hari ram ^1^ and Saniya zia ^1^
*^1^* *King George’s Medical University, Lucknow, India*
**Objectives**: To document and analyze an exceptionally rare clinical occurrence of Peripheral Giant Cell Granuloma (PGCG) appearing to undergo malignant transformation—or being masked by—Rhabdomyosarcoma in the maxillary region of a 37-year-old female at King George’s Medical College.
**Materials and Methods**: A 37-year-old female presented to the Department of Oral and Maxillofacial Surgery with a proliferative growth in the anterior maxillary region. The patient reported a 4 to 5-month history of progressive symptoms, including:
Acute difficulty in breathing and swallowing.
Impaired mastication (chewing).
Inability to achieve complete mouth closure.
**Results**: Initial Diagnosis: A previous biopsy suggested PGCG. Based on this, conservative therapy was initiated using weekly triamcinolone injections to reduce the lesion’s size.
Treatment Failure: The lesion showed no response to steroid therapy. During the planning phase for surgical excision, the patient’s condition worsened, characterized by rapid midface swelling and increased respiratory distress.
Definitive Diagnosis: A subsequent biopsy and histopathological examination revealed features of Rhabdomyosarcoma. This was confirmed via immunohistochemistry, showing strong sensitivity to the Desmin marker.
Management: Following the malignant diagnosis, the patient was transferred to the Medical Oncology Department for systemic management with chemotherapy.
**Conclusion**: This case highlights that while PGCG is traditionally a benign, reactive lesion, clinicians must remain vigilant for aggressive mimics or rare malignant transformations. The failure of conservative corticosteroid treatment and the rapid onset of functional impairment (breathing/swallowing) serve as critical clinical red flags.
The definitive role of histopathology and immunohistochemistry (Desmin marker) is paramount in such cases. A multidisciplinary approach is essential to ensure that aggressive malignancies like Rhabdomyosarcoma are identified early and transitioned from surgical to oncological care promptly.

**FA26/254—Oncology**
**Central Myofibroma of the Mandible in an Adult: Enucleation with Immediate Reconstruction Using a Patient-Specific Plate and Iliac Crest Bone Graft for Implant Site Preparation**
Nareerat Srihirundeth ^1^, Danaiya Supakanjanakanti ^2^ and Nattacha Jenwanichkul ^1^
*^1^* *Residency Training Program Oral and Maxillofacial Surgery, Faculty of Dentistry, Prince of Songkla University, Songkla, Thailand*
*^2^* *Lecturer, Department of surgery, Faculty of Dentistry, Prince of Songkla University, Songkla, Thailand*
**Objectives**: To report a rare case of central myofibroma of the mandible in an adult patient and to describe the surgical management using enucleation with immediate reconstruction via a patient-specific plate and iliac crest bone graft, with the goal of restoring adequate bone volume and profile for future dental implant placement.
**Materials and Methods**: A 58-year-old Thai female presented with a 3-month history of a 3 cm painful swelling in the right anterior mandible. CBCT revealed a well-defined unilocular radiolucent lesion with labial cortical expansion and perforation of both cortices. Incisional biopsy and immunohistochemistry confirmed the diagnosis of myofibroma. Enucleation was performed to preserve arch continuity. Given the critically thinned residual cortical plate and the elevated risk of pathological fracture, immediate reconstruction was carried out using a patient-specific plate. An iliac crest bone graft was used to restore sufficient bone volume and a favourable alveolar profile for future implant rehabilitation.
**Results**: The postoperative course was satisfactory. Temporary numbness of the lower lip and chin was observed. Intraoral wound healing was uneventful with no dehiscence. Postoperative CT confirmed the plate and screws at the planned positions, with adequate bone graft contour supporting the projected implant site.
**Conclusion**: Central myofibroma of the mandible in adults is rare and requires histopathological confirmation for definitive diagnosis. Enucleation combined with immediate reconstruction using a patient-specific plate and iliac crest bone graft effectively addressed the structural deficit, minimized intraoperative contouring time, reduced the risk of plate malalignment, and restored the alveolar profile necessary for future implant placement. A surgical guide further improved positional accuracy and streamlined the operative workflow.

**FA26/267—Oncology**
**Adenoid Cystic Carcinoma of Salivary Glands: A Ten-YearReview and an Assessment of the Current Management, Surgery, Radiotherapy and Chemotherapy**
Eyad Saleh ^1^
*^1^* *Eastman Dental Institute, London, UK*
**Objectives**: Adenoid Cystic Carcinoma (ACC) is a rare salivary gland malignancy accounting for 10% of salivary gland neoplasms and approximately 1% of head and neck cancers. It predominantly affects individuals in the sixth and seventh decades of life, with a slight female predilection (3:2). Lesions are typically slow-growing; pain and altered sensation indicate advanced disease. A hallmark feature is Perineural Invasion (PNI), contributing to local spread and a ~50% recurrence rate. Management is primarily surgical; radiotherapy improves local control with residual disease, though chemotherapy shows limited benefit.
**Materials and Methods**: PubMed/NLM and Cochrane Library were searched from August 2011 to August 2021 using terms related to “Adenoid Cystic Carcinoma” and “Salivary Glands.” Only salivary gland lesions were included. Duplicates, ineligible reports, and studies focusing on non-salivary ACC were excluded.
**Results**: Initial searches yielded 935 articles across both databases. After removing 278 duplicates, 323 ineligible reports, and 129 non-specific studies, 163 eligible studies remained—157 from PubMed and 6 clinical trials from Cochrane Library.
**Conclusion**: SACC is rare but among the most common salivary gland malignancies. Its etiopathogenesis remains poorly understood, though several genetic mutations and biomarkers have been identified. Diagnosis is complex, management is challenging, and recurrence and metastasis are frequent. Surgery with adjuvant radiotherapy remains first-line treatment. Advances in targeted therapies, including Tyrosine Kinase Inhibitors, offer promise but require further investigation. The rarity of SACC limits large-scale research; however, its aggressive clinical behaviour and poor prognosis warrant greater investment in laboratory and clinical studies to develop novel therapeutic approaches.

**FA26/336—Oncology**
**Masseter Muscle Carcinoma with Squamous Differentiation—A Rare Presentation**
Janice Thanga Raj ^1^, Jeremiah See ^1^, Mazita Ismail ^1^ and Jun Chan ^1^
*^1^* *Hospital Raja Permaisuri Bainun, Ipoh, Malaysia*
**Objectives**:
  (1) To identify the challenges of obtaining diagnosis of primary origin or metastatic disease
  (2) To explore the options of patient treatment
**Materials and Methods**: Case report. Multidisciplinary team approach
**Results**: 59 years old female with no medical illness, presented with 1-year painless progressive swelling over her right cheek. The lesion was 5 × 5 cm with poorly defined margin, fixed to the masseter muscle and no facial nerve palsy. CECT showed enhanced right masseter muscle lesion deep to the parotid gland with no primary or metastases disease seen. Ultrasound guided biopsy resulted in poorly differentiated squamous carcinoma after failed FNAC and open biopsy. Panendoscopy by the ENT review negative of airway tumors. She went through 2 cycles of chemotherapy which after that was withheld due to neutropenia and also 35 cycles of radiotherapy which shared significant reduction of disease with no functional deficit of massification and facial nerve palsy.
Metastasis to masseter muscle is extremely rare. There was no primary tumour identified in this case and it still leaves a room of suspicion if this is primary tumour. Further IHC will be send however regardless for now patient is under surveillance. HPE confirmation is crucial for definitive treatment plan. Surgery is crucial for definitive treatment plan however surgical resection of the tumour will cause significant morbidity especially injury to facial nerve. Therefore, the option of chemotherapy or radiotherapy comes in aid with comparative survival or recurrence rate.
**Conclusion**: Primary masseter carcinoma with squamous differentiation; is a rare disease which require multi-modality diagnostic tools and multidisciplinary approach with good response to chemotherapy or radiotherapy.

**FA26/341—Oncology**
**Thermal Versus Cold Instrument Incisions in Cervical Dissection for Oral Squamous Cell Carcinoma: A Prospective Comparative Study**
Sunil Kumar Gulia ^1^
*^1^* *SGT University, Gurugram, India*
**Objectives**: To compare intraoperative efficiency and postoperative outcomes between thermal electrocautery and cold steel incisions in cervical dissection for oral squamous cell carcinoma.
**Materials and Methods**: A prospective comparative study was conducted on 40 patients undergoing neck dissection. Patients were divided into cold steel and electrocautery groups. Intraoperative parameters included incision time, blood loss, and operative duration. Postoperative outcomes included pain scores, wound healing, drain output, and complications. Statistical analysis was performed with significance set at *p* < 0.05.
**Results**: Electrocautery significantly reduced incision time (7.4 ± 1.2 vs. 10.1 ± 1.6 min, *p* = 0.001) and blood loss (142.5 ± 30.2 vs. 198.4 ± 35.7 mL, *p* = 0.002). However, it was associated with higher postoperative pain (VAS 6.8 ± 0.7 vs. 5.2 ± 0.9, *p* < 0.001) and delayed wound healing (11.2 ± 1.3 vs. 9.1 ± 1.4 days, *p* = 0.004). No significant difference was observed in operative duration or oncological margin status.
**Conclusion**: Electrocautery improves intraoperative efficiency but is associated with increased postoperative pain and delayed wound healing. Cold steel incisions demonstrate better postoperative recovery while maintaining oncologic safety.

**FA26/344—Oncology**
**Central Versus Lateral Lip-Split Access in Head and Neck Oncologic Reconstruction: A Prospective Comparative Study**
Sunil Kumar Gulia ^1^
*^1^* *SGT University, Gurugram, India*
**Objectives**: To compare functional, aesthetic, and postoperative outcomes between central and lateral lip-split mandibulotomy approaches in head and neck oncologic resection with microvascular reconstruction.
**Materials and Methods**: A prospective comparative study was conducted in patients undergoing oral and oropharyngeal tumor resection with mandibulotomy and free flap reconstruction. Patients were divided into central lip-split and lateral lip-split groups. Outcomes assessed included operative exposure time, wound complications, salivary leakage, hospital stay, oral competence, speech intelligibility, and scar satisfaction. Statistical analysis was performed using independent *t*-test and chi-square test with significance set at *p* < 0.05.
**Results**: Central lip-split approach demonstrated shorter exposure time (18.6 ± 3.2 vs. 20.1 ± 3.5 min, *p* = 0.04). However, it was associated with higher wound dehiscence (18.7% vs. 5.8%, *p* = 0.03) and salivary leakage (15.6% vs. 5.8%, *p* = 0.04). The lateral lip-split group showed reduced hospital stay (10.8 ± 1.9 vs. 12.4 ± 2.1 days, *p* = 0.02) and significantly better functional outcomes, including oral competence (88.2% vs. 68.7%, *p* = 0.01), speech intelligibility (*p* = 0.02), and scar satisfaction (*p* = 0.01). Free flap success rates were comparable between groups.
**Conclusion**: Lateral lip-split approach provides superior functional and aesthetic outcomes with reduced postoperative morbidity while maintaining oncologic safety. It should be considered the preferred approach when long-term quality of life is a priority.

**FA26/345—Oncology**
**Digital Planning Versus Conventional Freehand Reconstruction in Fibula Free Flap Mandibular Reconstruction: A Comparative Study**
Sunil Kumar Gulia ^1^
*^1^* *SGT University, Gurugram, India*
**Objectives**: To compare the accuracy and clinical outcomes of digitally planned versus conventional freehand mandibular reconstruction using fibula free flap.
**Materials and Methods**: A retrospective comparative study was conducted on 40 patients undergoing mandibular reconstruction with fibula free flap following oncologic resection. Patients were divided into two groups: digitally planned reconstruction (n = 20) and conventional freehand reconstruction (n = 20). Digital planning included preoperative virtual surgical planning and patient-specific cutting guides. Conventional reconstruction was performed using intraoperative freehand techniques. Outcomes assessed included total operative time, reconstruction accuracy (measured as deviation between planned and achieved mandibular position in mm), and flap survival. Secondary parameters included intraoperative feasibility and postoperative outcomes. Statistical analysis was performed using independent *t*-tests, with significance set at *p* < 0.05.
**Results**: The digitally planned group demonstrated significantly reduced operative time (420 ± 35 vs. 510 ± 40 min, *p* < 0.01) and superior reconstruction accuracy (mean deviation 1.8 ± 0.6 mm vs. 4.5 ± 1.2 mm, *p* < 0.001) compared to the conventional group. Digital planning allowed more precise anatomical contouring and alignment. Flap survival rates were comparable between groups (95% vs. 90%), indicating no compromise in surgical safety. Improved intraoperative efficiency and reduced need for intraoperative adjustments were observed in the digitally planned group.
**Conclusion**: Digital planning significantly enhances reconstruction accuracy and reduces operative time without compromising flap survival in fibula free flap mandibular reconstruction. These findings support the integration of virtual surgical planning into routine practice to achieve predictable and reproducible outcomes in complex mandibular reconstruction.

**FA26/378—Oncology**
**Facial Reanimation Following Total Parotidectomy with Facial Nerve Sacrifice for a Left Parotid Adenoid Cystic Carcinoma: A Case Report**
Jose Paolo Diño ^1^ and Samantha Castañeda ^1^
*^1^* *Rizal Medical Center, Pasig City, Philippines*
**Objectives**: To describe immediate facial reanimation using combined temporalis muscle transposition and masseter muscle transfer following total parotidectomy with facial nerve sacrifice in a patient with adenoid cystic carcinoma.
**Materials and Methods**: A 33-year-old male presented with a 4-year history of a progressively enlarging left infraauricular mass associated with pain and facial weakness. Examination revealed House-Brackmann (HB) grade VI facial paralysis on the left. Imaging showed a heterogeneous parotid lesion, while fine needle aspiration biopsy was inconclusive. Due to high clinical suspicion for malignancy, the patient underwent total parotidectomy with facial nerve sacrifice, selective neck dissection (levels I–III), and immediate facial reanimation using temporalis muscle transposition for upper facial reanimation and masseter muscle transfer for lower facial reanimation.
**Results**: On postoperative day 1, the patient achieved complete left eye closure, although this regressed during the first postoperative week. At 1-month follow-up, partial recovery of eye closure with improved movement was noted. Oral competence improved immediately postoperatively, with resolution of drooling and maintained ability to achieve complete mouth closure during drinking at 1 month. Final histopathology confirmed adenoid cystic carcinoma.
**Conclusion**: Immediate combined temporalis and masseter muscle transfers provide effective dynamic facial reanimation following facial nerve sacrifice, enabling early functional recovery of eye closure and oral competence while avoiding more complex microsurgical reconstruction.

**FA26/380—Oncology**
**Grpwth Suppression of Oral Squamous Cell Carcinoma Cells by Lactobacillus Acidophilus**
Adel Al-Asfour ^1^
*^1^* *Research Administration, Kuwait University, Grant SRUL 01/14, Kuwait, Kuwait*
**Objectives**: Oral squamous cell carcinoma (OSCC) is a highly aggressive form of oral cancer. Probiotic Lactobacillus have shown anticancer effects, whilst their interaction with Streptococcus mutans in this context remains unexplored. The objective of this study was to investigate the antiproliferative effect of Lactobacillus acidophilus on SCC and to understand the effect of S mutans on OSCCs and whether it effects the antiproliferative potential of L acidophilus when co-exposed to OSCC.
**Materials and Methods**: The human head and neck squamous cell carcinoma cells of the oral cavity (HNO97 cell line) were exposed to cultures of L acidophilus and S mutans separately and in combination. Further, 3-(4,5-dimethylthiazol-2-yl)-2,5-diphenyltetrazolium bromide assay was performed to assess the viability of HNO97 cells. Bacterial adhesion to HNO97 cells was examined by confocal microscopy and apoptosis, expression of the TNF-related apoptosis-inducing ligand (TRAIL) gene and protein were determined by real-time polymerase chain reaction and quantitative enzyme-linked immunosorbent assay, respectively.
**Results**: A significant decrease (53–56%) in the viability of HNO97 cells on exposure to L acidophilus, S mutans and 2 species together demonstrated the antiproliferative activity of L acidophilus and S mutans. Both bacteria showed adhesion to HNO97 cells. The expression of the TRAIL gene increased 5-fold in HNO97 cells on treatment with L acidophilus and S mutans, which further increased to 17-fold with both species present. Expression levels of the trail protein were significantly increased in bacteria-treated cell lysates. Further, bacteria-treated HNO97 cells exhibited lower live and intact cell percentage with higher proportions of cells in early and late apoptotic stages.
**Conclusion**: L acidophilus exhibits the antiproliferative activity against OSCC cells possibly partially via a TRAIL-induced mechanism of apoptosis, which is not affected by the presence of S mutans. These findings may encourage further investigation into the possible therapeutic application of probiotic L acidophilus in OSCC.
Conflict of interest disclosure
Receipt of grants/research supports: Research Administration, Kuwait University, Grant SRUL 01/14.

**FA26/396—Oncology**
**Calcifying Odontogenic Cyst with Compound Odontoma of the Maxilla: A Case Report**
Patchara Phimolthares ^1^
*^1^* *Department of Oral and Maxillofacial Surgery, Faculty of Dentistry Mahidol University, Bangkok, Thailand*
**Objectives**: To report a case of calcifying odontogenic cyst (COC) associated with compound odontoma of the maxilla in a young patient, highlighting clinical presentation, surgical management, and postoperative outcomes.
**Materials and Methods**: A 19-year-old Lao male presented with progressive left facial swelling of 7–8 years duration. He had previously undergone incision and drainage in Laos with partial resolution. Extraoral examination revealed swelling at the left paranasal region. CBCT demonstrated a well-defined unilocular radiolucency at the left maxilla associated with compound odontoma and embedded teeth 23 and 24. Initial incisional biopsy suggested a dentigerous cyst; however, re-biopsy confirmed COC with compound odontoma. The patient subsequently underwent enucleation with curettage and removal of associated odontoma and embedded teeth under general anesthesia.
**Results**: At six-month follow-up, extraoral examination demonstrated reduced bony swelling at the left maxilla. Intraoral examination revealed an oronasal fistula (ONF) measuring 1 × 1.5 cm with normal surrounding tissue. CBCT confirmed increasing bone density at the operative site, indicating progressive bone formation.
**Conclusion**: COC is a rare benign odontogenic lesion accounting for less than 1% of all jaw cysts, with approximately 20% of cases associated with odontoma (COaCOC) as reported by the WHO. COaCOC characteristically affects teenage patients, shows predilection for the anterior maxilla, and appears radiographically unilocular in 96.8% of cases. COaCOC of the maxilla presents a surgical challenge due to lesion extent and risk of oronasal communication. Conservative enucleation with curettage provides effective oncologic control with low recurrence rates; however, awareness of postoperative ONF and the need for staged reconstruction remains essential for optimal long-term outcomes.

**FA26/417—Oncology**
**Strategic Management of Deep-Lobe Pleomorphic Adenoma Abutting the Temporomandibular Joint: A Case Study on Functional Preservation**
Sudirman Sudirman ^1^, Bramadita Satya ^2^ and Andi Budihardja ^1^
*^1^* *Faculty of Dentistry Universitas Pelita Harapan, Siloam Hospital Lippo Village, Tangerang Regency, Indonesia*
*^2^* *Siloam Hospital Lippo Village, Tangerang Regency, Indonesia*
**Objectives**: This report delineates the surgical nuances in the total extirpation of a benign parotid neoplasm situated within the deep lobe of the parotid gland, specifically in the immediate anterior proximity of the right temporomandibular joint (TMJ). The aim is to demonstrate the efficacy of a preauricular surgical corridor in achieving radical clearance of deep-seated lesions while safeguarding the structural and functional integrity of the facial nerve and stomatognathic apparatus.
**Materials and Methods**: A patient presented with a mass located deep and anterior to the condylar head. Surgical intervention utilized a preauricular approach to access and identify a 2.6 × 1.3 × 0.7 cm tumor residing within the deep lobe of the parotid gland. Precise micro-dissection was employed to isolate facial motor nerve branches and preserve the parotid ductal architecture. Given the lesion’s adherence to the TMJ capsule and masticatory muscles, meticulous plane-by-plane dissection was executed to preclude capsular disruption or postoperative joint dysfunction.
**Results**: Histopathological analysis confirmed a pleomorphic adenoma with classic biphasic morphology, exhibiting nests of ductal epithelial and myoepithelial elements within a chondromyxoid stroma. The postoperative course was unremarkable, showing no evidence of facial nerve neuropraxia or sialocele formation. Clinical follow-up demonstrated a stable maximal interincisal opening within physiological parameters and a complete resolution of symptoms without masticatory interference.
**Conclusion**: Pleomorphic adenomas located in the deep lobe and preauricular space present significant challenges due to the proximity of vital neurovascular and arthrodial structures. This case demonstrates that a controlled preauricular approach provides optimal visualization for the safe excision of deep-lobe tumors. Achieving superior clinical outcomes—specifically the dual preservation of neural integrity and functional joint excursion—is contingent upon advanced mastery of regional anatomy and atraumatic surgical technique.

**FA26/429—Oncology**
**Does the Non Radiated Non Vascularised Bone Graft (nvbg) Fare Better Than Radiated nvbg in Reconstruction of Oral Cancer Defects? A Systematic Review and Meta Analysis**
Amanjot Kaur ^1^, Aparna Ganesan ^2^, Sibgutulah Rashid ^3^, Nandakishor Nair ^1^ and Paras Khanna ^1^
*^1^* *AIIMS Jammu, Vijay Pore, India*
*^2^* *Privaram Cancer & Research Institute, Chennai, INDIA, Chennai, India*
*^3^* *AIIMS Patna, Patna, India*
**Objectives**: To compare post surgical functional outcomes and complications of NVBGs for segmental mandibular defects resulting from oral cancer resection between radiated and non-irradiated patients
**Materials and Methods**:
**Eligibility Criteria:** Studies comparing outcomes between radiated and irradiated patients who underwent NVBG reconstruction for segmental mandibular defects after oral cancer resection. Case reports, editorials, reviews, animal studies were excluded.
**Information sources:** A comprehensive electronic search of PubMed, Scopus, OvidSP, Embase, Cochrane Library, databases of oral and maxillofacial surgery journals, CTRI and American Trial Registry including articles from inception to June2025.
**Risk of Bias:** Methodological quality and risk of bias of studies assessed by Newcastle Ottawa Scale.
**Synthesis of results:** Two authors independently extracted data as per Cochrane Collaboration’s standard data collection form, 2013 08 12, based on PRISMA guidelines. Study demographics, type of graft, defect characteristics, irradiation details recorded. A quantitative synthesis was performed for graft failure, defined as clinical or radiographic non-union, between irradiated and non-irradiated NVBG.
**Results**:
**Included studies:** Four studies involving 125NVBGs were included. 57 (45.6%)were anterior iliac cortico-cancellous block grafts, 65 (52%) were particulate cancellous grafts from anterior iliac crest, 3 (2.4%) anterior or posterior iliac crest grafts. 71 (56.8%) were placed into a preradiated surgical bed or subjected to radiation post surgery.
**Synthesis of results:** Pooled risk ratio for irradiated group compared to non-irradiated group was 2.83 (95% CI: 0.75 to 10.61),indicating no statistically significant difference for graft failure (*p* = 0.12).
**Conclusion**:
**Limitations of evidence:** On quality assessment with NOS, 2 studies were good quality (scores ≥ 7) and 2 studies were low quality (scores < 7). Certainty of evidence determined by GRADE analysis for graft failure in irradiated compared with non-irradiated patients was rated as very low.
**Interpretation:** Our analysis did not detect a statistically significant difference in NVBG survival between irradiated and non-irradiated patients, the confidence interval being wide. Findings suggest that NVBG can be a valuable reconstructive option in resource-limited environments or when patient comorbidities preclude microsurgery.

**FA26/474—Oncology**
**Sohin’s Node—An Indicator for a Poor Over All Survival?**
Sohin Chaudhari ^1^
*^1^* *D Y Patil Deemed To Be University—School Of Dentistry, Navi Mumbai, Navi Mumbai, India*
**Objectives**: To confirm if a single node on the Internal Jugular Vein is directly related to a poor N stage in cases of OSCC and affects the Over-all Survival as well as Disease Free Survival of the patient.
**Materials and Methods**: A retrospective study of three patients was done at one single institution diagnosed with oral cancer. A single surgeon performed the surgery and a single large node over the Internal Jugular Vein at the area of Common Facial Vein and Internal Jugular Vein bifurcation was identified, excised separately and sent for histopathology along with the other samples of neck dissection. After the histopathology report was available, the reports were studied.
**Results**: It was noted that this node was directly associated with depth of invasion affecting the T stage, presence of extra nodal extension of lymph node along with metastatic deposit in the neck in all the three cases
**Conclusion**: The node may be considered an indicator for a higher N stage in the neck and affecting overall survival of the patient.

**FA26/504—Oncology**
**Network Pharmacology and Molecular Docking-Based Multi-Target Mechanism of Curcumin on Squamous Cell Carcinoma Genes**
Deddy Septian ^1^, Nurani Atikasari ^2^, Abel Yuza ^1^ and Andri Hardianto ^1^
*^1^* *Universitas Padjadjaran, Kota Bandung, Indonesia*
*^2^* *Universitas Airlangga, Kota Surabaya, Indonesia*
**Objectives**: The present study evaluates the multi-target therapeutic potential of curcumin in oral squamous cell carcinoma (OSCC) using network pharmacology and molecular docking approaches to identify adjunctive molecular pathways for mitigating disease recurrence and enhancing long-term post-surgical survival.
**Materials and Methods**: The databases SwissTargetPrediction, CTD, DisGeNet, and TTD were used to identify curcumin targets and OSCC-related genes. Gene Ontology annotation and KEGG pathways enrichment were performed using Metascape. Molecular docking was performed using PyRx to determine binding affinities against key OSCC targets. Standard-of-care inhibitors were used as comparative positive controls to establish a baseline of efficacy. Preliminary pharmacokinetic viability was assessed by in silico ADMET profiling.
**Results**: The network analysis identified 12 critical pathways driving OSCC pathology, with the p53 signaling network prominently featured. Curcumin showed highly competitive binding affinities for the eight major OSCC targets PIK3CA, PTEN, TNFRSF10B, CASP8, TP53, EGFR, CDKN2A, and CCND1. The strongest interaction was with PIK3CA (−8.6 kcal/mol), which competed favorably with well-established control inhibitors. Importantly, critical stabilizing interactions, including specific hydrogen bonding at the ASN170 residue of PIK3CA, indicate a strong potential to disrupt oncogenic protein kinase activity and positively influence apoptosis despite baseline pharmacokinetic limitations.
**Conclusion**: Curcumin exhibits strong multi-target binding efficacy comparable with targeted biologic therapies. When optimized for bioavailability, it has significant potential as a low-toxicity post-surgical adjunctive agent for inhibiting OSCC progression and restoring apoptotic function in cells.

**2FA26/1021—Oncology**
**Mandibular Desmoid-Type Fibromatosis: Navigating the Diagnostic and Surgical Challenges in a Pediatric Patient**
S. Hemanth Kumar ^1^, Uma Shanker Pal ^1^, Vijay Kumar ^1^, Sakshi Bhat ^1^ and Rukmini Sah ^1^
*^1^* *King George’s Medical University, Lucknow, India*
**Objectives**: To present the critical management of a rapidly proliferating mandibular desmoid-type fibromatosis (DTF) in a 2-year-old female, highlighting the diagnostic challenges of spindle cell neoplasms and the necessity of surgical intervention for impending airway obstruction.
**Materials and Methods**: A 2-year-old female presented with an 8-month history of a rapidly enlarging right submandibular mass. Initial high-resolution ultrasound (HRUSG) suggested sialadenitis, a common diagnostic pitfall in pediatric myofibroblastic lesions. Clinical examination revealed the tumor proliferating from the infero-lingual aspect of the mandibular body, displacing the submandibular gland and compressing the hyoid bone, thereby threatening the airway. Fine-needle aspiration cytology (FNAC) suggested a spindle cell tumor, but the aggressive growth rate raised suspicion of malignancy. An incisional biopsy under general anesthesia yielded conflicting results: one report suggested connective tissue malignancy, while the second report utilized immunohistochemistry (IHC), revealing a Beta-catenin and Vimentin positive profile while remaining negative for SMA, CD34, and S100, with a Ki-67 index of 3–4%, confirming DTF.
**Results**: Given the tumour’s encroachment on the hyoid and airway, medical oncology advised radical surgical management over medical therapy. A segmental resection of the right mandible with in-toto lesion, from the lateral incisor to the angle, was performed to achieve oncologic clearance. Although a vascularized free fibula flap was the intended reconstructive strategy, an intraoperative finding of a peronea magna vascular variant necessitated an immediate pivot to a non-vascularized free fibula graft to preserve limb viability. Final histopathology confirmed margins free of tumor.
**Conclusion**: Pediatric DTF can masquerade as common inflammatory conditions, making IHC-based diagnosis vital. In the presence of rapid growth and airway compromise, radical segmental resection is justified to ensure survival. This case demonstrates that intraoperative adaptability and multidisciplinary coordination are essential for managing rare mesenchymal neoplasms in the pediatric maxillofacial skeleton.

**2FA26/1049—Oncology**
**Application of toluidine blue for early detection of oral cancer and precancerous lesions.**
Fahmida Ferdousi ^1^
*^1^* *Evercare Hospitals Dhaka, Bangladesh, Dhaka, Bangladesh*
**Objectives**: This observational study aims to identify specific regions of oral lesions for histopathological diagnosis and assess the correlation between toluidine blue dye uptake in oral tissues and histopathological findings in premalignant and cancerous lesions.
**Materials and Methods**: A total of 216 oral lesion cases were selected based on predetermined inclusion and exclusion criteria, without restrictions on age, gender, religion, or socioeconomic status. Patients were identified via medical records and clinical investigations, focusing on nonhealing oral ulcers lasting over three weeks without prior oral cancer diagnosis. Only toluidine blue-positive cases were examined further. Histopathological analyses were conducted on toluidine blue-positive areas to detect premalignant and malignant lesions.
**Results**: Among the 216 cases, histopathological findings showed 98 cases of hyperplastic epithelium, 14 of verrucous carcinoma, 34 of premalignant lesions, 12 of dysplasia, and 58 of squamous cell carcinoma (SCC). Toluidine blue dye had a 100% staining response rate, with 86.2% accuracy for carcinoma and 15.7% for premalignant lesions. A significant histopathological difference was noted between oral potentially malignant lesions and SCC.
**Conclusion**: The findings suggest toluidine blue dye enhances early detection of malignant and premalignant oral lesions, improving diagnostic accuracy over traditional visual or therapeutic methods. This approach aids clinicians in promptly identifying high-risk lesions, potentially boosting patient outcomes.

**2FA26/1073—Oncology**
**Salivary Multi-Omics Biomarker Signatures for Risk Stratification Across the Oral Carcinogenesis Spectrum: From Oral Potentially Malignant Disorders to Oral Squamous Cell Carcinoma**
Ayesha Rais ^1,2^ and Hafiz Waqas Ahmed ^3^
*^1^* *Bolan Medical Hospital, Quetta, University of Medical and Health Sciences, Pakistan, Mayo Hospital, Lahore, King Edward Medical University, Pakistan, Quetta, Pakistan*
*^2^* *Department of Oral & Maxillofacial Surgery, Bolan Medical Complex/Bolan University of Medical and Health Sciences, Quetta, Pakistan, Quetta, Pakistan*
*^3^* *Department of Oral & Maxillofacial Surgery, Mayo Hospital/King Edward Medical University, Lahore, Pakistan, Lahore, Pakistan*
**Background:** Oral squamous cell carcinoma (OSCC) develops through progressive molecular alterations. Oral potentially malignant disorders (OPMDs) represent an intermediate stage of carcinogenesis and offer opportunities for early risk assessment using non-invasive biomarkers.
**Objective:** To evaluate salivary multi-omics biomarker signatures for differentiating healthy controls, OPMDs, and OSCC patients.
**Methods:** This multicenter prospective study included 400 participants: 200 OSCC patients, 120 OPMD patients, and 80 healthy controls. Saliva samples were analyzed for transcriptomic (miR-21, miR-145, miR-423-5p), protein (EGFR, IL-8, CYFRA 21-1, p53, Ki-67), and exosomal (Exo-miR-1246, Exo-EGFR) biomarkers using qRT-PCR, ELISA, and exosome isolation protocols. Biomarker expression was compared using ANOVA, multivariate analyses, and ROC analysis.
**Results:** Significant molecular alterations were observed across controls, OPMDs, and OSCC patients (*p* < 0.001). OPMD patients demonstrated intermediate biomarker expression levels between controls and OSCC patients. Expression of Exo-miR-1246, miR-21, EGFR, CYFRA 21-1, and Ki-67 increased significantly with disease progression. Combined biomarker signatures demonstrated superior discriminatory performance compared with individual biomarkers.
**Conclusion:** Salivary multi-omics biomarkers show promise for non-invasive risk stratification across the oral carcinogenesis spectrum and may facilitate early identification of high-risk lesions and improved surveillance of OPMDs.
**Keywords:** Oral squamous cell carcinoma; Oral potentially malignant disorders; Salivary biomarkers; Multi-omics; Risk stratification

**2FA26/1098—Oncology**
**Immediate Reconstruction After Posterolateral Marginal Mandibulectomy for Pediatric Plexiform Ameloblastoma Using a Non-Vascularized Costal Graft and Heavy-Profile Titanium Miniplate: A Case Report**
Ocly Davira Putri Laisnima ^1^ and Agus Roy Rusly Hariantana Hamid ^2^
*^1^* *Resident of Plastic, Reconstructive, and Aesthetic Surgery Residency Program, Faculty of Medicine, Udayana University, Denpasar, Indonesia*
*^2^* *Division of Plastic, Reconstructive, and Aesthetic Surgery, Ngoerah Hospital, Bali, Indonesia, Coordinator of Plastic, Reconstructive, and Aesthetic Surgery Residency Program, Faculty of Medicine, Udayana University, Denpasar, Indonesia*
**Objectives**: Pediatric mandibular reconstruction following ameloblastoma resection remains challenging because restoring mandibular continuity must be balanced against the preservation of facial growth, oral function, and aesthetic outcome. This report describes immediate reconstruction using a non-vascularized costal graft and heavy-profile titanium miniplate following posterolateral marginal mandibulectomy in a pediatric patient with plexiform ameloblastoma.
**Materials and Methods**: A 13-year-old boy presented with a progressively enlarging left mandibular mass for four months. Imaging demonstrated an expansile intraosseous lesion involving the left mandibular corpus, angle, and ramus, suggestive of ameloblastoma. The patient underwent posterolateral marginal mandibulectomy, resulting in a 6-cm mandibular defect, followed by immediate reconstruction using an autologous sixth costal graft stabilized with a heavy-profile 2.0-mm titanium miniplate. Maxillomandibular fixation was maintained for four weeks postoperatively.
**Results**: Histopathological examination confirmed conventional plexiform ameloblastoma. The patient was hospitalized for seven days without major postoperative complications. Postoperative radiographs demonstrated satisfactory graft alignment and stable fixation. At one-month follow-up, graft take was successful without graft infection, wound dehiscence, or donor-site morbidity. Occlusion and mandibular contour were preserved with complete intraoral epithelialization. Mild trismus persisted with a maximal interincisal opening of 1 cm, and rehabilitation therapy was initiated. Both the patient and family were satisfied with the postoperative functional and aesthetic outcomes.
**Conclusion**: Immediate reconstruction with a non-vascularized costal graft and a heavy-profile titanium miniplate may be a reliable option for selected pediatric mandibular defects following ameloblastoma resection, providing satisfactory early functional and aesthetic outcomes while avoiding the complexity of microsurgery.

**2FA26/1104—Oncology**
**Submental Island Flap can be a good option for “Tongue Reconstruction After Excision for Squamous Cell Carcinoma with N0 Neck”**
Fahmida Ferdousi ^1^, Motiur Molla ^1^ and Golam Chowdhury ^1^
*^1^* *Evercare Hospitals Dhaka, Bangladesh, Dhaka, Bangladesh*
**Objectives**: To evaluate the effectiveness and reliability of the submental island flap for tongue reconstruction following excision of squamous cell carcinoma in patients with clinically N0 neck disease.
**Materials and Methods**: A retrospective study was conducted on patients diagnosed with squamous cell carcinoma of the tongue with clinically N0 neck nodes who underwent tumor excision followed by reconstruction using a submental island flap. Patient demographics, tumor characteristics, operative details, flap viability, postoperative complications, functional outcomes including speech and swallowing, and aesthetic results were assessed during follow-up.
**Results**: The submental island flap provided adequate soft tissue coverage and satisfactory reconstruction of tongue defects in most patients. Flap survival was high with minimal donor site morbidity. Functional outcomes regarding speech and swallowing were acceptable, and cosmetic outcomes were satisfactory. Postoperative complications such as partial flap necrosis, infection, or wound dehiscence were minimal. No significant cervical nodal recurrence was observed during the follow-up period in appropriately selected N0 neck patients.
**Conclusion**: The submental island flap is a reliable, versatile, and cost-effective option for tongue reconstruction after excision of squamous cell carcinoma in patients with N0 neck disease. It offers good functional and aesthetic outcomes with low donor site morbidity and can be considered a valuable reconstructive technique in selected patients.

**2FA26/1157—Oncology**
**Preoperative Warning Signs for Non-Clear Surgical Margins in Complex Facial Skin Cancer: A Preliminary Single-Center Study**
Muhamad Saifuddin ^1^ and Kiki Rizki ^2^
*^1^* *Faculty of Medicine, Padjadjaran University, Bandung, Indonesia*
*^2^* *Department/Division of Surgical Oncology, Dr. Hasan Sadikin General Hospital, Bandung, Indonesia*
**Objectives:** Complex facial skin cancers frequently involve critical midfacial structures, making complete oncologic clearance challenging. This study aimed to evaluate clinicopathological and anatomical factors associated with non-clear surgical margins after resection of complex facial skin cancer.
**Materials and Methods:** A preliminary retrospective analytical study was performed using an anonymized dataset of patients with facial skin cancer who underwent surgical resection between January 2025 and April 2026. Margin status was categorized as clear or non-clear according to final postoperative pathological margin interpretation. The analyzed variables were histopathology, tumor size > 4 cm, recurrent disease, prior radiotherapy, maxillary sinus invasion, maxillary invasion, orbital invasion, intraoperative frozen section/consultation, and margin status. Comparisons between clear and non-clear margin groups were performed using Fisher exact test.
**Results**: A total of 48 cases were analyzed. The mean age was 65.3 ± 13.3 years, and the median age was 67 years (range, 14–89 years). Non-clear margin was identified in 32 cases (66.7%), while clear margin was achieved in 16 cases (33.3%). Tumor size > 4 cm was significantly associated with non-clear margin (25/32 vs. 7/16; OR, 4.59; *p* = 0.025). Maxillary invasion was found exclusively in the non-clear margin group (8/32 vs. 0/16; *p* = 0.039). Orbital invasion was also significantly more frequent in the non-clear margin group (13/32 vs. 1/16; OR, 10.26; *p* = 0.018). Recurrent disease, prior radiotherapy, and maxillary sinus invasion were more common among non-clear cases but did not reach statistical significance.
**Conclusion:** Tumor size > 4 cm, maxillary invasion, and orbital invasion were significantly associated with non-clear surgical margins. These practical preoperative warning signs may support multidisciplinary planning, margin strategy, reconstructive preparation, and patient counseling in complex facial oncology surgery.
Keywords: facial skin cancer; surgical margin; basal cell carcinoma; cutaneous squamous cell carcinoma; maxillary invasion; orbital invasion; reconstructive surgery

**2FA26/1161—Oncology**
**Mucosal Melanoma of the Hard Palate: A Case Report with Surgical Management and Literature Review**
Fairouz Bekrentchir ^1^
*^1^* *University hospital of Oran, ORAN, Algeria*
**Objectives**: Oral mucosal melanoma (OMM) is a rare, aggressive malignancy, representing less than 1% of all melanomas. The hard palate is the most frequent site, and early diagnosis is critical due to high local invasiveness and poor prognosis. Bone invasion can occur even in small lesions, emphasizing the importance of prompt surgical management.
**Materials and Methods**: A 30-year-old male, active smoker, presented with a **3-month history of a rapidly enlarging, pigmented lesion** of the posterior third of the hard palate. Initially asymptomatic, the lesion progressed to a 1.8 × 1.6 cm exophytic mass causing partial obstruction of the oral cavity.
Cervicofacial CT demonstrated a well-defined, hypodense lesion with **erosion of the palatal bone (~5 mm transosseous extension)** and bilateral cervical lymphadenopathy (levels IIb–III; largest nodes 21 × 26 mm right, 18 × 14 mm left). No distant metastases were identified.
Biopsy and immunohistochemistry confirmed **primary mucosal melanoma of the palate**.
Preoperative fabrication of a **resin obturator prosthesis** facilitated postoperative rehabilitation. The patient underwent **partial maxillectomy with 4-cm oncologic margins including the dental block**, combined with **bilateral functional neck dissection**. The defect was left for **secondary healing**, with adjuvant immunotherapy initiated postoperatively.
**Results**: OMM is often diagnosed late, with bone invasion frequently indicating advanced disease. According to AJCC classification, this case corresponds to **T4a N + M0 (Stage IVA)**. Surgical excision with wide margins and neck dissection remains the standard of care. Preoperative prosthetic planning enhances functional outcomes, while adjuvant immunotherapy addresses high-risk disease. The **rapid 3-month progression** in a young patient underscores the aggressive nature of mucosal melanoma and the need for early recognition.
**Conclusion**: This case illustrates that even small lesions can demonstrate early bone invasion and nodal involvement. Multidisciplinary management combining **oncologic surgery, prosthetic planning, and immunotherapy** is essential to optimize both survival and oral function.

**2FA26/1183—Oncology**
**Oral Basaloid Squamous Cell Carcinoma with Initial Clear Cell Features: A Diagnostic and Surgical Challenge**
Mohd Hazwan Mohd Ghazali ^1^, Nurharnani Harun ^1^, Mohd Kherman Suparman ^1^, Nazer Berahim ^1^, Tengku Intan Baizura Binti Tengku Jamaluddin ^1^, Azmeel Mazlee Anuar ^1^, Shah Jumaat Mohd Yusoff ^1^, Masaany Mansor ^1^ and Hazmyr Abdul Wahab ^1^
*^1^* *University Teknologi MARA, Sungai Buloh, Malaysia*
**Introduction**: Oral basaloid squamous cell carcinoma (BSCC) is a rare and aggressive variant of oral squamous cell carcinoma (OSCC) accounting for less than 1% of all OSCC cases. This case report aims to highlight the diagnostic challenge posed by focal clear cell changes on initial biopsy that may mask underlying BSCC, the clinicopathological features of HPV-associated BSCC in a young patient, and the complexity of its multidisciplinary surgical management.
**Case Report**: A case report of a 38-year-old male with a five-year history of untreated swelling in the anterior mandible, on the lingual region. Clinical, radiological, histopathological, and immunohistochemical findings were reviewed. Surgical interventions and reconstruction strategies were recorded and analyzed.
**Discussion**: Incisional biopsy showed moderately differentiated squamous cell carcinoma with focal clear cell changes, with positive result on immunohistochemistry staining for P16, indicating HPV association. On contrast enhanced computed tomography a 1.9 × 2.1 × 1.6 cm soft tissue lesion of the mandible was identified with erosion of the cortical bones, but no distant metastasis. Definitive resection specimen revised to oral BSCC poorly differentiated (Grade 3), measuring 36 mm, medullary invasion, perineural invasion of the mandibular cortex. Pathological staging was pT4a pN0 and all 26 lymph nodes were negative for metastasis. Surgical management included tracheostomy, bilateral modified radical neck surgery, anterior mandibulectomy, resection of the floor of mouth and reconstruction with a pectoralis major osteomyocutaneous pedicled flap.
**Conclusion:** This case highlights the difficulty in diagnosis of BSCC as a focal area of clear cell on initial biopsy can mask the actual histological diagnosis. High HPV positivity at a young age group with late presentation was involved in advanced disease at the time of resection. Multidisciplinary approach is still fundamental to obtain oncological control and dealing with the complexity of reconstructive surgery in this rare cancer.

**2FA26/1185—Oncology**
**Medication-Related Osteonecrosis of the Jaw in Indonesian Oncology Patients: A High-Burden Disease Driven by Functional Status and Oral Risk Factors**
Lilis Iskandar ^1^, Dinda Fadhliana ^1^, Rahmat Cahyanur ^2^, I Gusti Wibisana ^3^, Menik Priaminiarti ^4^, Bramma Kiswanjaya ^4^, Takao Fuchigami ^5^, Dwi Ariawan ^1^, Yudy Utomo ^1^ and Lilies Sulistyani ^1^
*^1^* *Oral and Maxillofacial Surgery Department, Faculty of Dentistry, Universitas Indonesia, Central Jakarta, Indonesia*
*^2^* *Medical Haematology Oncology Division, Internal Medicine Department, Faculty of Medicine, Universitas Indonesia, Central Jakarta, Indonesia*
*^3^* *Surgical Oncology Division, Surgery Department, Faculty of Medicine, Universitas Indonesia, Central Jakarta, Indonesia*
*^4^* *Dentomaxillofacial Radiology Department, Faculty of Dentistry, Universitas Indonesia, Central Jakarta, Indonesia*
*^5^* *Oral and Maxillofacial Oncology, Division of Maxillofacial Diagnostic Surgical Sciences, Faculty of Dental Science, Kyushu University, Fukuoka, Japan*
**Objectives**: Medication-related osteonecrosis of the jaw (MRONJ) is a serious adverse event associated with antiresorptive therapy in patients with cancer. Although the burden of MRONJ has been extensively investigated in high-income countries, evidence from Southeast Asia remains scarce. This study aimed to determine the prevalence of MRONJ and identify factors associated with its occurrence in an Indonesian oncology population.
**Materials and Methods**: A cross-sectional study was conducted at a national referral hospital in Indonesia. Adult patients with multiple myeloma or bone metastases receiving intravenous antiresorptive therapy were evaluated clinically and radiographically. MRONJ was diagnosed and staged according to the 2022 American Association of Oral and Maxillofacial Surgeons (AAOMS) criteria. Demographic, systemic, medication-related, and oral health variables were analyzed using multivariable logistic regression.
**Results**: Sixty-two patients were included. MRONJ was identified in 21 patients, yielding a prevalence of 33.9%. Most cases were diagnosed at an early stage, with Stage 0 accounting for 24.2% of the cohort. Multivariable analysis demonstrated that impaired functional status (Eastern Cooperative Oncology Group score ≥ 1; OR 3.94, 95% CI 1.12–13.86) and the presence of teeth requiring extraction or a history of oral surgical procedures during antiresorptive therapy (OR 8.18, 95% CI 1.51–44.25) were independently associated with MRONJ.
**Conclusion**: This study provides the first data on MRONJ prevalence in an Indonesian oncology population and demonstrates a substantially higher prevalence than most published reports. The findings suggest that local oral disease and systemic functional impairment are major contributors to MRONJ risk. Integration of routine dental assessment into oncology care pathways may be essential for reducing MRONJ burden in resource-constrained healthcare settings.

**2FA26/1212—Oncology**
**Evolving Epidemiology of Early-Onset Lip and Oral Cavity Cancer in South Asia: Female Burden, Sex Disparities, and Risk Attribution, 1990–2023—Analysis of Global Burden of Disease**
Jaspreet Kaur Deo ^1^ and Lucky Singh ^1^
*^1^* *Indian Council of Medical Research, New Delhi, India*
**Background:** Lip and oral cavity cancer is a major public health challenge in South Asia, characterized by widespread smokeless tobacco use. While the disease has been historically male predominant, contemporary trends among younger women remain relatively unexplored. This study evaluated the burden, temporal trends, sex disparities, and risk-factor attribution of early-onset lip and oral cavity cancer across South Asia between 1990–2023.
**Methods:** Using the GBD 2023 estimates, ASIR, ASMR, and DALYs with 95% uncertainty intervals were analysed for individuals aged 15–49 years in India, Pakistan, Bangladesh, Nepal, and Bhutan, with South Asia and global estimates for comparison. Female-to-male rate ratios were used to assess sex disparities. Risk-attributable fractions for smokeless tobacco, smoking, and alcohol use were examined using GBD-derived population attributable fractions.
**Results:** Between 1990 and 2023, age-standardized incidence rates among women increased across all study countries, with the largest increases observed in Bangladesh (+70.9%) and Pakistan (+44.6%). Surprisingly, Pakistan recorded the highest female burden, with ASIR rising from 17.4 to 25.2 per 100,000 and DALY rates from 363.3 to 477.9 per 100,000. Female-to-male incidence ratios increased in Pakistan from 1.55 to 1.73, indicating a sustained female predominance, whereas ratios remained below unity in other countries. Although absolute case counts increased substantially across the region, increases in age-standardized rates were comparatively modest. Smokeless tobacco represented the largest attributable behavioural risk factor for female burden across South Asia, whereas high alcohol use contributed minimally.
**Conclusions:** Early-onset lip and oral cavity cancer is an increasingly emergent health concern among South Asian women. The sustained female predominance observed in Pakistan suggests that evolving gender-specific behavioural and sociocultural risk exposures may be reshaping the epidemiology of oral cancer in the region. Smokeless tobacco remains the principal modifiable risk factor, reiterating the need for targeted prevention and tobacco-control strategies aimed towards young women.

**2FA26/1230—Oncology**
**Neoadjuvant Dabrafenib Monotherapy Enabling Conservative Surgery in BRAF V600E–Mutated Ameloblastoma: A Case Report**
Antti Pakkanen ^1^, Timo Muhonen ^2^, Marianne Nixdorf ^3^, Jetta Kelppe ^4^ and Tommy Wilkman ^5^
*^1^* *Department of Oral and Maxillofacial Diseases, University of Helsinki, Helsinki, Finland*
*^2^* *Department of Oncology, Faculty of Medicine, University of Helsinki, Helsinki, Finland*
*^3^* *Department of Oral and Maxillofacial Radiology, HUS Medical Imaging Center, Helsinki University Hospital, Helsinki, Finland*
*^4^* *Department of Pathology, Helsinki University Hospital, Helsinki, Finland*
*^5^* *Department of Oral and Maxillofacial Diseases, University of Helsinki and Helsinki University Hospital, Helsinki, Finland*
**Objectives**: Conventional ameloblastoma is managed with surgical resection requiring adequate margins, necessitating extensive mandibular resection and osseous reconstruction with significant morbidity. BRAF inhibitors have emerged as a treatment option for BRAF V600E–mutated tumors. We report a case in which neoadjuvant dabrafenib monotherapy produced rapid symptomatic relief and enabled a conservative surgical approach, avoiding mutilating resection.
**Materials and Methods**: A 69-year-old male with plexiform, BRAF V600E–mutated ameloblastoma (MIB-1 index 4%) involving approximately 80% of the mandibular ramus was treated with neoadjuvant dabrafenib 150 mg twice daily. Without neoadjuvant treatment, segmental mandibular resection would have been required. Dabrafenib monotherapy was selected based on case reports demonstrating its efficacy. The patient received two 3-month cycles.
**Results**: Within one week of initiating treatment, the patient reported marked relief of pressure sensation in the jaw and cessation of malodorous oral discharge. The only adverse effect was mild fever managed with ibuprofen. After 3 months, no radiologic size reduction was observed; however, MRI findings were consistent with reduced tumor activity. This is consistent with the slow-growing nature of ameloblastoma, in which osteolytic activity may arrest and ossification of cavity walls may commence before morphologic tumor shrinkage becomes apparent. A biopsy showed minimal residual ameloblastoma with prominent inflammatory infiltrate. The cystic component was fenestrated. After 6 months, ossification had developed around the lesion. The tumor was managed with enucleation and curettage rather than segmental resection, indicating effective downstaging. During 12 months of follow-up, progressive ossification was observed with no recurrence on CBCT or MRI.
**Conclusion**: Neoadjuvant dabrafenib monotherapy produced rapid symptomatic relief and enabled conservative surgery, avoiding mutilating mandibular resection. Further studies are needed to define optimal protocols for BRAF inhibitor use in BRAF V600E–mutated ameloblastoma.

**FA26/173—Oral Surgery**
**Large Sublingual Lipoma with Associated Sialadenitis of the Floor of the Mouth**
Bramadita Satya ^1^ and Andi Budihardja ^1^
*^1^* *Siloam, Kota Tangerang, Indonesia*
**Objectives**: Large sublingual lipomas are rare benign tumors of adipose tissue. This report describes a case associated with chronic sialadenitis and emphasizes the role of MRI and CT in diagnosis and surgical planning.
**Materials and Methods**: A 55-year-old man presented with a slowly enlarging, painless swelling in the left floor of the mouth, causing mild restriction of tongue movement and speech disturbance. Examination revealed a soft, ovoid, dome-shaped mass measuring approximately 3–4 cm, with intact mucosa and no cervical lymphadenopathy. MRI demonstrated a well-circumscribed lesion, homogeneously hyperintense on T1- and T2-weighted sequences, with complete signal suppression on fat-saturated STIR images. CT confirmed uniformly low attenuation (~–100 HU), consistent with fat. The lesion, situated between the mylohyoid and hyoglossus muscles, was excised via a transoral midline approach under general anesthesia, preserving Wharton’s duct and the lingual nerve.
**Results**: The mass appeared intraoperatively as a yellow, encapsulated lobulated tumor and was removed en bloc (3.7 × 1.8 × 2.9 cm). Gross examination showed a soft, lobulated, yellow lesion without hemorrhage or necrosis. Histopathology confirmed a benign lipoma composed of mature adipocytes in lobules separated by thin fibrous septa, with no atypia or lipoblasts, and revealed chronic sialadenitis in adjacent sublingual gland tissue. Recovery was uneventful, with no sensory deficit or salivary dysfunction, and no recurrence at six months follow-up.
**Conclusion**: Sublingual lipomas are exceptionally uncommon and may mimic more frequent floor-of-mouth lesions such as ranula, dermoid/epidermoid cysts, or salivary gland tumors. Characteristic MRI and CT findings enable confident preoperative diagnosis, prevent unnecessary gland resection, and support safe transoral excision with preservation of neurovascular structures. Complete removal of the capsule ensures excellent functional outcomes and a very low recurrence risk.

**FA26/248—Oral Surgery**
**Patient-Assisted Irrigation of Head and Neck Abscess Using Butterfly Scalp Vein: A Case Series**
Kar Tsyeng Ng ^1^, Gerald Joseph ^1^, Sabrina Ali ^1^ and Parmeet Singh ^1^
*^1^* *Department of Oral and Maxillofacial Surgery, Hospital Teluk Intan, Teluk Intan, Malaysia*
**Objectives:** This case series highlights a novel innovation for use in irrigation. Irrigating after incision and drainage (I&D) of head and neck abscesses serves as an essential part of postoperative care. Conventional cannula-based irrigation techniques have several disadvantages, including increased costs, physician dependence, limited access to deeper abscess cavities, and patient discomfort.
**Materials and Methods:** A butterfly scalp vein (BSV) is modified for irrigation. The needle component of the BSV is removed, and the tip end is sutured with silk. Perforations are made along the distal end of the tubing to allow for multidirectional flow of irrigant. After I&D, the deepest part of the abscess cavity is measured, and the BSV is inserted to the corresponding depth to ensure placement of the perforated tip at the deepest portion of the abscess cavity. The tubing is then secured to the adjacent skin using silk sutures to prevent displacement.
**Results:** BSV was used on 30 patients with head and neck abscesses. We found that patients could perform regular self-irrigation in the wards without any issues. Twenty-six patients were discharged home by day three. When continuous irrigation is required, patients are discharged with the device in situ. Two patients had dislodged BSV, which was replaced. The duration of irrigation is also significantly shortened with the use of BSV, from an average of 25 min to less than 5 min.
**Conclusion:** This user-friendly device enables patients to perform self-irrigation, fostering patient empowerment. Due to the BSV’s fixed nature, anyone can perform effective irrigation. This fits the motto: Plug & Flush.

**FA26/325—Oral Surgery**
**Intramucosal Dexamethasone Versus Photobiomodulation After Impacted Mandibular Third Molar Surgery: A Split-Mouth Randomized Controlled Study**
Vyakhya Gupta ^1^, Naqoosh Haidry ^1^, Ameera Salahudheen ^1^, Peeyush Shivhare ^1^ and Ejaz Mokhtar ^1^
*^1^* *All India Institute of Medical Sciences Patna, Patna, India*
**Objectives**: Postoperative pain, facial swelling, and trismus are common sequelae following surgical removal of impacted mandibular third molars. This study aimed to compare intramucosal dexamethasone injection and peri-extraction photobiomodulation therapy in reducing postoperative pain, oedema, and trismus after impacted mandibular third molar surgery.
**Materials and Methods**: An open-label, split-mouth randomized controlled trial was conducted in 30 patients requiring bilateral surgical removal of symmetrically impacted mandibular third molars. A total of 60 surgical sites were analyzed. One side received intramucosal dexamethasone injection, while the contralateral side received peri-extraction photobiomodulation therapy. Postoperative pain, facial swelling, and mouth opening were assessed at baseline, 24 h, 48 h, and 7 days postoperatively. The number of rescue analgesics consumed during the postoperative recovery period was also recorded. Data were analyzed using Mann-Whitney U and Wilcoxon signed-rank tests, with statistical significance set at *p* < 0.05.
**Results**: Both treatment modalities showed significant reductions in postoperative pain, swelling, and trismus over time. Intramucosal dexamethasone demonstrated significantly lower pain scores than photobiomodulation on the 2nd and 7th postoperative days (*p* = 0.000). Swelling scores were significantly lower in the dexamethasone group on the 2nd postoperative day (*p* = 0.009). The total number of rescue analgesics consumed was also significantly lower with dexamethasone (*p* = 0.000). Photobiomodulation showed greater improvement in mouth opening by the 7th postoperative day, although this was not statistically significant (*p* = 0.150).
**Conclusion**: Both intramucosal dexamethasone and peri-extraction photobiomodulation are effective in reducing postoperative morbidity after impacted mandibular third molar surgery. Intramucosal dexamethasone provides superior pain control and reduces analgesic requirement, while photobiomodulation may be a useful alternative when steroid use is contraindicated.

**FA26/331—Oral Surgery**
**The Non-Calcifying Langerhans Cell-Rich CEOT: A Rare Clinicopathologic Report**
Jakkapat Karusan ^1^
*^1^* *Chulalongkorn University, Bankok, Thailand*
**Background**: The Non-calcifying Langerhans cell-rich (NCLC) variant of the Calcifying Epithelial Odontogenic Tumor (CEOT) is an exceptionally rare entity, representing less than 1% of all odontogenic tumors. Unlike the classic Pindborg tumor, which typically affects the posterior mandible of males in their 4th–6th decades, the NCLC variant shows a distinct predilection for the anterior maxilla and Asian females. Its unique presentation, characterized by a complete lack of calcification, poses a significant diagnostic pitfall for clinicians and pathologists.
**Case Description**: A 32-year-old Thai female presented with an asymptomatic palatal depression, a significant clinical suspicion for this variant, along with mobility of the maxillary teeth. Radiographic examination revealed a well-defined, unilocular radiolucency in the maxilla involving the sinus, with evident cortical perforation and root resorption, but notably absent of any radiopaque foci. Histopathological analysis showed islands of polyhedral epithelial cells within a dense stroma of eosinophilic, amyloid-like material, which was confirmed by positive Congo Red staining. However, the classic Liesegang ring calcifications were entirely absent. Immunohistochemistry demonstrated strong and diffuse CD1a positivity, confirming a massive infiltration of Langerhans cells within the tumor islands. This profile is crucial for differentiating NCLC CEOT from more aggressive mimics such as ameloblastoma or amyloid-rich central odontogenic fibroma (AR-COF).
**Management**: Due to the low aggressiveness and lower recurrence rate, less than 5%, of this variant compared to classic CEOT, the patient was managed with conservative surgical enucleation and curettage. This approach ensured complete tumor removal while successfully preserving the palatal shelf and adjacent structures.
**Conclusion**: This case highlights the remarkable morphological diversity of CEOT. Precision diagnosis through histopathology and immunohistochemistry ensures that patients receive optimized care, avoiding unnecessarily radical resection and minimizing morbidity while maintaining excellent long-term outcomes.

**FA26/343—Oral Surgery**
**Giant Compound Odontoma in the Right Mandible: An Extremely Rare Case**
Calenthia Ekawati ^1,2^ and Marjono Wibowo ^1,2^
*^1^* *Dr. Soetomo General Hospital, Airlangga University, Surabaya, Indonesia*
*^2^* *Airlangga University Faculty of Medicine, Surabaya, Indonesia*
**Objectives**: The most common benign odontogenic tumour is the odontoma, accounting for 22% of odontogenic tumours. It is classified into two types: compound and complex. A compound odontoma is typically found in the anterior maxilla. We aimed to report a rare case of compound odontoma in the mandible.
**Materials and Methods**: A 12-year-old boy presented with an enlarged right mandible mass. He felt pain and difficulty with mastication. The physical examination revealed an erythematous mass in the right mandible, sized 3 × 5 × 1 cm, well defined, fixed. The panoramic radiograph showed a radioopaque mass in the right mandible with impacted tooth 46. Head Computed Tomography (CT) scan found a giant compound odontoma in the right mandible and impacted tooth 46. A surgery was done, and there was a cutaneous fistula in the right mandible, a firm, hard, fixed mass sized 6 × 4 × 3 cm in the corpus to the right mandible angle; and molar 46 beneath the odontoma base. His condition improved without any complications.
**Results**: Odontoma is commonly diagnosed in the first 20 years of life without any sex predilection. It is asymptomatic, commonly found on panoramic radiographs. Pain rarely occurs, is commonly due to infection through the area between the odontoma and bone, physical examination may reveal unerupted permanent teeth. Most cases are managed surgically without any complications. The management goals are not to disrupt permanent teeth eruption, prevent bone loss, and prevent cystic change.
**Conclusion**: Giant Compound odontoma may be found in another location and is required for proper management.

**FA26/383—Oral Surgery**
**Management of An Impacted Third Molar in a Mandible with Altered Anatomy Following Angle Reduction Using Sagittal Split Ramus Osteotomy: A Case Report**
Phattanakorn Hongthanakornhirun ^1^
*^1^* *Department of Oral and Maxillofacial surgery, Faculty of Dentistry, Mahidol University, Bangkok, Thailand*
**Abstract body**: Mandibular angle reduction is a widely performed aesthetic procedure for lower facial contouring. However, inadequate preoperative dental evaluation, particularly of impacted third molars, may result in complex secondary surgical challenges. Alterations in mandibular anatomy following contouring procedures can increase the risk of excessive bone loss and iatrogenic fracture during conventional third molar removal. This report describes an alternative surgical approach utilizing sagittal split ramus osteotomy (SSRO).
A 27-year-old female presented with intermittent pain associated with a left mandibular impacted third molar. She had previously undergone mandibular angle reduction and genioplasty at a private hospital. Radiographic evaluation, including panoramic imaging and cone-beam computed tomography, demonstrated a lower left impacted third molar in a mandible with altered anatomy and reduced cortical support, with close proximity to the inferior alveolar canal. Conventional surgical approaches were considered high risk due to potential excessive buccal bone loss and mandibular fracture. A unilateral SSRO was therefore performed to allow controlled access and removal of the impacted tooth while preserving the buccal cortex. Postoperatively, the patient was managed without maxillomandibular fixation, using guiding elastics and a liquid diet. No additional neurosensory deficit was observed, and functional and aesthetic outcomes were satisfactory.
This case highlights the importance of comprehensive dental assessment prior to aesthetic mandibular surgery. Failure to manage impacted third molars preoperatively may lead to technically demanding secondary interventions. In this case, SSRO provided improved surgical access, minimized uncontrolled bone removal, and reduced fracture risk. Although not a conventional indication, SSRO may be considered a viable alternative in selected cases with altered mandibular anatomy.
**Conclusion**: Impacted third molars should be evaluated before mandibular contouring procedures. When postoperative anatomical changes limit conventional access, SSRO may offer a safe and effective alternative while preserving mandibular integrity.

**FA26/385—Oral Surgery**
**Post-Surgical Infection Following Impacted Lower Third Molar Removal: A Retrospective Study in Oral & Maxillofacial Surgery, Hospital Cyberjaya**
Muhammad Syameem Abd Raub ^1^, Suraya Ahmad Nasir ^1^ and Farah Aliya ^1^
*^1^* *Ministry of Health Malaysia, Cyberjaya, Malaysia*
**Objectives**: Impacted third molar removal is one of the most common procedures in oral and maxillofacial surgery. Postoperative infection is a significant complication of third molar surgery, impacting patient recovery and healthcare resources. This study aimed to evaluate the incidence of post-surgical infection following elective lower third molar removal at Hospital Cyberjaya, describe patient demographics, and identify specific clinical and operative risk factors associated with these infections.
**Materials and Methods**: A retrospective, cross-sectional analytical study was conducted using clinical records from January 2024 to August 2024. The study included 320 elective surgical extraction sites, exceeding the minimum calculated sample size of 150 to ensure statistical power. Data on patient demographics (age, gender, ethnicity), smoking status, impaction type, and operative duration were extracted. Statistical associations were determined using odds ratios (OR) and *p*-values to identify significant risk markers.
**Results**: The overall infection rate was 9.1%, primarily presenting as localized swelling and pain. Three primary risk factors were significantly associated with increased infection rates: smoking status, type of lower third molar impaction, and operative duration. Smokers had a significantly higher infection proportion. Distoangular impactions showed the highest infection rate compared to mesioangular and horizontal. Procedures exceeding 30 min were associated with a four-fold increase in infection risk compared to shorter surgeries.
**Conclusion**: The infection rate at this centre sits at the upper end of the internationally reported range. Infection risk is multifactorial and predictable; smoking, anatomical complexity (distoangular impaction), and prolonged operative time are critical indicators. In the absence of prior local data, these findings serve as a critical marker for the department’s clinical direction, highlighting the need for targeted interventions in high-risk cases. Specifically, smoking cessation counselling and specialized operative planning for distoangular impactions are recommended to mitigate the risks associated with prolonged surgical time.

**FA26/392—Oral Surgery**
**Multiple Odontogenic Keratocysts as a Manifestation of Suspected Gorlin–Goltz Syndrome: Clinical, Radiological, and Surgical Perspectives**
Bianda Claresta ^1^, Lilies Dwi Sulistyani ^1^, Mohammad Farid Ratman ^1^, Eky Nasuri ^2^ and Ulfi Putriadi Ghassani ^1^
*^1^* *Department of Oral and Maxillofacial Surgery, Faculty of Dentistry, Universitas Indonesia, Jakarta, Indonesia*
*^2^* *Division of Oral and Maxillofacial Surgery, Cipto Mangunkusumo Hospital, Jakarta, Indonesia*
**Objectives**: This study aims to describe the clinical, radiographic, and histopathological features of a patient with multiple jaw cysts consistent with manifestations of Gorlin–Goltz syndrome (nevoid basal cell carcinoma syndrome), and to discuss the appropriate surgical management and diagnostic significance of this condition.
**Materials and Methods**: A 25-year-old female patient presented with multiple painless swellings on the upper and lower jaws persisting for two years. Clinical examination showed distinct intraoral masses associated with purulent discharge and impacted teeth. Panoramic imaging and CT scan, identified multiple cystic lesions in both jaws, calcification of the falx cerebri, and bifid ribs on chest X-ray were found during radiographic examinations. Surgical enucleation and peripheral ostectomy of multiple cysts, along with extraction of involved teeth, were performed under general anesthesia. For a definite diagnosis, excised specimens were evaluated histopathologically.
**Results**: Histopathological analysis confirmed odontogenic keratocyst (OKC) in four cystic lesions and a radicular cyst in one lesion. The presence of multiple OKCs, calcified falx cerebri, and bifid ribs fulfilled major diagnostic criteria for Gorlin–Goltz syndrome. Postoperative recovery was uncomplicated, with no recurrence observed during a six-month follow-up period.
**Conclusion**: This case highlights the importance of recognizing multiple odontogenic keratocysts as a potential manifestation of Gorlin–Goltz syndrome. Comprehensive diagnostic assessment, including evaluation of radiographic and other manifestations, is crucial for early identification and appropriate management. Surgical enucleation combined with peripheral ostectomy remains an effective treatment to minimize recurrence. A multidisciplinary approach and long-term monitoring are advised for optimal patient outcomes.

**FA26/455—Oral Surgery**
**Sublingual Gland Adenoid Cystic Carcinoma Mimicking Sialolithiasis: A Diagnostic Challenge**
Mohd Fazrin Hussin Tajul Ariffin ^1^, Shobina Sivanganam ^1^, Rosfatihah Ab Malek ^1^ and Aezy Noorazah Omar ^1^
*^1^* *Hospital Sultan Idris Shah Serdang, KAJANG, Malaysia*
**Introduction**: Sialolithiasis is the primary cause of swelling in the major salivary glands. It commonly involves the submandibular & parotid gland, whereas sialolithiasis of the sublingual gland is considered extremely rare. In contrast to the incidence of sialolithiasis, salivary gland neoplasms occur more frequently in the sublingual gland, followed by the submandibular and parotid glands. Among these neoplasms, adenoid cystic carcinoma (ACC) is a notable entity, characterized by its propensity for perineural invasion and metastases.
**Case Report**: In this report, we present a case initially diagnosed as sublingual gland sialolithiasis based on clinical presentation and calcified mimicker in computed tomography (CT) findings. However, subsequent surgical excision and histopathological examination established a diagnosis of ACC. The diagnostic and therapeutic process was particularly challenging due to its rarity and atypical presentation of this condition.
**Conclusion**: This case highlights the potential for diagnostic ambiguity when calcifications are present within salivary gland lesions, as certain malignancies, including ACC, may occasionally exhibit dystrophic calcification, thereby mimicking sialolithiasis on imaging studies. Hence, it is of utmost importance to maintain a high index of clinical suspicion, particularly in lesions involving the sublingual gland, to differentiate between neoplastic processes and true sialolithiasis.

**FA26/476—Oral Surgery**
**Retromolar Triangle Anesthesia as an Alternative to the Inferior Alveolar Nerve Block: A Prospective Clinical Study**
Ciprian Roi ^1^, Alexandra Roi ^2^, Ioana Rivis ^3^, Adrian Nicoara ^1^, Andrei Uritu ^1^ and Mircea Rivis ^1^
*^1^* *University Clinic of Anesthesiology and Oral Surgery, Research Center of Dento-Alveolar Surgery, Anesthesia and Sedation in Dental Medicine, “Victor Babes” University of Medicine and Pharmacy, Eftimie Murgu Sq. No. 2, 300041, Timisoara, Romania*
*^2^* *University Clinic of Oral Pathology, Multidisciplinary Center for Research, Evaluation, Diagnosis and Therapies in Oral Medicine, “Victor Babes” University of Medicine and Pharmacy, Eftimie Murgu Sq. No. 2, 300041, Timisoara, Romania*
*^3^* *Rivis Dental Clinic, Dumbravita, Romania*
**Objectives**: Achieving reliable mandibular anesthesia remains one of the most challenging aspects of dental practice. The conventional inferior alveolar nerve block (IANB) carries a well-documented failure rate and potential complications including hematoma, intravascular injection, and nerve injury. This study aimed to assess the clinical effectiveness of the retromolar triangle approach as an alternative nerve block technique for inferior molar extraction, evaluating both its success rate across the target nerve branches and the duration of the achieved anesthesia.
**Materials and Methods**: A prospective study was conducted on 50 adult patients, presenting for extraction of one or more inferior molars. After applying standardized inclusion and exclusion criteria, all participants received anesthesia via the retromolar triangle approach using articaine 4% with 1:100,000 epinephrine. Anesthetic efficacy for the inferior alveolar nerve (IAN) was assessed objectively with an electric pulp tester and confirmed through the mental nerve territory using a periodontal probe. The buccal and lingual nerve blocks were evaluated subjectively with the Visual Analog Scale (VAS). Anesthesia duration was recorded for all successful cases.
**Results**: Effective IAN block was achieved in 64% of cases. Lingual nerve anesthesia was obtained in 46% of cases, while buccal nerve block was recorded in 22%. Among cases where anesthesia was successful, the mean duration was 113.1 min (overall mean 72.4 min). Statistical analysis demonstrated that the VAS mental score was a highly significant predictor of anesthesia effectiveness (*p* < 0.001), explaining approximately 89% of the variance in post-anesthesia pulp test values.
**Conclusion**: The retromolar triangle technique represents a clinically viable alternative to conventional IANB, particularly for patients in whom deep block anesthesia is contraindicated—such as those with bleeding disorders or anticoagulant therapy. Its accessible injection site, low vascular risk, and acceptable anesthesia duration make it a valuable addition to the clinician’s armamentarium for mandibular pain management.

**FA26/481—Oral Surgery**
**Surgical Management of an Infected Complex Odontoma of the Mandible: A Case Report**
Dhira Rama Haidar ^1^, Eky Nasuri ^2^ and Dwi Ariawan ^1^
*^1^* *Department of Oral and Maxillofacial Surgery, Faculty of Dentistry Universitas Indonesia, Jakarta, Indonesia*
*^2^* *Cipto Mangunkusumo Hospital, Jakarta, Indonesia*
**Objectives**: Odontomas are among the most prevalent benign odontogenic tumors, typically characterized by slow growth and an asymptomatic nature. However, complications such as secondary infection can lead to significant clinical symptoms, including pain and swelling, necessitating prompt surgical intervention. This report describes the clinical presentation and surgical management of an infected complex odontoma in the posterior mandible, while addressing the diagnostic challenge of differentiating it from osteomyelitis.
**Materials and Methods**: A 29-year-old female patient presented with a persistent 1.5 cm mass in the region of tooth 48, accompanied by buccolingual expansion and pus discharge. Radiological imaging revealed a radiopaque mass with surrounding bone lysis. A key diagnostic challenge was distinguishing the lesion from osteomyelitis; while both conditions can present with cortical erosion, sequestration, and suppuration, the presence of a well-defined, highly radiopaque mass (calcified dental tissues) within the lytic area primarily suggested an infected odontoma rather than the more diffuse, “moth-eaten” appearance typical of chronic osteomyelitis.
**Results**: The patient was diagnosed with a suspected infected complex odontoma. The treatment plan involved an excisional biopsy and thorough debridement performed under general anesthesia. Intraoperatively, the lesion was enucleated, and the infected site was debrided to eliminate the source of suppuration. Postoperative management included a regimen of intravenous antibiotics (Ceftriaxone) and analgesics to support healing.
**Conclusion**: This case underscores the importance of considering infected odontoma in the differential diagnosis of mandibular swellings. Distinguishing it from osteomyelitis is critical, as the former requires complete enucleation of the calcified entity to resolve the infectious focus, whereas osteomyelitis management focuses more broadly on bone sequestrectomy and prolonged antimicrobial therapy. Early surgical intervention ensures a favorable prognosis and prevents extensive bone destruction.

**2FA26/1038—Oral Surgery**
**Mandibular Resection of Cemento-Ossifying Fibroma: Report of Serial Cases**
Muhammad Luthfi ^1^, Lilies Dwi Sulistyani ^1^, Wenny Yulvie ^2^ and Eky Nasuri ^2^
*^1^* *Universitas Indonesia, Depok, Indonesia*
*^2^* *Rsupn Dr. Cipto Mangunkusumo, Jakarta, Indonesia*
**Objective**: Cemento-ossifying fibroma (COF) is a rare benign neoplasm of the jaw, characterized by replacement of normal bone tissue by fibrous connective tissue with varying amount of bone formation as well as cementum-like material deposition within the lesion. This lesion typically has slow growth rate and oftentimes presented as asymptomatic expansile swelling on the jaw. Enucleation and curettage were regarded as the definitive therapy for smaller lesions due to its well demarcated margin, however larger lesions may require more radical approach such as segmental resection to ensure complete removal and prevent recurrence. In this article, we will report successful treatment of Cemento-ossifying fibroma cases.
**Materials and Methods**: We report a case series of 3 patients diagnosed with COF. All presented cases occurred asymptomatically in the mandible, presenting as slow growing mass with clear border and firm consistency. CT Scan examination revealed fibrous lesion on tooth bearing sites in the mandible, expanding lingually and inferiorly resulting in thin bony cortex surrounding the lesion.
**Results**: All patients underwent segmental resection with immediate reconstruction using osteoseptocutaneous free fibular flap on the defect site. Histology examination confirmed the diagnosis. Post-operative follow-up of the patients show good adaptation of the flap and no sign of recurrence.
**Conclusion**: COF is a form of rare benign tumour of the jaw which could be treated with enucleation and curettage of the lesion. On larger lesion however, surgical resection is the treatment of choice to ensure complete removal of the tumour. Immediate reconstruction with free fibular flap is also recommended to restore function and aesthetic after resection.

**2FA26/1062—Oral Surgery**
**Evaluation of Multidimensional Post Operative Recovery Following Minimally Invasive Third Molar Extraction**
Mausumi Iqbal ^1^, Mezbah Azeez ^1^, Umme kulsum ^2^ and Sumaya Mostofa ^3^
*^1^* *Apollo clinic, JMI Specialized Hospital, Tooth Care, Facio-Maxillary Reseach & Development Hub, Dhaka, Bangladesh*
*^2^* *World University Bangladesh, Dhaka, Bangladesh*
*^3^* *Bangladesh University of Professionals, Tooth Care, Facio-Maxillary Reseach & Development Hub, Dhaka, Bangladesh*
**Objectives**: To evaluate overall postoperative recovery and patient-reported outcomes following minimally invasive third molar extraction.
**Materials and Methods**: This prospective longitudinal clinical study included 39 patients undergoing minimally invasive surgical extraction of impacted third molars. Postoperative recovery was evaluated using pain recovery, functional recovery, psychosocial recovery, daily life recovery, and clinical recovery domains. Assessments were conducted preoperatively, on postoperative day 3 (POD3), and postoperative day 7 (POD7). Pain was assessed using the Visual Analogue Scale (VAS), while healing was evaluated using the Modified Likert Type Psychometric Scale (MLTPS).
**Results**: The mean VAS pain score decreased from 5.1 ± 2.3 preoperatively to 3.2 ± 1.9 on POD3 and 0.0 ± 0.0 on POD7. Maximum mouth opening decreased transiently on POD3 (33.2 ± 6.6 mm) but improved to near baseline levels by POD7 (41.3 ± 6.3 mm). Swallowing discomfort reduced from 69.2% on POD3 to 15.4% on POD7, while interference with daily activities decreased from 25.6% to 5.1%. No postoperative infections were observed. The MLPTS score improved from 4.3 ± 1.9 on POD3 to 1.8 ± 0.4 on POD7, indicating functional recovery.
**Conclusion**: Minimally invasive third molar extraction demonstrated favorable postoperative recovery characterized by progressive pain reduction, functional improvement, satisfactory healing and minimal postoperative morbidity. The technique may provide beneficial patient-centered recovery outcomes following third molar surgery.

**2FA26/1084—Oral Surgery**
**Mandibular Defect and Iliac Grafting—Complications and Limitations Faced in a Developing Country: A Clinical Case**
Althea Bumpu ^1^
*^1^* *Port Moresby Jacksons International, Papua New Guinea Dental Association, National Capital District Provincial Health Authority-Dental Division, Port Moresby, Papua New Guinea*
**Objectives**: To understand the complications of facial trauma and jaw fractures. To know the significance of bone repair and remodeling. To be able to learn the importance of mandibular reconstruction using bone grafting and its limitations in a developing nation.
**Materials and Methods**: This case report is an analysis of a mandibular defect from a male of 36, diagnosed with osteonecrosis and was managed with surgical intervention in the main operating theatre of Port Moresby General Hospital, Papua New Guinea (PNG). The reconstruction of the jaw bone was done using iliac crest grafting. History of the case has revealed physical assault to the face leaving behind a mal-union fracture of the jaw. Patient had initial treatment of maxillo-mandibular fixation. After a period of negligence to follow ups and non-compliant to post-operative instructions, patient returned to the clinic with an unsuccessful healing and was diagnosed with osteonecrosis.
**Results**: Skull radiographs showed bilateral radiolucent space on the body of mandible, left and right with radio-opaque screws hanging loose. Mandibular reconstruction management approach was taken. Patient was put under general anesthesia and sedation while a surgical incision was made. The necrotic bone was removed and an auto-graft of the patient’s iliac crest was embedded into the mandibular defect space. It was screwed tight to the mandible with titanium screws and plates. A final close up was done with tight sutures of the soft tissues.
**Conclusion**: Surgery was successful. However, a complete rehabilitation of implant and crowns where not done due to resource and specialist limitations in a developing nation. An alternative plan was to construct removable acrylic dentures for the patient. It is recommended that further studies be conducted and relevant data be collected to better analyse the success rate of post-operative mandibular fractures and defects.

**2FA26/1135—Oral Surgery**
**Comparison of Intraoperative Bleeding During Excision of Benign Soft Tissue Oral Lesions Using Scalpel and Diode Laser**
Dr. Waqas Javied Malik ^1^ and Dr. Quratul-Ain Zafar ^1^
*^1^* *Shaheed Zulfiqar Ali Bhutto University, Islamabad, Pakistan*
**Objective:** The objective of this study was to compare the grade of intraoperative bleeding during excision of benign soft tissue oral lesions using scalpel and diode lasers according to Boezart et al. bleeding scale.
**Methodology:** A single blinded randomized comparative trial was conducted over a span of one year at department of Oral and Maxillofacial Surgery, Shaheed Zulfiqar Ali Bhutto Medical University, PIMS Islamabad. A total of 128 patients with benign oral soft tissue lesions were selected and randomly divided into two groups Group A Scalpel and Group B Diode laser. Relevant history and clinical examination were carried out and recorded. Excision was carried out under Local anesthesia using diode laser and scalpel. The intraoperative bleeding grades were recorded in accordance with the criteria defined by Boezaart et al. attached as Annexure A.
**Results:** The grade of bleeding between both groups was compared. A *p* value ˂ 0.05 was considered significant at 95% confidence level. Data was presented in the form of tables and graphs. The results showed that 66 male and 62 female patients. The average age of patients in group A was 32.38 and group B was 35.19. The most common site of lesion was lower lip in both groups. The average size of lesion in group A was 0.84 cm and group B was 0.82 cm. There was statistically significant difference in bleeding grade between two groups with group B (Diode laser) showing significantly less intraoperative bleeding compared with group A (Scalpel group).
**Conclusion:** It was concluded that diode laser effectively reduces intraoperative bleeding during excision of benign oral soft tissue lesions.
**Key words:** bleeding, diode lasers, hemorrhage, oral, surgery

**2FA26/1149—Oral Surgery**
**Retrospective Analysis of Recurrence Rate of Odontogenic Keratocysts (OKCs) in Oman (2013–2022)**
Sara Al Rasbi ^1,2^ and Salah Al Din Al Azri ^3^
*^1^* *Oral and Maxillofacial Surgery Department, Al Nahdha Hospital, Ministry of Health, Oman*
*^2^* *Oman Medical Speciality Board, Muscat, Oman, Muscat, Oman*
*^3^* *Oral and Maxillofacial Surgery Department, Al Nahdha Hospital, Ministry of Health, Oman, Bawshar, Oman*
**Objectives**: This study aimed to (1) determine the recurrence rate of OKCs treated at Al Nahdha Hospital, Oman (2013–2022); (2) investigate the relationship between treatment modalities, lesion characteristics, and patient factors with recurrence; and (3) qualitatively interpret these quantitative associations to unravel clinical insights and propose evidence-based recommendations for reduce recurrence risk within the Omani healthcare.
**Methods**: A retrospective cohort study included 42 histopathologically confirmed OKC cases from Al Nahdha Hospital. Data on patient demographics, lesion characteristics (location, size, radiographic features), treatment modalities (marsuplisation vs. surgical enucleation), and recurrence status were collected. Statistical analyses (descriptive statistics, independent samples *t*-tests, Chi-square tests, binary logistic regression) identified significant associations and predictors. Interpretation focused on clinical meaning and implications.
**Results**: The overall OKC recurrence rate was 23.8% (10/42; 95% CI: 12.05–39.45%), consistent with international reports [1,2]. Significant associations were found with lesion size (*p* = 0.009), surgical approach (*p* = 0.002), and adjunct use (*p* = 0.005). Radical surgical approaches demonstrated a significantly lower recurrence rate (5.3%) compared to conservative methods (39.1%). Smaller lesions (mean 2.29 cm) were paradoxically associated with a higher recurrence rate than larger lesions (mean 3.36 cm). The use of surgical adjuncts correlated with a higher recurrence rate (50.0% vs. 10.7% without adjuncts), suggesting selection bias for more aggressive cases. A binary logistic regression model achieved 92.7% classification accuracy.
**Conclusion**: OKCs in Oman exhibit a significant recurrence rate, with surgical approach and lesion size being critical quantitative predictors. Qualitative interpretation suggests surgical intervention is paramount. These insights are essential for refining local treatment strategies, emphasizing meticulous surgical planning, and ensuring long-term patient follow-up to reduce recurrence and improve outcomes.

**2FA26/1189—Oral Surgery**
**The Position of Inferior Alveolar Canal in Relation to the Third Molar in a Selected Malay Population**
Nor Adilah Harun ^1^
*^1^ International islamic University Malaysia, Kuantan, Malaysia*
**Objectives**: The Inferior Alveolar Nerve (IAN) is an important vital structure in the mandible. The close proximity of the inferior alveolar canal (IAC) to the mandibular third molar (M3) has become a great concern prior to surgical removal of M3 due to the reported complications with regard to the content of this canal. We aimed to investigate its position in relation to the M3 roots in different impactions and to assess the complications caused by this relationship in a selected Malay population.
**Materials and Methods**: 205 Orthopantomogram (OPG) and cone beam computed tomography (CBCT) radiographs of the patients attending the polyclinic were selected and assessed. Statistical analysis was done using Javascript-STAR software.
**Results**: 42.4% of M3 were mesioangularly impacted and 73.2% had an interruption of the IAC white line in OPG. CBCT findings showed the majority of IAC were located buccal to the roots of M3 (55.6%) and type 2 (the canal superimposed less than half of the root structure, and disappearance of cortication of the canal) overlap of the root tip to IAC (32.2%). IAN injury (0.8%) following total surgical removal of the lower third molar tyrawas reported in a vertically impacted M3 with radiographic signs of darkening of the root and interruption of the white line; lingually positioned IAC with type 1 (the canal superimposed more than half of the root structure and disappearance of cortication of the canal) extent of root tip overlap.
**Conclusion**: The selected Malay population has mesioangular impaction of M3 with buccally positioned IAC and type 2 extension to its root. There was no significant relationship between radiographic risk predictor signs in OPG with the position of IAC in CBCT. Preoperative CBCT remains a gold standard assessment for M3 removal as OPG alone cannot predict post-operative complications.

**2FA26/1198—Oral Surgery**
**Clinicoradiographic Features Guiding Surgical Management of expansile Maxillary Lesions in Children—A Five-Case Series**
Bramasto Purbo Sejati ^1^, Yosaphat Bayu Rosanto ^1^ and Sepdhyo Nugroho ^1^
*^1^* *Universitas Gadjah Mada, Yogyakarta, Indonesia*
**Objectives**: Expansile maxillary lesions in children present unique diagnostic and therapeutic challenges because lesion progression may disrupt craniofacial growth, tooth eruption, and dentofacial development. Although these lesions frequently share similar clinical manifestations, their biological behavior varies considerably. This case series highlights the role of clinicoradiographic assessment in differentiating pediatric maxillary lesions and guiding individualized surgical management.
**Materials and Methods**: Five pediatric patients aged 6 months to 13 years presented with progressive unilateral maxillary enlargement, facial asymmetry, and minimal or absent pain. Clinical findings were largely similar, including cortical expansion, vestibular obliteration, delayed tooth eruption, and disturbances of developing dentition. Panoramic radiography and computed tomography revealed distinct lesion characteristics, including well-defined radiolucencies associated with unerupted teeth, maxillary sinus involvement, displacement of developing teeth, cortical expansion, bony erosion, and expansile fibro-osseous changes. Histopathological examination confirmed three dentigerous cysts, one odontogenic myxoma/myxofibroma, and one juvenile ossifying fibroma.
**Results**: Radiographic findings were pivotal in determining treatment strategies. Large cystic lesions involving developing dentition were managed with marsupialization followed by definitive surgery, localized cystic lesions underwent enucleation, while more aggressive lesions required excision or partial maxillectomy. Follow-up ranging from 1 to 4 years demonstrated satisfactory healing, preservation of facial symmetry and oral function, continued dentofacial development, and no evidence of recurrence.
**Conclusion**: Pediatric expansile maxillary lesions may present with similar clinical features despite representing distinct pathological entities. Clinicoradiographic assessment provides critical information regarding lesion extent, biological behavior, and potential impact on craniofacial development, thereby guiding appropriate surgical intervention. Individualized management based on these findings can achieve favorable long-term functional and esthetic outcomes while preserving growth and dental development.

**2FA26/1240—Oral Surgery**
**Paradoxical Somatosensory Disturbances Following Postoperative Vitamin B Supplementation After Third Molar Surgery: A Two-Patient Case Series**
Wan Nor Amira Wan Ahmad Abd. Nasir ^1,2^, Juliana Khairi ^1,2^, Sherrie Chong ^1,2^ and Mukhriz Hamdan ^1,2^
*^1^* *Hospital Tengku Ampuan Rahimah, Klang, Malaysia*
*^2^* *Ministry of Health, Putrajaya, Malaysia*
**Introduction**: Postoperative inferior alveolar nerve (IAN) trauma is a known complication of mandibular third molar surgery. Clinicians routinely prescribe neurotropic Vitamin B complexes to accelerate regeneration; however, certain formulations introduce neurotoxic risks. This case series compares outcomes of two patients with postoperative IAN trauma managed with standard B-complex (Neurobion) versus pure mecobalamin.
**Case Description**: Patient 1: A 39-year-old male experienced persistent hypoesthesia of the left lower lip following surgical removal of impacted tooth #38. He was prescribed with standard dose Neurobion (Vitamins B1, B6, and B12). At week two, he reported a paradoxical development of severe localized hyperesthesia (hypersensitivity).
Patient 2: A 22-year-old male presented with a similar surgical profile and postoperative left IAN hypoesthesia following surgical removal of tooth #38. He was prescribed pure Mecobalamin (500 mcg, three times daily). Patient 2 experienced spontaneous paresthesia (tingling) starting on post-op week 2.
**Discussion**: While active B12 (mecobalamin) supports myelination, excessive Vitamin B6 (pyridoxine) found in combination supplements can accumulate and induce sensory ganglionopathy, mimicking or worsening surgical trauma. Discontinuation of Neurobion in Patient 1 led to the stabilization and eventual reversal of the hypersensitivity, confirming supplement-induced neurotoxicity rather than secondary surgical mechanical trauma.
**Conclusion**: Over-supplementation with combination neurotropic vitamins can paradoxically exacerbate post-surgical somatosensory disturbances. Clinicians should consider highly targeted monotherapy formulations like Mecobalamin over high-dose B-complex combinations. Furthermore, clinicians must implement shorter patient monitoring intervals to actively track neurosensory changes, ensuring favorable outcomes and the early detection of any supplement-induced somatosensory disturbances. To achieve this, we recommend a strict clinical framework: mandatory pre-operative medication reconciliation to prevent toxic compounding, proactive patient education regarding early neurotoxic warning signs (such as a shift from numbness to hyperesthesia), and a prompt cessation protocol that immediately pivots to monotherapy if symptoms escalate within the first four weeks post-surgery.

**2FA26/1286—Oral Surgery**
**Decompression as a Conservative Strategy for Extensive Odontogenic Cystic Lesions: A Case Series**
Marwa Abdalgaffar ^1^
*^1^* *Nhs Fife, Kirkcaldy, UK*
**Background**: Extensive odontogenic cysts, such as dentigerous cysts and odontogenic keratocysts, can compromise adjacent vital structures and increase the risk of pathological fracture when treated with primary enucleation. Decompression offers a conservative alternative by reducing intracystic pressure, promoting bone regeneration, and enabling gradual lesion shrinkage.
**Methods**: This case series evaluates patients with large odontogenic cystic lesions initially managed by decompression. A drainage stent was placed to maintain cyst patency, and patients were instructed to perform daily saline irrigation. Lesion progression and bone regeneration were monitored using serial radiographic imaging.
**Results**: All cases showed significant reduction in cyst size, accompanied by progressive centripetal bone formation. Adjacent anatomical structures were preserved in all patients. When required, secondary enucleation was performed with reduced surgical complexity and minimal complications.
**Conclusion**: Decompression is an effective, minimally invasive, and bone-preserving approach for managing extensive odontogenic cystic lesions. It reduces lesion volume, lowers surgical morbidity, and facilitates safer definitive treatment, supporting its use as a first-line strategy in appropriately selected cases.

**2FA26/1295—Oral Surgery**
**Can Artificial Intelligence Predict Local Anesthesia Failure? Towards Precision Anesthesia in Oral Surgery**
Syarifah Nova Amiza Zam ^1,2^
*^1^* *RSUD Sultan Fatah (Sultan Fatah Public Hospital), Demak, Demak, Indonesia*
*^2^* *Universitas Muhammadiyah Semarang, Faculty of dentistry, Semarang, Indonesia*
**Background**: Local anesthesia failure remains a significant problem in oral and maxillofacial surgery, particularly during mandibular procedures and in patients with inflammatory conditions. The multifactorial etiology, involving anatomical, biological, psychological, and technical factors, limits the accuracy of conventional risk assessments.
**Aim**: To investigate the potential role of artificial intelligence (AI) in predicting local anesthesia failure and its implications for precision anesthesia in oral surgery.
**Methods**: A narrative review was conducted of recent evidence regarding AI applications in dentistry, predictive analytics, radiomics, and anesthesia-related clinical decision support. Emerging technologies capable of integrating clinical, radiographic, and patient-specific data were evaluated. Results: Machine learning algorithms have shown promising performance in identifying complex patterns related to treatment outcomes and procedural complications. In oral surgery, AI has the potential to integrate demographic, clinical, radiographic, and behavioral variables to estimate an individual’s risk of anesthetic failure. Advanced analysis of CBCT images can further improve the identification of anatomical factors associated with failed nerve blocks. Such predictive models could support personalized anesthesia strategies, including the choice of anesthetics, injection techniques, and additional analgesic measures.
**Conclusion**: AI-driven predictive models could represent a future paradigm shift from treating anesthetic failure to preventing it through individualized risk assessment. Although clinical validation and regulatory challenges remain, AI has the potential to advance precision anesthesia, improve patient comfort, and optimize surgical outcomes in oral and maxillofacial surgery.
**Keywords**: Artificial intelligence; Local anesthetic failure; Precision anesthesia; Machine learning; Oral surgery; Predictive analytics

**2FA26/1297—Oral Surgery**
**Jacob’s Disease: Two Cases Report**
Pamela Pichel ^1^, Sergio Recio ^1^ and Sofía Cedre ^1^
*^1^* *Hospital Municipal Dr. Leónidas Lucero, Bahía Blanca, Argentina*
**Objectives**: The objective of this work is to describe the diagnostic approach and surgical treatment of two patients with coronoid process osteochondroma, emphasizing the role of computed tomography in diagnosis and planning, as well as the functional results achieved after intraoral coronoidectomy and early physiotherapy.
**Materials and Methods**: A retrospective analysis of two female patients with progressive trismus secondary to coronoid process lesions were evaluated clinically and by computed tomography (CT). Surgical treatment consisted of intraoral coronoidectomy through a vestibular approach. Histopathological analysis was performed in all cases. Early postoperative physiotherapy was instituted to restore mandibular function and mouth opening.
**Results**: Both patients underwent surgical treatment through an intraoral vestibular approach with coronoidectomy. In the patient with severe limitation of mouth opening, nasotracheal fiberoptic intubation was performed. Resected specimens were submitted for histopathological examination. Postoperatively, both patients underwent early physiotherapy and temporomandibular joint rehabilitation to improve mandibular mobility and mouth opening.
**Conclusion**: Although rare, osteochondroma of the coronoid process should be considered in the differential diagnosis of progressive trismus. CT imaging is the gold standard for diagnosis and surgical planning. Surgical resection through an intraoral approach combined with early physiotherapy provides predictable functional recovery, significant improvement in mouth opening, and a low rate of recurrence.

**FA26/190—Orthognathic Surgery**
**Quantitative Assessment of Surgical Accuracy After Orthognathic Surgery: A Three-Dimensional Analysis**
Nadav Grinberg ^1^, Ariel Pokhojaev ^1^, Clariel Ianculovici ^1^, Shlomi Kleinman ^1^ and Oren Peleg ^1,2^
*^1^* *Tel-Aviv Sourasky University Medical Center, Tel-Aviv, Israel*
*^2^* *The Maurice and Gabriela Goldschleger School of Dental Medicine, Grey Faculty of Medical & Health Sciences, Tel Aviv University, Tel-Aviv, Israel*
**Objectives**: Quantitatively assess surgical accuracy of three-dimensional (3D) virtual surgical planning in orthognathic surgery.
**Materials and Methods**: This retrospective study assessed patients who underwent orthognathic surgery at a tertiary medical center between 2022 and 2025. Three-dimensional skeletal assessment was performed in 3D Slicer (version 5.9.0) using standardized anatomical landmarks on the maxilla and mandible. Grayscale-based spatial registration and rigid transformation based on the surgically unaffected Frankfurt plane were applied to determine maxillary and mandibular positional transformations and to compare the planned and achieved postoperative positions using the Kabsch-Umeyama algorithm without scaling. Surgical accuracy was quantified by translational discrepancies (mm) and rotational discrepancies (degrees) along the three spatial axes. Descriptive and comparative statistical analyses were then performed.
**Results**: A total of 43 patients were included with 23 females and 20 males at mean age of 26.4 (17–52) years. At the post-op time point, translational accuracy was higher in the maxilla than in the mandible. Maxillary mean, RMS, and maximum errors were 1.80 ± 1.15 mm, 1.91 ± 1.16 mm, and 2.63 ± 1.41 mm, compared with 2.99 ± 1.91 mm, 3.10 ± 1.90 mm, and 3.96 ± 2.14 mm for the mandible. These inter-jaw differences were statistically significant. Overall, the rotational error was more similar between the jaws. Total rotation error was 3.23 ± 2.21° for the maxilla and 3.63 ± 2.04° for the mandible, while the main between-jaw difference remained translational rather than rotational. Axis-specific analysis showed that the largest rotational component in both jaws was around the X axis, with greater mandibular Z-axis rotational error.
**Conclusion**: Digital planning demonstrated good overall correlation with the post-op skeletal position. Maxillary accuracy was higher and consistent than mandibular accuracy. These findings support the clinical reliability of the workflow, while highlighting the need for cautious interpretation of early post-op mandibular precision.

**FA26/206—Orthognathic Surgery**
**Is Dexamethasone Associated with Postoperative Surgical Site Infections in Orthognathic Surgery—A Retrospective Study**
Evelina Metsaranta ^1^, Sakari Kettunen ^1^, Johanna Snäll ^1^
*^1^* *University of Helsinki, Helsinki University Hospital, Helsinki, Finland*
**Objectives**: This study aimed to evaluate the association between perioperative dexamethasone administration and the risk of postoperative surgical site infections (SSI) in orthognathic surgery (OS), and to identify patient- and procedure-related variables contributing to the development of postoperative SSIs.
**Materials and Methods**: A retrospective single-center cohort study was conducted at Helsinki University Hospital, including patients ≥ 18 years undergoing bilateral sagittal split osteotomy (BSSO), LeFort I or bimaxillary surgery. The primary outcome variable was SSI within 4 weeks postoperatively. The main predictor variable was total perioperative dexamethasone dose. Additional variables included age, sex, BMI, smoking, alcohol and/or drug abuse, immunosuppressive medication or disease, type of surgery, bad split-complication, and the type of osteosynthesis. Statistical analyses were performed to assess associations between the predictor variables and the outcome.
**Results**: A total of 16 patients (3.8%) developed an SSI, of whom 68.75% were male. Patients with immunosuppressive medication were 4.7-times (*p* = 0.0067) more likely to have an infection, and smoking was also associated with 3.6-fold increase (*p* = 0.0076). Most infections occurred following BSSO (81.3%) and were more common with standard fixation plates compared to custom plates. Two patients (12.5%) required drain placement and one (6.3%) required reoperation. Preliminary analysis did not demonstrate a clear independent association between dexamethasone dose and SSI risk.
**Conclusion**: SSI is a clinically important complication following orthognathic surgery. In this cohort, 3.8% of patients had an SSI requiring pharmacological or surgical intervention. Infections were more frequent in procedures involving the mandible and were associated with smoking and immunosuppression. No increased SSI risk was observed with higher dexamethasone administration. To reduce SSIs, careful patient selection and risk assessment are crucial.

**FA26/284—Orthognathic Surgery**
**Knowledge, Perceptions, and Referral Patterns Regarding Orthognathic Surgery Among Dental Practitioners in Indonesia: A Cross-Sectional Survey**
Nissia Ananda ^1,2^, Muhammad Kamil Hassan ^1^, Maudina Dwi Heriasti ^1,3^, Firdaus Hariri ^1^ and Lindawati S Kusdhany ^4^
*^1^* *Department Oral and Maxillofacial Clinical Sciences, Faculty of Dentistry, Universiti Malaya, Kuala Lumpur, Malaysia*
*^2^* *Dental Unit, University of Indonesia Hospital, Universitas Indonesia, Depok, West Java, Indonesia*
*^3^* *Oral & Maxillofacial Surgery Department, Cipto Mangunkusumo Hospital, Jakarta, Indonesia*
*^4^* *Department of Prosthodontics Faculty of Dentistry Universitas Indonesia, Jakarta, Indonesia*
**Objectives**: This study aimed to evaluate Indonesian dental practitioners’ clinical experience, referral preferences, and perceptions regarding the indications, expected outcomes, and misconceptions of orthognathic surgery.
**Materials and Methods**: A cross-sectional online survey was conducted among dental practitioners in Indonesia. The questionnaire assessed participants’ professional background, experience with orthognathic surgery patients, referral preferences for dentofacial deformity, obstructive sleep apnea (OSA), and temporomandibular disorders (TMD), as well as their perceptions of orthognathic surgery outcomes and indications. Descriptive statistics were used to summarize the findings.
**Results**: A total of 132 Indonesian dental practitioners participated in this study. Most respondents were general dentists, followed by oral and maxillofacial surgeons. Clinical exposure to orthognathic surgery was low, with the majority reporting fewer than five orthognathic surgery patients in the past five years. Referral preferences varied according to clinical condition. Dentofacial deformity or facial asymmetry was mainly referred to oral and maxillofacial surgeons or orthodontists, while suspected OSA was mostly referred to ENT specialists. For TMD, respondents commonly referred to prosthodontists and oral and maxillofacial surgeons. Most respondents recognized the potential benefits of orthognathic surgery for mastication, speech, long-term dental health, psychosocial well-being, TMD related to asymmetry, airway or breathing function, and OSA management. However, misconceptions remained, particularly regarding insurance coverage and the need for postoperative jaw wiring.
**Conclusion**: Indonesian dental practitioners demonstrated generally positive perceptions of the benefits of orthognathic surgery, despite limited clinical exposure. Persistent misconceptions and variation in referral preferences highlight the need for improved education, clearer referral pathways, and multidisciplinary collaboration.

**FA26/290—Orthognathic Surgery**
**Soft Tissue Movement Algorithm to Predict Orthognathic Aesthetic Result**
Andrea Biglio ^1^, Giacomo De Riu ^1^, Luigi Vaira ^1^
*^1^* *University of Sassari Hospital, Sassari, Italy*
**Objectives**: To assess the accuracy and clinical reliability of a soft tissue movement algorithm in predicting postoperative aesthetic outcomes in patients undergoing orthognathic surgery.
**Materials and Methods**: A sample of patients requiring orthognathic surgery for dentofacial deformities was analyzed using a digital planning workflow. Preoperative data included computed tomography and three-dimensional facial scans. A soft tissue prediction algorithm was applied to simulate postoperative facial changes based on planned skeletal movements. The algorithm for predicting soft tissue displacement in relation to skeletal segment repositioning was developed through cephalometric analysis of preoperative and postoperative computed tomography scans, evaluating positional changes of selected soft tissue landmarks. Predicted outcomes were compared with actual postoperative results obtained with computed tomography and facial scanning at least six months after surgery. Quantitative analysis was performed by measuring linear and volumetric differences between predicted and actual soft tissue landmark positions.
**Results**: The algorithm demonstrated variable accuracy in predicting soft tissue changes in the midface and lower facial regions, with mean deviations below 2 mm for most anatomical landmarks. Greater discrepancies were observed in areas with complex soft tissue behavior, such as the lips and nasal tip. Overall, a strong correlation was found between predicted and postoperative outcomes (*p* < 0.05). The use of the algorithm improved treatment planning and patient communication by providing realistic visual simulations.
**Conclusion**: Soft tissue movement algorithms represent a valuable tool in orthognathic surgical planning, allowing for reliable prediction of aesthetic outcomes. Despite some limitations in highly dynamic regions, their integration into digital workflows enhances diagnostic precision and supports informed decision-making. Further refinement of these models may improve predictive capability and clinical applicability.

**FA26/293—Orthognathic Surgery**
**The Utilisation of 3D-Printed Titanium Cheek Implants for Aesthetic Improvement of the Midface Without Maxillary Le Fort I Advancement**
Firdaus Hanapiah ^1^ and Kathiravan Purmal ^1^
*^1^* *Avisena Specialist Hospital, Shah Alam, Malaysia*
**Objectives**: To evaluate the effectiveness of 3D-printed titanium patient-specific cheek implants in improving midfacial aesthetics without the need for maxillary Le Fort I advancement, and to assess their role as a minimally invasive alternative to conventional orthognathic surgery.
**Materials and Methods**: Patients presenting with mild to moderate midfacial deficiency seeking aesthetic improvement were assessed using cone beam computed tomography (CBCT) and 3D facial scanning. Virtual surgical planning was performed to design customized cheek implants tailored to individual anatomical requirements. The implants were fabricated using selective laser melting (SLM) technology in medical-grade titanium. Surgical placement was carried out via intraoral or transconjunctival approaches. Postoperative evaluation included clinical assessment of facial symmetry, malar projection, and overall aesthetic outcome.
**Results**: Patients demonstrated significant improvement in midfacial projection, malar prominence, and facial harmony. The procedure was associated with reduced operative time, minimal morbidity, and faster recovery compared to traditional orthognathic surgery. No major complications were observed, and implant positioning was accurate with good soft tissue adaptation.
**Conclusion**: 3D-printed titanium cheek implants provide a safe, predictable, and minimally invasive solution for midfacial aesthetic enhancement in selected patients. While not a substitute for orthognathic surgery in cases requiring occlusal correction, this approach represents a valuable addition to the armamentarium of modern maxillofacial surgery in the era of personalized digital medicine.

**FA26/296—Orthognathic Surgery**
**Use of PSI to Enhance Soft Tissue Appearance in Orthognathic Patients**
Guglielmo Ramieri ^1^ and Emanuele Zavattero ^1^
*^1^* *University of Turin, School of Maxillofacial Surgery, Turin, Italy*
**Objectives**: To discuss the use of Patient Specific Implants (PSI), alone or in combination with conventional osteotomies, to improve the soft tissues support and facial aesthetics.
**Materials and Methods**: Clinical cases are described and critically discussed.
**Results**: Soft tissues support and definition may be positively modified though use of PSI. Most common applications are in the malar area, gonial angle, lower mandibular border, as well as in symmetrisation of the face.
A low rate of complications and high patient’s satisfaction are observed.
**Conclusion:** The use of PSI alone or in combination with standard osteotomies should be considered for improved aesthetics in orthognathic cases.

**FA26/384—Orthognathic Surgery**
**Long-Term Skeletal Stability Following Clockwise Rotation in Orthognathic Surgery**
Mary Raj ^1^ and Mike Leung ^1^
*^1^* *Oral and Maxillofacial Surgery, Faculty of Dentistry, The University of Hong Kong, Hong Kong*
**Objectives:** To evaluate the long-term effects of varying degrees of clockwise rotation (CWR) of the maxillomandibular complex on skeletal stability following orthognathic surgery.
**Materials and Methods:** A retrospective cohort study was conducted at the Division of Oral and Maxillofacial Surgery, The University of Hong Kong. Consecutive patients who underwent bimaxillary orthognathic surgery with planned CWR between 2020 and 2024, with a minimum follow-up of 12 months, were included. Patients were stratified into mild (<2°), moderate (>2–5°), and severe (>5°) CWR groups based on changes in the occlusal plane–Frankfort horizontal (OP–FH) angle. Lateral cephalograms were analysed preoperatively, immediately postoperatively, and at 6 and 12 months. Primary outcome measured was the degree of horizontal and vertical positional changes of A point, B point, Pogonion, and Menton. Data were analysed using one-way ANOVA with significance set at *p* < 0.05.
**Results:** Twenty patients were included (mild n = 6, moderate n = 9, severe n = 5). At 6 months, greater horizontal instability was observed in the severe group, particularly at B point, Pogonion and Menton (*p* < 0.001), while vertical changes were also more pronounced at B point (*p* = 0.034) and Pogonion (*p* = 0.016). However, by 12 months, no statistically significant differences in horizontal or vertical skeletal stability were observed between groups. No significant associations were identified between demographic or surgical variables and stability outcomes.
**Conclusion:** Clockwise rotation of the maxillomandibular complex is associated with transient early skeletal instability, particularly in severe cases; however, long-term stability at 12 months is comparable across all degrees of rotation. These findings support the use of CWR as a predictable and stable surgical technique for orthognathic surgery.

**FA26/400—Orthognathic Surgery**
**Evaluation of Long-Term Skeletal and Soft Tissue Changes After Bimaxillary Surgery for Class III Skeletal Malocclusion Patients**
Shobina Sivanganam ^1^, Rozita Hassan ^2^ and AB Rani Samsudin ^3^
*^1^* *Hospital Sultan Idris Shah Serdang, Kajang, Malaysia*
*^2^* *Universiti Sains Malaysia, Kubang Kerian, Malaysia*
*^3^* *University of Sharjah, Sharjah, United Arab Emirates*
**Objectives**: This study aims to evaluate the long-term skeletal and soft tissue changes following bimaxillary surgery in Class III skeletal malocclusion patients.
**Materials and Methods**: The sample consisted of 15 patients (8 men, 7 women; mean age, 24.4 years) who had undergone simultaneous maxillary advancement and mandibular setback surgery to correct Class III skeletal malocclusion. Digital lateral cephalograms from the following time points were assessed using WinCeph software: pre-operative (T_0_), immediately post-operative (T_1_), and at least 2 years post-operative (T_2_). Comparisons were made between T_1_–T_0_ and T_2_–T_1_. Twenty-six linear and five angular parameters were used to evaluate postoperative changes. After linear and angular changes of skeletal and soft tissue variables were measured, the one-way repeated measures analysis of variance (ANOVA) with Bonferroni post-hoc test and Pearson correlation test were performed for statistical analysis, with significance set at *p* < 0.05.
**Results**: During long-term follow-up, statistically significant vertical changes were observed at ANS and Me, with a decrease in SNA angle and an increase in mandibular plane inclination. Statistically significant changes were observed at horizontal positions of Li, Si and Me’ when comparing long-term follow-up to pre-operative baseline. Skeletal and soft tissue movements following bimaxillary surgery were strongly correlated in the horizontal and vertical directions, for both maxilla and mandible.
**Conclusion**: Long-term follow-up showed significant vertical relapse and continued soft tissue adaptation. These findings provide a foundation for understanding potential long-term skeletal and soft tissue changes and predicting outcomes in Class III skeletal malocclusion patients after bimaxillary surgery.

**FA26/408—Orthognathic Surgery**
**Surgical Management of Facial Asymmetry: Combined High Condylectomy and Orthognathic Surgery for Active Condylar Hyperplasia and Skeletal Class III Deformity**
Peerach Kiatmanakul ^1^
*^1^* *Mahidol University, Bangkok, Thailand*
**Objectives**: To report a clinical case of facial asymmetry caused by active condylar hyperplasia (CH) and to demonstrate the efficacy of a single-stage surgical approach combining high condylectomy with orthognathic surgery in arresting pathological growth while achieving definitive skeletal correction.
**Materials and Methods**: A 26-year-old male patient presented with progressive facial asymmetry and a Skeletal Class III deformity that worsened between ages 22 and 25. Clinical and radiographic evaluations confirmed active left-sided CH as the primary etiology. The patient was managed through a comprehensive single-stage surgical intervention. The procedure involved a left high condylectomy to remove the active growth center, followed immediately by Le Fort I osteotomy and Bilateral Sagittal Split Ramus Osteotomy (BSSRO) for skeletal repositioning. Secondary aesthetic refinement was performed during hardware removal via mandibular angle reduction.
**Results:** The single-stage approach achieved a stable, functional occlusion and improvement in facial symmetry. Post-operative follow-up showed no signs of recurrent growth. The combined procedure immediately arrested the disease progression and restored aesthetic harmony. Concurrent mandibular angle reduction further refined the facial contour, leading to high patient satisfaction and stable long-term results.
**Conclusion:** The combination of high condylectomy and orthognathic surgery is a viable strategy for managing active condylar hyperplasia. By addressing the growth site and the skeletal deformity simultaneously during the active phase, clinicians can immediately arrest the disease progression and achieve definitive correction. This proactive approach avoids the psychological distress and the worsening of skeletal compensation that often occur when delaying surgery until the inactive phase, thereby providing more predictable and efficient long-term outcomes.

**FA26/425—Orthognathic Surgery**
**“The Intelligent Face: AI-Driven Transformation in Orthognathic Surgery”**
Hannah Arnantha ^1^ and Hitesh Bansal ^2^
*^1^* *St George’s, Epsom and St Helier Hospital Group, London, UK*
*^2^* *London North West University Healthcare NHS Trust, London, UK*
**Objectives**: To evaluate the current and emerging role of artificial intelligence (AI) in orthognathic surgery, with particular focus on its applications in diagnosis, treatment planning, surgical prediction, and perioperative risk assessment.
**Materials and Methods**: A narrative literature review was conducted using contemporary evidence on technological advancements in orthognathic surgery. Emphasis was placed on studies investigating machine learning (ML) and AI-driven systems in cephalometric analysis, virtual surgical planning (VSP), soft tissue prediction, and clinical decision-making. Additional applications in outcome prediction, aesthetic evaluation, and perioperative risk modelling were analysed to assess the breadth of AI integration within orthognathic workflows
**Results**: AI demonstrates significant potential across multiple stages of orthognathic care. Machine learning models have achieved high diagnostic accuracy, with reported rates of up to 96% in determining surgical versus non-surgical treatment and over 90% concordance with expert clinicians in treatment planning. AI-driven soft tissue prediction models show comparable or superior accuracy to conventional computational approaches, often achieving errors below 2 mm and improved reliability in the lower facial third. Beyond planning, AI has been successfully applied to predict perioperative outcomes, including blood loss with a mean error of 7.4 mL and postoperative infection risk with accuracy approaching 98.7%. Furthermore, AI-based aesthetic analysis suggests measurable improvements in facial attractiveness and perceived age following surgery. Despite these advances, limitations remain, including dataset size, variability in training populations, and challenges in clinical integration.
**Conclusion**: Artificial intelligence is poised to transform orthognathic surgery by enhancing diagnostic precision, optimising treatment planning, and enabling personalised, data-driven care. While current applications primarily support clinical decision-making rather than replace it, ongoing advancements in machine learning and data modelling are likely to further integrate AI into routine practice. Future work should focus on validation across diverse populations, ethical considerations, and seamless incorporation into clinical workflows.

**2FA26/1169—Orthognathic Surgery**
**Validation of Three-Dimensional Facial Imaging Technologies for Soft Tissue Assessment in Orthognathic Surgery: A Systematic Review**
Mohammad Azrin Abd Samad ^1^ and Azmeel Mazlee Anuar ^1^
*^1^* *Centre of Oral and Maxillofacial Studies, Faculty of Dentistry, Universiti Teknologi MARA, Sungai Buloh, Malaysia*
**Objectives**: Three-dimensional (3D) facial imaging technologies have become increasingly important in orthognathic surgery for evaluating facial soft tissue morphology and postoperative outcomes. Accurate soft tissue assessment is essential for treatment planning, outcome prediction, and postoperative evaluation. Conventional imaging modalities such as computed tomography (CT) and cone-beam computed tomography (CBCT) provide valuable information but involve ionizing radiation and are less suitable for repeated soft tissue assessment. Three-dimensional facial imaging offers a non-invasive and radiation-free alternative; however, the accuracy and reliability of different imaging technologies require evaluation before widespread clinical adoption. To systematically evaluate the accuracy and reliability of three-dimensional facial imaging technologies used for soft tissue assessment in orthognathic surgery.
**Materials and Methods**: A systematic search of MEDLINE (PubMed), Scopus, Web of Science, Embase, and Google Scholar was conducted up to 29 August 2025. Studies assessing the accuracy, reliability, or reproducibility of 3D facial imaging technologies in orthognathic surgery or validation settings were included. Study selection followed PRISMA guidelines. Data on imaging technologies, reference standards, surface deviation, and intraclass correlation coefficients (ICC) were extracted. Methodological quality was assessed using Joanna Briggs Institute (JBI) critical appraisal tools. Due to study heterogeneity, findings were synthesized narratively.
**Results**: Forty-seven studies comprising 54 datasets and approximately 3800 participants were included. Most 3D facial imaging systems demonstrated clinically acceptable accuracy, with mean surface deviations generally below 1.5 mm and high reproducibility (ICC > 0.90). Stereophotogrammetry showed the highest accuracy and reliability, with deviations ranging from 0.5–1.2 mm. Structured light scanners demonstrated comparable performance, while smartphone-based systems showed greater measurement errors.
**Conclusion**: Three-dimensional facial imaging technologies provide accurate and reliable soft tissue assessment for orthognathic surgery. Stereophotogrammetry remains the reference standard, whereas structured light scanners offer a practical and cost-effective alternative. Integration of 3D facial imaging can enhance surgical planning, outcome evaluation, and patient communication.

**2FA26/1204—Orthognathic Surgery**
**Guided Intraoperative Placement of a Bone-Borne Palatal Expander During Surgically Assisted Rapid Maxillary Expansion: A Two-Case Feasibility Report**
Libana Bjoernstrup ^1^, Louise Barnechow ^1^
*^1^* *Department of Oral and Maxillofacial Surgery, Copenhagen University Hospital, Rigshospitalet, Copenhagen, Denmark*
**Objectives**: Bone-borne palatal expansion devices are increasingly used in conjunction with surgically assisted rapid maxillary expansion (SARME). Conventional treatment pathways often require a separate preoperative appointment for appliance placement, adding time and inconvenience for patients. This report describes a digital workflow combining preoperative CBCT-based assessment of palatal bone thickness with guided intraoperative placement of a bone-borne palatal expander during SARME.
**Materials and Methods**: Two adult patients with transverse maxillary deficiency scheduled for SARME underwent preoperative CBCT imaging and digital planning. Palatal bone thickness was analyzed to identify optimal insertion sites for skeletal anchorage. A patient-specific surgical guide was designed using combined CBCT and digital dental models to facilitate accurate placement of the expansion device during surgery. The expander was inserted intraoperatively immediately following osteotomies. Feasibility of the workflow, appliance stability, and early postoperative outcomes were assessed.
**Results**: Guided placement of the bone-borne expander was successfully completed in both patients without intraoperative complications. Preoperative digital planning enabled placement of the anchorage screws in areas of favorable palatal bone thickness and anatomy. The surgical guide provided predictable positioning and satisfactory primary stability of the appliance. Both patients commenced postoperative expansion as planned, with no early device-related complications, screw loosening, or need for repositioning. The workflow eliminated the need for a separate preoperative appointment for appliance placement.
**Conclusion**: This two-case feasibility report demonstrates that guided intraoperative placement of a bone-borne palatal expander during SARME is achievable and may streamline the treatment pathway by reducing the number of patient visits while enabling patient-specific optimization of skeletal anchorage. Digital planning based on CBCT-derived palatal bone analysis may improve placement accuracy and appliance stability. Further studies are required to evaluate accuracy, clinical outcomes, and patient-reported benefits in larger cohorts.

**2FA26/1241—Orthognathic Surgery**
**Integrating Minds and Mandibles: A Multidisciplinary Approach**
Almas Prawoto  and Alki Andana
*Airlangga University, Surabaya, Indonesia*
**Introduction** Orthognathic surgery is essential for correcting malocclusions, such as mandibular prognathism, and addressing their functional and aesthetic impacts. Historically, traditional single-discipline surgical planning has presented significant limitations. Consequently, there is a growing need for a multidisciplinary approach that integrates plastic surgery, orthodontics, and biotechnical fields to optimize occlusion, facial aesthetics, and patient satisfaction. The primary objective of this study is to evaluate the effectiveness of this integrated planning approach and assess its impact on surgical accuracy, functional outcomes, aesthetic results, and patient satisfaction.
**Methods** The methodology relies on a collaborative team and a structured clinical algorithm, beginning with a referral from a primary care doctor. Key multidisciplinary roles include the orthodontist managing pre- and post-surgical tooth positioning, the plastic surgeon handling surgical design and execution, alongside medical engineering and psychiatric support. This process utilizes advanced digital tools to enable collaborative workflows, including 3D imaging (such as CBCT, CT scans, cephalometry, panoramic X-rays, and intraoral scanning), CAD/CAM, virtual surgical simulation, and 3D printing. Post-operative progress is systematically tracked using cephalometry immediately after surgery, and subsequently at one, three, six, and twelve-month intervals.
**Results** To assess the practical application of this multidisciplinary framework, case studies were conducted involving five specific patients: Nathanael, Supriyanto, Saddam, Ismail, and Faqih. Through these clinical cases, the study evaluated how the coordinated planning approach directly impacted surgical accuracy, functional outcomes, aesthetic results, and overall patient satisfaction.
**Conclusion** The summary of key findings highlights the significant benefits and practicality of utilizing multidisciplinary planning in orthognathic surgery. By combining diverse medical expertise with advanced technological innovations, this comprehensive approach improves patient care. Consequently, there is a strong call for the broader adoption of these collaborative methods and further research to continue advancing the field.

**FA26/37—Reconstruction**
**Extended Superolateral Periosteal Flap for Single-Stage Total Lower Eyelid Reconstruction**
Pattana Ongkasuwan ^1^
*^1^* *Nakornping Hospital, Chiang Mai, Thailand*
**Objectives:** Total lower eyelid defects following oncologic resection present significant reconstructive challenges, requiring restoration of both anterior and posterior lamellae with stable medial canthal fixation. Traditional approaches often necessitate staged reconstruction or composite grafts. This report describes an extended superolateral periosteal flap technique that enables single-stage reconstruction of total lower eyelid defects by maximizing flap length along the superior orbital rim.
**Materials and Methods:** A 71-year-old woman with basosquamous carcinoma involving the entire lower eyelid underwent full-thickness excision with 5-mm oncologic margins, resulting in a 3.0 × 3.5 cm defect. Posterior lamellar reconstruction was performed using a periosteal flap harvested from the lateral orbital rim and extended medially along the superior orbital rim to within 2–3 mm of the supraorbital notch, achieving a flap length of 3.5 cm. The lateral canthal attachment was preserved as the vascular pedicle. The flap was transposed inferiorly and secured to the medial canthal tendon using polypropylene sutures. Inferior fornix conjunctiva was advanced to cover the periosteal surface. The anterior lamella was reconstructed using a modified cheek rotation cutaneous flap.
**Results:** At 3-month follow-up, the reconstructed lower eyelid demonstrated stable margin position with preserved alignment and complete flap survival. No ectropion, entropion, lagophthalmos, symblepharon, or flap necrosis was observed. Postoperative chemosis and upper eyelid edema resolved completely within 3 months. Epiphora secondary to lower lacrimal system sacrifice persisted. The donor site achieved primary closure without complications. Longer-term follow-up was precluded by patient death from pneumonia unrelated to the surgical procedure.
**Conclusion:** The extended superolateral periosteal flap provides sufficient length (3.5 cm) for tension-free medial canthal fixation, allowing reconstruction of total defects beyond the traditionally reported limits of periosteal flaps. This anatomy-based modification expands the applicability of periosteal flaps for complete lower eyelid defects and may reduce the need for staged reconstruction in selected cases.

**FA26/58—Reconstruction**
**Delayed Nasal Tip Reconstruction Using a Triple Layer Flap-Ear Cartilage-FlapTechnique in a Pediatric Nasal Dog Bite Amputation**
Mark Jhervy Villanueva ^1^ and Arlex Michael Atanacio ^1^
*^1^* *Bataan General Hospital and Medical Center, City of Balanga, Bataan, Philippines*
**Objectives:** To present a case of pediatric nasal dog bite amputation and describe its reconstruction using a triple layer flap-ear cartilage-flap technique.
**Methods:**
  **Design:** Case Report
  **Setting:** Tertiary Government Training Hospital
  **Patient:** One
**Results:** A 6-year-old male sustained a nasal tip amputation after a dog attack. Initial management at a private hospital included anti-tetanus and anti-rabies prophylaxis, antibiotics, and wound care. One week post-injury, he presented with traumatic loss of the nasal tip and a full-thickness defect of the medial right nasal ala, including the lower lateral cartilage, with no additional intranasal defects on endoscopy. Planned reconstruction was delayed due to a COVID-19 infection.
Four weeks post-injury, a triple-layer nasal reconstruction was performed. A nasoseptal flap was used to restore the intranasal mucosal lining, an autologous cartilage graft replaced the missing lower lateral cartilage, and a right paramedian forehead flap reconstructed the nasal skin and subcutaneous tissue. The patient was discharged on postoperative day three without complications. Pedicle division, debulking, and final flap inset were completed three weeks later. No donor- or recipient-site complications occurred, and the aesthetic and functional outcomes were satisfactory.
Dog bites account for 69% of animal bite injuries in the Philippines (Department of Health & Department of Agriculture, 2020). Nasal reconstruction remains challenging due to complex anatomy and contour (Scott-Brown & Gleeson, 2008). Successful outcomes rely on replacing lost tissue with similar tissue components, including internal lining, cartilage support, and skin coverage (Baker, 2011; Baker, 2014; Flint, 2021). The paramedian forehead flap remains a reliable option for extensive nasal defects (Ryu & Jang, 2023).
**Conclusion:** A surgeon treating a pediatric nasal dog bite amputation needs to consider a delayed nasal tip reconstruction using a triple layer flap-ear cartilage-flap technique as a key surgical method because of its aesthetic, and functional implications.

**FA26/65—Reconstruction**
**The Use of a Dermal Regeneration Template and Customized Splint for Oral Cavity Defect Reconstruction**
Rita Martins ^1^, Lídia Gomes ^1^, Andreia Silva ^1^, Sara Lopes ^1^
*^1^* *Hospital de Braga, Braga, Portugal*
**Objectives**: This report presents a clinical case of a 35-year-old female who was referred to our department with a slowly growing palatal swelling. In this work, we aim to highlight a simple yet effective method for reconstructing oral cavity defects.
**Materials and Methods**: A thorough clinical history was obtained; the patient had no relevant medical history apart from a painless palatal tumefaction that had progressively grown over several years.
Intraoral examination revealed a fibrous-elastic mass on the left hemipalate. Magnetic resonance imaging and needle biopsy suggested a benign tumor of the minor salivary glands. The lesion was excised under general anesthesia. Due to the size of the resulting mucosal and bone defect, a dermal regeneration template was used for reconstruction. A removable, custom-made acrylic splint ensured optimal adaptation between the membrane and the defect.
**Results**: After two weeks, the template was removed, revealing newly formed mucosa with maintained oral function. Histopathological analysis confirmed a cystadenoma, a rare benign epithelial tumor of the salivary glands. The patient continues to attend periodic follow-up appointments, without evidence of recurrence.
**Conclusion:** This case underscores the effectiveness of dermal regeneration templates in reconstructing small to medium sized oral cavity defects, whether mucosal or bony. A customized acrylic palatal splint ensures precise adaptation of the template, promotes wound healing in a challenging anatomical site, and enhances patient comfort and hygiene. This method facilitates faster mucosal regeneration with reduced complications, minimal patient discomfort, and no need for a donor site.

**FA26/94—Reconstruction**
**Balancing Aesthetic Subunits and Oncologic Safety in Facial Reconstruction: A Case-Based Surgical Approach**
Fairouz Bekrentchir ^1^
*^1^* *University hospital of Oran, ORAN, Algeria*
**Objectives**: Basal cell carcinoma of the face frequently involves highly visible aesthetic units. Reconstruction after oncologic excision must restore function and facial harmony while ensuring complete tumor removal. This study aimed to evaluate the application of the facial aesthetic subunit principle in three complex midface basal cell carcinoma cases involving single and multiple facial subunits.
**Materials and Methods**: A retrospective analysis was performed on one patient treated in 2024 and two patients treated in 2026 for histologically confirmed basal cell carcinoma involving the upper eyelid, the nasogenian-cheek-upper lip region, and the nasal dorsum with cheek extension. All patients underwent complete tumor excision with histologically free margins. Reconstruction was planned according to facial aesthetic unit principles, considering skin color, thickness, defect depth, functional landmarks, and scar camouflage. When more than 50% of a subunit was involved, reconstruction aimed to restore the entire cosmetic unit. The upper eyelid defect was reconstructed using a frontoglabellar flap. The nasogenian-cheek-upper lip defect required a Mustardé flap, paramedian forehead flap, hemi-Karapandzic flap, and contralateral Estlander flap. The nasal dorsum-cheek defect was reconstructed using a Mustardé flap and a paramedian forehead flap.
**Results**: All three patients achieved complete excision with negative surgical margins. Functional and aesthetic outcomes were satisfactory in all cases. Eyelid mobility and ocular protection were preserved in the upper eyelid case. Oral competence and facial symmetry were maintained in the multi-unit nasogenian-cheek-upper lip case. Nasal contour and cheek projection were successfully restored in the nasal dorsum case. No flap necrosis or early local recurrence were observed.
**Conclusion**: Reconstruction of facial defects should follow aesthetic subunit principles to optimize both oncologic safety and cosmetic outcomes. Small lesions may be treated with full-thickness skin grafts, whereas larger multi-subunit defects require locoregional flaps, with careful scar placement along subunit junctions to ensure optimal functional and aesthetic results.

**FA26/102—Reconstruction**
**Revisiting Musculoclavicular Bone Flap Reconstruction for Post-Hemimandibulectomy Ameloblastoma Patients in a Tertiary Government Hospital: A Case Series**
Erica Aragon ^1^, Rachelle Marie Nonato-Deraja ^1^ and Emilio Raymund Claudio ^1^
*^1^* *Corazon Locsin Montelibano Memorial Regional Hospital, Bacolod, Philippines*
**Objectives**: To discuss outcomes associated with musculoclavicular bone flap for mandibular defect reconstruction after hemimandibulectomy among mandibular ameloblastoma patients and to highlight its role as an alternative reconstructive option in selected clinical settings.
**Materials and Methods**:
Study design: Case Series
Setting: Tertiary Public Hospital
Subjects: Six cases of mandibular ameloblastoma requiring hemimandibulectomy and immediate reconstruction. All patients underwent standardized preoperative evaluation and were followed post-operatively for functional and cosmetic outcomes.
**Results**: Six patients with biopsy-proven mandibular ameloblastoma underwent single-stage hemimandibulectomy and musculoclavicular bone flap reconstruction. The ipsilateral clavicle was harvested with preservation of the sternocleidomastoid muscle as its vascular pedicle and transferred to the mandibular defect, where it was rigidly fixed using mandibular titanium 2–0 plates. Post-operatively, four of the six cases exhibited good coaptation, stable fixation, and successful integration without major complications. Two out of six cases exhibited protrusion of the clavicle bone edge at the recipient site, which were subsequently managed with debridement and coverage using rotational skin flaps, resulting in acceptable wound healing and stability. Mandibular contour was generally maintained with the use of the clavicular flap providing adequate structural support. Functional outcomes, including oral competence and ability to tolerate oral intake, were preserved in most cases. Overall, favorable cosmetic results were attained, with acceptable facial symmetry and patient satisfaction noted during follow-up.
**Conclusion**: The musculoclavicular flap, though considered a dated technique and despite the advent of microvascular free flaps, remains a viable and dependable option for reconstructing mandibular defects after hemimandibulectomy, particularly in settings where free flap reconstruction is not feasible due to limited resources or expertise. In this series, musculoclavicular bone flap reconstruction achieved satisfactory mandibular contour and acceptable cosmetic outcomes. This technique continues to serve as a reliable, practical, and cost-effective alternative for mandibular reconstruction in resource- or expertise-limited settings.

**FA26/133—Reconstruction**
**SCIP Flap in Head an Neck Reconstruction**
Vedran Zubčić ^1^
*^1^* *University Hospital Osijek, Osijek, Croatia*
**Objectives**: SCIP flap was first introduced in clinical practice by Isao Koshima 2004. as an evolution of the original groin flap described by McGregor & Jackson 1972. Vascular supply is based is on an superficial circumflex iliac artery. So far it gained popularity in lower extremity reconstruction due to reduced bulkness, minimal donor side morbidity and possibilities for a versatile tissue reconstruction which may include soft tissue and bone. Although the pedicle lenght can be short, with a meticulous dissection pedicle can lenghten up to 6 cm.
**Materials and Methods**: A retrospective review of 13 head and neck patients treated between 2023. and 2025. was conducted. Most of the patients were advanced cancer/haemangioma/melanoma patients in areas including cheek, orbit, oral defects. Two cases were reconstruction of the soft tissue defeects due to a Parry-Romberg syndrome. Preoperative color Doppler and CT angio planning for perforator mapping were obtained in every case and in some cases including creation of the 3D printed models and cutting guides for the facial skeleton resection and reconstruction as well.
**Results**: A wide range of head and neck defects were reconstructed with a SCIP flap. Cosmetic and functional outcomes were satisfactory with less donor site morbidity and complications.
**Conclusion**: SCIP flap in head and neck reconstruction can provide a large thin skin and moderately sized vascularized bone with minimal donor site morbidity. With the improvement of the medical knowlegde and advancement of medical technologies, SCIP flap can become standard procedure in head and neck reconstruction.

**FA26/144—Reconstruction**
**A Painful Blink After Orbital Floor Repair: Cicatricial Entropion Reconstructed with Labial Mucosal Graft**
Gerald Joseph ^1^ and Kar Tsyeng Ng ^1^
*^1^* *Hospital Teluk Intan, Teluk Intan, Malaysia*
**Objectives**: Transconjunctival approaches for orbital floor fracture repair are widely used due to superior cosmetic outcomes and lower ectropion rates compared with subciliary incisions. However, cicatricial entropion remains a recognized but infrequent complication.
**Materials and Methods**: A patient who developed symptomatic lower eyelid entropion two weeks after transconjunctival orbital floor reconstruction. The patient presented with sharp ocular pain exacerbated on downward and medial gaze. Conservative management with massage and eyelid taping failed to improve symptoms. Clinical examination confirmed cicatricial entropion secondary to posterior lamellar shortening. Surgical correction was performed with scar release and posterior lamellar lengthening using autologous labial mucosal graft.
**Results**: Postoperatively, gaze-related pain resolved completely. Mild postoperative trichiasis caused transient foreign body sensation and epiphora, which was managed conservatively.
**Conclusion**: Labial mucosal grafting provides an effective, accessible, and well-tolerated option for correction of cicatricial entropion following transconjunctival orbital surgery.

**FA26/163—Reconstruction**
**Versatility of Free Fibula flaps in Maxillary and Mandibular Reconstruction Following Head and Neck Resection: Experience of a Single Surgeon**
Rahul Kapoor ^1^
*^1^* *Blk-Max Superspeciality Hospital, Karol Bagh, New Delhi, India*
**Objectives**: To showcase versatility of free fibula flaps in Head and Neck Reconstruction post tumor resection.
**Materials and Methods**: All planning and Reconstruction done by Single Surgeon at 3 different centres over period of 9 years. Our study showcase different variations and versatility in free fibula flaps using various components—bone, skin paddle, muscle (flexor hallucis longus and soleus) and periosteal flap, in maxillary and mandibular reconstruction respectively. Single free fibula flap can be utilised for simultaneous reconstruction of maxilla and mandible, in composite resection, provided the length of bone defect is equal to or less than the length of fibula flap harvested. This could provide possibility of dental rehabilitation in future. Fibula flap has 4 components, which if properly dissected out could be used in various combinations to fulfil appropriate tissue requirement for each defect. Bone could be used in single or double barrel combination, either for maxillary or mandibular reconstruction, depending on extent of defect. Double barrel gives advantage of adequate bone stock needed for dental rehabilitation, post treatment completion, in addition to tissue support and cosmesis. Proper planning and dissection upto perforator level could ensure 360 rotation of skin paddle without any compression and satisfactory inset. Muscle could be used for lining, cover or to fill up dead space or to give lip support and prevent oral incompentence. Periosteal flaps cover fibula-bone junction, enchance bony union and minimize plate exposure.
**Results**: Free fibula flap is most versatile flap for head and neck reconstruction. It has steep learning curve. Proper pre- operative investigations and intra-operative planning could, maximise its benefits in head and neck reconstruction.
**Conclusion**: Free fibula flap is most versatile flap for head and neck reconstruction. If planned properly, it could fulfill both bony and soft tissue requirements, in one sitting.

**FA26/195—Reconstruction**
**A New Era of Jaw Reconstruction: Restoring Form and Function with Subperiosteal Implants**
Mausumi Iqbal ^1^
*^1^* *Apollo Clinic, JMI Specialized Hospital, Tooth Care, Facio-Maxillary Reseach & Development Hub, Dhaka, Bangladesh*
**Objectives**: Extensive maxillofacial bone loss from trauma, oncologic resection, infection, or long-term edentulism presents significant reconstructive challenges. Conventional options—autogenous bone grafting, distraction osteogenesis, and endosseous implants—can be limited by donor-site morbidity, prolonged treatment, insufficient bone volume, or compromised soft-tissue envelope. Contemporary subperiosteal implant techniques, leveraging modern imaging, CAD/CAM design, and biocompatible materials, offer an alternative for immediate, patient-specific restoration of form and function.
**Materials and Methods**: We conducted two cases severe maxillary or mandibular defects treated with custom, 3D-printed subperiosteal implants between 2024–2025. Preoperative planning used high-resolution CT, virtual surgical planning, and CAD/CAM implant design to contour implants to the native cortical surface and avoid vital structures. Titanium alloy frameworks were manufactured by additive manufacturing, surface-treated to promote soft-tissue integration, and fixated with locking screws. Primary outcomes included implant fit accuracy, surgical time, time to prosthetic loading, peri-implant soft-tissue health, functional outcomes (masticatory ability, speech), aesthetic satisfaction, and complications (infection, exposure, screw loosening).
**Results**: All implants achieved accurate fit with no intraoperative conversions. Patients reported marked improvement in masticatory function and facial symmetry. Few complications like soft tissue dehiscence were manageable with local care or revision surgery or with prosthetic frame design in most cases.
**Conclusion**: Custom 3D-printed subperiosteal implants represent a viable reconstructive option for patients with extensive jaw bone loss who are poor candidates for conventional reconstruction.

**FA26/198—Reconstruction**
**Clinicoradiographic Evaluation of Injectable Platelet-Rich Fibrin and Hydroxyapatite in Maxillomandibular Bony Defects**
Vyakhya Gupta ^1^ and Naqoosh Haidry ^1^
*^1^* *All India Institute of Medical Sciences Patna, Patna, India*
**Objectives**: Enucleation of maxillomandibular cysts and tumors can result in postoperative bony defects that require prolonged healing. This study aimed to evaluate the clinical and radiographic healing potential of injectable platelet-rich fibrin combined with hydroxyapatite compared with hydroxyapatite alone in maxillomandibular bony defects following surgical enucleation.
**Materials and Methods**: A double-blinded randomized controlled trial was conducted in patients undergoing surgical enucleation of maxillomandibular cysts or tumors measuring 2–6 cm. After enucleation and primary closure, the defects were grafted with either hydroxyapatite alone or hydroxyapatite combined with injectable platelet-rich fibrin. Clinical parameters, including pain, edema, and mucosal color, were assessed on postoperative days 1, 3, and 7. Radiographic parameters, including percentage reduction in defect volume and density of newly formed bone, were assessed at 2 and 4 months postoperatively. Statistical analysis was performed to compare clinical and radiographic outcomes between the two groups.
**Results**: A total of 19 maxillomandibular cysts or tumors were included in the final analysis. Clinical outcomes were better in the combination group; however, the differences were not statistically significant. Radiographically, the combination group showed significantly greater healing ratios at 2 months and 4 months compared with hydroxyapatite alone. The healing ratio was 63.5 ± 2.34% versus 57.2 ± 5.21% at 2 months (*p* = 0.007) and 90.3 ± 7.32% versus 80.8 ± 5.33% at 4 months (*p* = 0.005). The density of newly formed bone was also significantly higher in the combination group at 2 months (623 ± 42.9 HU versus 528 ± 96.5 HU; *p* = 0.020).
**Conclusion**: Injectable platelet-rich fibrin combined with hydroxyapatite demonstrated faster and better radiographic bone healing than hydroxyapatite alone in post-enucleation maxillomandibular bony defects.

**FA26/214—Reconstruction**
**Alveolar Distraction Osteogenesis for vertical Ridge Augmentation Following Mandibular Ameloblastoma Resection: A Case Report**
Natchanon Mongkolrattanachat ^1^
*^1^* *Mahidol university, Bangkok, Thailand*
**Introduction**: Ameloblastoma is a benign yet locally aggressive odontogenic neoplasm, which is typically managed via surgical resection to minimize recurrence. Although radical excision is oncologically mandated, it often results in alveolar defects that compromise oral function. Consequently, reconstructive strategies are essential to restore both anatomical continuity and functional capacity for long-term oral rehabilitation.
**Treatment Overview**: A 32-year-old Thai female presented with a four-month history of persistent gingival swelling in the right mandibular premolar region. Clinical examination revealed a buccal expansion with a bony-hard consistency, extending from tooth 42 to tooth 43. Additionally, tooth 43 was distally displaced, resulting in spacing between tooth 42 and tooth 43. Radiographic examination revealed a well-defined, multilocular radiolucency size 1.5 × 1.5 × 1 cm with buccal cortical expansion and perforation. Histopathological evaluation confirmed a conventional ameloblastoma with a desmoplastic pattern. The lesion was managed via marginal mandibulectomy with concurrent nerve lateralization to preserve the inferior alveolar nerve. Immediate reconstruction was performed using an autogenous iliac crest bone graft. Following graft maturation, the patient exhibited an atrophic ridge in the regions of teeth 41–44. To address this vertical deficiency, alveolar distraction osteogenesis was utilized for vertical ridge augmentation. Successful distraction achieved sufficient bone height and volume, providing a stable foundation for subsequent dental implant rehabilitation.
**Conclusion**: Alveolar distraction osteogenesis remains a valuable alternative for secondary ridge augmentation after tumor resection and primary grafting. It offers the unique advantage of simultaneous hard and soft tissue expansion, which is crucial for achieving successful prosthetic outcomes in reconstructed patients.

**FA26/250—Reconstruction**
**Microsurgical Reconstruction of a Complex Fronto-Temporal Defect with Bone Exposure and Upper Eyelid Involvement Using a Radial Forearm Free Flap After Trauma**
L. Enrique G-Milanes ^1^, José Gerardo Solano Vazquez ^1^ and Armando Ocampo ^1^
*^1^* *Instituto Mexicano del Seguro Social, Mexico City, Mexico*
**Objectives**: To present a case of microsurgical reconstruction using a radial forearm free flap (RFFF) for a complex fronto-temporal defect with bone exposure and upper eyelid loss following high-energy trauma, and to evaluate functional and aesthetic outcomes.
**Materials and Methods**: A 38-year-old male sustained a high-energy motor vehicle accident in December 2024 resulting in extensive soft tissue loss involving the right fronto-temporal and periorbital regions with bone exposure, complete loss of the right upper eyelid, and absence of the levator palpebrae superioris muscle. A multistage reconstructive approach was performed. Following emergency debridement, a RFFF was used for soft tissue coverage. Subsequently, right upper eyelid reconstruction was achieved using contralateral upper eyelid full-thickness skin grafting, lateral canthopexy, and permanent tarsorrhaphy. Intraoperatively, a corneal ulcer and medial orbicularis oculi muscle loss were identified.
**Results**: At 3-month follow-up, the flap demonstrated complete integration with 100% survival. A small residual 15 × 2 mm superficial defect persisted in the supraciliary region. The patient preserved ocular motility; however, residual 5 mm lagophthalmos secondary to cicatricial retraction persisted, managed with intralesional methylprednisolone. No donor site complications occurred.
**Conclusion**: This case demonstrates that RFFF is a reliable option for complex traumatic periocular reconstruction. While most published series involve oncologic resections with well-defined margins, traumatic defects pose greater challenges due to unpredictable tissue loss and contamination. The RFFF’s thin, pliable characteristics and reliable vascularity make it particularly advantageous for periocular reconstruction where functional preservation is critical, achieving adequate soft tissue coverage while maintaining facial contour and ocular function.

**FA26/288—Reconstruction**
**Overcoming Height Mismatch in Fibula Flap Reconstruction Following Segmental Mandibulectomy: A Vertically Adjustable Double-Barrel Technique (VA-DBFF)**
Ching-En Chen ^1,2^
*^1^* *Taipei Veterans General Hospital, Beitou District, Taiwan*
*^2^* *National Yang Ming Medical College, Taipei, Taiwan*
**Objectives**: The fibula free flap (FFF) remains the gold standard for segmental mandibular reconstruction. While the conventional double-barrel configuration improves alveolar height, it may lead to vertical overcorrection in patients with smaller native mandibular height, particularly in Asian and elderly populations. This mismatch can adversely affect facial contour and prosthetic planning. We present the Vertically Adjustable Double-Barrel Fibula Flap (VA-DBFF), a modified technique that enables precise vertical customization while maintaining vascular reliability.
**Materials and Methods**: The VA-DBFF technique involves longitudinal segmentation of the fibula with preservation of the periosteum, vascular pedicle, and perforators. The segments are repositioned in a vertically adjustable configuration to match the patient-specific mandibular height. Rigid fixation is achieved using mini-plates and screws, followed by standard microvascular anastomosis.
**Results**: Two male patients with left lower gingival squamous cell carcinoma underwent segmental mandibulectomy and reconstruction using the VA-DBFF technique. One patient presented with a through-and-through composite defect requiring dual skin paddles, whereas the other had an isolated intraoral mucosal defect. The osteotomy was completed within approximately 75 min. The VA-DBFF enabled simultaneous reconstruction of osseous and soft tissue components while allowing controlled vertical adjustment to restore native mandibular height. The vertically stacked fibular segments provided improved structural support and contour alignment compared with conventional double-barrel configurations. Radiographic evaluation demonstrated stable fixation, reliable bone union, and satisfactory restoration of mandibular contour, facilitating future dental rehabilitation.
**Conclusion**: The VA-DBFF represents a refined evolution of the conventional double-barrel fibula flap, allowing patient-specific vertical modulation without compromising vascular integrity. This technique improves bony contact, restores native mandibular height, and enhances both aesthetic and functional outcomes, offering a practical solution to the limitations of traditional double-barrel reconstruction.

**FA26/292—Reconstruction**
**Study on the Application of Digital Design-Assisted Single Split-Fibula for Double Jaws Reconstruction After Multiple Facial Fractures**
Jinyang Wu ^1^, Peiru Min ^1^, Yixin Zhang ^1^ and Shilei Zhang ^1^
*^1^* *Shanghai Ninth People’s Hospital, Shanghai Jiao Tong University School of Medicine, Shanghai, China*
**Objectives:** Extensive post-traumatic composite defects involving simultaneous maxillary and mandibular loss with through-and-through intraoral-extraoral soft-tissue deficiency represent a formidable reconstructive challenge, often necessitating multiple free flaps and complex intraoperative adjustments to restore skeletal continuity, occlusion, and oro-nasal separation. This study describes a digitally assisted single split-fibula technique for synchronous double jaws reconstruction using one fibular osteomyocutaneous flap and investigates its effectiveness.
**Materials and Methods**: The patient, female, 57 years old, presented with left maxillary and mandibular defects, malocclusion, temporomandibular joint dislocation, and persistent oro-nasal communication following multiple craniofacial fractures. Preoperative three-dimensional imaging and virtual surgical planning were used to perform deformity analysis, mandibular repositioning, condylar reduction, and defect quantification. Fibular osteotomies were digitally designed, and three-dimensional printed guides and prebent reconstruction plates were prepared to facilitate accurate segment positioning. Lower-extremity computed tomography angiography and duplex ultrasonography confirmed two reliable perforators, enabling partition of a single skin paddle for simultaneous intraoral lining and extraoral resurfacing. Intraoperatively, scar release and intermaxillary fixation established a stable occlusal reference. The segmented fibula was inset to reconstruct the mandible and maxilla sequentially, followed by compartmentalized soft-tissue repair based on the dual perforators. Surgical outcomes, including the accuracy evaluation of jaws reconstruction, occlusion, and facial contour, were observed postoperatively.
**Results:** The reconstructive operation was completed successfully with the assistance of surgical guides and prebent plates. Postoperative imaging demonstrated high concordance between the virtual plan and surgical outcome. The flap survived completely, with restoration of mandibular continuity, maxillary support, stable occlusion, resolution of oro-nasal fistula, and improved facial contour. The patient was satisfied with her surgical outcomes.
**Conclusion:** This technique provides a reproducible workflow for digital design-assisted single-flap synchronous bimaxillary and intraoral-extraoral reconstruction, reducing donor-site morbidity and operative burden while achieving functional and aesthetic rehabilitation in selected complex post-traumatic cases.
Conflict of interest disclosure
Receipt of grants/research supports: This work was supported by the Natural Science Foundation of Shanghai, Shanghai Action Plan for Science, Technology and Innovation (24ZR1443700), Fundamental Research Funds for the Central Universities (YG2023LC05), SJTU Trans-med Awards Research (20230104), the National Clinical Research Center for Oral Diseases (NCRCO202307 and NCRCO202106), Shanghai Ninth People’s Hospital, Shanghai Jiao Tong University School of Medicine (Research Doctor, XKQJS202401, JS22A03), Shanghai’s Top Priority Research Center (2022ZZ01017), CAMS Innovation Fund for Medical Sciences(CIFMS) (2019-I2M-5-037), and Project 21DZ2271700-1 from Shanghai Key Laboratory of Medical Imaging Computing and Computer Assisted Intervention. This work was also supported in part by the National Nature Science Foundation of China (82372527).

**FA26/302—Reconstruction**
**Multistage Mandibular Reconstruction Following Segmental Mandibulectomy for Ameloblastoma: A Case Report**
Supakit Lumpaolertwilai ^1^
*^1^* *Department of Oral and Maxillofacial Surgery, Faculty of Dentistry, Mahidol University*
Mandibular reconstruction following segmental mandibulectomy for ameloblastoma presents significant challenges, particularly when initial reconstruction fails. Multistage reconstruction using different modalities may be required to achieve satisfactory functional and aesthetic outcomes.
A 56-year-old Thai male with a known case of ameloblastoma at the right mandible underwent segmental mandibulectomy with reconstruction using a left fibula free flap (FFF) and reconstruction plate at an external institution. Subsequently, wound dehiscence with necrotic bone exposure developed following a history of facial trauma, necessitating referral to a tertiary center.
At our institution, the patient underwent three additional staged operations. The first procedure involved removal of necrotic bone and the failed reconstruction plate, followed by reconstruction with a pre-bent reconstruction plate and right pectoralis major myocutaneous flap (PMMF). Following plate fracture, a second procedure was performed to replace the reconstruction plate and augment with a left iliac crest bone graft (ICBG), achieving osseous continuity. A third procedure addressed soft tissue refinement through reconstruction plate removal and scar revision at the lower lip. Currently, the patient demonstrates satisfactory healing with a bulky soft tissue flap at the reconstructed site, and vestibuloplasty with acellular collagen matrix (Mucoderm) is planned as a final step prior to prosthetic rehabilitation.
This case illustrates that multistage mandibular reconstruction, combining regional flaps, bone grafting, and soft tissue management, can achieve successful outcomes even after multiple reconstruction failures.

**FA26/316—Reconstruction**
**Staged Reconstruction of a Large Frontotemporal Defect with Orbital Involvement After Severe Craniofacial Trauma: Free Radial Forearm Flap and Secondary Eyelid Restoration**
Armando Ocampo ^1^, José Gerardo Solano Vazquez ^1^ and L. Enrique G-Milanes ^1^
*^1^* *Instituto Mexicano del Seguro Social, Ciudad de Mexico, Mexico*
**Objectives**: To assess reconstructive reliability and functional outcomes following staged management of a large frontotemporal defect with orbital involvement and secondary eyelid dysfunction after severe craniofacial trauma.
**Materials and Methods**: A 37-year-old male with no relevant prior comorbidities sustained a high-energy motor vehicle accident resulting in left orbital fractures, traumatic optic neuropathy with complete vision loss, and a large frontotemporal myocutaneous defect with cranial bone exposure. Initial multidisciplinary management included orbital floor reconstruction using an anatomical titanium mesh and microsurgical soft tissue reconstruction with a free radial forearm flap. Subsequent reconstructive stages addressed cicatricial lagophthalmos secondary to scar contracture through scar release and full-thickness retroauricular skin grafting. Clinical evaluation focused on soft tissue stability, eyelid function, and ocular surface protection.
**Results**: Free tissue transfer resulted in complete flap survival with stable and durable coverage of the exposed cranial structures. Secondary procedures achieved complete eyelid closure with resolution of cicatricial lagophthalmos and absence of corneal exposure. Functional protection of the ocular surface was restored. Visual deficit remained unchanged due to the primary optic nerve injury.
**Conclusion**: Staged reconstruction integrating anatomical orbital repair and microsurgical free tissue transfer offers reliable outcomes in large frontotemporal defects with cranial bone exposure following severe craniofacial trauma. Unlike reports focusing on isolated orbital reconstruction, soft tissue coverage, or eyelid repair, this case highlights the value of a structured reconstructive sequence addressing skeletal stabilization, vascularized coverage, and secondary functional eyelid restoration. Importantly, it demonstrates that effective ocular surface protection can be achieved even in the setting of irreversible vision loss, emphasizing functional rehabilitation beyond visual recovery.

**FA26/324—Reconstruction**
**The Need for Microvascular Surgeons to Assist in the Training and Care of War Injuries in Ukraine**
Warren Schubert ^1^, Steve Henry ^2^ and Patrick Kelley ^2^
*^1^* *University of Minnesota, St. Paul, MN, USA*
*^2^* *Dell Medical School, Austin, University of Texas, Austin, TX, USA*
A surgical mission to central Ukraine was organized in April, 2026 with the hope to help soldiers and civilians who require microsurgical reconstruction. This was coordinated with the Ukrainian Christian Medical Association. One microscope was donated by our team and another microscope had previously been donated. Micro instruments were purchased to be left behind. Dopplers were purchased and donated for the trip. There is a lack of centers in Ukraine that offer microsurgical services. All three of the surgeons on our team had a special interest in craniomaxillofacial injuries and reconstruction, but also had training in reconstructive repair of the extremities. Several Ukrainian surgeons from orthopedic surgery and oral and maxillofacial surgery scrubbed on 7 complex cases. Many of the cases may have resulted in amputation without free flaps or rotational flaps, and many patients already had amputations and needed flap coverage of exposed bone. As a result of the plethora of extreme cases in need, only one mandibular reconstruction could be performed, with other severe maxillofacial injuries left unattended. Arrangements are being made to bring one of the Ukranian surgeons to the United States for 3–6 months of additional microsurgical training. The plan is to organize two additional micro surgical missions for 2027, with an emphasis on further teaching and patient care.
We found that there is a tremendous interest and need for microsurgeons to assist Ukrainian surgeons in all forms of reconstructive services.

**FA26/363—Reconstruction**
**Reconstructing a Full Thickness Scalp Avulsion Defect with the Avulsed Scalp Tissue-Replacing Like with Like**
Janice Thanga Raj ^1^, Hau Tan ^1^ and Jeremiah See ^1^
*^1^* *Hospital Raja Permaisuri Bainun, Ipoh, Malaysia*
**Objectives**:
  (1) Proper storage of avulsed scalp tissue for graft
  (2) Replacing like with like tissue; hair bearing tissue
**Materials and Methods**: Case report
**Results**: We present a case of 17-year-old lady with traumatic scalp avulsion during her go kart sports sustaining full thickness defect 20 × 15 cm scalp defect exposing the pericranium. The avulsed scalp tissue was not well preserved with unfavourable vessels for anastomsis. Wound debridement and thick split skin graft (SSG) was harvested with hair follicle from the avulsed scalp tissue and used to reconstruct the scalp defect. On day 5, graft was well taken and on day 15 we noticed new hair growth from thick SSG
Scalp avulsion commonly occurs in young patients due to entanglement of long hair. The injury commonly occurs in loose areolar tissue layer, separating galea from pericranium. Microsurgical replantation of the well preserved avulsed scalp is the best treatment option to prevent alopecia and minimize scaring. In event of poorly preserved avulsed scalp tissue, thick SSG can be used with good results of hair bud growth.
**Conclusion**: Thick SSG with hair follicles harvest can be used as reconstruction option for poorly preserved avulsed scalp tissue and unfavourable vessels for microanatomies with acceptable hair growth.

**FA26/364—Reconstruction**
**Reconstruction After Resection of Head and Neck Skin Malignancy Defect Involving More Than One Subunit or Exposing Vital Facial Structures—Our Experience**
Janice Thanga Raj ^1^, Hau Tan ^1^, Nursheila Izrin ^1^ and Jeremiah See ^1^
*^1^* *Hospital Raja Permaisuri Bainun, Ipoh, Malaysia*
**Objectives**:
  (1) To achieve complete resection of skin malignancy
  (2) To restore facial aesthetic and provide coverage of vital structures with various tailored reconstruction options
**Materials and Methods**: Retrospective record and Case series
**Results**: We would like to present our experience of resection and various options of reconstruction defect involving more than one subunit defect or exposing vital structures (facial nerve, facial bone or scalp, orbit, ear meatus, dura) in head and neck skin malignancy from 2018–2025. Post reconstruction follows up of patients regarding recurrence rate, complications and patient’s satisfaction are included.
These complex defects exposing vital structures require a tailored reconstruction to restore facial aesthetic and function besides providing protection of vital structures. These can only be achieved with complete surgical resection of the primary malignancies, followed by a reconstruction options using local, regional or free tissue transfer.
**Conclusion**: Analytical planning based on function deficit and defect size during reconstruction will ensure satisfactory results and good long-term outcome

**FA26/368—Reconstruction**
**Computer-Assisted Surgical Planning for Soft Tissue Reconstruction in Complex Head and Neck Defects**
Marcus Couey ^1,2^, Ross Camiel ^1,2^, Owen Neuburger ^2^, Artem Kaliaev ^2^, Ali Elzieny ^2^, Osamu Sakai ^3,4^ and Baber Khatib ^5,6^
*^1^* *Boston University Goldman School of Dental Medicine, Boston, MA, USA*
*^2^* *Boston Medical Center, Boston, MA, USA*
*^3^* *Massachusetts General Hospital, Boston, MA, USA*
*^4^* *Harvard Medical School, Boston, MA, USA*
*^5^* *Head and Neck Surgical Associates, Portland, OR, USA*
*^6^* *Legacy Emanuel Medical Center, Portland, OR, USA*
**Objectives**: To develop and evaluate a novel computer-assisted surgical planning (CASP) workflow for soft tissue reconstruction in complex head and neck defects. This study aims to extend the principles of virtual surgical planning beyond osseous reconstruction to enable preoperative visualization and simulation of soft tissue contour and flap design.
**Materials and Methods**: Patient-specific imaging data (CT and PET/CT) were segmented using open-source software and imported into a professional 3D design platform. A customizable three-dimensional “analog” model of the head and neck was created, simplifying normative internal and external anatomy into a manipulable polygonal framework. This analog was conformed to patient-specific anatomy using skeletal and soft tissue landmarks. In collaboration with a professional animator, a reconstructive surgeon iteratively modified the model to simulate postoperative contours that could be achieved with flap reconstruction. Virtual resections were performed, and soft tissue flap dimensions were derived from the simulated defect. The workflow was applied to one retrospective case and five prospective cases.
**Results**: This approach enabled successful integration of patient-specific imaging with a customizable 3D analog, allowing visualization of anticipated soft tissue defects and the corresponding shape, contour and orientation of soft tissue reconstructions. The surgeons were able to simulate flap design, estimate skin paddle dimensions, and evaluate reconstructive strategies preoperatively. Early application in retrospective and prospective cases demonstrated feasibility and utility in surgical planning, particularly for through-and-through defects involving multiple tissue surfaces.
**Conclusion**: This novel CASP workflow facilitates preoperative visualization of soft tissue reconstruction by focusing on achievable surgical outcomes rather than exact anatomic replication. It has the potential to improve operative efficiency, enhance two-team coordination, and serve as a valuable educational tool. While currently labor-intensive and not intended as an exact templating method, this approach represents a promising step toward more comprehensive virtual planning in reconstructive microsurgery.
Conflict of interest disclosure
Receipt of grants/research supports: Oral and Maxillofacial Surgery Foundation Research Grant

**FA26/389—Reconstruction**
**Immediate Reconstruction of Orofacial Defects Using the Temporalis Muscle Flap: Aesthetic and Functional Outcomes**
Haider Alalawy ^1^ and Maha Mnahi ^1^
*^1^* *Gazi Alhariri Hospital Medical City, Baghdad, Iraq*
**Objectives**: The temporalis muscle flap represents a well-established reconstructive option for oral and facial defects. Due to its robust vascularity, anatomical proximity, and technical reliability, its indications have expanded substantially since its initial description.
**Materials and Methods**: This prospective study included 15 patients who underwent immediate reconstruction with a temporalis muscle flap following ablative surgery. The primary outcome measure was flap survival. Secondary outcome measures included postoperative trismus, donor-site contour deformity (temporal hollowing) and other outcomes dependent on defect location. The maxilla/maxillary sinus was the primary site in 8 patients (53%), including 3 cases (20%) with orbital extension. Other primary sites included the buccal mucosa (n = 2), orbit (n = 1), ethmoid sinus (n = 1), lower eyelid (n = 1), and oropharynx (n = 1). All patients underwent immediate reconstruction with a temporalis muscle flap. Abdominal dermal fat grafting to correct temporal hollowing was performed in 6 patients (40%)
**Results**: The overall flap survival rate was 93.3% (14/15). One case (6.7%) of total flap loss occurred in a pregnant patient at her second trimester. No oro-nasal or oro-antral fistula was encountered in the healing periods, nor after radiotherapy for the maxillary defect. Reconstructive objectives in the orbital and cheek areas were also obtained. Clinically significant postoperative trismus was predominantly associated with buccal mucosa reconstruction. In contrast, patients undergoing maxillary, orbital, or cheek reconstruction did not demonstrate comparable severity of mouth-opening limitation. Temporal hollowing was most evident in patients undergoing maxillary reconstruction and was effectively camouflaged in all cases where dermal fat grafting was performed.
**Conclusion**: Immediate reconstruction with the temporalis muscle flap remains a reliable and versatile reconstructive option for upper oral cavity and midface defects. However, reconstruction of defects located inferior to the maxillary occlusal plane appears to be associated with an increased risk of postoperative trismus. Donor-site contour deformity can be addressed with dermal fat grafting.

**FA26/448—Reconstruction**
**Comparative Outcomes of End-to-Side vs. End-to-End Microanastomosis in Head and Neck Free Flap Reconstruction: A Retrospective 100-Case Study**
Joseph Kyu-hyung Park ^1,2^, Baek-Kyu Kim ^1,2^, Jun Seo Yoon ^1^ and Nakwon Choi ^1^
*^1^* *Seoul National University Bundang Hospital, Seongnam-si, Republic of Korea*
*^2^* *Seoul National University College of Medicine, Seoul, Republic of Korea*
**Objectives**: Free flap reconstruction in head and neck oncology has become a standard practice following tumor excision. Previous studies based on large cohort datasets have reported that approximately 30% of cases used the End-to-Side (ETS) technique for vessel microanastomosis offering satisfactory outcomes, with safety and efficacy comparable to End-to-End (ETE) anastomosis. This study aims to evaluate whether ETS anastomosis is associated with a lower incidence of flap complications requiring reoperation in contrast to traditional expectations.
**Materials and Methods**: In this retrospective analysis, 100 cases of head and neck tumor excision followed by free flap reconstruction by a single surgeon were reviewed from January 2021 to February 2026. The study evaluated the percentage of ETS and ETE techniques for microanastomosis across all cases, along with the incidence of flap complications necessitating reoperation.
**Results**: The cohort included 103 arterial and 114 venous microanastomoses. ETS was the predominant technique, employed in 89.3% of arteries and 78.8% of veins. Fourteen cases required reoperation (14%) due to complications: necrosis (28.6%), hematoma (21.4%), venous congestion (21.4%), arterial insufficiency (14.3%), and infection (14.3%). Among the 30 vessels involved in these complication cases, the ETE technique was used in 43.3% (13/30), which is significantly higher than the overall ETE utilization rate of 16.6%. Statistical analysis revealed that the odds of requiring reoperation were significantly higher in the ETE group compared to the ETS group (OR: 2.60; 95% CI: 1.50 to 4.50; *p* < 0.05).
**Conclusion**: This study suggests that ETS anastomosis may offer significant advantages over ETE anastomosis, including lower complication rates, particularly in head and neck reconstruction following tumor excision. Further studies with larger cohorts are warranted to validate these findings and solidify ETS as a preferred method in microsurgical head and neck reconstruction.

**FA26/456—Reconstruction**
**A Digital Workflow for Bar-Supported Implants in Fibula Free Flap Mandibular Reconstruction: A Case Report**
Aditya Amrullah ^1^
*^1^* *Faculty of Dentistry Dental Hospital University Indonesia, Jakarta, Indonesia*
**Objectives**: Rehabilitating mandibular defects with dental implants after fibula free flap (FFF) reconstruction remains challenging due to altered bone morphology and non-keratinized, mobile soft tissue. Bar-supported prostheses improve load distribution and retention, but optimal implant positioning and peri-implant soft tissue conditioning are critical for long-term success. Digital workflows combining virtual planning, guided implant placement, and CAD/CAM fabrication offer enhanced accuracy. This case illustrates a fully digital approach to bar-supported implant rehabilitation with highlighting solutions for soft tissue complications.
**Materials and Methods**: A 25-year-old female underwent hemimandibulectomy and FFF reconstruction for an odontogenic keratocyst, resulting in an edentulous mandibular body. Clinical examination revealed adequate fibular bone height/width but excessive soft tissue thickness and a shallow vestibule. Using a guided surgical protocol, three implants were placed, each with bicortical anchorage. A complete digital workflow was implemented: virtual implant planning, a 3D-printed surgical guide, and intraoral scanning. Custom PEEK healing abutments and multi-unit abutments guided peri-implant soft tissue maturation. CAD/CAM-designed bar framework and a bar-retained fixed acrylic prosthesis were delivered as follows. 5-month follow-up, the prosthesis remained stable, with no implant loss, pain, or recurrent infection. Patient-reported satisfactory with function and esthetics.
**Results**: Implant survival in fibular grafts reaches high rates when bicortical engagement and preoperative planning are optimized. However, the non-keratinized, mobile skin paddle mucosa increases the risk of peri-implant tissue overgrowth and compromised hygiene. This case demonstrates guided placement enables accurate bar framework fit, simultaneous vestibuloplasty and flap thinning reduce but do not eliminate soft tissue complications, and laser revision of mucosal overgrowth is a reliable rescue measure.
**Conclusion**: Multidisciplinary planning, proactive soft tissue management, and post-operative laser revision when needed contribute to predictable long-term outcomes. This case underscores the value of a fully digital pathway—from virtual planning to final CAD/CAM bar fabrication—in managing complex, thin-soft-tissue reconstructions.

**FA26/459—Reconstruction**
**Single Stage Bony Reconstruction of Left Total Maxillectomy & Hemi Mandibulectomy Bone Defect with Single Fibula Flap**
Rahul Kapoor ^1^
*^1^* *Blk-Max Superspeciality Hospital, Karol Bagh, New Delhi, India*
**Objectives**: Single stage bony reconstruction of left total maxillectomy & hemi mandibulectomy bone defect with single fibula flap.
**Materials and Methods**: 2 patients case study done in blk—max superspeciality hospital, New Delhi, India by single surgeon. Both had undergone total maxillectomy including orbital floor removal. First case—reconstruction done before radiation treatment and second done post radiation—both showcasing utilisation of whole length of fibula for proper cosmesis and soft tissue support. In one case anteriolateral thigh flap harvested was utilised for soft tissue reconstruction as well as flow through flap avoiding need for vein graft. In another case latissmus dorsi flap was utilised for cover and vein loop with additional vein graft utilised for flap revascularisation.
**Results**: in both the cases patients had uneventful recovery. they followed their normal lifestyle. Follow up period was six years post radiation in first case. patient had plate exposure at lateral canthus and probable recurrence / new finding. patient couldn’t come for treatment due to financial issues. Second case had passed away 2 years after surgery from cardiovascular cause.
**Conclusion**: These cases showcase need to preserve/utilise whole length of fibula bone and avoid two stage reconstruction or simultaneous harvest of double bone flap to provide adequate cosmesis and support for aesthetic reconstruction.

**FA26/480—Reconstruction**
**Bridging the Gap Between Patient-Reported Outcome Measures (PROMs) and Current Approaches For Zygomaticomaxillary Complex Fracture Management: A Literature Review**
Maram Ismail ^1^
*^1^* *Ain Shams University, Cairo, Egypt*
**Objectives:** ZMC fracture management has predominantly relied on objective morphometric analysis to restore anatomical alignment and symmetry. However, the use of heterogeneous PROM tools in the literature reveals a significant gap between current decision-making and patient satisfaction. This review highlights this gap by analysing the current evidence on the use of PROMs in ZMC fracture management and exploring their potential role as essential predictive tools that could guide future management.
**Materials and Methods**: A narrative review was conducted to investigate recent incorporation of PROMs within ZMC fracture management in literature. We searched the database (PubMed, Google Scholar) for articles published in the last two decades, including retrospective studies, prospective cohorts, and meta-analyses. Furthermore, this review explores emerging conceptual frameworks from observational studies that integrate PROMs into predictive clinical algorithms.
**Results**: Studies using PROMs to assess the success of ZMC fracture management, which are predominantly retrospective, most commonly included generic health-related quality-of-life surveys and custom symptom questionnaires, and such as **Hospital Anxiety and Depression Scale (HADS), Oral Health Impact Profile (OHIP-14), and the Short Form (SF) survey**. Patient satisfaction is generally high and often independent of treatment modality or surgical approach. Although there is a distinct lack of PROM integration within current ZMC decision-making algorithms, emerging studies have successfully utilized PROM-driven machine learning models to forecast long-term patient satisfaction.
**Conclusion**: PROMs are used heterogeneously to investigate the outcome of ZMC fracture management, yet they predominantly show high overall satisfaction and a marked mismatch between surgeon and patient perspectives. The suggested introduction of PROMs as a predictive rather than descriptive tool with potential integration with ZMC fracture morphometric analysis and machine learning models could provide a future benchmark a more holistic patient-centred strategies.

**FA26/484—Reconstruction**
**Free ALT Flap Reconstruction for the Treatment of Severe Facial Asymmetry: A Case Seies**
Maram Ismail ^1^
*^1^* *Ain Shams University, Cairo, Egypt*
**Objectives**: Significant facial asymmetry causes both aesthetic, as well as functional problems. Deficiencies of both the facial skeleton and the overlying soft tissue must be addressed to achieve the optimal correction. Microsurgical free tissue transplantation using the anterolateral thigh (ALT) adiopfascial flap is a reliable technique for soft tissue restoration in severe grades of facial asymmetry. They can be utilized independently or in conjunction with other techniques when necessary.
**Materials and Methods**: Two cases of severe hemifacial atrophy were addressed with the use of free ALT microsurgical transfer. Preoperative and postoperative computerized tomography scans were obtained for a detailed assessment of the facial skeleton. Postoperative follow-up for up to two years was done to monitor **flap viability** in addition to observing the presence of complications such as **vascular compromise, flap atrophy, donor site morbidity, and chronic inflammation**. Objective evaluation of **symmetry**, and **long-term aesthetic results were done with the help of customized patient satisfaction** surveys.
**Results**: The follow up post-operative period of up to two years revealed adequate facial volume restoration and minimal complications rate. Satisfactory aesthetic contour was achieved in the reported two cases.
**Conclusion**: Our findings highlight the versatility of free ALT flap in addressing complex facial asymmetry and restoring Free tissue transfer using the ALT flap provides a reliable and stable method of volume restoring and is associated with increased patient satisfaction in severe facial deformities.

**FA26/489—Reconstruction**
**Emergency Resection and Immediate Free Flap Reconstruction in Bleeding Paediatric Head and Neck Arteriovenous Malformations: Lessons from Four Challenging Cases**
Hui Yuan Lam ^1^, Mohammad Ali Mat Zain ^2^ and Khai Luen Koh ^1^
*^1^* *Plastic And Reconstructive Surgery, Hospital Kuala Lumpur, Kuala Lumpur, Malaysia*
*^2^* *Plastic Reconstructive Surgery, Kuala Lumpur, Malaysia*
**Objectives:** To evaluate the feasibility, surgical challenges, and clinical outcomes of emergency single-stage resection with immediate microvascular free flap reconstruction in paediatric patients presenting with life-threatening head and neck arteriovenous malformations.
**Materials and Methods:** Four paediatric patients presenting with severe haemorrhage from extensive head and neck AVMs underwent urgent preoperative embolization followed by radical resection and immediate microvascular free flap reconstruction. Operative challenges, perioperative complications, and clinical outcomes were retrospectively reviewed to identify key technical and management insights.
**Results:** Haemorrhage was successfully controlled in all cases. Tracheostomy was performed in all patients to secure the airway in anticipation of significant postoperative facial oedema. Two patients with mandibular AVMs underwent reconstruction using free fibula flaps. One patient required a free anterolateral thigh flap following orbital exenteration for a periorbital AVM, and another underwent double free flap reconstruction after hemimandibulectomy. One case required revision of the microvascular anastomosis due to thrombosis, while another required conversion to a pedicled pectoralis major flap due to flap ischaemia. A further patient required secondary revision of the fibula flap due to implant exposure. All flaps ultimately survived, and there was no perioperative mortality. No early recurrence was observed.
**Conclusion:** Emergency resection with immediate free flap reconstruction is a feasible and effective option in selected paediatric patients with bleeding AVMs. Successful outcomes depend on careful anticipation of complications, meticulous surgical technique, and flexibility in intraoperative decision-making.

**FA26/495—Reconstruction**
**Microsurgical Reconstruction of Complex Scalp Defects: Case Series**
Jonathan Victor ^1^
*^1^* *Christian Medical College, Vellore, India*
**Objectives**: Extensive scalp defects caused by various etiologies often require free-tissue transfer. We aimed to review our experience in the reconstruction of extensive scalp defects with microvascular flap cover.
**Materials and Methods**: A retrospective analysis was performed at a tertiary hospital on all patients with extensive scalp defects that were reconstructed with free microvascular free flaps from January 2019 to January 2026. 18 patients, 12 males and 6 females, were included in the study.
**Results**: 18 free-tissue transfers were used to reconstruct the extensive scalp defects. The range of the age of the patients was from 8–68 years.11 cases were done for oncological resections and 6 cases were done for post burn defects and 1 case was done following resection of a arteriovenous malformation. The flaps done included 12 anterolateral thigh flaps, 3 Latissmuss dosi flaps and 1 groin flap. 4 vein grafts were used and 1 AV loop was used. Flap survival rate was 100%.
**Conclusion**: Microvascular free-tissue transfer is the mainstay for the treatment of extensive scalp defects. We recommend microsurgical free flap use for challenging and complex cases, given the numerous advantages, including, reliability and safety.

**FA26/500—Reconstruction**
**Rethinking Mandibular Reconstruction: An Outcome-Based Evaluation of Patient-Specific Total Joint Replacement in Fibula-Reconstructed Mandible**
Pravish Vishnudas ^1^, Lin Jacob ^1^, Jijo Joseph ^2^, Namitha Prem ^1^ and Thejas Girishan ^1^
*^1^* *Sree Anjaneya Institute of Dental Sciences, Kozhikode, India*
*^2^* *Malabar Medical College Hospital, Kozhikode, India*
**Objectives**: To evaluate the feasibility, surgical predictability, and functional outcomes of a single-stage reconstruction integrating a patient-specific mandibular implant with a total temporomandibular joint (TMJ) prosthesis in segmental mandibular defects including the condyle following ameloblastoma resection.
**Materials and Methods**: A patient diagnosed with mandibular ameloblastoma underwent segmental mandibulectomy including condylar resection. Reconstruction was performed in a single stage using a free fibula flap in combination with a patient-specific implant (PSI) incorporating a customized TMJ prosthetic component.
Preoperative planning involved virtual surgical planning (VSP), 3D modeling, and CAD-CAM fabrication of a customized PSI–TMJ construct designed to integrate with the fibula graft while restoring mandibular continuity, condylar position, and occlusal alignment. Intraoperative placement was performed using guided protocols to ensure precise adaptation, accurate joint articulation, and optimal biomechanical load distribution.
Outcome measures included:
  - Maximum interincisal opening (MIO)
  - Occlusal stability
  - Masticatory efficiency
  - Pain (VAS score)
  - Radiographic evaluation of implant positioning, fixation, and joint function
**Results**: The single-stage PSI–TMJ reconstruction demonstrated accurate intraoperative fit and stable fixation to the fibula graft. Postoperatively, the patient showed significant improvement in mandibular function with increased mouth opening, stable occlusion, and satisfactory masticatory efficiency. Pain was minimal, and no early complications such as infection, prosthetic loosening, or malposition were observed. Radiographic assessment confirmed appropriate anatomical alignment and functional articulation of the joint.
**Conclusion**: Single-stage integration of a patient-specific mandibular implant with a total TMJ prosthesis in fibula-based reconstruction is a predictable and effective approach for simultaneous restoration of mandibular continuity and joint function. This technique overcomes the inherent limitations of conventional fibula reconstruction in condylar defects and represents a significant advancement toward precision-driven, function-oriented mandibular rehabilitation.

**FA26/508—Reconstruction**
**Handmade Acrylic Plate Placement Guide For The Reconstruction of Mandibular Defects, A Case Series**
Aimiel Blessa Amorado ^1^
*^1^* *Jose R. Reyes Memorial Medical Center, Metro Manila, Philippines*
ABSTRACT
**Background**: Mandibular reconstruction following segmental mandibulectomy remains surgically challenging due to the need to restore mandibular contour, occlusion, and facial symmetry. Intraoperative adaptation and positioning of reconstruction plates are often time-consuming and technically demanding, particularly in fibular free flap reconstruction. Advanced computer-assisted surgical guides have demonstrated improved surgical accuracy and reduced operative time; however, such technology remains costly and limited in accessibility in the local setting.
**Objectives**: This study aims to describe the use of a handmade acrylic Plate Placement Guide (PPG) in mandibular reconstruction and evaluate its potential impact on operative efficiency, flap ischemia time, plate placement accuracy, and surgical complications.
**Materials and Methods**: This retrospective case series includes patients who underwent segmental mandibulectomy with fibular free flap reconstruction using a handmade acrylic Plate Placement Guide at Jose R. Reyes Memorial Medical Center from January 2024 to March 2026. The guide was fabricated preoperatively using acrylic polymer powder mixed with self-curing acrylic monomer liquid and molded along the inferior aspect of a stereolithographic mandibular model and pre-bent titanium reconstruction plate based on patient computed tomography imaging. Collected variables included total operative time, reconstruction time, flap ischemia time, need for maxillomandibular fixation, intraoperative plate adjustment, plate placement accuracy, and surgical complications.
**Results**: Use of the Plate Placement Guide demonstrated feasibility in assisting intraoperative positioning of the reconstruction plate and restoration of mandibular alignment. Preliminary findings demonstrated minimal intraoperative plate adjustment and improved efficiency during mandibular reconstruction while maintaining satisfactory plate placement accuracy on postoperative imaging.
**Conclusion**: The handmade acrylic Plate Placement Guide is a low-cost and accessible adjunct for mandibular reconstruction that may improve intraoperative efficiency and facilitate restoration of mandibular occlusion in fibular free flap reconstruction. Further studies with larger sample sizes and comparative analyses are recommended to evaluate long-term outcomes, reproducibility, and overall surgical efficacy.

**2FA26/1009—Reconstruction**
**Superolateral Periosteal Flap as a Tarsal Substitute: A Defect-Stratified Framework for Single-Stage Reconstruction of Full-Thickness Lower Eyelid Defects**
Pattana Ongkasuwan ^1^
*^1^* *Nakornping Hospital, Chiang Mai, Thailand*
**Objectives:** To propose a defect-stratified single-stage reconstructive framework for full-thickness lower eyelid defects in which advanced forniceal conjunctiva provides the mucosal lining in every case, the superolateral periosteal flap serves as the tarsal substitute, and anterior coverage is selected by defect size and location.
**Materials and Methods:** A consecutive series of six patients with full-thickness lower eyelid defects (2019–2025) was reviewed. The inferior fornix conjunctiva was advanced superiorly in every case to provide the mucosal lining. In Phase 1 (n = 5; 50–75% medial/central defects) the superolateral periosteal flap was combined with a modified Mustardé flap, a transconjunctivally raised composite of skin, muscle, tarsus and conjunctiva advanced medially. In Phase 2 (n = 1; total defect, 30 × 35 mm) the periosteal flap was extended along the superior orbital rim to within 2–3 mm of the supraorbital notch, secured to the medial canthal tendon, and paired with a cheek rotation flap providing skin coverage. The lateral canthal attachment was preserved as the pedicle in both phases.
**Results:** Six patients (3 male, 3 female; age 62–90 years) underwent reconstruction for basal cell (n = 5) or basosquamous (n = 1) carcinoma. Histological margins were tumor-free in all cases. Follow-up ranged 3–60 months (Phase 1 mean 20 months; Phase 2 limited by unrelated patient death). No major functional complications were observed, including ectropion, entropion, lagophthalmos, symblepharon, or flap failure. No secondary revisions were required. Stable eyelid position and binocular vision were preserved in all patients.
**Conclusion:** A three-component single-stage reconstruction comprising universal forniceal conjunctival advancement, the superolateral periosteal flap as a tarsal substitute, and location-specific anterior coverage is proposed as a reconstructive framework for full-thickness lower eyelid defects, enabling single-stage repair with preserved binocular vision while avoiding multistage eyelid occlusion.

**2FA26/1063—Reconstruction**
**Functional and Postoperative Outcomes with Autogenous Non-Vascularized Rib Graft in Patients with Mandibular Defects at Western Visayas Medical Center**
Carlito Altimo ^1^
*^1^* *Western Visayas Medical Center, Iloilo City, Philippines*
Objectives:
  1. To determine the demographic profile of patients with mandibular defect who underwent non-vascularized autogenous rib graft
  2. To assess the functional outcome of patient who underwent autogenous rib graft
  3. To assess the postoperative complications of the recipient site who underwent autogenous rib graft
  4. To assess the postoperative complications of the donor site who underwent autogenous rib graft
**Materials and Methods**: A retrospective chart review was conducted on six patients who underwent mandibular reconstruction with autogenous non-vascularized rib grafts from 2019 to 2023. Data were collected on demographic characteristics, functional outcomes (jaw movement, swallowing, and mandibular function), and postoperative complications at both the recipient and donor sites.
**Results**: The mean age of the patients was 34.5 ± 9.29 years, with a majority of male participants (83.3%). Ameloblastoma (83.3%) was the most common preoperative diagnosis. Postoperatively, all patients (100%) could swallow without difficulty and chew soft foods, though 33.3% experienced pain on jaw movement, and none were able to chew hard foods. Complications at the recipient site included wound dehiscence (66.7%), partial bone necrosis (83.3%), and graft infection (33.3%), with no cases of total necrosis or tumor recurrence. No donor site complications such as morbidity, sensory loss, or delayed healing were observed.
**Conclusion**: Autogenous non-vascularized rib grafts provide a reconstructive option for mandibular defects, offering functional recovery with minimal donor site morbidity. However, the high incidence of recipient site complications underscores the importance of meticulous surgical technique and postoperative management. Further studies with larger sample sizes and long-term follow-up are recommended to refine protocols and improve patient outcomes.

**2FA26/1077—Reconstruction**
**One-Stage Approach for Maxillary Ameloblastoma: Resection, Free Flap Reconstruction, and Immediate Implant-Supported Rehabilitation**
Dheeratt Choengprapakorn ^1^
*^1^* *Dental faculty, KhonKhaen University, KhonKhaen, Thailand*
**Objectives**: To report a case of maxillary ameloblastoma treated with wide tumor resection, fibula free flap reconstruction, and immediate implant placement for oral rehabilitation.
**Materials and Methods**: A patient diagnosed with ameloblastoma of the maxilla underwent wide tumor resection followed by immediate fibula free flap reconstruction. Implant-supported oral rehabilitation was placed at the time of reconstruction without loading prosthesis. Clinical outcomes including osseointegration, masticatory function, aesthetics, and patient quality of life were assessed during follow-up.
**Results**: Successful osseointegration was achieved for all implants placed within the reconstructed fibula free flap. After flap was fully healed, temporary prosthetic was inserted, the patient demonstrated significant improvement in masticatory function, phonetics, and facial aesthetics. No major complications, implant failures, or tumor recurrence were observed during the follow-up period.
**Conclusion**: Wide resection combined with fibula free flap reconstruction and immediate implant placement is a viable and effective approach for managing maxillary ameloblastoma. This integrated strategy provides favorable functional and aesthetic outcomes while minimizing the number of surgical interventions. A multidisciplinary approach remains essential to optimize treatment outcomes in this complex patient population.

**2FA26/1100—Reconstruction**
**Upper Lip Reconstruction with Heterologous Cartilage Graft Following Custom Plate Exposure After Ossifying Fibroma Resection: A Case Report**
Diego Espinosa Huerta ^1^
*^1^* *IMSS, Mexico City, Mexico*
**Objectives**: We present a 21-year-old woman with a history of ossifying fibroma resection and placement of a custom-made plate in 2023, and Z-plasty of the upper lip and commissure in 2024 for postoperative synechiae. She presented with plate exposure and intraoral eversion of the upper lip. A combined maxillofacial and plastic reconstructive procedure was performed on 23 May 2026 at Centro Médico Nacional Siglo XXI, IMSS, Mexico City. Maxillofacial surgery shortened the previously placed custom plate and covered it with a local advancement flap. For upper-lip reconstruction, a central incision with subcutaneous tunneling was made, and a 2 × 0.5 cm heterologous cartilage plate was placed to support the philtrum and Cupid’s bow. Redundant lip tissue was excised, and anterior and posterior sutures were placed, achieving an aesthetically satisfactory immediate postoperative result.
**Materials and Methods**: Case Report
**Results**: Upper-lip reconstruction must address mucosa, muscle, skin, vermilion, and the philtral subunit. Several techniques have been described—including direct closure, V-Y and crescentic perialar advancement flaps, the Abbé and Abbé-Estlander cross-lip flaps, the Karapandzic flap, Bernard-Webster cheiloplasty, and free tissue transfer—each selected according to defect size, location, and the status of remaining tissue. Autologous (auricular, septal, costal) and heterologous cartilage grafts have been used as structural scaffolds to support the philtrum, vermilion, and Cupid’s bow, particularly in secondary cleft and post-burn deformities, providing predictable long-term projection. In our patient, a heterologous cartilage plate offered structural support and avoided donor-site morbidity, which is especially advantageous in patients who have already undergone multiple surgical interventions.
**Conclusion**: A staged, multidisciplinary approach combining maxillofacial plate management and plastic reconstruction with a heterologous cartilage strut is a feasible option for restoring upper-lip contour and function after complications of fibro-osseous tumor resection. Continued follow-up and definitive dental prosthetic rehabilitation are required to fully evaluate aesthetic and functional outcomes.

**2FA26/1116—Reconstruction**
**Maxillary Reconstruction Using an Orochi Flap–Style Combination of a Chimeric Scapular/Parascapular Flap and a Radial Forearm Free Flap: A Case Report**
Takakuni Tanaka ^1^ and Chihiro Matsui ^2^
*^1^* *Toyooka Public Hospital, Kyoto University, 1094Tobera Atoyooka City Hyogo Prefecture, Japan*
*^2^* *Juntendo University, Tokyo, Japan*
**Objectives**: Free-flap reconstruction of the maxilla istechnically demanding because multiple reconstructive requirements must be addressed simultaneously, including the long distance to recipient vessels, the need for well-vascularized tissue to obliterate the contamination-prone maxillary sinus, the requirement for vascularized bone for future dental implant placement, and the preference for a thin flap on the oral side for functional prosthetic rehabilitation. The Orochi flap concept, described by Koshima and colleagues, refers to a fabricated chimeric flap in which one or more secondary free flaps are anastomosed to the main pedicle of a primary or chimeric flap. We report a case of maxillary reconstruction using an Orochi flap–style design.
**Methods**: A 56-year-old woman with an odontogenic keratocyst of the right maxilla underwent partial maxillectomy followed by immediate reconstruction using a chimeric scapular/parascapular flap combined with a radial forearm free flap. The scapular bone was used for osseous reconstruction, while soft tissue was used for obliteration of the maxillary defect and intraoral lining.
**Results**: The combined free-flap transfer achieved stable reconstruction with satisfactory separation of the oral and sinonasal cavities and adequate tissue volume for defect obliteration. Osseous reconstruction enabled subsequent implant placement and prosthetic rehabilitation, resulting in recovery of occlusal function.
**Conclusion**: An Orochi flap–style combination of a chimeric scapular/parascapular flap and a radial forearm free flap may represent a useful reconstructive option for maxillary defects when long pedicle reach, sinus obliteration, vascularized bone transfer for future implants, and thin intraoral lining are all required simultaneously.
Conflict of interest disclosure
Receipt of grants/research supports: KAKENHI

**2FA26/1118—Reconstruction**
**Salvage Reconstruction of a Recurrent Tongue–Oral Floor Squamous Cell Carcinoma Using Radial Forearm Free Flap: A Case Report**
Natassya Tillasman ^1^, Agus Roy Rusly Hariantana Hamid ^2^, I Gusti Putu Hendra Sanjaya ^2^, Shita Diwyani Sudarsa ^2^, Astrinita Lestari Suyata ^2^ and Gede Wara Samsarga ^2^
*^1^* *Resident of Plastic Surgery Reconstruction and Aesthetic Division, IGNG Ngoerah General Hospital, Denpasar, Bali, Indonesia*
*^2^* *Division of Plastic Surgery Reconstruction and Aesthetic, IGNG Ngoerah General Hospital, Denpasar, Bali, Indonesia*
**ABSTRACT**
**Background**: Recurrent oral cavity squamous cell carcinoma after previous surgery and chemoradiotherapy presents a significant reconstructive challenge. Salvage surgery is often complicated by scarred tissue planes, altered vascular anatomy, trismus, limited local tissue availability, and impaired wound healing. The radial forearm free flap remains a reliable option for tongue and oral floor reconstruction because of its thin, pliable skin paddle, long vascular pedicle, and consistent vascular anatomy.
**Case Presentation:** A 55-year-old male presented with recurrent moderately differentiated invasive squamous cell carcinoma involving the posterior third of the tongue, palate, and oral floor. He had previously undergone subtotal glossectomy, wide excision of the palate, supramyohyoid neck dissection, and completed chemoradiotherapy with partial response. Intraoperative assessment revealed severe trismus with a maximum interincisal opening of approximately 1.5 cm. After tumor excision, a 6 × 2.5 cm intraoral defect was noted at the right anterior tongue and oral floor region. Reconstruction was performed using a left radial forearm free flap, inset through a sublingual tunnel. End-to-end microvascular anastomoses were performed between the radial artery and superior thyroid artery, the cephalic vein and external jugular venous branch, and one vena comitans to the superior thyroid vein. The donor site was covered with split-thickness skin graft from the left thigh.
**Discussion**: This case demonstrates the utility of radial forearm free flap reconstruction in a complex salvage setting. Its thin and pliable characteristics allowed precise intraoral inset despite limited access, while its reliable pedicle supported safe microvascular reconstruction in a previously treated neck.
**Conclusion**: Radial forearm free flap reconstruction is a dependable option for recurrent tongue–oral floor squamous cell carcinoma defects, particularly after prior surgery and chemoradiotherapy.
**Keywords:** Radial forearm free flap; salvage reconstruction; recurrent oral squamous cell carcinoma; tongue reconstruction; oral floor reconstruction; microvascular reconstruction; chemoradiotherapy; case report

**2FA26/1122—Reconstruction**
**Preserving the Smile After Sarcoma Ablation: Reconstruction of a Complex Upper Lip Defect**
Mohamad Shahrin Shahrul Zaman ^1^, Siti Fatihah Ramli ^2^, Nor Anis Razali ^2^, Muhamad Fadzlyazwad Kaider ^1^
*^1^* *Hospital Sultan Ismail Petra, Kuala Krai, Malaysia*
*^2^* *Hospital Raja Perempuan Zainab II, Kota Bharu, Malaysia*
**Objectives**: Low-grade myofibroblastic sarcoma (LGMS) is a rare deep seated soft tissue malignancy that exhibits broad anatomical distribution and rarely involves the upper lip. Reconstruction of upper lip defects following tumour ablation presents significant challenges as both require a balance of functional and aesthetic outcomes. This report emphasizes the surgical technique for complex upper lip defects using a combination of bilateral perialar advancement flaps and an Abbe flap following tumour ablation.
**Materials and Methods:** A 26-year-old Malay man presented with a one-month history of a progressively enlarging upper lip swelling associated with mild discomfort. Clinical examination revealed a firm, non-tender submucosal lesion measuring approximately 2 × 1 cm within the upper labial mucosa. Although the lesion was clinically suggestive of a benign pathology, histopathological assessment following excision established the diagnosis of low-grade myofibroblastic sarcoma.
Wide local excision achieved complete tumour removal with clear surgical margins. Tumour ablation resulted in a composite defect necessitating complex reconstruction. Bilateral perialar advancement flaps combined with an Abbe flap based on the lower labial artery were performed to reconstruct the central upper lip defect. The Abbe flap pedicle was separated two weeks following the initial reconstruction.
**Result:** Postoperatively, the patient demonstrated satisfactory oral competence, lip mobility, and speech. Good lip symmetry, vermilion continuity, preservation of philtral definition, and favourable facial harmony were achieved. Scars were well concealed within natural skin creases, with minimal donor-site morbidity. The postoperative course was uneventful, with no major complications or local recurrence during follow-up.
**Conclusion:** Reconstruction of extensive upper lip defects following tumour resection remains surgically challenging due to the need to restore both lip form and function. This case demonstrates that a combination of bilateral perialar advancement flaps and an Abbe flap can provide satisfactory functional and aesthetic outcomes, offering a reliable reconstructive option for complex upper lip defects.

**2FA26/1151—Reconstruction**
**Role of 3D Printing in Mandibular Reconstruction**
Coulibaly Konimba ^1^
*^1^* *Mohammed VI Foundation for Health Sciences, Mohammed VI University of Health Sciences (UM6SS), Mohammed VI International University Hospital and Cheikh Khalifa International University Hospital, Casablanca, Morocco*
**Objectives:** To evaluate the feasibility and clinical relevance of patient-specific 3D-printed prostheses in mandibular reconstruction following tumor resection. The study also aimed to assess the contribution of digital planning and additive manufacturing to improving surgical precision, functional outcomes, and personalization of treatment in maxillofacial surgery.
**Materials and Methods:**
This was a prospective, descriptive, and longitudinal study involving two patients. The study was conducted in the Departments of Oral and Maxillofacial Surgery at the Cheikh Khalifa International University Hospital and the Mohammed VI International University Hospital in Casablanca.
Study Population and Inclusion Criteria
The study included all patients diagnosed with a mandibular tumor requiring mandibular resection and reconstruction and who agreed to undergo reconstruction using a patient-specific 3D-printed prosthesis.
**Results:** Digital planning enabled accurate preoperative simulation and optimization of mandibular reconstruction. The use of patient-specific 3D-printed titanium prostheses facilitated precise adaptation to the mandibular defect and improved surgical predictability. Both patients underwent successful mandibular reconstruction with satisfactory functional and aesthetic outcomes. The technology reduced intraoperative adjustments and contributed to improved personalization of treatment. Despite these advantages, challenges related to cost, accessibility, and the absence of comprehensive regulatory frameworks remain barriers to wider implementation.
**Conclusion:** Mandibular reconstruction using 3D printing is a promising therapeutic option that enables more precise and personalized surgical interventions, providing more satisfactory outcomes than conventional mandibular reconstruction techniques.
Keywords: Maxillofacial Surgery, 3D Printing, Prosthesis, Titanium, Digital Technology.

**2FA26/1152—Reconstruction**
**Improved Volumetric Accuracy with One-Piece Milled Patient-Specific PEEK Implants in Inferomedial Blow-Out Fractures: A Comparative Study**
Jinsol Shim ^1^ and Dong Yeon Kim ^1^
*^1^* *The Catholic University of Korea, College of Medicine, St. Vincent’s Hospital, Suwon, Republic of Korea*
**Objectives**: Inferomedial orbital blow-out fractures present significant reconstructive challenges due to the complex 3D curvature of the orbital walls and the functional role of the inferomedial strut. This study aimed to evaluate the clinical efficacy and anatomical 26 accuracy of customized one-piece milled polyetheretherketone patient-specific implants compared with conventional manually contoured implants.
**Materials and Methods**: Patients who underwent primary reconstruction for unilateral inferomedial orbital fractures between 2019 and 2026 were divided into a PEEK PSI group (n = 18) and a manual-reconstruction group (n = 26). Reconstructive outcomes were assessed using CT-based 3D volumetric analysis. The primary endpoint was the absolute volumetric deviation from the mirrored contralateral orbit. Secondary endpoints included the volumetric restoration ratio, operative time, postoperative complications, and patient satisfaction scores.
**Results**: The PEEK PSI group demonstrated significantly higher anatomical precision, with a lower absolute volumetric deviation compared to the manual-reconstruction group (*p* < 0.001). The volumetric restoration ratio was closer to the ideal value of 1.0 in the PEEK group (1.006 ± 0.041) than in the manual group (1.079 ± 0.057) (*p* < 0.001). Operative time did not differ significantly between groups (*p* = 0.081), and no major implant-related complications occurred.
**Conclusion**: Customized one-piece milled PEEK PSIs may provide more accurate orbital volume restoration than conventional manually contoured implants in unilateral inferomedial blow-out fractures, while maintaining comparable functional outcomes and safety.

**2FA26/1162—Reconstruction**
**Orbital Nasolabial Flap for Reconstruction of Oral Cavity Defects: A Case Report and Surgical Perspective**
Fairouz Bekrentchir ^1^
*^1^* *University Hospital of Oran, Oran, Algeria*
**Objectives**: Reconstruction of oral cavity defects following oncologic resection remains challenging, particularly in recurrent tumors where local tissues are compromised. While free flaps are widely used, locoregional alternatives can provide effective reconstruction with reduced morbidity. The orbito-nasolabial flap is a reliable and versatile option, although its intraoral application remains underreported. This study aims to demonstrate the effectiveness of a tunneled, retrograde pedicled orbito-nasolabial flap for reconstruction of an intraoral defect following resection of recurrent gingivomaxillary squamous cell carcinoma.
**Materials and Methods**: We report the case of a 56-year-old chronic smoker diagnosed with locally advanced maxillary squamous cell carcinoma (T4aN0M0). The patient initially underwent tumor resection with bilateral selective cervical lymph node dissection. The postoperative course was marked by a local recurrence involving the gingivomaxillary region, extending from tooth 16 to 26. After wide excision of the recurrent lesion, reconstruction was performed using a retrograde pedicled orbito-nasolabial flap. The flap was elevated and transposed through a subcutaneous tunnel into the oral cavity to achieve defect closure. Surgical technique, flap viability, postoperative course, and functional outcomes were evaluated.
**Results**: The tunneled flap provided stable and adequate coverage of the defect, ensuring effective separation between the oral and nasal cavities. Postoperative recovery was uneventful, with no flap necrosis, infection, or dehiscence. Functional outcomes were satisfactory, particularly regarding speech and swallowing. Donor site morbidity was minimal, with acceptable aesthetic results.
**Conclusion**: The tunneled retrograde orbito-nasolabial flap is a simple, reliable, and minimally invasive option for intraoral reconstruction, especially in previously operated fields. It provides favorable functional outcomes with low morbidity and represents a pragmatic alternative to more complex reconstructive techniques, expanding the maxillofacial surgeon’s options.

**2FA26/1163—Reconstruction**
**Full-Thickness Reconstruction of the Nasal Dorsum Combining a Free Temporoparietal Fascia Flap, a Calvarial Bone Graft and a Forehead Flap**
Alicia Dean ^1^, Inmaculada Centella ^1^, Orlando Estevez ^1^, Abel Marin Martin ^1^ and Francisco Jesús Alamillos ^1^
*^1^* *Oral and Maxillofacial Surgery Department, University Hospital Reina Sofía, Córdoba, Spain*
**Objectives**: To highlight the role of a free temporoparietal fascia flap covered with a skin graft for inner lining in a full-thickness nasal dorsum reconstruction, used as a salvage option when the planned temporal artery posterior auricular skin (TAPAS) flap proved unfeasible.
**Materials and Methods**: A patient who had undergone oncological full-thickness resection of the nasal dorsum at another department, reconstructed with a silicone prosthesis, requested autologous reconstruction because of poor aesthetics and discomfort. Reconstruction was performed following the three-layer principle. The defect margins were freshened. A TAPAS flap was planned for the inner lining but proved unfeasible because the posterior auricular skin flap had no connection with the superficial temporal system; instead, a free temporoparietal fascia flap covered with a retroauricular skin graft was raised and microanastomosed to the right facial (nasolabial) vessels for the inner lining. For external coverage we used a paramedian supratrochlear (forehead) flap. Four weeks later, the forehead flap was partially elevated, a calvarial bone graft was fixed to the glabella with a miniplate as structural support, and the fascia flap was suspended to it. Four weeks later, the cutaneous flap was elevated, thinned and repositioned. Finally, four weeks later, the forehead flap pedicle was divided and inset.
**Results**: All flaps survived and the reconstructive stages were completed as planned. At 2.5 years’ follow-up the patient remained free of tumour recurrence and the nasal reconstruction remains stable. Aesthetics and patient comfort improved markedly.
**Conclusion**: A free temporoparietal fascia flap with a skin graft is a reliable inner-lining option for full-thickness nasal dorsum reconstruction and a useful salvage when a TAPAS flap is not feasible; combined with a calvarial bone graft and a forehead flap, it provides a reproducible reconstruction despite requiring several stages.
Conflict of interest disclosure:
Participation in a company sponsored speaker’s bureau: In 2021

**2FA26/1181—Reconstruction**
**From Radical Resection to Mandibular Preservation: Neoadjuvant Dabrafenib and Trametinib in BRAF V600E-Mutated Ameloblastoma: A Case Report**
Anders Torp Jensen ^1^
*^1^* *Department of Oral and Maxillofacial Surgery, Rigshospitalet University Hospital Copenhagen, Copenhagen, Denmark*
**Objectives**: Ameloblastoma is a locally aggressive odontogenic tumor traditionally managed by radical surgical resection, often requiring segmental mandibulectomy and complex reconstruction. The discovery of BRAF V600E mutations in a substantial proportion of ameloblastomas has created opportunities for targeted therapy. This case report demonstrates how neoadjuvant BRAF-targeted treatment can reduce tumor burden and facilitate a less invasive surgical approach while preserving mandibular continuity.
**Materials and Methods**: A 47-year-old man was diagnosed with a mandibular ameloblastoma harboring a BRAF V600E mutation. Neoadjuvant targeted therapy with Dabrafenib and Trametinib was administered for 16 months. Clinical and radiological evaluations were performed before and during treatment to assess tumor response. Initial CT imaging demonstrated a lesion measuring 24 × 70 × 30 mm. Following significant tumor regression, definitive surgical excision was undertaken. Histopathological examination was performed to assess treatment response and surgical margins.
**Results**: Targeted therapy resulted in marked clinical and radiological tumor shrinkage, substantially reducing the extent of the lesion and its mandibular involvement. The reduction in tumor volume enabled definitive surgical management without segmental mandibulectomy, continuity resection, mandibular reconstruction, or placement of osteosynthesis hardware. Mandibular integrity was preserved, and reconstruction with a free vascularized flap was avoided. Histopathological examination confirmed residual ameloblastoma with treatment-related changes, and complete excision was achieved with clear margins. No major perioperative complications occurred, and both functional and aesthetic outcomes were favorable.
**Conclusion**: This case highlights the potential role of neoadjuvant BRAF-targeted therapy in the management of BRAF V600E-mutated ameloblastoma. Significant tumor downsizing may allow for less extensive surgery, preservation of mandibular continuity, and avoidance of complex reconstruction procedures. Targeted therapy may represent a valuable adjunct in selected patients and warrants further investigation in larger clinical studies.

**2FA26/1210—Reconstruction**
**OMFS-Led Microvascular Reconstruction in a Resource-Limited Rural Hospital: Report of Two Consecutive Successful Cases**
Thrrishala Ramachandran ^1^, Yuen Wern Tham ^1^, Jeremy Ju Kuan Lee ^1^, Azuriyati Baharom ^1^, Steve Maisi ^2^ and Rubinderan Muthusamy ^3^
*^1^* *Department of Oral and Maxillofacial Surgery, Hospital Duchess of Kent, Sandakan, Malaysia*
*^2^* *Department of Oral and Maxillofacial Surgery, Hospital Lahad Datu, Lahad Datu, Malaysia*
*^3^* *Department of Oral and Maxillofacial Surgery, Hospital Queen Elizabeth II, Kota Kinabalu, Malaysia*
**Objectives**: Microvascular free flap reconstruction is the gold standard for restoration of complex head and neck defects following ablative surgeries. However, establishing such services in rural settings remains challenging due to resource limitations and technical demands. This case series reports outcomes of the first two consecutive free flap reconstructions performed by an OMFS-led team in a rural tertiary hospital.
**Materials and Methods**: A retrospective review was conducted on two patients who underwent microvascular reconstruction in a rural tertiary hospital. The first patient, diagnosed with left mandibular ameloblastoma, had a left hemimandibulectomy and immediate reconstruction using an osteomyocutaneous fibula free flap. The second patient, diagnosed with lower lip squamous cell carcinoma, underwent wide local excision of primary tumor and immediate reconstruction using a radial forearm free flap. Postoperative outcomes, complications, and functional recovery were evaluated.
**Results**: Both free flaps survived successfully, yielding a flap survival rate of 100%. The fibula free flap patient recovered uneventfully and achieved independent ambulation by postoperative day 7, with satisfactory oral function and mastication. The radial forearm free flap patient developed postoperative bleeding resulting in a threatened flap on day 3. Prompt recognition and surgical intervention successfully salvaged the flap, preventing flap loss. The patient subsequently achieved good oral competence and intelligible speech. Both patients were discharged after a 2-week hospital stay with favorable functional outcomes.
**Conclusion**: This case series demonstrates the feasibility of establishing an OMFS-led microvascular reconstruction service in a resource-limited rural hospital. Careful patient selection, meticulous surgical technique, vigilant postoperative monitoring by a well-trained team, and timely intervention contributed to successful outcomes. These cases highlight the potential for expanding access to advanced head and neck reconstructive services in previously underserved regions.

**2FA26/1213—Reconstruction**
**Optimising Aesthetic and Functional Outcomes Using INTEGRA for Large Complex Facial Defects**
Elaine Huang ^1^ and Tee Sin Lee ^1^
*^1^* *Changi General Hospital, Singapore, Singapore*
**Objectives**: Facial reconstruction is challenging as it requires meticulous texture matching and tissue replacement to achieve optimal aesthetic and functional outcomes. There are limitations in utilising single modality reconstruction options for large complex multilayer facial defects: Grafts do not take well on bare bone, local flaps are limited by blood supply and proximity to critical structures and free flaps may be overly aggressive and have suboptimal cosmetic outcomes.
Integra Dermal Regeneration Template (IDRT) is commonly used for wound reconstruction. However, there is limited literature on its outcomes for large complex facial defects. Our experience highlights the use of IDRT (1) as an excellent intermediate graft providing temporary coverage for exposed bone and nerves in large complex facial defects after tumour resection and (2) as a well-vascularised bed to optimise subsequent Full Thickness Skin Graft (FTSG) survivability.
**Materials and Methods**: Two patients with large Squamous Cell Carcinoma (SCCs) over the zygomaticotemporal regions of the face underwent wide excision of tumour with 1 cm margins and superficial parotidectomy. The resultant defects measured 6–8 cm, with exposure of underlying bone and distal branches of the facial nerve. IDRT was used to reconstruct the defects, with the silicone layer removed after three weeks. Formal second stage reconstruction was performed using FTSG with excellent graft survival.
**Results**: At 18–24 months after surgery, no revision and/or adjunct procedures were required and both patients demonstrated significant satisfaction with the overall outcomes.
**Conclusion**: IDRT is an effective intermediate graft and optimises aesthetic and functional outcomes in patients with large complex facial defects.

**2FA26/1228—Reconstruction**
**Fatal Progressive Dysautonomia Following Periorbital Basal Cell Carcinoma Resection and Anterolateral Thigh Free Flap Reconstruction in a Patient with Xeroderma Pigmentosum: A Case Report**
Maria Limpomo ^1^
*^1^* *dr Soetomo General Hospital, Head and Neck Surgery Division, Surabaya, Indonesia*
**Objectives**: Xeroderma pigmentosum(XP) is a rare autosomal recessive disorder characterized by defective DNA repair and extreme sensitivity to ultraviolet radiation, resulting in a markedly increased risk of cutaneous malignancies. Basal cell carcinoma(BCC) is one of the most common skin cancers associated with XP. Progressive neurodegeneration and dysautonomia are rare but potentially fatal manifestations of XP. This report describes a patient with advanced periorbital BCC associated with XP who developed fatal progressive dysautonomia following surgical resection and reconstruction.
**Materials and Methods**: A 65-year-old woman with XP presented with a recurrent ulcerative lesion involving the right periorbital region. Histopathological examination confirmed nodular basal cell carcinoma infiltrating the reticular dermis(Clark level IV) with a depth of invasion of 7 mm. Staging investigations revealed no distant metastasis. The patient was diagnosed with right periorbital BCC(T4aN0M0) and underwent wide excision, orbital exenteration, and reconstruction using an anterolateral thigh(ALT) free flap. Clinical findings, perioperative management, postoperative course, and outcomes were reviewed.
**Results**: The surgical procedure was completed successfully, and the ALT free flap remained viable throughout postoperative monitoring. During the first postoperative day, the patient developed persistent unexplained tachycardia despite adequate fluid status and absence of significant pain. Progressive dysphagia and inability to clear oral secretions subsequently occurred, resulting in desaturation episodes requiring suctioning. Several hours later, the patient experienced sudden autonomic instability characterized by transient hypertension followed by profound hypotension, bradycardia, apnea, and cardiac arrest. Despite immediate intervention, the patient died. The clinical course was consistent with progressive dysautonomia associated with XP-related neurodegeneration.
**Conclusion**: Fatal dysautonomia should be recognized as a potential complication of XP, particularly in patients undergoing major head and neck surgery. Early signs such as unexplained tachycardia, dysphagia, secretion retention, and autonomic instability may indicate progressive neurodegeneration. Intensive postoperative monitoring and airway surveillance should be considered in XP patients to facilitate early recognition of life-threatening autonomic dysfunction.

**2FA26/1231—Reconstruction**
**Reconstructing Extensive Composite Facial Defects Using Double Free flapS**
Devesh Verma ^1^
*^1^* *pims City Hospital, Udaipur, India*
**Objectives**: To illustrate the benefit of implementing double free flaps in extensive composite facial defect
**Materials and Methods**: Total of 42 patients underwent double free flap reconstruction from duration sept 22 to June 26. Indication were (1) extensive composite defects involving bone, oral mucosa, external skin, and soft tissue, which cannot be adequately reconstructed with one composite flap; (2) huge coverage and intraoral lining defects that cannot adequately be resurfaced with one large cutaneous or Myocutaneous flap (3) difficult in setting with a single free flap because of extent and tridimensional nature of the defect.
**Results**: The predominant combination was that of free fibula osteo-cutaneous flap with free anterolateral thigh (ALT) fascio-cutaneous flap (n = 31). Defects warranting the use of double free flaps were extensive bone and soft tissue defects (n = 35), defects involving soft tissue only (n = 3), (c) defects encountered during secondary reconstruction (n = 7) and osteoradionecrosis (n = 7). Out of 42 patients, 13 patients had prior history of radiotherapy. Flap survival rate was 91%. Median follow-up period was 13 months. One patient expired owing to severe sepsis. Complete loss of both flaps was seen in one case
**Conclusion**: Double free-flap reconstruction can provide better oromandibular function and a more socially acceptable appearance as they give us freedom of choosing best osseous and soft-tissue elements may be independently selected, yielding appropriate tissue characteristics for ideal defect reconstruction. They also impose greater morbidity on patients and is time consuming for surgeons. With proper patient selection and planning and the use of 2 surgical teams, reduce length of surgery and complication rates.

**2FA26/1239—Reconstruction**
**The Used of Customized Titanium Plate in the Reconstruction of the Mandibular Defect Secondary to Gun Shot Wound**
Mario Esquillo ^1^
*^1^* *de Los Santos Medical Center, Quezon City, Philippines*
**Objectives**: The purpose of this presentation is to discuss a workflow for the jaw reconstruction in a gunshot wound patient, using virtual planning of a 3D model and fabrication of customized titanium plate.
**Materials and Methods**: This is a case of a 27 year old male who was referred to our department complaining of limited mouth opening and difficulty in mastication due to malocclusion secondary to a gunshot wound reconstruction. The patient had undergone wound debridement and soft tissue management previously. CT scan revealed the malunion and non-union of comminuted fracture on the right maxilla, orbital wall and floor, zygoma condyle and angle and symphysis of the right mandible resulting to posterior crossbite.
STL model was used to reconstruct the mandibular defect and correct the crossbite. 3D planning of customized implant was facilitated together with the computer engineer and final design was fabricated. Reconstruction of the mandible was done using fibula flap with reinforcement of customized titanium implant. During the surgical procedure, efficiency and ease of placement of the custom plate was noted.
**Results**: The contemplated treatment plan was executed well due to the precise fitting of the implants during the surgical procedure. Significant decrease in surgical time was observed. Patient recovery was uneventful and marked improvement on mouth opening as well as occlusion. Masticatory function was restored resulting to increase weight of the patient.
**Conclusion**: 3D model and fabrication of customized titanium plate provides precise and efficient reconstruction of a severe mandibular defect secondary to gunshot wound resulting to a predictable esthetic and functional outcome.

**2FA26/1244—Reconstruction**
**Angiogenic and Regenerative Biomarker Responses of Mesenchymal Stem Cells Exposed to Decellularized Freeze-Dried Bovine Periosteum**
Kornelius Naben ^1^, Indra Mulyawan ^1^, Coen Danudiningrat ^1^
*^1^* *Faculty of Dentistry Airlangga University, Surabaya, Indonesia*
**Objectives**: To evaluate and correlate *Matrix Metalloproteinase-9* (MMP-9) and *Vascular Endothelial Growth Factor* (VEGF) levels as biomarker of tissue regeneration in *Mesenchymal Stem Cells* (MSCs) exposed to *Native Bovine Periosteum* (NBP) and *Decellularized Freeze-Dried Bovine Periosteum* (DFDBP) as a promising alternative periosteal graft for maxillofacial reconstruction.
**Materials and Methods**: This true experimental post-test-only study cultured MSCs with NBP and DFDBP. Supernatants were collected on days 3 and 5, then analyzed via ELISA to quantify VEGF and MMP-9. Following Shapiro-Wilk and Levene’s tests for data distribution and homogeneity, statistical analysis was performed using the Independent T-test for group comparisons and the Pearson test for correlation (*p* < 0.05).
**Results**: On day 3, MMP-9 levels were comparable between the NBP and DFDBP groups (*p* > 0.05), while VEGF was significantly higher in NBP (OD: 1.350 ± 0.108; *p* < 0.05). On day 5, MMP-9 levels significantly increased in DFDBP compared to NBP (OD: 1.126 ± 0.080 vs. 0.988 ± 0.094; *p* < 0.05). Meanwhile, VEGF levels decreased across both groups by day 5 with no significant difference (*p* > 0.05). Furthermore, Pearson analysis indicated a weak, non-significant positive correlation between MMP-9 and VEGF in both NBP (r = 0.322, *p* = 0.364) and DFDBP (r = 0.382, *p* = 0.276) groups.
**Conclusion**: DFDBP is a promising alternative periosteal graft for maxillofacial reconstruction, modulating the expression of MMP-9 and VEGF in MSCs to as biomarker of tissue regeneration to promote angiogenesis and bone regeneration.
Keywords: Bovine Periosteum, Mesenchymal Stem Cells, MMP-9, VEGF

**2FA26/1264—Reconstruction**
**Virtual Surgical Planning, a Patient-Specific Implant Designed for Navigation, and Surgical Navigation for Silent Sinus Syndrome**
Francisco Jesús Alamillos ^1^, Abel Marin Martin ^1^, Orlando Estevez ^1^ and Alicia Dean ^1^
*^1^* *Department of Oral and Maxillofacial Surgery, University Hospital Reina Sofía, Córdoba, Spain*
**Objectives**: To present the combined surgical treatment of silent sinus syndrome using virtual surgical planning, a custom titanium patient-specific implant with navigation lines, and intraoperative navigation, in a rare case with right maxillary and ethmoid involvement.
**Materials and Methods**: A 47-year-old woman presented with progressive right enophthalmos and hypoglobus over 18 months, without trauma and with preserved visual acuity; she had undergone functional endoscopic sinus surgery elsewhere one year earlier. CT showed complete right maxillary and partial right ethmoid opacification, with downward displacement of the orbital floor and medialisation of the medial wall. A virtual plan was created by mirroring the healthy orbit, and a custom titanium implant was designed to restore the orbital floor, medial wall and volume, anchored to the infraorbital rim with three self-drilling screws and incorporating two grooves (“navigation lines”) for navigated checking. Treatment combined functional endoscopic sinus surgery with transconjunctival implant reconstruction. Navigation confirmed subperiosteal dissection (anatomical navigation), located the screw positions, and finally cheeking implant position by tracing the navigation grooves with a blunt probe. A forced duction test was performed before closure.
**Results**: Recovery proceeded without complications. The postoperative CT scan showed adequate drainage of the right maxillary and ethmoid sinuses, as well as proper reconstruction of the orbital floor and medial wall, with resolution of the preoperative diplopia. Superimposition of the preoperative and postoperative CT scans on the implant image revealed a misalignment of less than 1 mm in all three planes.
**Conclusion**: In this rare case of silent sinus syndrome with combined involvement of the maxillary and ethmoid sinuses, virtual planning, a navigation-ready custom implant, and intraoperative navigation allowed for accurate orbital reconstruction, correction of enophthalmos and hypoglobus, and resolution of diplopia. This combination may offer greater precision and predictability than conventional approaches; larger series are needed to confirm its value.
Conflict of interest disclosure:
Participation in a company sponsored speaker’s bureau: In 2021

**2FA26/1285—Reconstruction**
**Traumatic Amputation of Anterior One Third of the Tongue Following Human Bite. A Case Report**
Oum-Kalsoum Zakaria Adam ^1^, M O Boamah ^1^ and G E Parkins ^1^
*^1^* *University of Ghana, Accra, Ghana*
**Background:** Traumatic tongue amputation is an uncommon but devastating injury that may result from assault, accidents or self-inflicted trauma. Human bite injuries to the tongue are particularly rare and present unique reconstructive, functional and psychological challenges. Prompt assessment and appropriate management are essential to optimize speech, swallowing, and aesthetic outcomes.
**Case Presentation:** A 26-year-old patient presented to the emergency department at Korle Bu Teaching Hospital three hours after sustaining a human bite injury during an assault, resulting in traumatic amputation of the anterior one-third of the tongue. The amputated segment was retrieved and brought to the hospital with the patient. Initial assessment revealed that the avulsed tongue segment was non-viable and unsuitable for replantation. The patient was hemodynamically stable, and no other significant injuries were identified.
**Management and Outcome:** Following thorough evaluation, a decision was made to reconstruct the remaining posterior two-thirds of the tongue to restore form and function. Surgical reconstruction was successfully performed. As part of the initial management, tetanus prophylaxis was administered. Screening for Human Immunodeficiency Virus (HIV) and Hepatitis B infection was conducted and Hepatitis B vaccination was initiated. Postoperative recovery was uneventful, with satisfactory wound healing observed during follow-up visits. The patient remains clinically stable and is currently undergoing speech and psychological therapy to facilitate functional rehabilitation and psychosocial recovery. Ongoing outpatient follow-up has been planned.
**Conclusion:** Human bite-induced tongue amputation is a rare surgical emergency requiring multidisciplinary management. When the amputated tissue is non-viable, reconstruction of the remaining tongue can provide satisfactory healing and functional outcomes. Early institution of infection prophylaxis, psychological support, speech rehabilitation and close follow-up are essential components of comprehensive patient care.

**FA26/35—Technology**
**Evaluating a Novel 3D Photoacoustic Tomography (PAT) for the Pre-Operative Mapping of Anterolateral Thigh (ALT) Flap Perforators**
Ali Alabdullah ^1^, Muholan Kanapathy ^1^, Ahmed Yassin ^1^, Nam Huynh ^1^, Paul Beard ^1^ and Afshin Mosahebi ^1^
*^1^* *University College London, London, UK*
**Objectives**: This study aimed to evaluate the accuracy and practicality of a novel photoacoustic tomography (PAT) device for pre-operative mapping of anterolateral thigh (ALT) perforator arteries. Because anatomical variability makes perforator identification challenging, PAT was compared with three established imaging modalities—hand-held Doppler (HHD), colour duplex ultrasound (CDU), and infrared thermography (IRT)—to assess its performance in both accuracy and practical usability.
**Materials and Methods**: Five healthy volunteers (ten thighs) were examined. Practicality was assessed through subjective evaluation of device portability, size, scanning time, and cost. Accuracy was evaluated by comparing PAT-identified perforator sites with those detected by HHD, CDU, and IRT. PAT images were also subjectively compared with CDU for perforator count, vessel diameter, scanning depth, and image quality (2D vs. 3D). These comparisons provided an integrated assessment of PAT’s technical performance and real-world usability.
**Results**: PAT demonstrated superior accuracy, detecting a greater number of perforators and visualizing smaller vessels with high-resolution 3D imaging. It showed moderate concordance with IRT in perforator site localisation (Kappa = 0.503, *p* < 0.001). However, PAT scored lower in practicality due to its larger size, slower scanning time, and higher cost relative to HHD, CDU, and IRT.
**Conclusion**: While PAT offers enhanced accuracy and advanced 3D vascular imaging, its practical limitations may restrict routine clinical implementation. Nonetheless, its ability to identify more and smaller perforators highlights its potential as a valuable tool for precise pre-operative mapping of ALT perforators.

**FA26/406—Technology**
**Artificial Intelligence for Patient-Facing Communication in Oral and Maxillofacial Surgery: A Scoping Review**
Othman Zuhir ^1,2^ and Joanna Matthan ^2^
*^1^* *Newcastle University Medicine Malaysia, Iskandar Puteri, Malaysia*
*^2^* *Newcastle University, Newcastle upon Tyne, UK*
**Objectives**: To map the extent, characteristics, and main application areas of artificial intelligence for patient-facing communication in oral and maxillofacial surgery (OMFS).
**Materials and Methods**: A scoping review was conducted following PRISMA-ScR guidelines. MEDLINE, Embase, Scopus, and Web of Science were searched for English-language studies published from 2023 onwards. Eligible studies evaluated AI-based tools, including large language models and chatbots, in patient-facing OMFS or related craniofacial domains. Relevant applications included patient education, patient information, informed consent, readability improvement, postoperative follow-up, caregiver education, and triage-related communication. Data were charted descriptively and synthesised thematically.
**Results**: Of 270 records identified, 92 unique records remained after duplicate removal. Thirty-two full-text reports were assessed for eligibility, and 21 studies were included in the final synthesis. Included studies most commonly evaluated ChatGPT-family models, alongside Gemini, Claude, Copilot, and Bard, across dentoalveolar, orthognathic, cleft, craniofacial, and trauma-related contexts. The most consistent findings were seen in readability optimisation, with AI-assisted rewriting reducing educational material reading levels from approximately grade 9–11 to grade 6–8 across cleft, craniofacial, and oral surgery settings. Evidence was also relatively consistent for patient information generation and consent drafting. By contrast, performance was more limited and less consistent for direct patient–AI interaction, subspecialty postoperative counselling, caregiver support, and triage-related applications. Recurring concerns included variable factual accuracy, omission of clinically important risks, limited procedural specificity, and readability that remained unsuitable for complex OMFS treatment decisions.
**Conclusion**: Current evidence suggests that AI has meaningful potential to support low-risk informational communication in OMFS, particularly readability optimisation, patient information generation, and consent drafting. However, it does not support its use as a replacement for clinician judgement and counselling, as the literature remains heterogeneous and largely evaluator-based. Further evaluation using patient-centred outcomes will be essential as AI capabilities evolve and responsible integration into OMFS practice is considered.

**FA26/449—Technology**
**Automated Wrinkle Severity Rating Scale Grading Using Standardized Facial Photographs and Deep Learning**
Joseph Kyu-hyung Park ^1,2^, Baek-Kyu Kim ^1,2^, Nakwon Choi ^1^ and Jun Seo Yoon ^1^
*^1^* *Seoul National University Bundang Hospital, Seongnam-si, Republic of Korea*
*^2^* *Seoul National University College of Medicine, Seoul, Republic of Korea*
**Objectives**: The Wrinkle Severity Rating Scale (WSRS) is widely used for clinical assessment of nasolabial fold severity, but manual grading remains subjective and dependent on evaluator experience. This study aimed to develop and evaluate a deep learning–based workflow for automated WSRS grading from standardized facial photographs.
**Materials and Methods**: A retrospective dataset of standardized frontal and oblique facial photographs was assembled from patients undergoing aesthetic facial evaluation or treatment. Nasolabial fold severity was independently graded by plastic surgeons using the 5-point WSRS. Images were preprocessed by facial alignment, region-of-interest extraction around the nasolabial fold, resolution normalization, and side-specific labeling. A convolutional neural network–based ordinal classification model was trained to predict WSRS grade for each nasolabial fold. Model performance was assessed using accuracy, mean absolute error, weighted kappa, and agreement with expert consensus. Grad-CAM visualization was used to evaluate whether model attention corresponded to clinically relevant nasolabial fold regions.
**Results**: The automated model successfully generated side-specific WSRS predictions from clinical photographs and demonstrated substantial agreement with expert consensus grading. Most discordant predictions differed by only one WSRS grade, reflecting the ordinal nature of wrinkle severity assessment. Attention mapping showed predominant activation along the nasolabial fold, melolabial crease, and adjacent midface soft-tissue shadowing. Lower agreement was observed in images with suboptimal lighting, makeup, facial expression asymmetry, or prominent cheek ptosis.
**Conclusion**: Automated WSRS grading using standardized facial photographs may provide an objective and reproducible tool for aesthetic assessment, treatment planning, and longitudinal outcome evaluation. Further validation with multicenter datasets and prospective clinical photographs is warranted.

**2FA26/1070—Technology**
**Emerging Technologies in Orbital Trauma Management: A Case Series on Endoscopic, Exoscopic and Navigation-Assisted Surgery**
Radhika Menon ^1^, Masako Fujioka-Kobayashi ^1^, Reon Morioka ^1,2^ and Takahiro Kanno ^1^
*^1^* *Department of Oral and Maxillofacial Surgery, Shimane University Faculty of Medicine, Izumo, Shimane-6930021, Japan, Izumo, Japan*
*^2^* *Department of Pharmacology, Shimane University Faculty of Medicine, Izumo, Shimane-693-8501, Japan, Izumo, Japan*
**Objectives:** To present a case series demonstrating the application of emerging technologies, including surgical navigation, intraoperative cone beam computed tomography (CBCT), endoscopic assistance, exoscopic visualization, and virtual surgical planning (VSP), in the management of complex orbital trauma and reconstruction.
**Materials and Methods:** Three patients with complex orbital injuries underwent orbital reconstruction using advanced multimodal surgical technologies. Case 1 involved a 72-year-old female with panfacial fractures, extensive orbital floor and medial wall fractures, and traumatic optic neuropathy. Reconstruction was performed using intraoperative CBCT integrated with surgical navigation, endoscopic transnasal optic canal decompression, and VSP-assisted calvarial bone graft reconstruction. Case 2 involved a 39-year-old male with a type IV orbital defect who underwent endoscopically assisted orbital floor reconstruction using a calvarial bone graft. Case 3 involved a 50-year-old male with a type I naso-orbito-ethmoid fracture who underwent orbital floor reconstruction using a three-dimensional exoscope (ORBEYE).
**Results:** The combined use of navigation-guided surgery, intraoperative imaging, endoscopic assistance, exoscopic visualization, and VSP facilitated accurate identification of anatomical landmarks, improved access to complex orbital regions, and enabled precise reconstruction of orbital defects. Intraoperative imaging allowed verification of fracture reduction and implant positioning, while enhanced visualization aided surgical precision and ergonomics. Successful orbital reconstruction was achieved in all cases without major intraoperative complications.
**Conclusion:** Emerging technologies such as surgical endoscopic navigation, intraoperative CBCT, exoscopic visualization, and virtual surgical planning can enhance the precision, safety, and effectiveness of orbital reconstruction in complex maxillofacial trauma. These technologies may improve intraoperative decision-making and facilitate accurate restoration of orbital anatomy, supporting their growing role in contemporary orbital trauma management.
Conflict of interest disclosure
Receipt of grants/research supports: AOCMF Start-up Grant 2025

**2FA26/1138—Technology**
**PEEK PSI Use in Low-Income Country: A Case of Giant Maxillary Ossifying Fibroma**
Jaime Marcelo Nkomy Vargas ^1^, Jacques Gérard Edouma Bohimbo ^2^ and Christian Djieukam Monkam ^3^
*^1^* *Clínica Nuestra Señora de Los Remedios, Brain Project Africa, Cali, Colombia*
*^2^* *Hospital Central, Brain Project Africa, FMSB Yaoundé, Yaoundé, Cameroon*
*^3^* *Hôpital Régional de Maroua, Centre Hospitalier Régional de Maroua, Brain Project Africa, Maroua, Cameroon*
**Objectives**: To raise up the use of PEEK PSI (Patient Specific Implant) for immediate reconstruction of the maxillary after maxillary tumour resection in low-income countries.
**Materials and Methods**: An 18-year female with no previous health condition presenting a giant right maxillary tumour from 2 years ago, she had failed surgical attempt of removal by neurosurgeon 18 months ago. She was referred to maxillofacial surgery where incisional biopsy ruled out malignancies. The current available evidence showed that for some head and neck tumours, PSI could be used, with advantages compared to free flaps like excellent biomechanics outcomes in long-term, less donor and recipient sites morbidity, short hospital length, reduced healthcare cost, no need of 2 teams of maxillofacial surgeons simultaneously. The choice of PEEK instead of titanium was made on biomechanics similarity, lower cost and available evidence. The digital workflow included imaging (facial TC realized in low-income country and DICOM obtention), the virtual surgical planning (VSP) using a common software for craniomaxillofacial was done by biomedical engineers in middle-income country, the same team continued with the manufacturing of PEEK PSI, the surgery was made in the low-income country where the patient lived and included a maxillofacial surgeons of both countries.
**Results**: After 12 h surgery a giant tumour of almost 10 kg was removed, and the PEEK PSI was installed. The patient suffered cardiac arrest twice during the surgery and passed away in ICU a couple of hours later. The IHC (immunohistochemistry) test showed an ossifying fibroma.
**Conclusion**: The lack of trained team in head and neck microvascular reconstruction in many low-income countries decrease possibilities of treatment. For low-income countries PEEK PSI are a good option of treatment for maxillary according to the best available evidence, but to be chosen according to specific case.
Conflict of interest disclosure:
Receipt of grants/research supports: PEEK PSI was donate by Representaciones Hospitalarias FEYA to Brain Project Africa for a patient operated in Hôpital central de Yaoundé.

**2FA26/1211—Technology**
**Orthognathic Surgery Made Easy with Virtual Surgical Planning and 3D Printing: Our Method**
Yuen Wern Tham ^1^, Jeremy Ju Kuan Lee ^1^, Steve Maisi ^2^ and Rubinderan Muthusamy ^3^
*^1^* *Department of Oral and Maxillofacial Surgery, Hospital Duchess of Kent, Sandakan, Malaysia*
*^2^* *Department of Oral and Maxillofacial Surgery, Hospital Lahad Datu, Lahad Datu, Malaysia*
*^3^* *Department of Oral and Maxillofacial Surgery, Hospital Queen Elizabeth II, Kota Kinabalu, Malaysia*
**Objectives**: Virtual surgical planning and three-dimensional printing have improved the accuracy and predictability of orthognathic surgery. However, commercial computer planning systems remain costly and may not be accessible to all patients. This study evaluates the clinical applicability of a low-cost orthognathic surgery workflow based on open-source virtual surgical planning software and in-house 3D printing. We also present our workflow for computer-assisted surgical planning, guide fabrication, and implementation of 3D printing technology in orthognathic surgery.
**Materials and Methods**: A retrospective review was conducted on 14 patients who underwent orthognathic surgery between 2022 and 2025. Virtual surgical planning was performed using open-source software, followed by in-house fabrication of cutting guides, surgical wafers, and anatomical models using 3D printing technology. Clinical outcomes, operative efficiency, complications, and postoperative accuracy were assessed. Accuracy analysis was performed in two patients by superimposing planned and postoperative three-dimensional images using colour mapping.
**Results**: 14 patients underwent successful orthognathic surgery using the described workflow. Procedures included orthognathic and craniofacial skeletal corrective surgeries, including maxillomandibular advancement for obstructive sleep apnea, and Le Fort III midface advancement. No major complications or significant relapse were observed during follow-up. Postoperative accuracy analysis demonstrated deviations largely within clinically acceptable limits. The total cost of planning, printing, and plating systems ranged from USD1,200 to USD1,880 per patient, substantially lower than reported costs for commercial computer-planned workflows. In-house manufacturing enabled rapid production of patient-specific guides and models while maintaining satisfactory clinical and functional outcomes.
**Conclusion**: The combination of open-source computer planning and in-house 3D printing provides an accurate, affordable, and practical alternative to commercial orthognathic planning systems. This workflow expands access to advanced surgical planning technologies in resource-constrained settings while maintaining favorable clinical outcomes.

**2FA26/1223—Technology**
**Innovative Ramus Distractor for Condylar Trauma**
Dr. Sureshkumar Palani ^1^, Senthilkumar Palanichamy ^2^, Anandapadmanaban S ^3^ and Venkadasalapathi Narayanasamy ^4^
*^1^* *Vignesh Dental Hospital, Maeoris Dental Implants and Esthetic Care center, Hannah Joseph Hospital, Chennai, India*
*^2^* *Govt. Rajaji Medical College, Maeoris Dental Implants and Esthetic Care Center, Hannah Joseph Hospital, Dindigul, India*
*^3^* *Govt. Theni Medical College and Hospital, Maeoris Dental Implants and Esthetic Care Center, KGS Scan Center, Theni, India*
*^4^* *Hannah Joseph Hospital, KGS Scan Center, Maeoris Dental Implants and Esthetic Care Center, Madurai, India*
**INNOVATIVE RAMUS DISTRACTOR FOR CONDYLAR TRAUMA**
**Objectives:** To evaluate the efficiency of a custom designed Ramus distractor for reducing fracture segments in Condylar trauma.
**Materials and Methods:** A prospective study was conducted on Condylar trauma cases treated between 2018 to 2025. All types of condylar trauma were included. The specially designed indigenous ramus distractor was employed to enhance control of the fragments during open reduction and internal fixation(ORIF). Preoperative Imaging, Intraoperative assessment and Post operative imaging were recorded to evaluate the alignment precision and stability of the fixation.
The ease of usage, efficiency in reduction and shorter operating time were assessed and compared with the conventional techniques.
**Results:** The custom designed instrument provided excellent three dimensional control during the fracture segment manipulation and resulted in better fragment approximation.
The surgeons reported positive feedback about the tactile sensation while using this instrument. Postoperative images and clinical function showed encouraging results.
None of the cases required a re entry due to malunion or non union.
**Conclusion:** Our Custom made Ramus Distractor improved the alignment precision and made the reduction process easier and operator-friendly, reducing the operating time. It can be used in a variety of Condlyar fractures. These advantages support this instrument to be incorporated in the armamentarium.

**2FA26/1267—Technology**
**Advances in Virtual Planning and Surgical Navigation in Orbital Pathology: A Single-Centre Experience**
Alicia Dean ^1^, Inmaculada Centella ^1^, Orlando Estevez ^1^, Ana Fortis ^1^, Francisco Jesús Alamillos ^1^
*^1^* *Oral and Maxillofacial Surgery Department, University Hospital Reina Sofía, Córdoba, Spain*
**Objectives**: To present a single-centre experience with virtual surgical planning and intraoperative navigation across the spectrum of orbital pathology, and to assess accuracy, navigation-related complications and planning and operative times.
**Materials and Methods**: Retrospective review of a prospectively maintained database (January 2012–April 2026) of 157 patients: 99 trauma, 51 tumours (benign and malignant), 5 endocrine exophthalmos and 2 silent sinus syndrome. Tools comprised 3D virtual planning in the Elements^®^ navigation software, standard and custom CAD-CAM designs in selected cases, intraoperative navigation, and image-fusion verification when indicated. Assessed variables included placement accuracy of implants/meshes, resection margins, navigation-related complications, virtual-planning time, intraoperative navigation time, and functional and aesthetic outcomes.
**Results**: In orbital fractures, virtual planning defined the defect and mirrored the healthy orbit, allowing placement of meshes and custom anatomical implants with a mean deviation <1.2 mm. In thyroid orbitopathy, planned decompression reduced exophthalmos by a mean of 5 mm while preserving key structures and reducing diplopia. In tumours, navigation guided resection with margins >6 mm and assisted reconstructive planning. In silent sinus syndrome, correct globe repositioning and symmetry were achieved. Mean virtual-planning time for an orbital fracture is currently 15 min, and mean intraoperative navigation time 20 min. The most frequent navigation-related complication was stripping or loosening of the skull post fixation screw. The team rated navigation as moderately difficult initially, easing with experience.
**Conclusion**: In this experience, virtual planning and navigation proved feasible, safe and accurate across a wide range of orbital pathology, supporting millimetric implant placement, adequate resection margins and predictable orbital repositioning, with planning times that decreased as experience grew. They have become an integral tool in orbital surgery; comparative studies are needed to confirm their benefit over conventional techniques.
Conflict of interest disclosure:
Participation in a company sponsored speaker’s bureau: In 2021

**2FA26/1268—Technology**
**Transoral Robotic Surgery in Maxillofacial Practice: A Retrospective Case Series and Complication Profile**
Nicolò Olimbo ^1^, Giulio Pagnani ^1^, Andrea Battisti ^1^, Marco Della Monaca ^1^, Danilo Di Giorgio ^1^ and Valentino Valentini ^1^
*^1^* *Department of Oral and Maxillofacial Sciences, Sapienza University of Rome, Unit of Maxillofacial Surgery, Azienda Ospedaliero-Universitaria Policlinico Umberto I, Rome, Italy*
**Objectives**: Transoral robotic surgery is widely adopted for selected head and neck diseases. This study reports a maxillofacial surgery experience and evaluates perioperative complications and oncologic outcomes. Secondary aims were to describe surgical targets, histopathology, adjuvant management, and outcomes after occult primary work-up.
**Materials and Methods**: A retrospective review was performed of consecutive adults undergoing TORS for H&N conditions over a defined study period. Demographics, surgical targets, histology, operative time, intraoperative tracheostomy, length of stay, complications, adjuvant therapies, and follow-up outcomes were collected. Occult primary cases were classified using nodal cytology/histology; primary detection rate and completeness of excision were recorded. Associations between complications and treatment extent were tested with Fisher’s exact test. Survival outcomes were estimated using Kaplan–Meier analysis.
**Results**: Fifty-five patients underwent 59 procedures, with mean age 61.0 ± 16.2 years, mean length of stay 5.21 ± 2.28 days, mean operative time 102.6 ± 71.9 min, and intraoperative tracheostomy in 4/59 procedures (7.0%). The most frequent surgical target was base of tongue (29.1%). Malignancy was present in 30/55 patients (54.5%), predominantly squamous cell carcinoma. Immediate complications included acute dysphagia (7.2%) and wound dehiscence (7.2%); persistent dysphagia occurred in 1.8%. Complication risk was 23.8% after isolated procedures versus 7.7% after combined procedures (*p* = 0.266). Occult primary work-up accounted for 10/55 patients (18.2%): the primary was identified in 6/10 (60.0%), with complete excision in 3/10 (30.0%). Recurrence occurred in 6/55 patients (10.9%) at a mean of 15.0 months (range 6–33); five-year recurrence-free survival was 0.810 (95% CI 0.510–0.936) and five-year overall survival was 0.886 (95% CI 0.677–0.963).
**Conclusion**: TORS can be performed safely and efficiently within an oral and maxillofacial surgery setting, with complication rates and oncologic outcomes consistent with current prevailing standards. These findings support incorporating TORS into the clinical practice and training pathways of maxillofacial surgeons involved in head and neck oncology.

**FA26/59—TMJ**
**Temporomandibular Joint Replacement Compared to Total Hip Arthroplasty by Patient-Specific Implant in the Growing Patient**
Ofir Librush ^1^, Evyatar Yefet ^1^, Navot Givol ^1,2^, Michael Pesis ^1,2^
*^1^* *Soroka University Medical Center, Beer Sheva, Israel*
*^2^* *Faculty of Health Sciences, Ben Gurion University, Beer Sheva, Israel*
**Objectives**: Temporomandibular joint (TMJ) total joint replacement (TJR) with alloplastic patient-specific implants (PSI) is increasingly used as a salvage option for severe end-stage TMJ disease in growing patients, although supporting evidence is limited. In contrast, pediatric and adolescent total hip arthroplasty (THA) is supported by larger cohorts and longer follow-up, offering a useful benchmark for implant-related risks in skeletally immature patients. This report evaluates the effectiveness and safety of TMJ TJR with PSI in growing patients and contextualizes these findings against contemporary pediatric and adolescent THA outcomes.
**Materials and Methods**: A systematic search of Medline, Embase, and Web of Science (1946–2025) identified studies on TMJ TJR with PSI and pediatric/adolescent THA in patients ≤18 years, reporting implant details and ≥12-month outcomes, following PRISMA 2020 guidelines.
**Results**: For TMJ TJR, 442 records were identified; after de-duplication, 312 were screened, and 30 underwent full-text review, resulting in 12 included studies encompassing 32 growing patients. For THA, 1325 records were identified; 650 unique studies were screened, and 41 underwent full-text review (eight not retrieved), yielding 12 included studies comprising 318 patients and 364 procedures. TMJ indications were primarily ankylosis, juvenile idiopathic arthritis–related end-stage degeneration, condylar resorption, and complex craniofacial pathology. TMJ TJR consistently resulted in functional improvement, with infrequent complications and failures, predominantly related to infection. In pediatric/adolescent THA, validated outcome scores improved markedly, mid-term implant survivorship was high, and late failures were mainly due to aseptic loosening, while deep infection and instability were uncommon.
**Conclusion**: Current evidence indicates favorable short- to mid-term functional outcomes of TMJ TJR with PSI in growing patients, broadly paralleling trends seen in pediatric/adolescent THA. However, limited and heterogeneous data constrain conclusions regarding long-term durability, growth-related effects, and rare complications, underscoring the need for prospective, multicenter studies with standardized outcomes and longer follow-up.

**FA26/271—TMJ**
**Experience in Managing a Case of Congenital Bilateral Temporomandibular Joint Ankylosis: A Case Report**
Miguel Carlo Custodio ^1^, Jehan Grace Maglaya ^1^, Enrico Donato ^2^, Karen Mae Ty ^1^, Deanna Guillen ^3^ and May Christine Obana ^4^
*^1^* *University of the East Ramon Magsaysay Medical Center, Quezon City, Philippines*
*^2^* *East Avenue Medical Center, Quezon City, Philippines*
*^3^* *Cebu Doctors University, Cebu City, Philippines*
*^4^* *Philippine Children Medical Center, Quezon City, Philippines*
**Objectives**: The aim of the study is to present the surgical and post-surgical management of a rare case of congenital bilateral temporomandibular joint ankylosis in a pediatric patient.
**Materials and Methods**: We present the case of a 15-year-old male diagnosed with congenital bilateral TMJ ankylosis at 2 years of age, who presented with micrognathia and trismus with a maximal incisal opening (MIO) of 0.5 cm. CT scan revealed partial fusion of the anterior and posterior aspects of the mandibular condyle and temporomandibular joint with evidence of sclerotic changs of the bilateral mandibular condyle. Initially, the patient underwent interpositional gap arthroplasty using temporalis myofascial flap. After the procedure, the patient underwent post-operative physiotherapy and dental rehabilitation. However, there was re-development of ankylosis of the bilateral temporomandibular joint after a few months from the initial treatment.
**Results**: To manage the re-ankylosis, a revision gap arthroplasty with coronoidectomy and distraction osteogenesis of the bilateral rami was performed on the patient. During the post-operative period, the patient underwent temporomandibular joint and muscle rehabilitation, in conjunction with the patient’s post-surgical follow-up, which led to an improvement of the maximal incisal opening (MIO) to 2.3 cms.
**Conclusion**: A holistic approach is essential in managing patients with congenital temporomandibular joint ankylosis. The implications of both surgical intervention as well as post-surgical rehabilitation and evaluation are crucial in achieving the long-term goals for optimal nutrition, growth and general well-being especially for pediatric patients.

**FA26/314—TMJ**
**A Method For Treatment Of Temporomandibular Joint Dysfunction In Patients With Dentofacial Anomalies**
Kseniia Piksaikina ^1^
*^1^* *North-Eastern Federal University named after Maxim Kirovich Ammosov, Yakutsk, Russian Federation*
**Objectives**: To improve the effectiveness of treatment of temporomandibular joint dysfunction in patients with dentoalveolar anomalies by using the developed method of therapy.
**Materials and Methods**: The study was conducted using the developed treatment method (patent application No. 2024134335 dated 18 November 2024) in 119 patients aged 18–49 years. The studies were conducted at the Clinic of the M.K. Ammosov North-Eastern Federal University and the Dentex Dental Clinic (Yakutsk). Initially, based on MRI results, a custom-made plastic splint made of EV material (Gasket, USA) was fabricated for the mandible. The working part of the device was represented by occlusal pads in the lateral sections with a supporting and retaining beam on the vestibular side. Then, TENS therapy was performed with deprogramming of the maxillofacial muscles with additional electrodes for the shoulder girdle and neck muscles using a J5 myomonitor (Myotronics, USA).
**Results**: Overall, the application of the developed method confirms the clinical efficacy of treating TMJ dysfunction in patients with dentoalveolar anomalies associated with relaxation of the maxillofacial muscles, as well as the shoulders and neck. This leads to the elimination or reduction of myofascial pain syndrome by reducing intra-articular disc compression and restoring mandibular biomechanics. Furthermore, the obtained results indicate restoration of mandibular biomechanics. The method is characterized by its simplicity and ease of use in practice, regardless of the specialist’s professional experience. The next stage is orthodontic treatment of dentoalveolar anomalies and subsequent orthopedic rehabilitation.
**Conclusion**: The conducted analysis and evaluation of the application of the developed treatment method characterize the clinical effectiveness, which determines the prospects of its application in clinical dentistry, in particular, in the treatment of myofascial pain syndrome in TMJ dysfunction in patients with dentoalveolar anomalies, which to a certain extent determines the prevention of further complications of dentoalveolar anomalies and joint diseases.

**FA26/317—TMJ**
**Functional Outcomes of Suprafoveal Condylar Arthroplasty in Wilkes Stage III Temporomandibular Joint Internal Derangement**
Arunkumar Shadamarshan R ^1^
*^1^* *Vajra Corps Dental Unit, Jalandhar, India*
**Objectives**: To assess whether suprafoveal condylar arthroplasty improves joint function in patients with unilateral Wilkes stage III temporomandibular joint internal derangement, corresponding to disc displacement without reduction.
**Materials and Methods**: This retrospective, single-institution, non-blinded case series included patients who underwent unilateral suprafoveal condylar arthroplasty for clinically and radiologically confirmed Wilkes stage III temporomandibular joint internal derangement from 2008 to 2020. The primary outcome was pain assessed by Visual Analog Scale. Secondary outcomes included interincisal mouth opening, deviation on mandibular opening, laterotrusive and protrusive movements, occlusal changes, and perioperative complications.
**Results**: 23 patients (10 Males, 13 females) were included in the study with a mean age of 35.09 years and mean follow up period of 37.7 months (Range: 12–58 months; SD = 15.3). Paired *t*-test revealed statistically significant(*p* = 0.000) reduction in the mean pain scores on Visual Analog Scale(VAS) from 6.35 preoperatively to 1.13 postoperatively. The improvement of the mean pre-operative mouth opening of 21.83 mm to a mean post-operative mouth opening of 42.09 mm was also found to be statistically significant (*p* = 0.00). McNemar’s test revealed statistical significant subjective improvement after one year in protrusive (*p* = 0.0003), laterotrusive (*p* = 0.0005) and opening movements (0.0001). No patient developed any changes in occlusion post-operatively. 4/23(17.3%) patients developed transient neuromotor deficit of the temporal branch of the facial nerve.
**Conclusion**: Suprafoveal condylar arthroplasty without disc management is an effective open surgical option for unilateral Wilkes stage III temporomandibular joint internal derangement.

**FA26/321—TMJ**
**Novel Hypotheses on the Temporomandibular Joint Derived from Ankylosing Spondylitis: Insights into Disc Anatomy, Enthesis, and Evolution**
Arunkumar Shadamarshan R ^1^
*^1^* *Vajra Corps Dental Unit, Jalandhar, India*
**Objectives**: Ankylosing spondylitis (AS) is a seronegative arthropathy that primarily affects fibrocartilaginous joints, often leading to arthritis and ankylosis; however, the temporomandibular joint (TMJ) is only rarely involved, and TMJ ankylosis in this condition remains poorly explained in the literature. This paper proposes several integrative hypotheses to explain the relative resistance of the TMJ to ankylosis in AS and to explore the anatomical and functional role of the articular disc.
**Materials and Methods**: This study is a conceptual, literature-based analysis synthesizing published data on AS-related joint pathology, TMJ anatomy, entheseal structures, and comparative evidence from extant monotremes. Clinical observations of TMJ ankylosis in patients with AS are reviewed alongside established concepts of functional enthesis and the enthesis organ, applied to the TMJ disc and its associated ligaments.
**Results**: Several working hypotheses are proposed. First, pathological condylar loading may predispose a subset of AS patients to TMJ ankylosis. Second, the TMJ articular disc may function as a functional enthesis, linking the lateral pterygoid muscle to the mandibular condyle and the temporal bone via elastic-fiber-rich transitional tissue. Third, the disc may be phylogenetically and functionally analogous to an involuted tendon, with the discomalleolar ligament extending to the malleus. Fibrocartilaginous changes on the disc undersurface may support this entheseal concept, while evidence from monotremes may provide insights into the embryological development of the disc. The available data remain largely anecdotal, and further experimental, imaging, and histological studies are required for validation.
**Conclusion**: These hypotheses may refine the understanding of TMJ anatomy, the functional significance of the articular disc, and its role in TMJ disorders, including those associated with ankylosing spondylitis. If validated, these concepts could influence current treatment paradigms involving discectomy and the emerging field of TMJ tissue engineering aimed at reconstructing or replacing the articular disc.

**FA26/349—TMJ**
**Synovial Chondromatosis: Case Report and Review of the Literature**
Ville Lehtonen ^1^, Riitta Lithovius ^2^, Jennina Kangasniemi ^1^, Timo Myllärinen ^3^ and György Sandor ^4^
*^1^* *Research Unit of Population Health, Faculty of Medicine, University of Oulu, Finland, Oulu, Finland*
*^2^* *Adjunct Professor, Research Unit of Population Health, Faculty of Medicine, Oulu University, Oral and Maxillofacial Surgeon, Oulu University Hospital, Finland, Medical Research, Oulu, Finland*
*^3^* *Oral and Maxillofacial Surgeon, Oulu University Hospital, Oulu, Finland*
*^4^* *Emeritus professor Research Unit of Population Health, Faculty of Medicine, University of Oulu, Finland, Oral and Maxillofacial Surgeon, Plastic Surgeon, Oulu University Hospital, Medical Research Center, Oulu, Finland*
**Objectives**: Synovial chondromatosis (SC) of the temporomandibular joint (TMJ) is a rare, progressive condition in which synovial metaplasia produces cartilaginous nodules. Only 3% of all SC cases involve the TMJ. Due to nonspecific symptoms, diagnosis is frequently delayed. This study aimed to systematically review all reported English-language cases of TMJ-SC in humans and to report a patient case.
**Materials and Methods:** A systematic literature search was conducted in PubMed, Scopus, and Medline databases up to April 2021. Inclusion criteria required histopathologically confirmed diagnosis, reported imaging, documented treatment, and a minimum 12-month postoperative follow-up. After removing duplicates and applying inclusion criteria, 44 publications comprising 103 cases were identified. A new case from our institution was added, yielding 104 cases in total. Patient demographics, imaging modalities, surgical treatment, synovectomy, follow-up duration, and recurrence were tabulated and analyzed.
**Results**: The mean patient age was 45.6 years. Females represented 70.2% of cases. The right TMJ was affected in 51.9% of cases, and the upper joint compartment was predominantly involved. Intracranial extension was observed in 17% of cases. MRI was the most sensitive imaging modality, while CT was valuable when nodules were calcified. Arthrotomy was the definitive treatment in 77.9% of cases, frequently combined with synovectomy. With a mean follow-up of 3.8 years, recurrence occurred in only 2.9% of cases. Our patient, a 55-year-old male, presented with left TMJ pain and over 250 calcified nodules confirmed on imaging. Arthrotomy with removal of all loose bodies from the upper joint space was performed with no intraoperative complications.
**Conclusion**: TMJ-SC is rare and diagnostically challenging. MRI is the preferred imaging modality for early detection. Arthrotomy with synovectomy remains the most common and effective treatment, with low recurrence rates. The role of synovectomy in preventing recurrence warrants further investigation, as does its necessity in advanced Milgram stage disease.

**FA26/386—TMJ**
**Osteochondroma of the Mandibular Condyle Treated with Navigation-Guided Low Condylectomy: A Case Report**
Nithit Kitsoponphisan ^1^
*^1^* *Mahidol University Faculty of Dentistry, Bangkok, Thailand*
**Objectives**: To report a case of a large mandibular condylar osteochondroma treated with a navigation-guided surgical approach. The treatment aimed to safely excise the tumor and restore functional occlusion and facial harmony without the need for additional orthognathic surgery.
**Materials and Methods**: A 48-year-old healthy female presented with left temporomandibular joint (TMJ) pain during mastication, severe malocclusion, and noticeable facial asymmetry. 3D computed tomography (MDCT) revealed a 31.9 × 16.6 mm well-defined, lobulated mass at the left mandibular condyle, causing a significant contralateral mandibular shift. Under general anesthesia and computer-assisted surgical navigation, a left low condylectomy was performed via a preauricular approach to completely excise the tumor. An autologous abdominal fat graft was interposed into the left TMJ space to prevent postoperative ankylosis. Associated third molars (18, 38, 48) were extracted, and bone-borne arch bars were utilized for stable fixation and postoperative
**Results**: Surgical navigation provided high precision, allowing for the complete excision of the large condylar mass while preserving the facial nerve and adjacent vital structures. Following the procedure and subsequent postoperative elastic therapy, the patient achieved gradual improvement in facial symmetry and progressive restoration of stable functional occlusion. Postoperative histopathological examination definitively confirmed the diagnosis of benign osteochondroma.
**Conclusion**: Managing large condylar osteochondromas is complex. A navigation-guided low condylectomy combined with autologous fat graft interpositioning serves as a safe and effective treatment. In this case, the approach successfully eradicated the tumor, leading to a gradual but significant restoration of facial harmony and occlusal function through postoperative management, demonstrating that a focused condylectomy can achieve excellent aesthetic and functional outcomes

**FA26/403—TMJ**
**Left Condylar Osteochondroma Treated with Condylectomy and Intraoperative Nerve Stimulation: A Multidisciplinary Approach**
Benya Sutthiwiwat ^1^
*^1^* *Mahidol university, Bangkok, Thailand*
**Objectives**: To evaluate the management of mandibular osteochondroma of the left condyle, specifically focusing on the integration of intraoperative nerve stimulation for facial nerve preservation and the necessity of staged multidisciplinary rehabilitation.
**Materials and Methods**: A 44-year-old male presented with a 3-year history of progressive right mandibular deviation. Clinical findings included a 10 mm mandibular midline shift, anterior and right posterior crossbite, and a maximum mouth opening (MMO) of 3.5 cm with deflection to the left. MDCT imaging confirmed a well-defined exophytic mass at the medial aspect of the left condyle, which resulted in an antero-inferior condylar dislocation. The patient underwent a left condylectomy via a preauricular approach. Given the lesion’s medial proximity, intraoperative nerve stimulation was employed to provide real-time functional mapping and protection of the facial nerve branches during the deep dissection.
**Results**: Histopathology confirmed the diagnosis of osteochondroma. While intraoperative stimulation verified nerve continuity, the patient experienced transient postoperative facial weakness (House-Brackmann Grade V). This neuropraxia was managed conservatively, with function improving to Grade II during the follow-up period. Postoperatively, MMO reached approximately 3 finger breadths, and facial symmetry was restored. However, an incomplete tooth contact was noted on the left (surgical) side. Orthodontic evaluation advised a period of observation rather than immediate intervention, citing concerns over potential skeletal relapse and the current inability to definitively correct the occlusal discrepancy during the early healing phase.
**Conclusion**: Condylar osteochondroma presents significant surgical risks to the facial nerve due to its anatomical location. Intraoperative nerve stimulation serves as a critical safeguard, providing the surgical team with confidence in nerve integrity despite temporary postoperative deficits. Achieving a successful outcome relies on a cautious, staged multidisciplinary strategy, allowing for a period of clinical observation to ensure skeletal stability before committing to definitive orthodontic treatment.

**FA26/430—TMJ**
**“Beyond the Joint: Reimagining TMJ Surgery Through Artificial Intelligence”**
Hitesh Bansal ^1^ and Hannah Arnantha ^2^
*^1^* *London North West University Healthcare NHS Trust, London, UK*
*^2^* *St George’s, Epsom and St Helier Hospital Group, London, UK*
**Objectives**: To review the current and emerging applications of artificial intelligence (AI) in temporomandibular joint (TMJ) surgery, with a focus on its role in diagnosis, surgical planning, intraoperative guidance, and outcome prediction.
**Materials and Methods**: A narrative literature review was conducted based on contemporary evidence surrounding technological advancements in TMJ surgery, including imaging, navigation systems, augmented reality (AR), and AI-driven tools. Emphasis was placed on studies evaluating machine learning in imaging interpretation, virtual surgical planning, and intraoperative assistance. Relevant developments in arthroscopy, total joint replacement, and digital workflows were also analysed to contextualise AI integration within modern TMJ practice
**Results**: AI applications in TMJ surgery are rapidly evolving. Deep learning algorithms have demonstrated the ability to automatically classify TMJ degeneration on MRI and assist in diagnosing internal derangements. AI-enhanced imaging and planning tools enable improved accuracy in preoperative assessment and patient-specific surgical simulation. Intraoperatively, the integration of AI with AR and navigation systems facilitates real-time anatomical visualisation, instrument tracking, and precision in portal placement and prosthetic positioning, often achieving millimetre-level accuracy. Additionally, predictive analytics show promise in forecasting surgical outcomes and disease progression, supporting clinical decision-making. Despite these advances, clinical adoption remains limited by data heterogeneity, validation requirements, and integration challenges.
**Conclusion**: Artificial intelligence represents a transformative adjunct in TMJ surgery, enhancing diagnostic accuracy, surgical precision, and personalised care. While current applications are largely supportive rather than autonomous, ongoing advancements in machine learning, real-time imaging, and digital surgery are expected to further integrate AI into routine practice. Future research should focus on clinical validation, standardisation of datasets, and seamless incorporation into surgical workflows to fully realise the potential of AI in TMJ surgery.

**FA26/450—TMJ**
**Range of Motion After Gap Arthroplasty for Temporomandibular Joint Ankylosis: A Case Series**
Martie Sophia Teves ^1^ and Benedick Borbe ^1^
*^1^* *Jose B Lingad Memorial General Hospital, San Fernando, Pampanga, Philippines*
**Objectives**: In patients with temporomandibular joint ankylosis seen in a Philippine tertiary training hospital, does condylectomy with interpositional gap arthroplasty result in improved post-operative range of motion, compared with pre-operative baseline mouth opening, over a 6–12 month post-operative period?
**Materials and Methods**: A retrospective case series of five patients with either unilateral or bilateral temporomandibular joint ankylosis in a tertiary training hospital was conducted. Demographic data, etiology, duration of symptoms, maximal interincisal opening (MIO), surgical approach, and post-operative measurements were reviewed. All patients underwent condylectomy with interpositional gap arthroplasty using a temporalis myofascial flap via an Alkayat-Bramley approach.
**Results**: Five patients (mean age: 16.2 years; 3 females, 2 males) presented with severe trismus. Etiologies included post-traumatic (60%) and infective (40%) causes. Mean pre-operative MIO was 5.6 mm (range: 0–18 mm). Following surgical release, mean intra-operative MIO increased to 27.8 mm (range: 19–40 mm). Postoperative follow-up demonstrated sustained improvement, with mean MIO of 25.6 mm (range: 20–34 mm). Patients with shorter duration of ankylosis and less severe pre-operative restriction demonstrated greater functional gains. No immediate major complications or early recurrence were observed.
**Conclusion**: In our experience, condylectomy with interpositional gap arthroplasty using a temporalis myofascial flap via an Alkayat-Bramley approach provided significant and sustained improvement in post-operative mouth opening in patients with temporomandibular joint ankylosis. Earlier intervention appears to yield superior range of motion, highlighting the importance of timely diagnosis and management.

**2FA26/1110—TMJ**
**Condylo-Zygomatic Fusion and Contralateral Sawhney Type III TMJ Ankylosis: A Case Report of Complex Bilateral Reconstruction TMJ**
Anak Agung Istri Ayu Detritha Sarasmartha Putri ^1^, Agus Roy Rusly Hariantana Hamid ^2^ and I Gusti Putu Hendra Sanjaya ^3^
*^1^* *Resident of Plastic, Reconstructive, and Aesthetic Surgery Residency Program, Faculty of Medicine, Udayana University, Denpasar, Indonesia, Denpasar, Indonesia*
*^2^* *Division of Plastic, Reconstructive, and Aesthetic Surgery, Ngoerah Hospital, Bali, Indonesia, Coordinator of Plastic, Reconstructive, and Aesthetic Surgery Residency Program, Faculty of Medicine, Udayana University, Denpasar, Indonesia, Denpasar, Indonesia*
*^3^* *Division of Plastic, Reconstructive, and Aesthetic Surgery, Ngoerah Hospital, Denpasar, Indonesia, Staff of Plastic, Reconstructive, and Aesthetic Surgery Residency Program, Faculty of Medicine, Udayana University, Denpasar, Indonesia, Denpasar, Indonesia*
**Objectives**: To report an exceptionally rare combination of extra-articular condylo-zygomatic fusion and contralateral Sawhney type III temporomandibular joint (TMJ) ankylosis after neglected pediatric facial trauma, and to describe a single-stage surgical strategy using temporoparietal fascia flap (TPFF) interposition for complex bilateral joint release in a resource-constrained setting.
**Materials and methods:** A 23-year-old male presented with total trismus (maximum interincisal opening, MIO 0 cm), severe facial asymmetry, and “bird-face” appearance eight years after untreated childhood facial trauma. Preoperative computed tomography with three-dimensional reconstruction suggested right-sided type III TMJ ankylosis. A single-stage procedure was performed, including prophylactic tracheostomy, bilateral extended preauricular–temporal and retromandibular transparotid approaches, left condylar neck-base ostectomy, right subcondylotomy, bilateral coronoidotomies, and gap arthroplasty with bilateral TPFF interposition via a tunneled route anterior to the zygomatic arch.
**Results**: Intraoperative exploration revealed marked discordance with the initial radiologic impression: the left side demonstrated classic intra-articular Sawhney type III bony ankylosis, whereas the right side showed massive extra-articular fusion between a laterally displaced condyle and the zygomatic arch. Following bilateral release and TPFF interposition, intraoperative MIO improved from 0 cm to 3.5 cm with stable occlusion, allowing safe tracheostomy decannulation. At postoperative day 12, MIO regressed to 1.5 cm with an anterior open bite, which improved to a functional MIO of 2.5 cm at 5.5 weeks after intensive home-based physiotherapy and referral for structured medical rehabilitation.
**Conclusion**: Direct extra-articular condylo-zygomatic fusion is an exceptionally rare sequel of neglected facial trauma and may not be fully predicted by conventional imaging, underscoring the need for meticulous surgical exploration in severe craniofacial deformities. A tailored single-stage approach combining secure airway management, extended exposure, gap arthroplasty, and TPFF interposition can effectively restore jaw function, but long-term success critically depends on rigorous, sustained postoperative physiotherapy to maintain gains and minimize re-ankylosis, particularly in resource-limited settings.

**FA26/66—Trauma**
**Comparison Between Occlusal Versus Apical Wire Endings in Patients on Erich Arch Bars: A Prospective Split-Mouth Randomized Controlled Trial**
Kar Tsyeng Ng ^1^, Marzuki Omar ^2^ and Gerald Joseph ^1^
*^1^* *Department of Oral and Maxillofacial Surgery, Hospital Teluk Intan, Teluk Intan, Malaysia*
*^2^* *Oral and Maxillofacial Surgery Department, Universiti Sains Malaysia, Kubang Kerian, Malaysia*
**Introduction**: The Erich arch bar (EAB) is a stainless-steel device used for maxillomandibular immobilization. It is secured to the teeth using circumferential stainless-steel wires. Traditionally, the free ends of these circumferential wires are directed in the apical direction, despite limited evidence supporting this practice.
**Objectives**: This study aimed to determine whether changing the ending of the stainless-steel wire from apical to occlusal direction affects: (i) mucosal trauma score, (ii) plaque score, (iii) stability of the EAB, and (iv) incidence of glove perforations.
**Materials and Methods**: A split-mouth randomised controlled trial was performed on 23 patients (46 sides). Patients were recruited using convenience sampling, and randomization was performed to assign the apical and occlusal groups. Glove perforations of the inner and outer glove were assessed immediately after completion of EAB. Mucosal injury and the stability of the EAB were evaluated at the second week, while plaque scores were assessed at the fourth week.
**Results**: The occlusal group demonstrated statistically significant lower mucosal trauma (*p* < 0.005) and a lower incidence of glove perforations (*p* < 0.005) compared with the apical group. No statistically significant differences were observed between these two groups with respect to plaque accumulation or arch bar stability (*p* > 0.05).
**Conclusion**: Surgeons face the possibility of exposure to bloodborne pathogens due to perforations in surgical gloves. Our research indicated that occlusal ending significantly reduces the incidence of glove perforations and mucosal trauma. Therefore, the authors highly advise that circumferential wires be directed in an occlusal direction.

**FA26/77—Trauma**
**Endoscopic Assisted Intraoral Open Reduction and Internal Fixation (Eaiorif) of Sub Condylar Fractures—A Decade of Experience Based Clinical Outcome Study**
Venkatesh Anehosur ^1^
*^1^* *sdm Craniofacial Centre, Sdm College of Dental Sciences And Hospital, Sdm University, Dharwad, India*
**Objectives**: The surgical innovations have progressed from wider exposure to minimal invasive surgeries. Evolution of novel technique such as Endoscopically Assisted Open Reduction and Intraoral Fixation (EAORIF) of sub condylar fractures has gained significant popularity in managing these cases as they are aesthetically accepted and exhibit low comorbidity status. Also, it offers prompt recovery with comparable results while diminishing the risk of facial nerve injury and sparing facial scars. This article presents and highlights about functional and aesthetic outcomes of EAORIF of mandibular sub condylar fractures over 10 years in our tertiary care centre.
**Materials and Methods**: A single tertiary centre study was conducted on 51 patients who underwent EAORIF of mandibular sub condylar fractures from January 2015 to December 2025, all patients’ medical records were scrutinised for the demographic details, preliminary diagnosis, post operative Functional, esthetical outcomes (FO) and complications. All data collected were, tabulated and were subjected to suitable statistical analysis.
**Results**: Out of 54 patients who underwent (EAORIF), 51 patients were included, 3 were excluded for lack of data. 38 (74.5%) were male and 13 (25.5%) were female patients. The mean age group was 40.9 years (20–70 years). All patients were scrutinized post operatively for function and aesthetic outcomes up to 5-year, maximal mouth opening (interincisal range in cms), mandibular movements range deficit, facial asymmetry and neural deficit (sensory and motor). surgical site infection was seen in 1 patient (1.9%) which was managed conservatively and no other major complications were noted. No cases were converted to extraoral approach and 1 (1.9%) case was abandoned for EAORIF due to endoscopy malfunction.
**Conclusion**: EAORIF provide superior functional and aesthetic results with the advantage of early mobilisation and no neural deficit. Though with steep learning curve the endoscopic armamentarium techniques offers predictable results with excellent function.

**FA26/93—Trauma**
**Mandible First or Midface First? Practical Guidelines from Real Cases**
Fairouz Bekrentchir ^1^
*^1^* *University hospital of Oran, ORAN, Algeria*
**Objectives**: The sequencing of reconstruction in panfacial fractures remains controversial. According to craniomaxillofacial principles, reconstruction may follow bottom-up, top-down, or hybrid strategies depending on the availability of stable skeletal reference points. This study aims to compare two sequencing strategies through analysis of complex panfacial trauma cases and to highlight decision-making based on anatomical stability rather than a fixed algorithm.
**Materials and Methods**: Two patients with severe panfacial fractures treated between 2025 and 2026 were retrospectively analyzed. The first case presented with Le Fort I and II fractures, a right hemi–Le Fort III component, orbitozygomatic disjunction, orbital blow-in fracture, and comminuted mandibular fractures. A bottom-up strategy was used, prioritizing reconstruction of the mandible as a stable condyle-to-condyle reference to guide maxillofacial reconstruction.
The second case involved an orbitozygomatic complex fracture associated with bifocal mandibular fractures. A top-down strategy was selected, prioritizing restoration of the zygomatico-orbital framework as the primary determinant of facial width and projection, with delayed mandibular reconstruction after midfacial stabilization.
**Results**: In the first case, mandibular reconstruction provided a stable occlusal and vertical reference, enabling accurate restoration of midfacial buttresses and orbital alignment. In the second case, early reconstruction of the zygomatico-orbital complex restored facial width and projection, facilitating subsequent mandibular reconstruction and occlusal re-establishment. Both cases achieved stable postoperative occlusion and satisfactory facial symmetry without revision surgery.
**Conclusion**: Sequencing in panfacial fracture reconstruction should be individualized according to the most reliable anatomical reference points. A bottom-up approach is indicated when mandibular continuity provides a stable occlusal framework. A top-down strategy is preferable when the zygomatico-orbital complex defines facial projection and midfacial structure. These cases illustrate that surgical sequencing should be guided by fracture pattern and skeletal stability rather than a fixed reconstruction algorithm.

**FA26/107—Trauma**
**TRANSCONJUNCTIVAL Approach in Orbital Fractures: Experiences, Aesthetics and Complication Analysis in 30 Cases**
Dr Suresh Bommaji ^1^
*^1^* *St Theresa’s hospital, City neuro hospital, Rnc hospital, Hyderabad, India*
**Objectives**: To analyze the aesthetic efficacy, surgical experience, and complication rates of the 30 orbital fractures treated through transconjunctival approach.
**Materials and Methods**: In this case series, we present 30 cases of surgically treated orbital fractures using open reduction and internal fixation. A retro-septal transconjunctival approach was utilized for infra orbital rim and orbital floor access in all patients. The fixation of the orbital floor and infraorbital rim was accomplished utilizing an orbital floor mesh (KLS Martin system 25-045-01-09) and/or orbital rim plates. We documented intraoperative and postoperative complications, as well as postoperative healing.
**Results**: Out of 30 patients, 15 were males and 15 were females with a mean age of 38.6 ± 3.7 years. Unilateral orbital fractures were predominant, constituting 83% of cases. Orbital bone access utilized a transconjunctival incision in 43.3% (n = 13) and a combination of transconjunctival and lateral orbitotomy incisions in 56.7% (n = 17). During surgery, 10% experienced bradycardia, and postoperative complications, specifically scleral show were observed in another 10%. Healing outcomes were excellent in 36.7% and good in 53.3% of patients.
**Conclusion**: Despite the occurrence of transient and manageable intraoperative complications in selected cases, the overall success of the transconjunctival approach underscores its efficacy in the repair of orbital fractures.

**FA26/112—Trauma**
**Real-Time Endoscopic Visualization for Enhanced Nerve Protection in Mandibular Sagittal Split Osteotomy**
Venkatesh Anehosur ^1^
*^1^* *SDM Craniofacial Centre, Sdm College of Dental Sciences And Hospital, SDM University, Dharwad, India*
**Objectives**: Minimal invasive surgical procedures are popularized in various surgical fields, the utilization of endoscopic assisted techniques continues to grow across in the field of Maxillofacial Surgery. Orthognathic surgery is a commonly employed procedure for skeletal deformities. Bilateral sagittal split osteotomy(BSSO) is a versatile, result oriented procedure and its utility are well documented. The main pitfall is injury to the neurovascular bundle. Although various methods and modified instrumentation are advocated to minimize the injury to IAN. The primary objective is to study the efficacy of innovative endoscopic assisted procedure for locating the nerve and preventing its injury during the BSSO.
**Material and Methods**: This initial prospective study involving 10 patients undergoing BSSO (advancement/setback) has explored the role of endoscopic assisted to precisely identify and safeguard the nerve during osteotomy cuts, identification and releasing of IAN while splitting the proximal and distal fragments of BSSO.
**Results and Conclusion**: Integration of the endoscopy in BSSO protocols showed more than 50% reduction in injury to Neurovascular bundle and offers a promising advancement for achieving favourable results.

**FA26/115—Trauma**
**Validity of the Tongue Blade Bite Test (TBBT) as a Clinical Predictor for the Presence or Absence of Mandibular Fractures in Facial Trauma Cases in a Philippine Tertiary Hospital**
Allan Fred Camungol ^1^, Philip Fullante ^1^ and Genilou Liv Baligod ^1^
*^1^* *Philippine General Hospital, Manila, Philippines*
**Objectives**: This study evaluated the diagnostic validity of the Tongue Blade Bite Test (TBBT) as a bedside clinical predictor of mandibular fractures among facial trauma patients in a Philippine tertiary referral center. Subgroup performance according to fracture location and fracture complexity was also assessed.
**Materials and Methods:** A retrospective diagnostic accuracy study was conducted through systematic chart review of facial trauma patients managed from January 2018 to December 2024. Records were included if TBBT results and radiographic confirmation of fracture status were explicitly documented. Radiographic imaging served as the reference standard. Diagnostic performance indices—including sensitivity, specificity, positive predictive value (PPV), negative predictive value (NPV), and overall accuracy—were calculated using contingency table analysis. Stratified analyses were performed based on fracture location and fracture complexity.
**Results**: Of 907 charts screened, 99 met inclusion criteria. Fifty-eight patients (58.6%) had radiographically confirmed mandibular fractures, while 41 (41.4%) had none. The TBBT yielded 43 true positives, 39 true negatives, 2 false positives, and 15 false negatives. Overall sensitivity was 74.1%, specificity 95.1%, PPV 95.6%, NPV 72.2%, and diagnostic accuracy 82.8%. The high specificity and PPV demonstrate strong rule-in capability, indicating that a positive TBBT result was highly predictive of fracture. Although sensitivity and NPV were moderate, a negative result substantially reduced the probability of fracture. Diagnostic performance was more consistent in multi-site and higher-complexity fractures, while location-specific estimates were limited by small subgroup sizes.
**Conclusion**: The TBBT demonstrated good overall diagnostic performance, with particularly strong rule-in value. While imaging remains the diagnostic gold standard, routine incorporation of the TBBT may enhance early clinical decision-making and optimize imaging utilization in emergency and resource-limited settings.

**FA26/204—Trauma**
**Is Bioresorbable Plates Are Comparable to Conservative Management in Pediatric Maxillofacial Fracture? A Comparative Clinical Study**
Krishna Vasaiya ^1^ and Geeta Singh ^1^
*^1^* *King George’s Medical University, Lucknow, India*
**Objectives**: Pediatric maxillofacial trauma presents unique clinical challenges, as treatment must balance immediate fracture stabilization with the long-term developmental needs of a growing skeleton. While traditional metallic fixation provides stability, it carries risks of growth restriction and requires secondary removal procedures. This study evaluates the efficacy and safety of bioresorbable plating systems as a modern alternative to traditional conservative management.
**Materials and Methods**: A comparative clinical study was conducted involving 21 pediatric patients (below 16 years of age). Participants were divided into two groups: Group A received open reduction and internal fixation (ORIF) using bioresorbable plates (n = 5), and Group B underwent conservative management with Erich arch bars and maxillomandibular fixation (n = 16). Clinical parameters—including pain (VAS), swelling, occlusion, mouth opening, and mobility—were assessed over a 12-month follow-up period.
**Results**: Both treatment modalities achieved successful fracture healing with no significant complications reported in either group. However, the bioresorbable plates group (Group A) demonstrated significantly faster recovery across several key metrics:
Pain Reduction: Group A showed a more rapid decline in pain intensity, achieving near-complete relief by the 15th day compared to Group B.
Functional Recovery: Group A exhibited significantly greater improvements in mouth opening by the 1-month(34.40 mm vs. 28.00 mm in Group B).
Dietary Progression: Patients with bioresorbable plates transitioned to semi-solid and solid diets significantly faster than those in the conservative group.
Stability: Group A achieved immediate fracture stability postoperatively, whereas Group B showed a gradual reduction in fracture mobility over 15 days.
**Conclusion**: Bioresorbable plating systems offer a superior alternative to conservative management for pediatric maxillofacial fractures by facilitating functional recovery, faster pain relief, and earlier dietary advancement without the need for implant removal. These findings support the adoption of bioresorbable materials to enhance postoperative patient comfort and optimize clinical outcomes in the pediatric population.
Conflict of interest disclosure
Support: Study Guide

**FA26/221—Trauma**
**Hidden Sequelae of Frontal Sinus Fracture Repair: Late Mucocele Formation After ORIF**
Kah Yin Chor ^1^, Yee Hao Tan ^1^ and Jeremiah See ^1^
*^1^* *Department of Plastic & Reconstructive Surgery, Hospital Raja Permaisuri Bainun, Ipoh, Malaysia*
**Objectives**: Frontal sinus mucoceles are recognized delayed complications following trauma or surgery, typically resulting from obstruction of the frontonasal duct. Although open reduction and internal fixation (ORIF) with titanium mesh is a standard treatment for anterior table fractures, it does not eliminate the risk of late mucocele formation. We present a case of delayed post-traumatic frontal mucocele despite prior reconstruction, highlighting surgical management and reconstructive considerations.
**Materials and Methods**: A 31-year-old male with a history of a motor vehicle accident in 2016 sustained a frontal sinus anterior table fracture treated with ORIF and titanium mesh placement. He presented 10 years later with a 5-year history of progressive, asymptomatic forehead swelling. Examination revealed a soft, non-mobile frontal mass without signs of infection, nasal symptoms, or visual disturbance.
Surgical exploration via a coronal approach identified a 2 × 2 cm mucocele arising from the previous fracture site and adherent to the mesh. The lesion expanded through a pre-existing left orbital roof defect (3 × 1 cm), abutting but not infiltrating the orbital contents. The left frontonasal duct was obliterated.
**Results**: Complete excision of the mucocele was performed with stripping of sinus mucosa and removal of the implant. The orbital roof defect was reconstructed using an iliac bone graft. Frontal sinus obliteration was achieved with a pericranial flap, followed by anterior table reconstruction with titanium mesh. A dermofascial graft was applied over the thinned glabellar skin. Recovery was uneventful with satisfactory functional and aesthetic outcomes.
**Conclusion**: Frontal sinus mucoceles may develop years after fracture repair with mesh fixation. Persistent or delayed frontonasal duct obstruction remains the key pathogenic factor. Long-term follow-up is recommended, and timely surgical intervention is essential to prevent orbital or intracranial complications.

**FA26/247—Trauma**
**Surgical Correction of Post-Traumatic Anterior Dentoalveolar Extrusion: Achieving Occlusal Harmony by Mandibular Subapical Osteotomy and Unilateral Maxillary Osteotomy**
Gery Gaghana ^1^, I Dewa Gde Budhi Asthana ^1^, Yudy Utomo ^1^ and Wenny Yulvie ^2^
*^1^* *Universitas Indonesia, Jakarta, Indonesia*
*^2^* *Cipto Mangunkusumo Hospital, Jakarta, Indonesia*
**Objectives**: To evaluate the surgical efficacy of a mandibular subapical osteotomy in repositioning a dentoalveolar segment that underwent extrusion followed by unilateral maxillary osteotomy in case of untreated maxillofacial trauma, with the aim of restoring functional occlusion, facial symmetry, and stable dentoskeletal relationships.
**Materials and Methods**: This case analysis details a 51-year-old female presenting with post-traumatic trismus and severe occlusal discrepancy four months after motorcycle collision. Clinical and radiographic evaluation using computed tomography (CT) demonstrated malunited fractures involving the right maxilla and the anterior mandibular dentoalveolar segment 32–41 in an extruded position, resulting in open bite and function impairment for the patient. The surgical intervention prioritized a mandibular subapical osteotomy to achieve three-dimensional mobilization of the anterior segment, performed in conjunction with a unilateral right maxillary osteotomy to correct the maxillary discrepancies. Skeletal stability was obtained by Open Reduction Internal Fixation (ORIF) with L-plates and non-locking screws, while intraoperative occlusal relationships were guided by maxillomandibular fixation (MMF).
**Results**: The subapical osteotomy enabled precise repositioning of the extruded mandibular segment, successfully correcting the malocclusion and functional impairment. Postoperative examination showed normalized overjet and overbite without segmental relapse or neurovascular compromise. Healing process produced stable functional occlusion and significantly improved facial symmetry throughout the follow-up period.
**Conclusion**: In cases of maxillary and mandibular malunion characterized by anterior dentoalveolar extrusion and open bite, a mandibular subapical osteotomy followed by unilateral maxillary osteotomy serves as a predictable definitive procedure for restoring masticatory function and craniofacial harmony. This technique offers a good repositioning precision compared to conventional ORIF in the management of untreated fractures.

**FA26/313—Trauma**
**Patterns of Facial Injury in Horse-Related Trauma: A Comparative Analysis**
Apanjit Sahi ^1^, Pharibe Pope ^2^, Agata Szulia ^3^, Christopher Wen ^2^, Cailin Cruess ^1^, Ruchika Gadagkar ^1^, Judy Pan ^3^ and Natalie Justicz ^2^
*^1^* *University of Maryland School of Medicine, Baltimore, MD, USA*
*^2^* *Department of Otorhinolaryngology—Head and Neck Surgery, University of Maryland Medical System, Baltimore, MD, USA*
*^3^* *Division of Plastic and Reconstructive Surgery, R. Adams Cowley Shock Trauma Center, University of Maryland Medical Center, Department of Plastic and Reconstructive Surgery, Johns Hopkins Hospital, Baltimore, MD, USA*
**Objectives**: Facial fractures resulting from equestrian injuries represent a significant source of morbidity. This study describes fracture patterns, injury profiles, and management strategies among patients sustaining horse-related craniofacial trauma.
**Materials and Methods**: This preliminary analysis examines data from January 2018 to December 2022, comprising the first five years of an eight-year study (2018–2025). A retrospective review was conducted comparing injury patterns and characteristics among patients presenting with horse-related facial trauma. Adult patients with CT imaging of the face were included. The primary outcome was the presence of a facial fracture.
**Results**: Of 16 patients, 8 (50%) sustained facial fracture, and 4 (50%) of these patients required operative repair. Common mechanisms of injury within the facial fracture subgroup were horse kicks (n = 4 [50%]) and falls from a horse (n = 3 [38%]). Notably, 100% of patients who sustained facial fractures secondary to horse kicks required surgical intervention. While not statistically significant, a greater proportion of patients without facial fractures were mounted at the time of injury (5 [62%] vs. 3 [38%], *p* = 0.619). Among patients with facial fractures, nasal (6 [75%]) and orbital floor fractures (6 [75%]) were most common. Facial lacerations (2 [25%] vs. 5 [62%], *p* = 0.315) and spinal injury (1 [12%] vs. 3 [38%], *p* = 0.569) were more frequent in patients with facial fractures, though neither of these associations reached statistical significance. Similarly, patients with fracture had higher rates of inpatient admission (3 [38%] vs. 6 [75%], *p* = 0.315).
**Conclusion**: Equestrian trauma is associated with high incidence of facial fractures, particularly affecting the midface. No factors were significantly associated with fracture risk in this cohort. The conclusions of this preliminary analysis are limited due to low sample size. Statistical significance may be obtained upon complete analysis of patients across the 8-year period.

**FA26/339—Trauma**
**Complex Mandibular Trauma: Delayed Management of a Symphyseal Fracture and Bilateral Condylar Head Fracture with Superolateral Dislocation: A Case Report**
Kunlanut Borrisut ^1^, Natton Ng-pooresatien ^1^, Anupong Suraseraneewong ^2^
*^1^* *Hatyai Hospital, Songkhla, Thailand*
*^2^* *Banplee Hospital, Samut Prakan, Thailand*
**Objectives**: To present the surgical management and reduction technique for a delayed case of a comminuted mandibular symphysis fracture accompanied by bilateral sagittal split condylar fractures with extracapsular superolateral dislocation beyond the zygomatic arches.
**Materials and Methods**: A 25-year-old male presented with complex mandibular fractures and bilateral superolateral condylar dislocations following a motorcycle accident. Surgical intervention was delayed for nearly a month due to active pulmonary tuberculosis. Initial attempts at reduction using zygomatic bone hooks failed due to severe displacement and chronicity. A secondary operation utilized 3D-printed models to simulate the reduction. Preoperatively, an acrylic custom guide was fabricated for the lower border of the mandible to guide the mandibular contour, and reconstruction plates were pre-bent. Intraoperatively, orthopedic periarticular reduction forceps were applied alongside the acrylic guide to exert controlled pressure, successfully seating the displaced condyles and mandibular angles into proper anatomical positions before rigid fixation.
**Results**: The bilaterally dislocated condyles were successfully reduced into the glenoid fossae, and rigid internal fixation was achieved at the symphysis using the reconstruction plates. The patient was instructed to perform postoperative mouth-opening exercises. At six months, the reconstruction plates were removed. Over a 3-year follow-up period, the patient maintained a stable, proper occlusion with a maximum mouth opening of 3 cm. However, he developed bilateral condylar fibrous ankylosis as a late complication. Surgical release of the ankylotic mass was proposed to further improve jaw mobility, but the patient declined additional surgical intervention.
**Conclusion**: Utilizing 3D-printed models, acrylic custom guides, and orthopedic reduction forceps provides an effective protocol for anatomically reducing delayed, complex bilateral superolateral condylar dislocations. Although this approach successfully restores functional occlusion, the long-term risk of developing fibrous ankylosis highlights the inherent challenges and the necessity of prolonged monitoring in chronic condylar trauma

**FA26/359—Trauma**
**Development and Validation of a Novel Treatment-Oriented Classification for Zygomaticomaxillary Complex Fractures: A Retrospective Cohort Study**
Rakesh Meenachi ^1,2,3^, Balakrishnan Ramalingam ^4^, Shanmugapriyan D ^5^, Wasim Ahmed Sg ^6^, Vijay Ebenezer ^4^, Abu Dakir I ^7^, Vigil Dev Asir R ^4^ and Y Naren Kumar ^8^
*^1^* *Sree Balaji Dental Hospital, Mohans Dental Clinic and Maxillofacial Centre, Deepam Hospital, Annai Arul Hospital, Chennai, India*
*^2^* *Annai Arul Hospital, Chennai, India*
*^3^* *Deepam Hospital, Chennai, India*
*^4^* *Sree Balaji Dental Hospital, Chennai, India*
*^5^* *Hande Speciality Surgical Centre, Chennai, India*
*^6^* *Dr KSGA Nasser Dental Clinic, Chennai, India*
*^7^* *Sudar Hospitals, Chennai, India*
*^8^* *Apollo Dental Clinics, Chennai, India*
**Objectives**: Existing classifications of zygomaticomaxillary complex fractures are largely descriptive and do not consistently guide treatment decisions. therefore, a clinically relevant, treatment-oriented system that integrates anatomical, functional, and radiological parameters is needed. this study aims to develop and validate a novel treatment-based classification for zygomaticomaxillary complex fractures and evaluate its ability to guide management and predict outcomes.
**Materials and Methods**: This retrospective cohort study will be conducted using data from 100 patients with CT-confirmed zygomaticomaxillary complex fractures. Fractures will be classified using a novel classification system comprising: (1) a vertical component based on the involved buttresses (Class 1–5), with functional subclassification at 1 week (A: absence of trismus, B: presence of trismus), and (2) a horizontal subcomponent based on orbital floor involvement (Type 1–4), incorporating infraorbital nerve involvement, herniation, and enophthalmos. Treatment modality and clinical outcomes will be analyzed in relation to fracture classification.
**Results**: Lower-grade fractures (Class ≤ 2, Type 1/2A, subtype A) are expected to show favorable outcomes with conservative management. In contrast, higher grade fractures (Class ≥ 3, Type 2B/3B/4, subtype B) are anticipated to be associated with an increased need for open reduction and internal fixation and improved outcomes following surgical intervention.
**Conclusion**: This proposed classification system offers a practical, treatment-oriented framework that links fracture characteristics with management decisions and outcomes. It has the potential to serve as a simple, reproducible tool that can guide surgical decision-making and optimize outcomes in zygomaticomaxillary complex fractures.

**FA26/372—Trauma**
**3D Condylar Remodeling Following Conservative Management in an Orthodontic Patient: An 18-Month Longitudinal Study**
Sudirman Sudirman ^1^, Anton Anton ^2^, Irwin Setiadi ^3^ and Bramasto Purbo Sejati ^3^
*^1^* *Faculty of Dentistry Universitas Pelita Harapan, Siloam Hospital Lippo Village, Tangerang Regency, Indonesia*
*^2^* *Rumah Sakit Khusus Gigi dan Mulut Pemprov Sumatera Selatan, Palembang, Indonesia*
*^3^* *Faculty of Dentistry Universitas Gadjah Mada, Sleman, Indonesia*
**Objectives**: Management of mandibular condylar fractures remains a clinical debate between open reduction internal fixation (ORIF) and conservative protocols. While surgery provides immediate reduction, conservative therapy utilizes the biological potential for functional remodeling, particularly in young adults. This report details the 18-month evolution of a fracture, focusing on osseous union and morphological adaptation.
**Materials and Methods**: A 21-year-old female undergoing active orthodontic treatment presented with a unilateral left condylar fracture. A non-surgical approach was selected due to the patient’s age and the stability of her orthodontic appliances. Intermaxillary fixation (IMF) via screws was maintained for eight weeks, followed by structured functional therapy.
**Results**: Serial orthopantomograms (OPG) at 1 and 2 weeks, and 1, 2, 3, and 18 months documented the healing process. Early follow-ups (1–3 months) showed progressive callus formation and cortical bridging. By 18 months, OPG and CBCT confirmed complete radiographic union and sophisticated 3D structural remodeling. The CBCT revealed a well-contoured neocondyle harmoniously integrated with the glenoid fossa. Functionally, the patient achieved excellent maximal interincisal opening (MIO) and stable occlusion without orthodontic interference.
**Conclusion**: This case underscores the remarkable regenerative and adaptive capacity of the mandibular condyle in young adults. The 18-month longitudinal data provide robust evidence that conservative management, supported by precise IMF and rigorous clinical monitoring, can yield anatomical and functional results comparable to surgical intervention. In patients with specific dental considerations, such as ongoing orthodontics, this approach offers a highly effective, minimally invasive alternative.

**FA26/395—Trauma**
**Contemporary Surgical Management of Pediatric Mandibular Fractures: A 15-Year Tertiary Center Cohort**
Rafael Rocha ^1^, Margarida Godinho ^2^ and José Guimarães Ferreira ^1^
*^1^* *Department of Plastic Surgery, Unidade Local de Saúde Santa Maria, Plastic and Reconstructive Surgery Clinic, Faculty of Medicine of the University of Lisbon, Lisbon, Portugal*
*^2^* *Faculty of Medicine of the University of Lisbon, Lisbon, Portugal*
**Objectives**: To characterize the surgical management of pediatric mandibular fractures in a tertiary referral center over a 15-year period, and to evaluate patterns of treatment selection in relation to patient age, fracture characteristics, and fixation strategy.
**Materials and Methods**: A retrospective cohort study was conducted including all patients <18 years undergoing surgical management for mandibular fractures at a tertiary center between January 2008 and December 2023. Demographic data, mechanism of injury, fracture pattern, treatment modality (closed reduction with intermaxillary fixation (IMF), open reduction and internal fixation (ORIF), or combined approaches), type of osteosynthesis material (resorbable vs. non-resorbable), and postoperative complications were analyzed. Patients were stratified into three age groups (0–6, 7–12, 13–18 years). Associations between age and treatment strategy were assessed.
**Results**: Seventy-eight patients with 142 mandibular fractures were included (mean age 12.2 ± 4.0 years; male predominance 4:1). The most frequent mechanisms of injury were road traffic accidents (26.9%), falls (24.4%), and bicycle-related trauma (21.8%). Treatment modalities included ORIF in 37.2% of cases, IMF in 34.6%, and combined approaches in 21.8%. No significant association was found between patient age and choice of treatment modality (*p* = 0.273). In contrast, the use of resorbable fixation systems was significantly associated with younger age groups (*p* < 0.001). The mean clinical follow-up was 7.4 months, during which the overall complication rate was 17%, consistent with previously reported pediatric series.
**Conclusion**: Surgical management of pediatric mandibular fractures is heterogeneous and not solely determined by patient age. While treatment modality does not differ significantly across age groups, fixation strategy (particularly the use of resorbable systems) reflects age-specific considerations related to craniofacial growth. These findings support individualized decision-making over simplified age-based treatment paradigms.

**FA26/404—Trauma**
**Characteristics and Patterns of Maxillofacial Soft Tissue Injuries in Hospitalized Pediatric Patients at Temanggung Regional Hospital: A Retrospective Study**
Muhammad Reza Pahlevi ^1^, Bramasto Purbo Sejati ^1^ and Pingky Krisna Arindra ^1^
*^1^* *Department of Oral and Maxillofacial Surgery, Faculty of Dentistry, Universitas Gadjah Mada, Yogyakarta, Indonesia*
**Objectives**: Maxillofacial soft tissue injuries in pediatric patients are frequently associated to traumatic events and may coexist with dentoalveolar and facial fractures. However, data focusing on injury patterns and associated injuries among hospitalized pediatric patients remain limited. This study aimed to analyze the injury patterns and associated characteristics of maxillofacial soft tissue injury in hospitalized pediatric patients.
**Materials and Methods**: A retrospective study was conducted on hospitalized pediatric maxillofacial soft tissue trauma patients in Temanggung Regional Hospital between January 2023 to December 2025. Demographic and clinical data including age, sex, injury etiology, location of trauma, length of hospital stay, type and distribution of soft tissue injuries, and associated injuries were collected from hospital medical record. Chi-square analysis were performed to examine associations between variables.
**Results**: A total of 130 patients were included in our study. Male patients predominated (68.46%), with the highest incidence observed in adolescents aged 15–18 years (57.69%). Traffic accidents were the leading etiology (72.3%), indicating a predominance of high-energy trauma mechanisms. Falls were the most common etiology in children under 10 years old. Lacerations were the most frequent injury type (43.08%), followed by combination of laceration and abrasion (37.69%). Lip laceration was the most common facial injury (44.44%), followed by chin laceration (33.33%) and cheek abrasion (28.45%). Labial mucosa was the most common intraoral injury (56.77%). Dentoalveolar fractures (25.67%) and zygomaticomaxillary complex fractures (11.23%) were the most frequent associated injuries. Adolescents and traffic accidents were associated with complex injuries including combination of laceration-abrasion, and extraoral-intraoral involvement.
Conclusion: Maxillofacial soft tissue injuries in hospitalized pediatric patient predominantly affect male adolescents and are frequently associated with dentoalveolar and midfacial fractures. Adolescent age and high-energy trauma are linked to more complex injury patterns. These findings may assist clinicians in early risk stratification when managing maxillofacial injuries in pediatric patients.

**FA26/416—Trauma**
**Dentoalveolar Injury Patterns Across Craniofacial and Multisystem Trauma Phenotypes: A Five-Year Retrospective Analysis in Temanggung Regional Hospital**
Nathaniel Dean Wanaditya ^1^, Bramasto Purbo Sejati ^1^ and Didit Istadi ^1^
*^1^* *Department of Oral and Maxillofacial Surgery, Universitas Gadjah Mada, Yogyakarta, Indonesia*
**Objectives**: Dentoalveolar trauma is often considered a localized injury of the teeth and supporting alveolar structures. In a hospital trauma setting, however, these injuries may also present as part of a wider craniofacial or multisystem trauma pattern. Although previous studies have commonly reported traumatic dental injuries in relation to age, sex, cause of injury, and type of dental trauma, few have examined how dentoalveolar trauma characteristics vary according to the presence and extent of associated injuries. This study aimed to evaluate the clinical characteristics of dentoalveolar trauma and compare injury patterns between isolated and polytrauma-associated dentoalveolar injuries.
**Materials and Methods**: A retrospective cross-sectional study was conducted to assess all patients diagnosed with a dentoalveolar trauma who presented to Temanggung Regional Hospital from September 2020 to September 2025. Data were obtained from electronic health record systems and Chi Square analysis was performed.
**Results**: Review of 171 dentoalveolar trauma cases highlighted a slight male predominance (59.65%) and affects mostly adults aged 31–60 years (29.82%). Motor vehicle accident (79.53%) was the leading etiology. A strong site predilection for the anterior maxilla (73.06%) was observed, with mostly affect 3–4 teeth (47.95%). Among the recorded dentoalveolar injuries, subluxation was the most common presentation (35.32%), followed by alveolar bone fracture (24.88%). The type of dentoalveolar injury was significantly associated with the affected site and the number of teeth involved (*p* < 0.01).
**Conclusion**: The significant association between injury type, affected anatomical site, and number of teeth involved suggests that dentoalveolar trauma patterns are not randomly distributed, but may reflect differences in trauma mechanism, impact direction, and injury extent. These findings highlight the importance of systematic documentation of injury type, site involvement, and number of affected teeth in the clinical assessment of dentoalveolar trauma.

**FA26/422—Trauma**
**Code, Cut, Reconstruct: AI Redefining Craniofacial Trauma**
Hitesh Bansal ^1^ and Hannah Arnantha ^2^
*^1^* *London North West University Healthcare NHS Trust, London, UK*
*^2^* *St George’s, Epsom and St Helier Hospital Group, London, UK*
**Objectives**: Artificial intelligence (AI) is rapidly emerging as a transformative tool in healthcare. This study aims to review the current and evolving applications of AI in the management of craniofacial trauma, with a focus on its role in diagnosis, surgical planning, and clinical decision-making.
**Materials and Methods**: A narrative literature review was conducted using contemporary publications and technological reports relating to AI in craniofacial trauma. Key domains analysed included AI-assisted imaging interpretation, predictive analytics, and integration with advanced technologies such as 3D modelling and surgical planning tools. Relevant developments in machine learning, particularly convolutional neural networks (CNNs), were evaluated alongside their clinical applicability and limitations.
Results: AI applications in craniofacial trauma are most advanced in imaging analysis, where CNNs demonstrate the ability to detect facial fractures on CT imaging with high speed and accuracy. These systems can reduce clinician workload and support diagnostic consistency, although challenges remain in sensitivity, dataset bias, and anatomical variability.
AI also shows promise in predictive analytics, supporting clinical decision-making through pattern recognition and outcome prediction. Integration with imaging data enables enhanced surgical planning, including segmentation and the development of patient-specific models. However, limitations include the risk of automation bias, lack of transparency in algorithm decision-making, and the need for robust validation in diverse clinical settings.
Emerging applications include AI-assisted workflow optimisation, virtual planning, and potential integration with augmented reality and navigation systems, although these remain largely investigational.
**Conclusion**: AI has significant potential to enhance the management of craniofacial trauma, particularly in diagnostic imaging and preoperative planning. While early results are promising, widespread clinical adoption requires further validation, improved algorithm robustness, and careful integration into clinical workflows to ensure safe and effective use. AI is likely to augment, rather than replace, clinician expertise in the foreseeable future.

**FA26/426—Trauma**
**Effect of Treatment Delay on the Risk of Post-Operative Complications of Mandible Fractures**
Sean Harrop ^1^, Justin Lee ^1^, Ralph Hsiao ^1^, Gorman Louie ^1^ and Joshua DeSerres ^1^
*^1^* *University of Alberta, Edmonton, Canada*
**Objectives**: Mandible fractures are common in craniomaxillofacial trauma. Patients often wait several days for surgery in resource-constrained healthcare systems. This study aims to identify an association between time to fracture repair and risk of complications to determine if patients need to remain admitted to hospital while awaiting surgery. Secondary exploratory analyses were performed to identify any other factors that increase the risk of post-operative complications.
**Materials and Methods**: A retrospective chart review was performed of all consecutive mandible fractures treated by a single surgeon at the University of Alberta Hospital from 2012 to 2024. Inclusion criteria were adult patients who underwent operative treatment for a traumatic mandible fracture. Ballistic injuries and out-of-province patients were excluded. Logistic regression was performed to assess for variables associated with increased complication risk.
**Results**: 306 patients met the selection criteria. There were 248 (81%) males and 58 (19%) females, with a median age of 30 years (IQR, 23–44). 51 (16.7%) patients had a complication, with 26 (8.5%) experiencing post-operative infection. Delay to surgery over 7 days was not correlated with post-operative complications (*p* = 0.77). 139 patients were admitted to hospital at time of presentation and 158 patients were discharged home and readmitted electively for surgery. No difference was seen in complications for patients admitted to hospital compared to those who were discharged while awaiting surgery (*p* = 0.14). Complications, however, were significantly associated with older age (median 36 vs. 29 years, *p* = 0.033) and substance use (*p* = 0.001).
**Conclusion**: Neither time to operative management nor outpatient management of mandible fractures increased the risk of post-operative complications. Post-operative complications were associated with age and substance use. With appropriate patient selection, patients with mandible fractures may be discharged home and readmitted electively for surgical management with no increased risk of complications.

**FA26/428—Trauma**
**Huge Facial Trauma After Bear Attack**
Mircea Rivis ^1^, Mircea Balasoiu ^2^, Andrei Uritu ^2^, Andrada Staicu ^2^ and Ciprian Roi ^1,3^
*^1^* *University Clinic of Anesthesiology and Oral Surgery, Research Center of Dento-Alveolar Surgery, Anesthesia and Sedation in Dental Medicine, “Victor Babes” University of Medicine and Pharmacy, Eftimie Murgu Sq. No. 2, 300041, Timisoara, Romania, Timisoara, Romania*
*^2^* *University Clinic of Anesthesiology and Oral Surgery, Doctoral school, Research Center of Dento-Alveolar Surgery, Anesthesia and Sedation in Dental Medicine, “Victor Babes” University of Medicine and Pharmacy, Eftimie Murgu Sq. No. 2, 300041, Timisoara, Romania, Timisoara, Romania*
*^3^* *University of Medicine and Pharmacy “Victor Babes” Timisoara, Romania, Timisoara, Romania*
**Objectives**: The primary objective of this study is to delineate the surgical management and reconstructive strategies for extensive maxillofacial trauma resulting from an animal-inflicted injury. It aims to evaluate the effectiveness of rigid internal fixation in restoring anatomical integrity and facial projection in highly comminuted fractures.
**Materials and Methods**: We present the management of a male patient with multi-fragmentary midfacial fractures and catastrophic soft tissue lacerations following a bear attack. Under general anesthesia, the surgical intervention involved thorough debridement followed by open reduction and internal fixation (ORIF). A comprehensive system of titanium plates and screws was utilized to stabilize the facial pillars and restore orbital volume, using remaining skeletal landmarks for anatomical guidance
**Results**: The surgical approach achieved successful restoration of the facial framework and aesthetic contour. Rigid internal fixation provided immediate stability, allowing for early functional rehabilitation. Meticulous wound management and targeted antibiotic protocols prevented major infectious complications despite the contaminated nature of the injury. Follow-up evaluations confirmed the successful restoration of facial relief, symmetry, and basic physiological functions.
**Conclusion**: Managing massive maxillofacial trauma from bear attacks requires a systematic approach involving aggressive debridement and precise internal fixation. The use of titanium osteosynthesis remains the gold standard for restoring structural support and facial relief. Early, comprehensive reconstruction is essential for achieving optimal functional and aesthetic outcomes in complex craniofacial injuries.

**FA26/443—Trauma**
**Measuring What Matters: A Systematic Review of Quality of Life PROMs for Patients with Orbital Trauma**
Saranda Ombashi ^1^, Parinaz Rostamzad ^1^, Max Wevers ^1^, Elske Strabbing ^1^, Ozlem Engin ^1^ and Eppo Wolvius ^1^
*^1^* *Erasmus Medical Center, Rotterdam, The Netherlands*
**Objectives**: Orbital fractures are common in facial trauma, yet outcome assessment predominantly relies on clinician-reported parameters that may not reflect patient experience. This study aimed to systematically review the literature on the availability, application, and methodological quality of quality-of-life (QoL) Patient-Reported Outcome Measures (PROMs) used in patients with orbital fractures, and to evaluate their content in relation to relevant health domains.
**Materials and Methods**: A systematic review was conducted in accordance with PRISMA-COSMIN guidelines. Multiple databases were searched, studies were screened independently by two reviewers. Studies were included if they assessed QoL in orbital fracture patients using validated PROMs. Extracted PROM items were analyzed using a concept-sort. Data was categorized into health domains. Methodological quality was assessed using the COSMIN risk of bias checklist. Determinants associated with QoL outcomes were identified.
**Results**: The search yielded 3024 studies, of which seven met inclusion criteria, comprising nine PROMs. A total of 225 items were identified and categorized into physical, psychological, social, esthetic, and general QoL domains. Physical functioning was most frequently represented (n = 58), primarily focusing on activities of daily living, followed by psychological impact (n = 35) and social interaction (n = 33). Vision-related aspects were underrepresented across most PROMs. Overall methodological quality was low. A significant association with QoL was seen in one determinant, namely surgical treatment. No PROMs were specifically developed or validated for orbital fracture populations, although one study reports about the development of a new (future) PROM for facial trauma, including an orbital scale.
**Conclusion**: Currently available PROMs applied in orbital fracture patients lack population-specific validation and insufficiently address vision-related outcomes. Existing instruments predominantly emphasize general physical functioning rather than ocular and esthetic consequences. Future research should focus on the development or adaptation of a facial trauma–specific PROM incorporating eye-related domains, followed by validation in orbital fracture populations.

**FA26/464—Trauma**
**Surgical Approach of the Lower Eyelid in Trauma Patients. Subciliar vs. Transconjuntival Approach. Analysis of 150 Cases by a Single Operator**
Rodrigo Liceaga-Reyes ^1^
*^1^* *Hospital de Especialidades, AO CMF LATAM, CAMPECHE, Mexico*
**Objectives**: To determine in the quantitative and qualitative analysis the result and the patient’s satisfaction with the outcome obtained in 150 consecutive cases operated on by the same surgeon using two surgical approaches (subciliary and transconjunctival).
**Materials and Methods**: A satisfaction survey was conducted in 150 patients undergoing lower orbital rim surgery for facial trauma using (subciliary and transconjunctival) approaches. The choice of approach was made by the surgeon. 114 men and 36 women operated on over three and a half years were included. All were operated on by the same surgeon. Patients were evaluated at weeks 1, 3, 6, 10, and 20. The patient provided informed consent and completed a questionnaire in which they assessed their degree of satisfaction. Patients who did not complete the evaluation on all four occasions were excluded from the study.
**Results**: A total of 113 patients completed the evaluations on all 4 occasions. The rest of the cases were not considered for the analysis of this study. Of the total 113 patients, 91 were men and 22 women. Of the total 113 patients, 78 underwent the subciliary approach, 35 the transconjunctival approach. The total satisfaction percentage was 9.6 on a scale from 0 to 10. There was no significant difference in the evaluation by sex. There was no significant difference in the evaluation across the different weeks.
**Conclusion**: Although the literature continues to have controversies about which approach is superior to the other, this study helps to show in a controlled clinical manner that the patient’s evaluation is not significantly different between both approaches and the clinical outcome is very similar over the weeks.

**FA26/469—Trauma**
**Characteristics and Patterns of Maxillofacial Fractures in Geriatric Patients at Temanggung Regional Hospital: A Retrospective Study**
Tio Hara Sujarwanta ^1^, Bramasto Purbo Sejati ^1^ and Pingky Krisna Arindra ^1^
*^1^* *Department of Oral and Maxillofacial Surgery, Faculty of Dentistry, Universitas Gadjah Mada, Yogyakarta, Indonesia*
**Objectives**: This study aimed to describe the demographic characteristics, fracture patterns, etiology, treatment modalities, and clinical outcomes of maxillofacial fractures in geriatric patients at RSUD Temanggung.
**Materials and Methods**: A retrospective descriptive-analytical study was conducted on medical records of 56 geriatric patients (≥60 years) with maxillofacial fractures treated at RSUD Temanggung from September 2020 to August 2025. Variables included age, sex, fracture type and region, etiology, treatment modality, length of hospital stay, and mortality. Statistical analyses included descriptive statistics, Kruskal-Wallis test, Mann-Whitney U test, Chi-square test, and Spearman correlation (α = 0.05).
**Results**: Of 56 patients, 32 were male (57.1%) and 24 female (42.9%), yielding a male-to-female ratio of 1.45:1. Mean age was 66.8 ± 5.8 years; the 60–64 year group predominated (44.6%). Maxillary fracture was the most common type (35.7%), followed by dentoalveolar (25.0%), mandibular (14.3%), and condylar fractures (12.5%). Road traffic accidents were the leading etiology (66.1%), followed by falls (21.4%). Surgical intervention was performed in 85.7% of cases. Mean length of hospital stay was 5.1 ± 2.5 days, with a mortality rate of 1.8%. No significant associations were found between length of stay and fracture type (*p* = 0.645), sex (*p* = 1.000), fracture region (*p* = 0.924), or age (ρ = 0.316, *p* = 0.095).
**Conclusion**: Maxillofacial fractures in geriatric patients at RSUD Temanggung predominantly affect males in the early geriatric age group, with maxillary fractures from road traffic accidents as the most common presentation. The high surgical rate and short hospital stay indicate effective management. The absence of significant associations across subgroups suggests consistent care delivery.

**2FA26/1007—Trauma**
**Reconstruction Timing in Mandibular Gunshot Wounds: A Comparative Case Series**
Theodore Kao ^1^
*^1^* *Cook County Health, Chicago, IL, USA*
**Objectives**: Gunshot wounds (GSWs) to the mandible and facial skeleton require complex, time-sensitive reconstruction. Optimal sequencing of fixation relative to injury severity and systemic condition remains debated, spanning immediate fixation, early staged damage-control, early single-stage reconstruction, and delayed staged approaches. This case series compares different levels of injuries, reconstructive philosophies to characterize timing-dependent clinical trajectories, surgical and functional outcomes.
**Materials and Methods**: A retrospective case series of four patients with ballistic maxillofacial trauma was performed at a single tertiary care, level I trauma center. Injuries included comminuted mandibular fractures with variable maxillofacial, and skull bone extension. Patients were categorized by timing strategy: immediate, early staged, early one-stage, and delayed staged reconstruction. Outcomes included timing to fixation, ICU use, hospital length of stay (LOS), airway management, revision surgeries, and dental rehabilitation milestones.
**Results**: Across cases, increasing injury severity and systemic burden correlated with delayed definitive fixation, prolonged ICU utilization, and extended recovery trajectories. Immediate reconstruction achieved same-day ORIF with the shortest LOS, but still required later revision. Early staged reconstruction was delayed due to multisystem trauma, with prolonged ICU stay and delayed recovery. Early one-stage reconstruction allowed comprehensive skeletal restoration (mandible, orbit, and midface) within 4–5 days, resulting in efficient anatomical restoration and intermediate LOS. Delayed staged reconstruction demonstrated the most prolonged course, requiring sequential, multiphase cranio-maxillofacial repairs, with extended ICU dependence, tracheostomy management, and infectious/metabolic complications, followed by delayed rehabilitation.
**Conclusion**: GSW-related mandible and maxillofacial reconstruction demonstrates a variety between timing of definitive fixation, defect and injury involvement, and systemic complexity. Immediate and early one-stage approaches enable rapid structural restoration, whereas staged and delayed strategies prioritize physiologic stability at the cost of prolonged LOS and functional recovery period. Therefore, a systemic- and injury-driven algorithm is essential for optimizing outcomes in GSW-related cranio-maxillofacial trauma.

**2FA26/1064—Trauma**
**“Jaw Fracture Management at Dental Clinic. Emergency Management Approach—A Case Series and Review of Literature.”**
Althea Bumpu ^1^
*^1^* *Port Moresby Jacksons International, Papua New Guinea Dental Association, National Capital District Provincial Health Authority- Dental Division, Port Moresby, Papua New Guinea*
**Objectives**: To explain the significance of Jaw Fracture (JF) as an emergency case while revealing the best Jaw Fracture management in an emergency approach and to show that jaw fracture management at an emergency is purposely to rehabilitate the normal occlusion, function and stability of the jaw bone.
**Materials and Methods**: Two cases attended to at a local dental clinic was assessed and compared by their outcomes after management. This case report is a presentation of two clinical cases of Mandibular JF due to physical assault. **One was presented to the clinic immediately after trauma while the other came 7 days later. This difference in duration had a significant impact on the post-operative outcome**. Both are males of 30 years, from the highlands region of PNG having Mandibular fracture at the symphysis region. Skull Radiographs shown a radiolucent vertical line separating the radiopaque mandible creating a discontinuity in the bone anatomy of mandible.
**Results**: Clinically, both cases showed a marked malocclusion in the anterior mandibular dentition with soft tissue injuries. They were both covered on antibiotics and managed with close reduction under administration of local anaesthesia. The malocclusion was achieved by manually pushing the jaw while doing wire splinting. However, the case presenting later than 7 days came back with poor prognosis and malocclusion.
**Conclusion**: Literatures reviewed signifies that JF is a dental emergency and must be attended to immediately whether with intermaxillary fixation or osteosynthesis. This is mainly because of the bone remodelling process that happens within 7 days and may cause permanent complications gif not given immediate management.

**2FA26/1091—Trauma**
**Forgotten But Functional: Intraosseous Wiring in Mandible Fracture Repair**
Nur adibah Mat Zubir ^1^, Liyana Shafiqah Ahmad Muzamir ^1^, Joon Wei Tan ^1^ and Aiman Mohd Azmi ^1^
*^1^* *Oral and Maxillofacial Department, Hospital Tengku Ampuan Afzan, Kuantan, Malaysia*
**Objective:** This case report aims to highlight the continued role of intraosseous wiring in mandibular fracture management, particularly in selected cases where modern fixation options such as reconstruction plates may not be feasible and limited. It also emphasizes the importance of managing postoperative complications in immunocompromised patients.
**Materials/Method:** A 40-year-old Malaysian male prisoner with advanced HIV and Hepatitis C presented with one month of mandibular pain and swelling following facial trauma. Clinical assessment revealed a right facial abscess secondary to untreated fractures involving the right angle and left body of the mandible. The patient underwent open reduction and internal fixation (ORIF) using intraosseous wiring, sequestrectomy, extraction of teeth 34 and 35, and arch bar placement under general anesthesia in March 2026. Postoperatively, he was managed with prolonged antibiotics, wound irrigation, and daily dressing due to persistent surgical site infection.
**Result:** Intraosseous wiring offered a straightforward, affordable, and easily accessible fixation technique, particularly in considering the patient’s welfare and financial limitations. With appropriate postoperative monitoring, satisfactory mandibular alignment and occlusal restoration were achieved, even though this technique provides less rigid stability than conventional plating techniques and may necessitate prolonged maxillomandibular fixation. The case further highlighted the necessity of closely monitoring and controlling infections in people with impaired immune systems.
**Conclusion:** Intraosseous wiring is no longer considered a routine first-line treatment for mandibular fractures; however, it remains a useful option in carefully selected cases where modern fixation methods are limited or unavailable. With appropriate case selection, stable fixation, occlusal restoration, and close follow-up, satisfactory outcomes may still be achieved.
*Keywords: intraosseous wiring, immunocompromised patients, open reduction internal fixation*

**2FA26/1114—Trauma**
**A novel Innovation—Dynamic Compression screws: Enhancing Anatomical Alignment in Parasymphysis of mandible fracture**
Anandapadmanaban S ^1^, Venkadasalapathi Narayanasamy ^1^ and Senthilkumar palanichamy ^2^
*^1^* *Hannah Joseph Hospital, Madurai, India*
*^2^* *Maeoris Dental, Dindigul, India*
**Objectives**: Precise anatomical reduction and stable fixation are essential in the management of mandibular parasymphysis fractures to restore function, occlusion, and facial symmetry. This study evaluates the effectiveness of dynamic compression screws (DCS) used for mandibular parasymphysis fracture reduction from 2022 to 2025, with emphasis on three-dimensional anatomical reduction and control of lingual splaying.
**Materials and Methods**: A clinical and radiological evaluation was conducted on patients with mandibular parasymphysis fractures treated with open reduction and internal fixation using dynamic compression screws between 2022 and 2025. Surgical management involved anatomical reduction followed by DCS fixation to achieve controlled interfragmentary compression. Postoperative assessment included clinical evaluation of occlusion, fracture stability, mouth opening, and functional recovery. Radiographic assessment using an computed tomography (CT) imaging was performed to evaluate three-dimensional anatomical reduction, lingual cortical alignment, and lingual splaying. Patients were followed periodically to assess healing and complications.
**Results**: Dynamic compression screws provided satisfactory fracture reduction and stable fixation in mandibular parasymphysis fractures. Improved three-dimensional anatomical alignment and effective control of lingual splaying were observed postoperatively. Patients demonstrated stable occlusion, reduced interfragmentary mobility, and favorable healing outcomes with minimal postoperative complications.
**Conclusion**: Dynamic compression screws demonstrated promising clinical effectiveness in mandibular parasymphysis fracture management. The technique provided reliable fixation, enhanced three-dimensional anatomical reduction, and improved control of lingual splaying, contributing to satisfactory healing and functional recovery.

**2FA26/1117—Trauma**
**Management of Post-Fixation Secondary Extrusion of an Intruded Maxillary Central Incisor Following Arch Bar Fixation in Dentoalveolar Fracture: A Case Report**
Lilis Ria Ariana ^1^, Cahya Hasan ^1^, Poerwati Soetji Rahajoe ^1^ and Yosaphat Bayu Rosanto ^1^
*^1^* *Universitas Gadjah Mada, Yogyakarta, Indonesia*
**Objectives**: Dentoalveolar fractures including intrusive and extrusive luxation, require early repositioning and stabilization to ensure optimal pulpal and periodontal repair. Although arch bar fixation is widely used, inadequate vertical control can lead to secondary tooth displacement. This case report describes the utilization of delayed secondary wiring as an adaptive corrective method to manage post-fixation secondary extrusion of a previously intruded central incisor caused by the mechanical limitations of conventional arch bar fixation.
**Materials and Methods**: A 25-year-old female presented after a motorcycle accident with a maxillary dentoalveolar fracture, severe intrusion of tooth 11, and extrusive luxation of teeth 21 and 22, causing malocclusion. Emergency management involved wound debridement, manual repositioning of the luxated teeth, and arch bar splinting under local anesthesia to restore occlusion and stabilize the dentoalveolar segment. At the 7- and 14-day follow-up showed a loss of vertical stability, resulting in the secondary extrusion of tooth 11 due to insufficient vertical retention of the arch bar. Corrective management was immediately performed via manual repositioning of the displaced tooth and additional tooth-specific wire stabilization on tooth 11 to enhance individual vertical control and maintain its position within the arch.
**Results**: The application of delayed secondary wiring achieved satisfactory clinical stabilization by improving individual tooth control and preventing further vertical displacement. At 42 days post-operation, the entire splinting apparatus was removed after clinical stabilization of the dentoalveolar segment. Evaluation upon removal demonstrated clinical stability, absence of pathological mobility, stable occlusion, and satisfactory panoramic radiographic outcomes showed preserved lamina dura continuity and dense alveolar bone around tooth 11.
**Conclusion**: Post-fixation secondary extrusion can occur when arch bars provide insufficient vertical control for repositioned intruded teeth. Early recognition and additional wire stabilization are crucial for dentoalveolar stability, while long-term pulpal and periodontal follow-up remains mandatory to monitor for late complications.

**2FA26/1200—Trauma**
**Reconstructing the Midfacial Framework: A Sequential Buttress-Oriented Approach to Combined Le Fort II and Zygomaticomaxillary Complex Fractures**
Bebby Maulida ^1^, Bramasto Purbo Sejati ^1^ and Pingky Krisna Arindra ^1^
*^1^* *Universitas Gadjah Mada, Yogyakarta, Indonesia*
**Objectives**: Combined Le Fort II and zygomaticomaxillary complex (ZMC) fractures represent complex midfacial injuries characterized by disruption of multiple facial buttresses, resulting in altered facial projection, asymmetry, and occlusal instability. In such cases, successful reconstruction depends not only on rigid fixation but also on appropriate sequencing of reduction and restoration of the three-dimensional midfacial framework.
**Materials and Methods**: A 46-year-old male presented with severe midfacial trauma following a road traffic accident. Clinical examination revealed facial edema, left malar depression, restricted mouth opening (1.5 cm), edge-to-edge occlusion, positive Guerin sign, and maxillary mobility. Three-dimensional computed tomography demonstrated combined Le Fort II and displaced left ZMC fractures associated with frontal and temporal bone fractures. Open reduction and internal fixation were performed through combined lateral eyebrow, subtarsal, and bilateral maxillary vestibular approaches. Reconstruction was guided by a sequential buttress-oriented strategy, restoring zygomatic projection and midfacial width before definitive stabilization of the infraorbital rims, zygomaticomaxillary buttresses, and occlusal relationship. Titanium miniplates and screws were applied at key buttress regions, and interdental wiring arch bars were used for occlusal stabilization.
**Results**: At 2-month follow-up, restoration of facial projection, zygomatic symmetry, mouth opening, and stable dental occlusion was achieved. No visual disturbance, persistent neurosensory deficit, infection, wound dehiscence, or hardware-related complications were observed.
**Conclusion**: Complex combined Le Fort II and ZMC fractures require reconstruction based on restoration of the three-dimensional facial buttress framework rather than fracture lines alone. A sequential buttress-oriented approach can facilitate predictable functional and esthetic outcomes while providing a practical strategy for managing complex midfacial trauma.

**2FA26/1201—Trauma**
**Surgical Management of Guardsman Fracture in Pediatric Patient Using Thermoplastic Mandibular Cap Splint: A Case Report**
Mirna Oktavia Sari ^1^, Pingky Krisna Arindra ^1^ and Didit Istadi ^1^
*^1^* *University of Gadjah Mada, Yogyakarta, Indonesia*
**Objectives**: Guardsman fracture or parade fracture, is classically defined as mandibular condylar fractures associated with a symphyseal or parasymphyseal fracture resulting from direct impact to the chin. Variants involving unilateral condylar fractures with associated anterior mandibular fractures are uncommon, particularly in pediatric patients, and present unique management challenges due to ongoing craniofacial growth and developing dentition. To report the surgical management and clinical outcome of a pediatric Guardsman fracture consisting of a unilateral mandibular condylar fracture associated with a displaced mandibular symphyseal fracture treated using a thermoplastic mandibular cap splint and postoperative Barton head bandage immobilization.
**Materials and Methods**: A 5-year-old boy presented with pain during mastication, facial asymmetry, malocclusion, limited mouth opening, and mandibular deviation following facial trauma. Clinical and radiographic examinations confirmed a right mandibular condylar fracture associated with a displaced mandibular symphyseal fracture. Surgical treatment was performed under general anesthesia through anatomical reduction and fixation of the symphyseal fracture using wiring techniques, followed by stabilization with a custom-made thermoplastic mandibular cap splint. A Barton head bandage was applied postoperatively to provide additional mandibular support. Clinical and radiographic evaluations were conducted periodically 6-month follow-up period.
**Results**: Postoperative evaluation demonstrated satisfactory fracture healing with improved occlusion, mouth opening, mandibular alignment, and facial symmetry. No major complications, infection, temporomandibular joint dysfunction, mandibular deviation, or growth disturbance were observed throughout the 6-month follow-up period. The thermoplastic cap splint combined with Barton bandage immobilization provided favorable functional and esthetic outcomes while preserving mandibular growth potential.
**Conclusion**: Surgical reduction combined with thermoplastic mandibular cap splint stabilization and postoperative Barton head bandage immobilization may provide an effective minimally invasive treatment option for pediatric Guardsman fracture involving unilateral condylar and displaced symphyseal fractures. This approach offers adequate stabilization, supports functional rehabilitation, and preserves mandibular growth potential while minimizing the risk of long-term complications.

**2FA26/1217—Trauma**
**Updated Clinical Practice Guideline: Management of Isolated Mandibular Fractures in Adults**
Karen Mae Ty ^1^, Jose Florencio Lapeña Jr. ^2^, Jehan Grace Maglaya ^3^, Joselito David ^4^, Jaypee Amable ^3^, Candice Que ^5^, Dann Joel Caro ^6^ and Veronica Jane Yanga-Rabe ^7^
*^1^* *University of the East Ramon Magsaysay Memorial Medical Center Inc., Jose R. Reyes Memorial Medical Center, Quezon City, Philippines*
*^2^* *Healthway Medical Network Philippines Inc., Ayala Health, University of the Philippines College of Medicine and Philippine General Hospital, Mak, Philippines*
*^3^* *University of the East Ramon Magsaysay Memorial Medical Center Inc., Quezon City, Philippines*
*^4^* *University of Santo Tomas Hospital, Manila, Philippines*
*^5^* *Manila Central University Filemon D Tanchoco Medical Foundation, Caloocan, Philippines*
*^6^* *Ilocos Training and Regional Medical Center, San Fernando City, Philippines*
*^7^* *Manila Doctors Hospital, Manila, Philippines*
**Objectives:** To present the updated clinical management of isolated mandibular body fractures in adults that is relevant to current practice.
**Materials and Methods:** A systematic literature search was conducted from January 2018 to April 2026 across major international and local databases. Retrieved studies were independently appraised and classified, and the 15 key action statements were reviewed by the Guideline Development Group using a Delphi consensus process.
**Results:** The update includes 15 key action statements covering clinical diagnosis, ancillary diagnostic tests, definitive treatment, and preventive measures. Recent advances in mandibular fracture management were identified and evaluated. However, the available evidence did not warrant changes to the existing recommendations.
**Conclusion:** This updated resource serves as an implementation guide for evidence-based management of isolated mandibular body fractures in adults. Contemporary evidence supports the continued validity and robustness of the 2021 Clinical Practice Guideline recommendations while providing clinicians with current information relevant to practice.

**2FA26/1225—Trauma**
**Clinical Patterns and Severity of Le Fort I and II Fractures in a Regional General Hospital: A 5-Year Retrospective Study**
Rinan Dihana ^1^, Bramasto Purbo Sejati ^1^ and Cahya Hasan ^1^
*^1^* *Universitas Gadjah Mada, Yogyakarta, Indonesia*
**Objectives**: Maxillofacial fractures, particularly Le Fort types, are significant indicators of high-energy trauma. Facial fracture severity and concomitant injuries are hypothesized to correlate directly with higher Le Fort classifications. This study aim to describe and analyze the characteristics and facial injury severity associated with Le Fort I and II fractures.
**Materials and Methods**: A retrospective study was conducted on patients diagnosed with Le Fort I and II fractures between 2020 and 2025. Data on demographics, etiology, FISS scores, concomitant injuries, and length of stay (LOS) were extracted from the medical records of a regional general hospital in Central Java, followed by a bivariate analysis.
**Results**: Out of 52 identified cases, Le Fort I fractures predominated (59.62%), with males (76.9%) and the 20–29 age group (23.08%) being the most affected. Road traffic accidents were the primary etiology (94.32%). The mean hospital length of stay was 6.47 ± 2.05 days, and 15% of patients required ICU admission. Concomitant brain injuries were present in 76.9% of patients and extremity fractures were found in 25%. Statistical analysis showed no significant association (*p* > 0.001) between fracture classification and length of stay, brain injuries, or extremity fractures. However, the median FISS score was significantly associated with the fracture classification (*p* < 0.001), being significantly higher in Le Fort II [5.00, IQR 5–6] than Le Fort I fractures [3.00, IQR 3–4].
**Conclusion**: Le Fort fractures predominantly affect young adult males due to road traffic accidents and are highly associated with concomitant brain injuries. Although Le Fort II fractures present with significantly higher FISS scores than Le Fort I, the fracture classification does not significantly influence hospital length of stay or the risk of sustaining associated brain and extremity traumas.

**2FA26/1259—Trauma**
**Impact of Virtual Planning and Reduction on the Treatment Of Panfacial Fractures: A Case Report Secondary to a Gunshot Wound**
Ilona Marchenko Kasyanchuk ^1^, Víctor Zafra Vallejo ^1^, Fernando Leporace Jiménez ^1^ and Gregorio Sánchez Aniceto ^1^
*^1^* *Hospital 12 de Octubre, Madrid, Spain*
**Objectives**: The objective was to analyze how virtual surgical planning (VSP) and intraoperative navigation can adapt traditional, rigid sequencing protocols (top-bottom or bottom-top) to the patient-specific needs of complex panfacial fractures lacking stable bone references.
**Materials and Methods**: A 51-year-old male presented with a panfacial fracture secondary to a gunshot wound (frontal sinus, naso-orbito-ethmoid [NOE] type III, Le Fort I, II, and III, and a triple mandibular fracture). Following emergency intervention, a digital workflow was implemented:
  - Virtual segmentation and reduction: Software (3D Slicer, Meshmixer) was utilized to align bone fragments and preserve condylar position.
  - Biomodels and surgical guides: Stereolithographic models were 3D-printed for pre-bending the reconstruction plate and fabricating reduction guides.
  - Assisted surgery: The upper third was approached using navigation (Brainlab) to reconstruct the frontal sinus and NOE region. The mandibular approach was based on the virtual model, enabling precise fixation independent of the midface.
**Results**: The patient demonstrates a satisfactory surgical outcome and occlusion. Currently, a small palatal fistula is present and in the process of resolution. Although VSP improves predictability, technical challenges remain:
  - Limited Simulation: A lack of algorithms that accurately replicate maxillomandibular fixation (MMF) and tactile resistance, which can lead to fragment overlapping.
  - Biomechanical interference: Masticatory muscle forces hinder the actual manipulation of fragments compared to the simulation.
  - Logistics: Design and processing times limit its use in immediate emergency settings.
**Conclusion**: Digital workflow enables more efficient and precise hybrid approaches. However, it does not replace clinical judgment; surgeons must perform intraoperative adjustments due to the lack of tactile feedback in the virtual environment. Standardization of protocols is required for these tools to establish themselves as the gold standard in maxillofacial trauma.

**2FA26/1270—Trauma**
**Reduction and Reconstruction of Zygomatic Complex Fractures with Patient Specific CAD/CAM Implants**
Konstantinos Katoumas ^1^ and Christos Perisanidis ^1^
*^1^* *Department of Oral and Maxillofacial Surgery, Dental School, National and Kapodistrian University of Athens, Athens, Greece*
**Objectives**: Virtual surgical planning offers the opportunity to meticulously study fracture lines, perform mock surgery and find the optimal reduction pattern in zygomatic complex fractures. CAD/CAM patient specific guides and plates can be used to transfer the virtual plan to the patient with precision.
The aim of this study is to present the reduction and fixation of new and secondary zygomatic fractures with CAD/CAM patient specific implants.
**Materials and Methods:** Two cases are analyzed. In both cases a meticulous clinical examination and a thin-slice CT scan were performed. The data were transferred to KLS Martin’s IPS CaseDesigner and virtual surgery was performed reducing the fractures digitally. Then the virtual design of the guides and plates followed.
**Results**: The first patient presented with a complex and severely displaced zygomatic fracture. A CT scan followed by virtual reduction of the fracture was performed. Patient specific CAD/CAD guides and plates were designed and used for the reduction and fixation of the fracture under general anesthesia. The second case regarded a patient with severe diplopia, enophthalmos, hypophthalmos and facial asymmetry in the zygoma area, due to a non-reduced zygomatic fracture. Fracture pattern and healed fracture lines were studied and virtual osteotomies were performed followed by reduction. The mirroring of the non-affected side was also taken into consideration. Osteotomy of the zygomatic complex and reduction and fixation with CAD/CAM patient specific guides and plates was performed under general anesthesia. In the second patient there was a temporary residual diplopia that restored. Otherwise, both patients had an uncomplicated postoperative course.
**Conclusion**: In conclusion, reduction and reconstruction of zygomatic complex fractures can be challenging. CAD/CAM patient specific implants allow the transfer of virtual surgical planning to the patient diminishing unnecessary tissue exploration and saving surgical time.

**FA26/46—Other**
**Recurrent Sublingual Thyroglossal Duct Cyst: A Rare Case Report**
Anh Thai-Hoang Truong ^1^, Chon Thanh Ho-Nguyen ^1^, Tuan Van Nguyen ^2^ and Chanh Trung Le ^2^
*^1^* *Faculty of Dentistry, University of Medicine and Pharmacy at Ho Chi Minh city, Vietnam, Ho Chi Minh, Vietnam*
*^2^* *National Hospital of Odonto-Stomatology in Ho Chi Minh City, Ho Chi Minh City, Ho Chi Minh, Vietnam*
**Objectives**: To describe a rare case of a sublingual thyroglossal duct cyst that communicates with the foramen caecum, while emphasizing the diagnostic problems and surgical therapy associated with recurrence.
**Materials and Methods**: A 13-year-old female patient presents with a cyst located at the base of the tongue, which has recurred three times despite prior endoscopic excisions. A clinical examination, ultrasonography, and a contrast-enhanced CT scan were performed to assess lesion features. A complete excision was conducted by a transoral approach. The histopathological examination validated the diagnosis.
**Results**: V A cystic lesion in the sublingual space, and communicating with the foramen caecum, situated superior to the mylohyoid muscle and inferior to the geniohyoid muscle. A transoral approach has been used for complete excision while preserving adjacent structures. No recurrence was noted at the 8-month follow-up.
**Conclusion**: The cyst communicates with the foramen caecum and presents as a cyst at the base of the tongue; therefore, excising only the cyst at the base of the tongue results in recurrence. CT scans and ultrasounds are necessary for diagnosis. Transoral excision provides favorable outcomes while avoiding external scarring.

**FA26/275—Other**
**Beneath the Surface: Medicine Beyond the Conventional Setting**
Reema Mahmoud ^1^, Clariel Ianculovici ^1^ and Shlomi Kleinman ^1^
*^1^* *Tel Aviv Sourasky Medical Center, Tel Aviv, Israel*
**Objectives**: What happens when a modern tertiary hospital is forced underground?
**Materials and Methods**: We report a real-world institutional response from Tel Aviv Sourasky Medical Center, a tertiary referral center in central Israel, during sudden military escalation. Within less than five hours, the entire hospital was transitioned into a fully operational underground facility originally constructed as a parking structure. Approximately 1000 inpatients were evacuated from conventional wards into the subterranean complex. Clinical activity across trauma, oncology, and oral and maxillofacial surgery was continuously monitored, alongside implementation of a real-time multidisciplinary triage system to prioritize urgent surgical interventions under severe resource constraints.
**Results**: Despite the unprecedented speed and scale of the transition, critical care delivery was maintained without interruption. Emergency and urgent surgical services continued almost seamlessly, supported by full hospital infrastructure including oxygen supply, electricity, and direct access to protected operating rooms. This represented a rare demonstration of healthcare system continuity under direct threat conditions and extreme operational pressure.
However, the subterranean environment introduced significant challenges. Overcrowding, reduced privacy, infection-control difficulties, limited sanitation access, and absence of natural light contributed to substantial physical and psychological strain for both patients and healthcare personnel. Vulnerable populations required targeted relocation to protected zones, while expanded home hospitalization and accelerated outpatient evacuation pathways were implemented to reduce inpatient burden and maintain flow.
**Conclusion**: This experience demonstrates that a modern healthcare system can preserve full clinical functionality even after abrupt relocation underground during armed conflict. However, it also highlights that resilience extends beyond maintaining services, it requires sustaining patients and staff under extreme environmental stress. Ultimately, when conventional infrastructure fails, healthcare does not merely relocate; it adapts in real time, reinforcing that true system resilience is defined by coordination, flexibility, and the ability of multidisciplinary teams to sustain care when normal conditions cease to exist.

**FA26/329—Other**
**Primary Oral Leprosy Mimicking Squamous Cell Carcinoma: A Diagnostic Challenge Resolved by Next-Generation Sequencing**
Warittha Mukkhaphrom ^1^ and Amontep Mungmee ^1^
*^1^* *Khon Kaen university, Khon Kaen, Thailand*
Primary oral leprosy is an exceptionally rare clinical entity that can easily masquerade as oral squamous cell carcinoma (OSCC) or deep fungal infections. This severe clinical mimicry poses a significant diagnostic challenge, risking delayed treatment and unwarranted, mutilating oncologic surgeries. We report the case of a 75-year-old patient who presented with a progressively enlarging, symptomatic oral lesion of four months’ duration. Intraoral examination revealed a 5 × 4 cm indurated, ulcerative exophytic mass extending from the mid-hard palate to the uvula, alongside discrete skip lesions on the buccal mucosa and alveolar ridge. Given the destructive clinical appearance, OSCC was highly suspected. However, an incisional biopsy revealed severe non-caseating granulomatous inflammation densely packed with intracellular acid-fast bacilli (AFB), definitively ruling out malignancy but triggering a diagnostic mystery. Initial diagnostic investigation utilizing a PCR swab suggested an unspecified non-tuberculous mycobacterium (NTM). To achieve definitive microbiological characterization, the tissue sample was further analyzed using targeted next-generation sequencing (tNGS) alongside Genotype LepraeDR. Ultimately, these advanced genomic assays conclusively identified the pathogen as *Mycobacterium leprae*.
The patient initially responded to a multidisciplinary NTM regimen before definitively transitioning to a 12-month course of WHO multibacillary multidrug therapy (rifampicin, clofazimine, and dapsone). Remarkably, the lesion completely resolved within six months, requiring no surgical intervention. This case highlights a critical diagnostic pitfall: the non-specificity of acid-fast staining and the cross-reactivity of oral PCR samples. It underscores the vital role of advanced molecular tools like tNGS in distinguishing leprosy from SCC or NTM, ensuring timely pharmacological treatment, and sparing patients from unnecessary surgical morbidity.

**FA26/424—Other**
**Developing a Care Pathway for Assaulted Patients in the Emergency Department**
Johanna Uittamo ^1^
*^1^* *University of Helsinki, University hospital of Helsinki, Helsinki, Finland*
**Objectives**: Emergency departments (EDs) encounter the consequences of violence on a daily basis. A substantial proportion of injuries are not accidental but result from assault; for example, up to 30–40% of patients with facial fractures have sustained their injuries due to violence. Without systematic protocols, these cases are easily overlooked. Supporting victims of violence requires clear care pathways and practical tools.
**Materials and Methods**: During 2024–2025, a care pathway for assaulted patients was developed for the joint EDs of the Helsinki University Hospital (HUH). The aim was to improve identification of violence, patient encounters, documentation of injuries, and referral to support services, while also reducing the ethical burden on staff. The development process was carried out as a multiprofessional collaboration with authorities and third-sector organizations and targeted patients over 16 years of age, particularly those in vulnerable positions.
Written guidelines were produced for healthcare staff and made available at workstations and in the HUH guideline repository. A structured documentation template was implemented in the patient record system to support comprehensive recording and to prompt mandatory notifications and referrals. In addition, designated nurse coordinators were appointed in each ED to support implementation and staff training. The model was piloted at a small ED in autumn 2024 and subsequently expanded to other HUH Eds in 2025
**Results**: Challenges have included onboarding of new physicians, highlighting the importance of permanent nursing staff and clear written instructions. High-quality documentation is a key objective; in less severe cases, patient records and photographs may provide sufficient material for police without a separate medico-legal statement.
**Conclusion**: The care pathway has been positively received. Active engagement across professional groups and the central role of nurse coordinators have supported its implementation, alongside a shared commitment to improving care for vulnerable patients.
Conflict of interest disclosure
Receipt of grants/research supports: Research support from the University Hospital of Helsinki.

**FA26/492—Other**
**Carbon Footprint of Third Molar Surgery Under Local Anaesthesia Versus Total Intravenous General Anaesthesia: A Retrospective Life Cycle Assessment**
Nawal Suhaimi ^1^ and Weifa Yang ^1^
*^1^* *Oral and Maxillofacial Department, Faculty of Dentistry, The Hong Kong University, Hong Kong*
**Objectives**: To compare the carbon footprint of third molar surgery performed under local anaesthesia (LA) and total intravenous general anaesthesia (TIVA), and to evaluate the impact of staged procedures on overall emissions in a setting without volatile anaesthetic use.
**Materials and Methods:** A retrospective life cycle assessment was conducted on 40 patients undergoing third molar surgery at the Faculty of Dentistry, The University of Hong Kong, including 20 LA and 20 GA-TIVA cases. Carbon emissions were estimated per patient episode (kgCO_2_e), incorporating preoperative visits, intraoperative resource use (anaesthesia, consumables, sterilization, energy), and postoperative care including patient transport. For LA cases, a staged two-visit model was applied to reflect common clinical practice for bilateral extractions. Emission factors were derived from published healthcare life cycle assessment literature. Sensitivity analyses were performed to test key assumptions.
**Results**: The mean carbon footprint per procedure was 15.88 kgCO_2_e for GA-TIVA and 6.75 kgCO_2_e for single-session LA. When adjusted for staged bilateral procedures, the total carbon footprint for LA increased to 13.49 kgCO_2_e per patient. Despite this, GA-TIVA remained associated with higher emissions compared to LA. The increased carbon footprint in GA-TIVA was primarily driven by greater intraoperative resource use, including intravenous anaesthetic drugs, airway equipment, consumables, and higher energy utilization. In contrast, transport contributed a larger proportion of emissions in the LA group. Sensitivity analyses confirmed that these findings were robust across a range of assumptions.
**Conclusion**: Third molar surgery under total intravenous general anaesthesia is associated with a higher carbon footprint compared to local anaesthesia, even when accounting for multiple visits required for staged procedures. The difference is driven primarily by increased procedural resource use rather than anaesthetic gases. Where clinically appropriate, local anaesthesia may represent a lower-carbon alternative. These findings highlight opportunities to improve environmental sustainability in oral and maxillofacial surgery.

**FA26/503—Other**
**The Charged Tongue: A Case Report on Chemical Burns and Vascular Mimicry**
Siti Nur Nabihah Zainul Abidin ^1^ and Sooreesha Arangkulavan ^1^
*^1^* *Ministry of Health, Tanjong Karang Selangor, Malaysia*
**Objectives**: Chemical burns of the oral mucosa are frequently documented, yet injuries resulting from the intentional biting of household batteries remain rare in adult populations. This report aims to describe a unique case of an oral chemical burn caused by an AAA battery, emphasizing its clinical resemblance to a vascular lesion (hemangioma) and the critical role of patient history in avoiding unnecessary invasive interventions.
**Materials and Methods**: A 28-year-old female presented to the Tanjong Karang Hospital Department of Oral & Maxillofcaial Surgery with a one-day history of progressive tongue swelling and bluish-red discoloration. The patient admitted to biting an AAA battery to restore its function. Clinical examination revealed a 7 × 2 cm tender, soft-to-firm swelling on the left lateral and ventral surfaces of the tongue, characterized by multiple blood-filled vesicles and dysgeusia. Diagnostic protocols included a full blood workup (FBC, CRP, Coagulation Profile) and a scheduled MRI to investigate potential vascular involvement. Management consisted of a seven-day course of systemic antibiotics and analgesics to prevent secondary infection.
**Results**: Initial review at five days showed partial resolution, though symptoms temporarily recurred due to poor medication compliance. Upon completion of the full antibiotic course, the bluish discoloration, swelling, and lymphadenopathy completely resolved within 14 days. Follow-up at two months confirmed the restoration of normal tongue function, sensation, and morphology. Due to the rapid clinical resolution, the scheduled MRI was deemed unnecessary and cancelled.
**Conclusion**: This case highlights the “vascular mimicry” of chemical burns, where localized tissue trauma and hematoma can clinically simulate a hemangioma. It underscores the necessity for dental practitioners to maintain a high index of suspicion and perform diligent history-taking. Early diagnosis and conservative therapeutic measures are effective in ensuring rapid healing and preventing long-term mucosal deformity or neurological deficit.

**2FA26/1041—Other**
**Developing an AI-Based Tool for Objective Assessment of Microsurgical Anastomosis**
Muhammad Adnan Shakeel ^1^, Faith Xin Rei Sze ^2^, Nelson Ming Hao Teo ^1^, Elijah Cai ^1^, Alphonsus Chong ^1^, Siti Yusoff ^1^, Yan Lin Yap ^1^, Jane Lim ^1^, Priya Tiwari ^3^, O-Wern Low ^3^, Vigneswaran Nallathamby ^3^, Thiam Chye Lim ^1^ and Janet Ngai Man Hung ^1^
*^1^* *National University Hospital, Singapore, Singapore*
*^2^* *Lee Kong Chian School of Medicine Nanyang Technological University, Singapore, Singapore*
*^3^* *Ng Teng Fong General Hospital, Singapore, Singapore*
**Objectives**: To develop Anastomosis Coach, a machine-learning based system for automated, objective assessment of microsurgical anastomosis. Microanastomosis training requires repeated feedback to achieve proficiency, but conventional faculty-dependent assessment is resource-intensive and subject to variability. This study aims to develop a scalable image-based assessment tool capable of providing structured feedback for microsurgical skill acquisition.
**Materials and Methods**: A deep-learning regression model was developed using transfer learning with an EfficientNet-B0 architecture pretrained on ImageNet. A dataset of 200 microsurgical suturing images was annotated by a plastic surgeon using the Manual Suture Parameters for Training and Assessment (M-SParTA) scoring system. It is selected for its objective, visually identifiable scoring domains and strong inter-rater reliability. M-SParTA comprises six domains (ALIGN, ANGLE, AREA, YY_DIST, LENGTH, MISSING) and a TOTAL SCORE. Images were divided into training (80%) and test (20%) sets. The model was trained to predict individual domain scores, with TOTAL SCORE calculated as their sum. Performance was evaluated on the test set using Pearson correlation coefficient (r).
**Results**: The model demonstrated strong agreement with expert assessment, achieving a Pearson correlation coefficient of 0.88 for TOTAL SCORE. At the domain level, ANGLE (r = 0.73) and LENGTH (r = 0.59) showed the strongest correlations. Lower correlations were observed for AREA (r = 0.30), ALIGN (r = 0.09), and YY_DIST (r = −0.22) as the model was trained to recognise overall suture strip patterns rather than individual suture points. Correlation for MISSING could not be calculated because all test images received an expert score of 0, resulting in insufficient variability for Pearson correlation analysis. Despite variability in individual domains, the model accurately predicted overall anastomosis quality.
**Conclusion**: Anastomosis Coach demonstrated strong agreement with expert scoring and provided objective, domain-specific assessment of microanastomosis from a single image. With larger datasets and further validation, it may serve as a scalable adjunct for microsurgical training and competency assessment.

**2FA26/1147—Other**
**Deceptive Presentation of Aggressive Organised Haematoma: Clinical Identification Challenge**
Abdullah Anas bin Hussin ^1,2^, Mohammed Amin bin Mohammed Jelani ^1^, Saravanan Gopalan ^1^ and Eb Robson Gubod ^1^
*^1^* *Queen Elizabeth Hospital, Kota Kinabalu, Malaysia*
*^2^* *Shah Alam Hospital, Shah Alam, Malaysia*
**Objectives**: To report a rare case of an organised haematoma (OH) of the maxillary sinus presenting as a post-exodontia complication and highlight the importance of distinguishing its locally aggressive features from malignancies to avoid unnecessary radical resections.
**Materials and Methods**: A 47-year-old male presented with a two-year history of persistent right cheek swelling following the extraction of tooth 16, which rapidly expanded after recent blunt trauma. Diagnostic assessments included clinical examination, blood aspiration, ultrasonography, and a Contrast-Enhanced Computed Tomography (CECT) scan. Multiple incisional biopsies were performed to rule out haematological and solid malignancies before definitive surgical intervention
**Results**: Clinical examination revealed a tender, soft bucco-palatal expansion at the 16 region with tissue overgrowth and localized tooth mobility. The CECT scan demonstrated an aggressive, hyperdense cyst-like growth occupying half of the right maxillary sinus with associated bony erosion of the sinus wall, mimicking a malignant tumor. Following a multi-disciplinary team review, total surgical enucleation and primary closure were successfully performed. The final histopathological examination (HPE) confirmed an organised haematoma characterized by fibrous tissue, blood clot, and chronic inflammatory cells, with no neoplastic features.
**Conclusion**: Organised haematomas can mimic sinonasal malignancies radiographically and clinically due to their bone-resorbing, expansile nature. Oral surgeons and dentists should include OH in their differential diagnoses for slow-growing post-extraction swellings with bony changes. Comprehensive histopathological validation is crucial to ensure conservative, complete surgical enucleation instead of highly morbid, extensive tissue resections.

**2FA26/1167—Other**
**A Rare Case of Orbital Schwannoma**
Todrol Davaadorj ^1^
*^1^* *First Central Hospital, Ulaanbaatar, Mongolia*
**Objectives**: To present a rare case of orbital schwannoma causing compressive optic neuropathy and to highlight the importance of early diagnosis and complete surgical excision in achieving favorable visual and functional outcomes.
**Materials and Methods**: A 44-year-old woman with progressive unilateral proptosis and visual impairment underwent comprehensive ophthalmic examination and orbital computed tomography. A posterior orbital mass compressing the optic nerve was identified. Complete surgical excision was performed through an infrabrow deep orbitotomy under general anesthesia. Histopathological examination with hematoxylin and eosin staining was used to establish the final diagnosis. Postoperative clinical outcomes were assessed during follow-up visits.
**Results**: A 44-year-old woman presented with progressive left-sided proptosis, diplopia, and visual deterioration. Preoperative examination revealed visual acuity of 6/60, positive RAPD, impaired color vision (2/14 Ishihara plates), and 22 mm of proptosis. CT imaging demonstrated a well-defined orbital mass measuring 4.1 × 2.2 × 2.3 cm compressing the optic nerve. Complete surgical excision was successfully performed through an infrabrow deep orbitotomy. Histopathological examination confirmed the diagnosis of orbital schwannoma. At one month postoperatively, visual acuity improved to 6/10 (BCVA 6/8.5), RAPD resolved, color vision normalized (14/14 Ishihara plates), ocular motility was fully restored, and proptosis decreased from 22 mm to 16 mm. No postoperative complications or evidence of recurrence were observed.
**Conclusion**: Orbital schwannoma is a rare benign orbital tumor that may present with progressive proptosis and compressive optic neuropathy. Early diagnosis and complete surgical excision can achieve excellent visual and functional outcomes. Long-term follow-up remains essential because of the potential risk of recurrence.

**2FA26/1202—Other**
**Craniofacial Phenotype, Management, and Outcomes in First and Second Branchial Arch Syndromes: A Scoping Review**
Mohd Hazwan Mohd Ghazali ^1^, Su Keng Tan ^1^, Shah Jumaat Mohd Yusoff ^1^, Xia Zhou ^2^ and Hazmyr Abdul Wahab ^1^
*^1^* *University Teknologi MARA, Sungai Buloh, Malaysia*
*^2^* *Peking University School and Hospital of Stomatology, Beijing, China*
**Objectives**: This scoping review aimed to systematically map the existing evidence on craniomaxillofacial phenotypes, treatment approaches, clinical outcomes, and psychosocial factors associated with First and Second Branchial Arch Syndromes (FASBAS).
**Materials and Methods**: A scoping review was conducted per the Arksey and O’Malley framework and reported as per PRISMA-ScR guidelines. Electronic searches were conducted in PubMed (MEDLINE), Scopus, and Web of Science for studies published between January 2020 and December 2025. Eligibility criteria were peer-reviewed studies with human participants diagnosed with FASBAS and reporting clinical phenotypes, treatment outcomes, or psychosocial findings. Title or abstract screening and full text assessment were carried out by two independent reviewers, with data arranged in a standardized extraction form.
**Results**: A total of 13 studies fulfilled the inclusion criteria. Presently, mandibular hypoplasia, condylar hypoplasia, zygomatic asymmetry, and microtia were the most often reported phenotypic features. The severity classification was applied inconsistently with the OMENS/OMENS+ and Pruzansky–Kaban systems deployed across some studies. Mostly surgical treatment was divided into a stage-based approach, featuring mandibular distraction osteogenesis and orthognathic surgery as main processes. Post-operatively significant functional improvements were recorded but residual asymmetry and growth-related revision burden lingered. Psychosocial consequences, like behavioral challenges and caregiver distress, have been reported in both quantitative and qualitative studies, but there are no condition-specific patient-reported outcome measures for this population.
**Conclusion**: This review found significant heterogeneity in the phenotypic classification and management pathways for FASBAS as well as a persistent deficit in psychosocial assessment and patient-centered care tools. These findings highlight the need for standardized outcome measures and a condition specific framework to support holistic, patient-centered care in this population.

**2FA26/1222—Other**
**Psychological Impacts of maxillofacial surgery**
Nor Adilah Harun ^1^, Khairul Bariah Chi Adam ^1^, Sofea Hanin Mohd Faisal ^1^ and Nur Iffah Aqilah Khairul Hazamy1 ^1^
*^1^* *International islamic University Malaysia, Kuantan, Malaysia*
**Objectives**: Maxillofacial surgery patients have proven to sustain psychological impacts, namely anxiety, depression, and post-traumatic stress disorder. Current research has mostly focused on the clinical and functional results of the surgery, with little consideration given to the psychological well-being of the patients. This study analyses the immediate and delayed psychological impact in minor and major maxillofacial surgeries, to compare the difference of psychological impacts between both surgeries, and to identify the need for mental health interventions for patients undergoing maxillofacial surgeries.
**Materials and Methods**: This hospital-based longitudinal observational study included 25 adult patients undergoing minor (n = 10) and major (n = 15) maxillofacial surgery. Psychological status was assessed using the Hospital Anxiety and Depression Scale (HADS) at baseline (T0), one week (T1), and one month (T2) postoperatively. Data were analysed using SPSS version 29.0, and group comparisons were performed using the Friedman test with a significance level of *p* < 0.05.
**Results**: Across all assessed time points, the minor surgery group remained entirely stable within the normal psychological category. Conversely, the major surgery group exhibited a shift toward borderline and abnormal psychological distress, particularly at one-week post-operation (T1). Consequently, 20% of major surgery patients warranted psychology department referrals (13.3% borderline, 6.7% abnormal), whereas no minor surgery patients required referrals. Despite this numerical increase in distress, the difference in mean HADS scores between the groups was not statistically significant (*p* = 0.549)
**Conclusion**: Minor maxillofacial surgeries are not associated with significant psychological distress. Major procedures may induce transient distress early postoperatively, which tends to stabilize over time. Although the overall statistical difference between groups was insignificant, the distress observed in major surgery patients highlights the necessity of incorporating routine psychological screening to enhance comprehensive perioperative care. Further studies with larger sample sizes are recommended to clarify this relationship.

**2FA26/1254—Other**
**Microbiological Profile in Head and Neck Odontogenic Infections. A Retrospective Study**
Konstantinos Katoumas ^1^, Eleni Spiliopoulou ^1^, Marios Kourmoulis ^2^, George Chrisochoou ^2^ and Christos Perisanidis ^1^
*^1^* *Department of Oral and Maxillofacial Surgery, Dental School, National and Kapodistrian University of Athens, Athens, Greece*
*^2^* *Dental School, National and Kapodistrian University of Athens, Athens, Greece*
**Objectives**: The purpose of this retrospective study is to assess the microbiological profile of odontogenic head and neck infections in a tertiary hospital quantifying the prevalence of poly-microbial infections and identifying the most frequently isolated pathogens. Routine systemic biomarkers in admission are also evaluated.
**Materials and Methods**: A retrospective cohort study was conducted among patients hospitalized with head and neck odontogenic infections in a tertiary hospital the year before covid (2019). Data regarding inflammatory markers obtained by routine blood test upon admission, microbiological results from exudate or pus cultures obtained during surgical drainage and the antibiotics prescribed during hospitalization were analysed. Statistical analysis was performed using SPSS Statistics, IBM for MAC.
**Results**: A total of 140 patients, 75 males (53.57%) and 65 females (46.42%) were included. Age ranged from 14 to 93 years old [mean 44.52 (±17.52), median 43 years]. The average length of stay (LOS) was 4 days [mean 4.02 (±2.46), median 4.00 days]. Cultures were obtained in 69 patients with isolates ranging from 1 to 6 per patient, revealing strong predominance of polymicrobial (mixed flora) aetiology [mean number of isolates per patient 2.80 (±1.53), median 3.00]. The most prevalent microorganisms were Prevotella sp., and Clostridium sp. each isolated in 25 patients (36.23%). Staphylococcus epidermidis was isolated in 21 patients (30.43%) and Streptococcus mitis/oralis isolated in 20 patients (28.99%). In 78.26% of the cases infections were polymicrobial with co-isolation of Clostridium sp and Prevotella sp in 15 patients (21.74% of all cultures). Mean LOS was 4.46 (±2.16) in Polymicrobial infections and 3.80 (±1.97) days in monomicrobial infections however, the increased LOS for polymicrobial infections is not statistically significant [*p* = 0.17, 95% CI (−0.55, +1.88 days)].
**Conclusion**: The epidemiology of odontogenic infections in a tertiary hospital is presented. In 78.26% of the obtained cultures multiple microorganisms were identified

**2FA26/1294—Other**
**Gender-Affirming Facial Feminization Procedures in Southern India population**
Dinesh Kadam ^1^
*^1^* *A J Institute of Medical Sciences, Mangalore, India*
**Objectives**: Over the past decade, the number of gender affirmation procedures has been on the rise with increased awareness and favourable legislation. Though the genital and chest procedures are widely performed now, facial feminisation is far less common due to expertise and complexities. We present our approach in our population of Southern India
**Materials and Methods**: Transgender individuals who underwent genital surgery and were on hormonal supplements and who sought facial procedures were included. The procedures were either done in a single stage or in two stages for the upper and lower face. Procedures for the upper face include: frontal bone and sinus reduction, supraorbital margin reduction, eyebrow lift through a bicorneal approach. Feminising rhinoplasty and lower jaw procedures include mandibular angle and lower border reduction and genioplasty.
**Results**: Eleven patients underwent full face procedures with satisfactory outcomes. The techniques will be discussed with short videos. No unfavourable outcomes noted.
**Conclusion**: Facial feminisation is an essential step towards the complete transgender affirmation procedure. Applying principles and various techniques determines the outcome. It is still an evolving field with a rapid increase in procedures.

